# Morphological, chemical and electrophysiological investigations of *Telchin licus* (Lepidoptera: Castniidae)

**DOI:** 10.1371/journal.pone.0231689

**Published:** 2020-04-16

**Authors:** Merybeth F. Triana, Paulo H. B. França, Abel F. O. Queiroz, Jakeline M. Santos, Henrique F. Goulart, Antônio E. G. Santana

**Affiliations:** 1 Chemistry and Biotechnology Institute, Federal University of Alagoas, Maceió, Alagoas, Brazil; 2 Agricultural Science Center, Federal University of Alagoas, Maceió, Alagoas, Brazil; INRA-UPMC, FRANCE

## Abstract

The giant sugarcane borer *Telchin licus* (Drury, 1773) (Lepidoptera: Castniidae) is a day-flying moth pest of sugarcane, pineapples and bananas. To better understand the chemical communication in this species, we examined the morphology of its olfactory system and the chemical composition of its body parts. The ventral surface of the clubbed antennae of *T*. *licus* has six morphological types of sensilla: sensilla trichodea, basiconica, chaetica, squamiforma, coeloconica, and auricillica. The telescopic ovipositor shows no evidence of a sexual gland, or female-specific compounds. On the other hand, the midleg basitarsus of males releases (*E*,*Z*)-2,13-octadecadienol and (*Z*,*E*)-2,13-octadecadienol, which are electroantennographically active in both sexes. These compounds are known female sex pheromones in the Sesiidae family and are male-specific compounds in another castniid moth, although further investigations are necessary to elucidate their ecological role in the Castniidae family.

## Introduction

The giant sugarcane borer *Telchin licus* (Drury, 1773) (Lepidoptera: Castniidae) is distributed throughout Central and South America [1–6]. Its larvae feed on the stems and rhizomes of sugarcane, pineapple and banana [7,8], causing severe damage in sugarcane crops [6,9].

Castniidae occur in the Neotropical region, Southeast Asia and Australia. The species present in Asia and Australia are commonly known as "sun moths", while the species that reside in the Neotropical regions are often referred to as “butterfly moths” [10,11]. Castniid moths have day-flying activity and usually exhibit features that resemble those of butterflies, such as clubbed antennae; apposition eyes; broad, colored wings and slender bodies [12–14].

Currently, Castniidae are part of the Sesioidea superfamily, forming a clade related to the Sesiidae and Brachodidae families [15,16]. However, because of differing opinions about the exact relationship between Sesiidae and Castniidae, the systematic position of the Castniidae family is not clearly resolved [17].

The reproductive behavior of Lepidoptera is largely related to diurnal or nocturnal habit. Sexual chemical communication in moths usually involves the release of long-range sex pheromones by females to attract conspecific males. Then, when close enough to the female, males release short-range pheromones that act as an aphrodisiac to facilitate copulation [18,19]. Day-flying butterflies, on the other hand, make extensive use of visual cues to attract the opposite sex, with males releasing short-range pheromones when near females [20–22].

Similar to butterflies, moths from the Castniidae family are thought to utilize visual and chemical signals [13,14,23]. Previous studies reported that females of *Paysandisia archon* (Burmeister, 1880) (Lepidoptera: Castniidae) have lost the gland responsible for the production of long-range sex pheromone [13,22,24], and releasing structures have been identified in males of *P*. *archon* [25–27].

Few investigations aimed at elucidating the mechanisms underlying chemical communication in *T*. *licus* have been carried out. Rebouças et al. (1999) hypothesized the existence of a putative pheromone released by female ovipositors since hexane extracts elicited male responses in bioassays [28]. However, one preliminary ethological study suggested that visual and chemical cues are needed for mating in *T*. *licus* [23].

This investigation aims to contribute to our understanding of chemical communication in the Castniidae family through characterization of the sensilla on the antennae and scent-producing organs in *T*. *licus*, in addition to the identification of sex-specific electrophysiologically active compounds in female and male specimens.

## Materials and methods

### Ethics statement

The permit granting access to genetic patrimony and associated traditional knowledge in Brazil was granted by the Brazilian Ministry of Environment-SISGEN and CGEN (no. 010428/2012-7).

### Insect collection and maintenance

*T*. *licus* larvae and pupae were collected during 2015–2019 from infested sugarcane crops at the Triunfo Agroindustrial Mill in Boca da Mata (9°38'27.6°S, 36°13'12"W) and the Santo Antônio Mill in São Luís do Quitunde (9°19'5.7"S, 35°33'39.2"W), which are both in the state of Alagoas, Brazil. The larvae were kept individually in Petri dishes and fed sugar cane until reaching the pupal stage, at which point they were sexed [29] and placed in trays containing sugarcane straw inside cages (2 m x 1 m x 0.6 m) maintained at room temperature (27.2 ± 0.4°C, 81 ± 3% relative humidity (RH)). The adults were fed a 10% honey solution.

### Scanning electron microscopy

Scanning electron microscopy images were used to describe the antennal sensilla, as well as to observe the legs of male *T*. *licus*. The antennae and legs were removed from one- to two-day-old cold-anesthetized (-20°C) males and females. Immediately after removal, the legs were immersed in 2% glutaraldehyde in phosphate buffer solution (0.1 M, pH 7) for five min and then dehydrated in a gradient series of ethanol (50%, 70%, 80% and 90%, 10 min each). They were metallized with gold at 10 mA for 8 min (Sanyu Electron, Mini Quick Coater SC701, Tokyo, Japan). Antennae and tarsi were observed using a scanning electron microscope (Shimadzu SSX-550 Superscan, Tokyo, Japan) at 12 kV. The number of sensilla was quantified in a 100 μm^2^ area on the club of five antennae per sex. The differences in antenna, scape, pedicel, and antennal sensillum size and flagellomere and sensillum number in five male and five female antennae were analyzed using an independent-sample t-test (p < 0.05) in R software (v. 3.4.1).

### Light microscopy

We used light microscopy to study the anatomy and histology of the ovipositor, focusing on the intersegmental membrane between the eighth and ninth abdominal segments, where the pheromone gland is usually located in moths. After females were cold anesthetized, the ovipositor was extracted and fixed with Bouin’s solution for 24 hours at room temperature, followed by dehydration in a gradient ethanol series. The organs were incorporated into paraffin wax using xylene as a transition solvent, and 5 μm sections were obtained using a microtome (Leica RM2125 RTS, Buffalo Grove, United States). Finally, the slices were mounted on glass slides, hydrated and stained with hematoxylin-eosin to be observed under a light microscope (Olympus BX41, Center Valley, United States). Digital images were recorded using a high-resolution digital camera (Olympus DP25, Center Valley, United States).

### Compound extraction

Extracts of the whole thorax, abdomen, forewings, hindwings, legs (three pairs), and genitalia were obtained from three one- to two-day-old virgin adults for each sex. Each organ was separated from the body of the previously cold-anesthetized insects and immersed in hexane (high-performance liquid chromatography (HPLC) grade, Sigma-Aldrich®) for 30 min. The volume used for each extract was adjusted to cover the entire sample (2 mL for the thorax, abdomen, and wing extracts and 200–500 μL for the other extracts). All extracts were then purified by filtration on a silica gel column and concentrated with flowing nitrogen or adjusted with hexane to 200 μL. By using the previous methodology, one further extraction of whole forelegs, midlegs and hindlegs, followed by extraction in triplicate of the femur, tibia, basitarsus, tarsus, and claw (all from the midlegs), was performed.

### Electroantennogram recordings

The electroantennographic (EAG) responses of antennae from one- to two-day-old males and females to the body extracts were measured, using hexane as a negative control. The last flagellomeres of antennae removed from previously cold-anesthetized insects were cut and then placed on Ag electrodes containing Spectra 360 conductive gel (Parker Laboratories Inc., Hellendoorn, Netherlands). The club of the antenna was subsequently positioned perpendicularly to the air flow from the capillary. Then, a 10 μL (equivalent to 0.15 of an insect) sample from each treatment was placed on filter paper (1 cm x 2 cm, Whatman No. 1) inside a glass pipette, and EAG recordings were obtained from one insect per treatment (each antenna received seven stimuli). The magnitude of the antennal response was measured using Syntech EagPro 2.0.2 (Syntech, Hilversum, Netherlands), and air flux (30 mL/s) was generated with a Syntech CS-55 controller (Syntech, Hilversum, Netherlands) with a 0.5 s pulse duration. We measured the responses of 7 independent antennae for each sex. Amplification of the electrical signals was performed with a high-impedance amplifier (IDAC-4, Syntech Hilversum, Netherlands), while data were analyzed using the Syntech EagPro 8 program (Syntech, Hilversum, Netherlands). EAG responses to extracts were expressed relative to responses to the negative control (hexane) because of the large differences in overall sensitivity between individual antennae. In this normalization procedure, the responses to the control were defined as 100%. A Mann-Whitney test (p< 0.05) performed in R software (v. 3.4.1) was used to determine whether normalized EAG responses to extracts differed from responses to the control. One antenna of an insect was used for each test.

### Chromatographic analysis

Gas chromatography coupled to mass spectrometry (GC-MS) was used to elucidate the structure of the compounds present in the extracts. A gas chromatograph coupled to a mass spectrometer (Shimadzu QP2010 Ultra, Kyoto, Japan) equipped with an Rtx-5 column (30 m x 0.25 mm x 0.25 μm, Restek®) was used under the following chromatographic conditions: 50°C (5 min hold), followed by heating at 15°C/min until reaching 220°C and heating at 4°C/min to 250°C (20 min hold). Then, 1 μL of each extract was injected in splitless mode at 250°C, with helium as the carrier gas (1.5 mL/min). The mass spectrometer was operated with electron impact ionization (70 eV) in scan mode between 35 and 500 *m/z*. The ion source remained constant at 200°C, and the interface remained constant at 250°C. The identification of compounds was performed by comparing each mass spectrum and Kovats index with those of authentic standards and/or by comparison with the NIST08, NIST08s and WILEY229 databases.

The midleg extracts divided into femur, tibia, basitarsus, tarsus and claw extracts (N = 3) were analyzed by GC with flame ionization detection (GC-FID) in a chromatograph (GC-2010 Shimadzu, Kyoto, Japan) equipped with an Rtx-5 column (30 m x 0.25 mm x 0.25 μm, Restek®). The temperature program was from 100°C to 250°C at 15°C/min (5 min hold). Then, 1 μL of the standard solution was injected in splitless mode at 250°C, using hydrogen as the carrier gas, with a flow rate of 1.0 mL/min. The ratio of (*E*,Z)-2,13-octadecadienol (E2,Z13-18:OH) to (*Z*,*E*)-2,13-octadecadienol (Z2,E13-18:OH) in the leg and hindwing extracts was quantified using a calibration curve (E2,Z13-18:OH, 0.1 μg– 1.0 μg– 10.0 μg– 100.0 μg—500 μg). Midleg extracts as well as E2,Z13-18:OH and Z2,E13-18:OH solutions (200 μg/mL) were analyzed in an Rtx-Wax column (30 m x 0.25 mm x 0.25 μm, Restek®). The temperature program was from 120°C to 240°C at 8°C/min (5 min hold). Then, 1 μL of the standard solution was injected in splitless mode at 240°C, using hydrogen as the carrier gas, with a flow rate of 1.0 mL/min.

Midleg extract was derivatized at 40°C overnight in a mixture of dimethyl disulfide (DMDS) (50 μL) and tetrahydrofuran (THF) solution with iodine (60 mg/mL, 5 μL). The resulting DMDS adducts were analyzed by GC-MS using the abovementioned chromatographic parameters.

GC coupled to electroantennography (GC-EAD) was used to analyze the electrophysiological activity of male hindwing and midleg extracts and synthetic Z2,E13-18:OH and E2,Z13-18:OH solutions (200 μg/mL for standards) against those of antennae from one- to two-day-old insects (N = 3). A gas chromatograph (Schimadzu, GC-2010, Kyoto, Japan) equipped with an Rtx-5 column (30 m x 0.25 mm x 0.25 μm, Restek®) was used for the analyses. Three microliters of sample was injected. The same chromatographic parameters described above for GC-FID runs were used for GC-EAD analyses. The column effluent was split with a Y-connector into two 0.25 mm ID branches, with one branch towards the FID at 300°C and the other towards a heated transfer line (200°C) with a connection to the capillary glass releaser. The effluent from the capillary glass was diluted with humidified air. Antennae were prepared following the same methodology used for EAG recordings, and data were analyzed using the Syntech GC-EAD32 program (version 4.6, 2008; Syntech, Hilversum, Netherlands).

### Standards

The linear (C7-C30) hydrocarbons, squalene, nonanal, (*E*)-2-nonenal, (*E*)-2-decenal, decanal, (*E*,*Z*)-2,4-decadienal, nonanol, dodecanol, tetradecanol, hexadecanol, octadecanol, docosanol, nonanoic acid, dodecanoic acid, myristic acid, palmitoleic acid, palmitic acid, linoleic acid, oleic acid, and geranyl acetone were purchased from Sigma-Aldrich, and 2,13-octadecadienol diastereomers were kindly donated by Dr. Wittko Francke from the University of Hamburg.

## Results

### Morphological analysis of antennae

**Fig 1:**
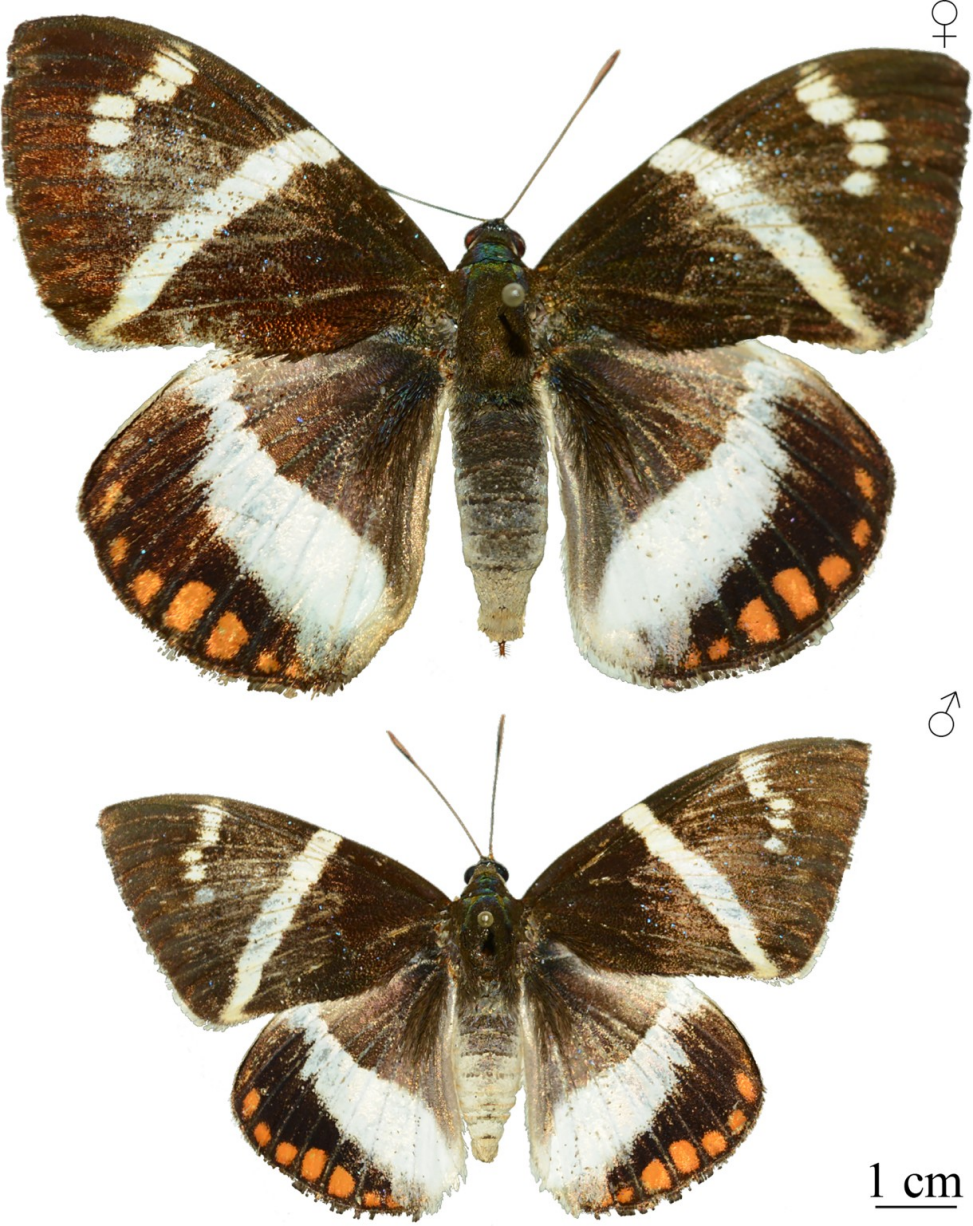
The giant sugarcane borer (*Telchin licus*). Female and male *T*. *licus* in dorsal view. The apparent difference in their sizes in this illustration is a particularity of these two individuals and does not reflect a trend for the species.

**Fig 2:**
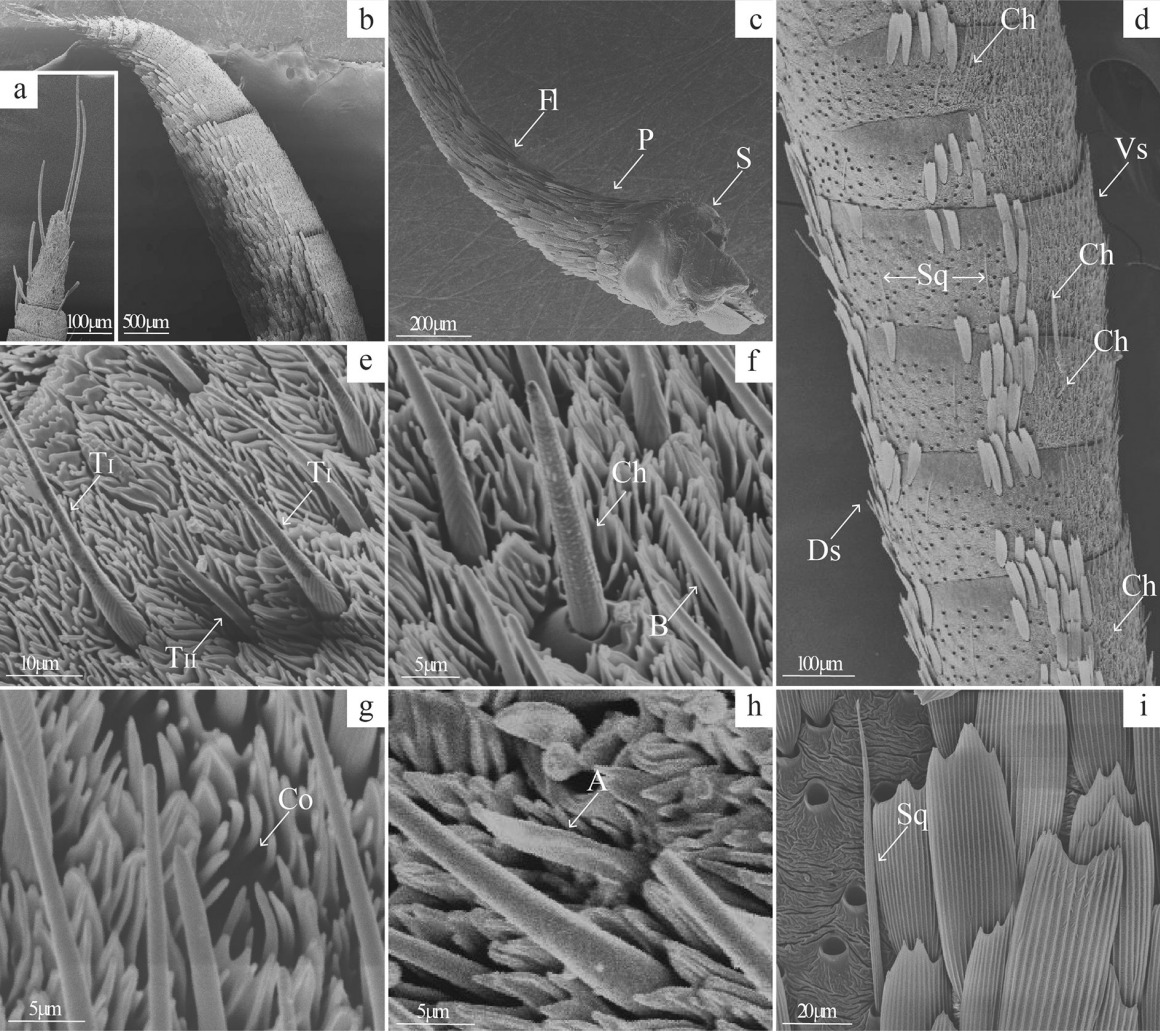
General morphology of male and female *Telchin licus* antennae. Microphotographs showing the (a) apiculus; (b) club; (c) antenna base with a scape (S), a pedicel (P) and the first segments of the flagellum (F1); and (d) flagellum composed of ventral (Vs) and dorsal (Ds) surfaces with squamiform (Sq) and chaetic (Ch) sensilla. (e) Trichoid sensilla (TI, TII). (f) Basiconic sensilla (B) and chaetic sensilla (Ch). (g) Coeloconic sensilla (Co). (h) Auricillic sensilla (A). (i) Squamiform sensilla (Sq).

The antennae of both sexes of *T*. *licus* are clubbed (Fig 1) and consist of three segments, namely, the scape, pedicel and flagellum, with the latter composed of 62–69 flagellomeres that end in an apiculus (Fig 2A and 2B). The surfaces of the scape, pedicel and first flagellomeres are covered with overlapping scales (Fig 2C). The antennae and scapes of males are longer than those of females (p < 0.05; Table 1). The flagellomeres are subcylindrical, gradually flattening towards the clubbed region, and equal in number between the sexes. The last flagellomere is more elongated and forms the apiculus with the largest trichoid sensillum on the antenna.

**Table 1:** General characteristics of male and female *Telchin licus* antennae.

Antenna length (mm)	Scape		Pedicel		No. flagellomeres
	Length (mm)	Width (mm)	Length (mm)	Width (mm)	
18.2 ± 0.3	0.5 ± 0.0	0.7 ± 0.1	0.3 ± 0.0	0.3 ± 0.0	67± 2
19.8 ± 0.3*	0.8 ± 0.1*	0.8 ± 0.1	0.3 ± 0.0	0.4 ± 0.1*	64± 2

Values are presented as the means ± SEMs.

* within a column indicates a significant difference between the sexes (p < 0.05; independent-sample t-test, N = 5 per sex).

The flagellum’s dorsal surface is covered with scales, and some squamiform sensilla are spread between the scales (Fig 2D). In contrast, an abundance of sensilla can be found on the ventral surface. The length of the flagellar segments decreases from the base to the apex (from 375.6 ± 1.1 μm between the 5^th^ and 20^th^ flagellomeres to 54.7 ± 0.6 μm in the last five segments, N = 5), while the width is more prominent in the clubbed area (625.9 ± 27.8 μm in females and 757.1 ± 19.9 μm in males between the 45^th^ and 50^th^ flagellomeres, N = 5 per sex).

Six morphological types of sensilla were observed on the ventral surface of the flagellomeres in female and male *T*. *licus*, namely, trichoid, basiconic, chaetic, squamiform, coeloconic and auricillic sensilla. Trichoid sensilla are the most abundant type of sensilla on the antennae of *T*. *licus*. We observed two subtypes, trichodea I and trichodea II (T_I_ and T_II_), according to the substructure of the surface and the length (Fig 2E). The T_I_ sensilla are distributed across the ventral surface, except for the first segments of the antenna. They exhibit a round base with a long cylindrical axis that decreases towards the tip, becoming slightly curved towards the apex. Their surface has loop-shaped annular grooves inclined towards the tip (Fig 2E). In addition, the T_I_ sensillum length differs between sexes, measuring 46.4 ± 0.8 μm in females and 43.5 ± 1.1 μm in males (p < 0.05, Table 2). T_II_ sensilla are less abundant than T_I_ sensilla and initially distributed between the 18^th^ and 20^th^ flagellomeres, reaching maximum density in the clubbed area between the 40^th^ and 50^th^ flagellomeres. Their morphological features include a smooth cuticular surface and a cylindrical axis. For comparison purposes, the length of T_I_ sensilla is approximately three times that of T_II_ sensilla (Table 2, Fig 2).

**Table 2:** Morphological features of male and female *Telchin licus* antennal sensilla.

Sensillum type	Sex	Length (μm)	Basal width (μm)	No. sensilla/100 μm^2^
**S. trichodea I**	♀	46.4 ± 0.8*	4.3 ± 0.1	20.7 ± 2.4
	♂	43.5 ± 1.1	4.3 ± 0.2	20.0 ± 2.1
**S. trichodea II**	♀	17.1 ± 0.7	2.6 ± 0.1*	6.7 ± 0.3
	♂	16.7 ± 0.5	2.1 ± 0.1	7.7 ± 1.2
**S. basiconica**	♀	27.8 ± 0.7	3.0 ± 0.1	6.0 ± 0.6
	♂	26.8 ± 0.7	2.8 ± 0.1	4.3 ± 0.7
**S. chaetica**	♀	38.7 ± 2.3*	9.9 ± 0.3	1.0 ± 0.0
	♂	29.3 ± 1.6	9.6 ± 0.3	1.0 ± 0.0
**S. squamiforma**	♀	77.4 ± 2.0	3.6 ± 0.1	1.0 ± 0.0
	♂	71.9 ± 2.1	3.3 ± 0.2	1.0 ± 0.0
**S. coeloconica**	♀	-	11.1 ± 0.8	5.3 ± 1.2
	♂	-	11.0 ± 0.4	6.0 ± 1.5
**S. auricillica**	♀	11.1 ± 0.3	2.5 ± 0.2	3.7 ± 1.2
	♂	13.8 ± 0.3*	2.7 ± 0.1	3.7 ± 0.9

Values are presented as the means ± SEMs.

* within a column indicates a significant difference between the sexes (independent-sample t-test at a 5% probability, N = 12 per sensillum type). The number of sensilla was quantified in 100 μm^2^ areas on the club (N = 5 per sex).

The basiconic sensilla (Fig 2F) appear to have the same distribution as the T_II_ sensilla. The axis is cylindrical with a conical end and is slightly curved towards the tip of the antenna. No sexual dimorphism was observed for these sensilla.

One to three chaetic sensilla are distributed on the flagellum from the 14^th^ to 16^th^ antennal segment (Fig 2D). These sensilla have a sharp, pointed shape and a broad base and are surrounded by a round collar, typically vertical or with a slope of 60° towards the surface of the antenna. The length is slightly shorter than that of T_I_ sensilla, and the cuticle is rough with longitudinal grooves (Fig 2F). These sensilla are longer in females than in males (38.7 ± 2.3 μm in females and 29.3 ± 1.6 μm in males, p <0.05, Table 2).

One to six coeloconic sensilla in each flagellar segment are mainly distributed in the clubbed area of the antenna and composed of 11–14 small, smooth, spiked structures with a circular base that surrounds a central sensillum (Fig 2G).

The auricillic sensilla are less abundant and spread mainly along the sides of the flagellomeres around the scales. These sensilla are bent towards the apex and are almost parallel to the surface of the antenna. They have a smooth cuticular surface with the appearance of a leaf (Fig 2H) and are larger on the male antenna than on the female antenna (11.1 ± 0.3 μm in females and 13.8 ± 0.3 μm in males, p <0.05; Table 2).

Squamiform sensilla are distributed on the dorsal surface of the flagellum and are covered with scales (Fig 2D). They are elongated and flat with grooves arranged longitudinally, similar to the texture of the scales, but shorter and wider (Fig 2I).

### Morphohistological analysis of the ovipositor

*T*. *licus* has a telescopic ovipositor formed by the last three abdominal segments. The eighth highly sclerotized uromere (length: 3.27 ± 0.04 mm, N = 4) forms the base of the ovipositor, while the ninth and tenth uromeres are fused (length: 3.26 ± 0.05 mm, N = 4). The apical part forms the anal papillae, which in turn are characterized by an abundance of long sensilla (length: 655 ± 5 μm, N = 12). The ninth and tenth uromeres are connected to the eighth by an intersegmental membrane (length: 3.24 ± 0.03 mm, N = 4).

**Fig 3:**
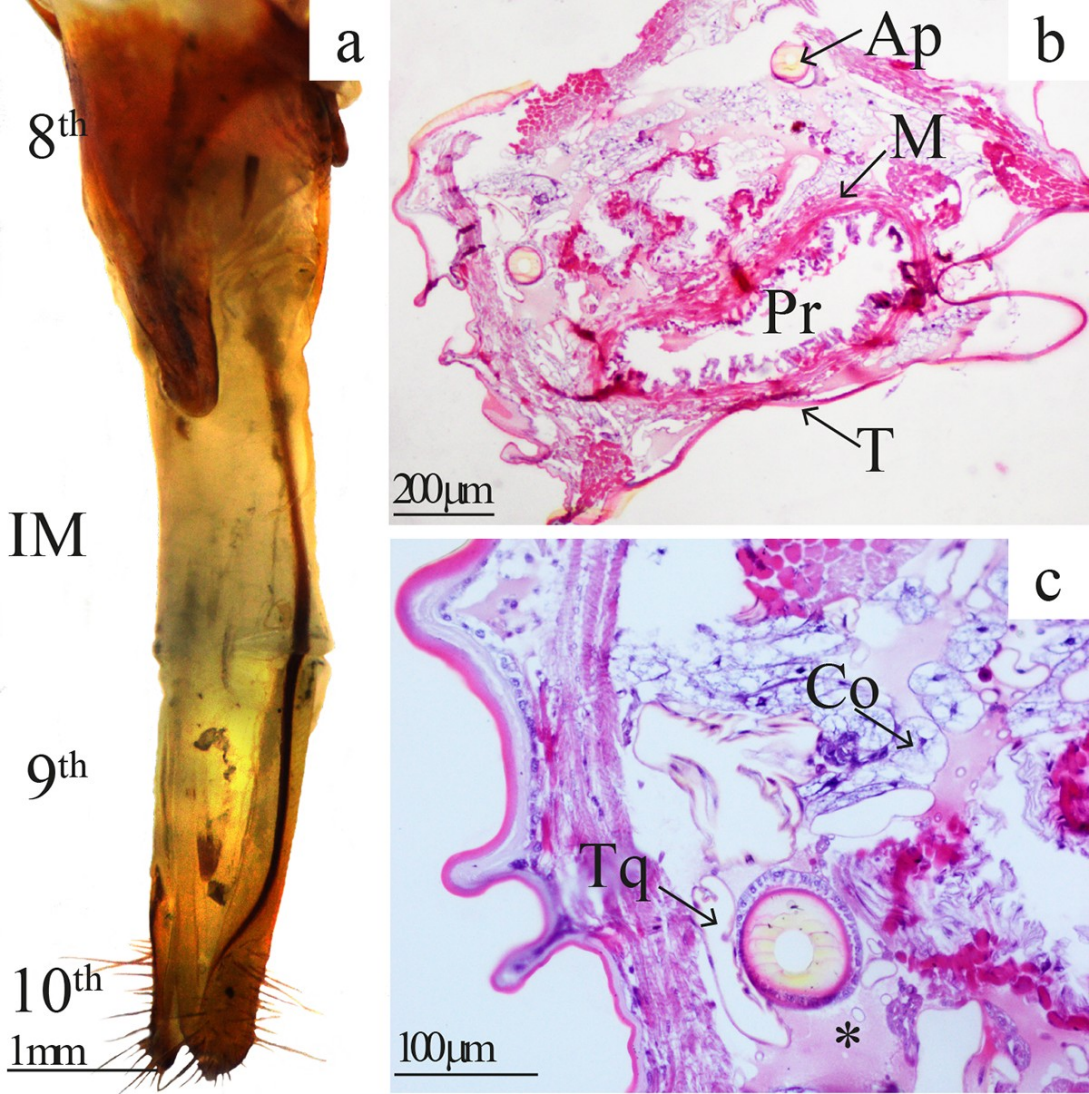
Morphohistological features of the ovipositor of *Telchin licus*. (a) Dorsolateral view of the ovipositor of *T*. *licus* showing the 8^th^ uromere, intersegmental membrane (IM), and 9^th^ and 10^th^ uromeres. (b) Cross-section of the IM showing the tegument (T), muscles (M), proctodeum (Pr) and apodemes (Ap). (c) Enlargement of the cross-sectional area of the IM showing the trachea (Tq), connective tissue (Co) and hyaline eosinophilic fluid (*).

The entire surface of the ovipositor is smooth, and a pair of apophyses appears laterally from the anal papillae and fuses to the intersegmental membrane up to the eighth uromere (Fig 3A). In the resting position, the ovipositor is completely retracted under the abdomen. Histological sections of the intersegmental membrane show a tegument from a thick cuticle on a single layer of cuboidal epithelial cells resting on a thin basement membrane. The apodemes are surrounded by a layer of cubic epithelium followed by muscle tissue. The proctodeum is also surrounded by muscle cells. Furthermore, the intersegmental membrane contains trachea and a large amount of connective tissue surrounded by hyaline eosinophilic fluid (Fig 3B and 3C).

### Chemical composition of *T*. *licus* body extracts

Saturated and monounsaturated hydrocarbons, mainly in the form of long chains (>20 carbons), are the major compounds in the body extracts of females and males of *T*. *licus*. These hydrocarbons account for 56% and 64% of the total volatile constituents of extracts from females and males, respectively. Saturated, mono- and diunsaturated aldehydes with chains of 8 to 26 carbons were identified with relative abundances of 10% in females and 2% in males. In addition, we managed to identify saturated alcohols with chains ranging from 9 to 26 carbons in length, Z2,E13-18:OH and E2,Z13-18:OH, mostly on the male legs. Seven carboxylic acids, two ketones, two esters and three terpenoids were the minor components of the extracts. A total of 76 compounds were identified (S1 Table).

**Fig 4:**
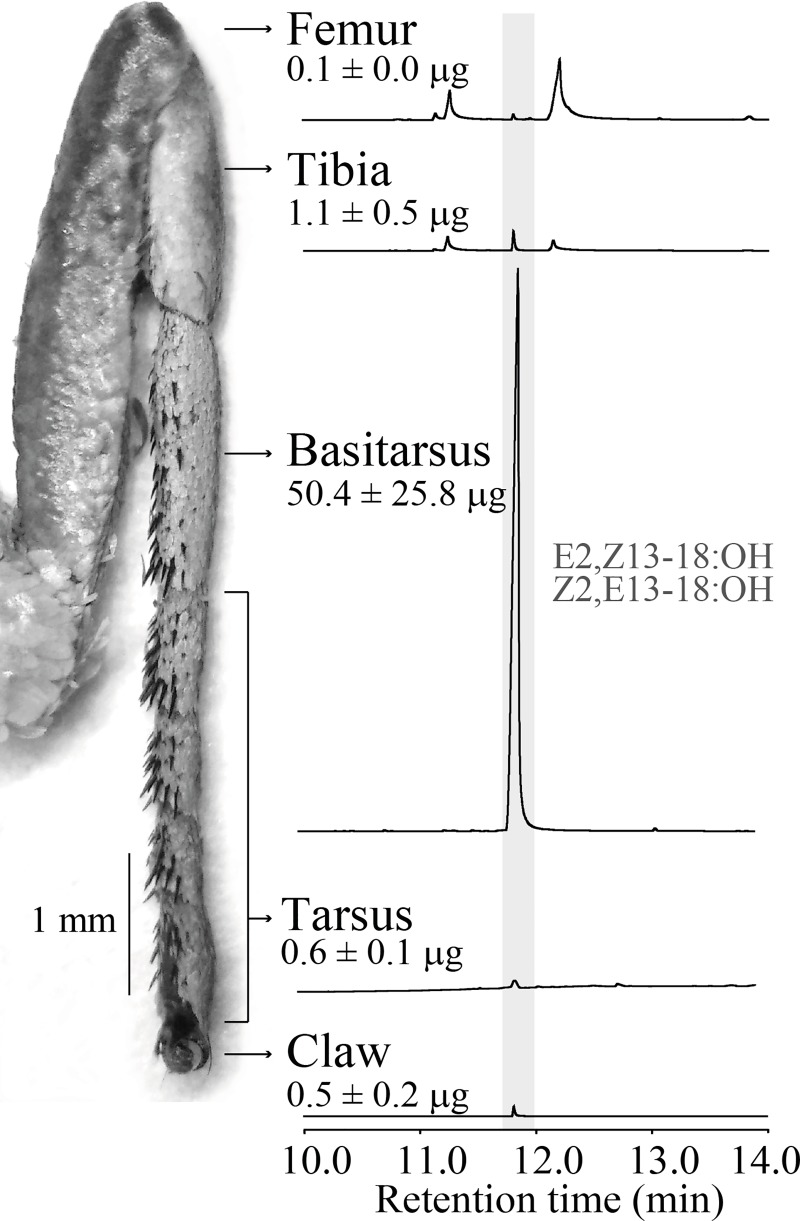
Chromatogram of midleg segments of male *Telchin licus*. Chromatogram showing the presence of Z2,E13-18:OH and E2,Z13-18:OH (highlighted) and the amount of the diastereoisomer mixture in each midleg segment per male (means ± SEMs). GC-FID equipped with an Rtx-5 capillary column.

The mixture of Z2,E13-18:OH and E2,Z13-18:OH was identified at a higher concentration in the midleg extract of males (S1 Fig), more specifically from the midleg basitarsus (Fig 4). The total quantity of these compounds in the basitarsus extracts was calculated to be 50.4 ± 25.8 μg/male (N = 3). Morphologically, the midleg basitarsus of males and females differed significantly in length (3.3 ± 0.0 μm in females and 3.8 ± 0.0 μm in males, p<0.05, N = 5) and in width (0.6 ± 0.0 μm in females and 0.8 ± 0.0 μm in males, p< 0.05, N = 5) (S2 Fig).

**Fig 5:**
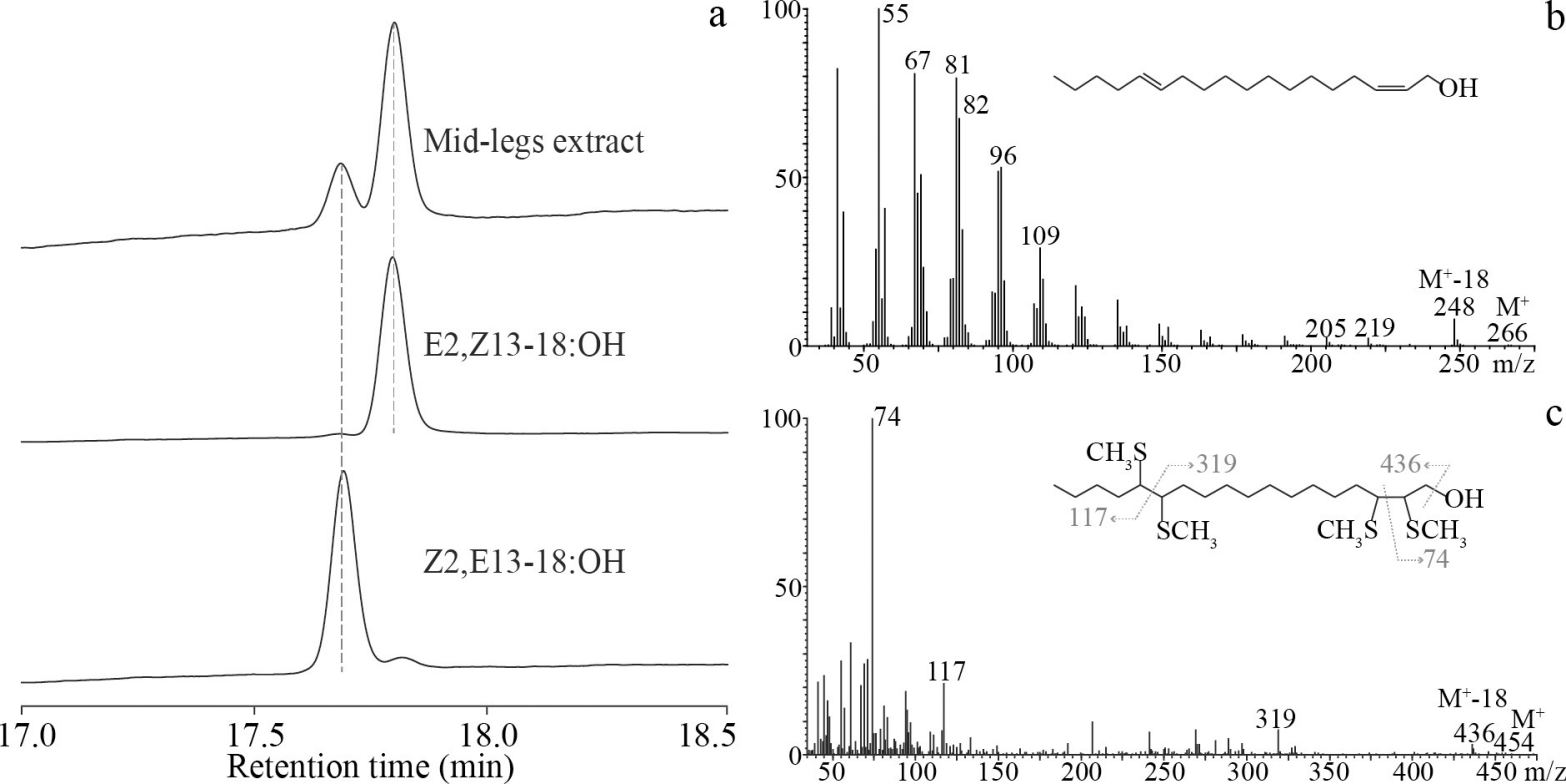
Configuration assignment of 2,13-octadecadienol in the midlegs of male *Telchin licus*. (a) Chromatograms of the midleg extract of male *T*. *licus*, Z2,E13-18:OH and E2,Z13-18:OH. GC-FID equipped with an Rtx-Wax capillary column. (b) Mass spectra (EI) of (*Z*,*E*)/(*E*,*Z*) 2,13-octadecadienol in male midlegs and (c) corresponding DMDS adducts. Each spectrum shows diagnostic fragment ions, and the adducts show the fragment ions produced by cleavage of the bond between the sulfur-substituted carbons, indicating positions 2 and 13 of the double bonds. GC-MS equipped with an Rtx-5 capillary column.

Coelution of Z2,E13-18:OH and E2,Z13-18:OH was observed in the low-polarity column (Rtx-5); however, in the polar column (Rtx-Wax), they could be differentiated (Table 3). Additionally, DMDS derivatives of Z2,E13-18:OH and E2,Z13-18:OH standards were analyzed in a low-polarity column (Rtx-5), and their retention times did not differ. In the basitarsus extracts of male midlegs, these alcohols were found as a mixture with a 20:80 ratio (Fig 5A).

**Table 3:** Kovats indices for synthetic and natural 2,13-octadecadienol diastereoisomers in columns with different polarities.

	Rtx-5	Rtx-Wax
**E2,E13-18OH**	2080	2666
**Z2,E13-18OH**	2083	2665
**E2,Z13-18OH**	2083	2672
**Z2,Z13-18OH**	2085	2671
**Midlegs**	2084	2664
		2672

The mass spectrum of E2,Z13-18:OH in the midlegs showed a small molecular ion peak of *m/z* 266 (0.1% M^+^) and an *m/z* 248 ion peak (5.4%) corresponding to the loss of a water molecule [M^+^-18]. The base peak corresponds to the cation [CH_2_CH(CH_2_)_2_]^+^ of *m/z* 55 (100%). The molecule undergoes rearrangement and the migration of hydrogen, leading to the formation of a series of homologous [CH_2_(CH)_3_(CH_2_)_n_]^+^ cations that generate *m/z* 109 (29.0%), *m/z* 95 (51.8%), *m/z* 81 (79.4%) and *m/z* 67 (80.7%) ions. Moreover, dehydration leads to the formation of a three-double bond fragment series of homologous [CH_2_(CH)_4_(CH_2_)_n_]^+^ cations, resulting in *m/z* 233 (0.3%), *m/z* 219 (2.2%), and *m/z* 205 (2.3%) and further sequential losses of methylene radicals (Fig 5B). The analysis of DMDS adducts from the extract revealed the fragments *m/z* 74 (100%), *m/z* 117 (21.3%) and *m/z* 319 (7.5%), which in turn confirmed the position of the double bond at C(2) and C(13) in 2,13-octadecadienol (Fig 5C).

### EAG analysis

**Fig 6:**
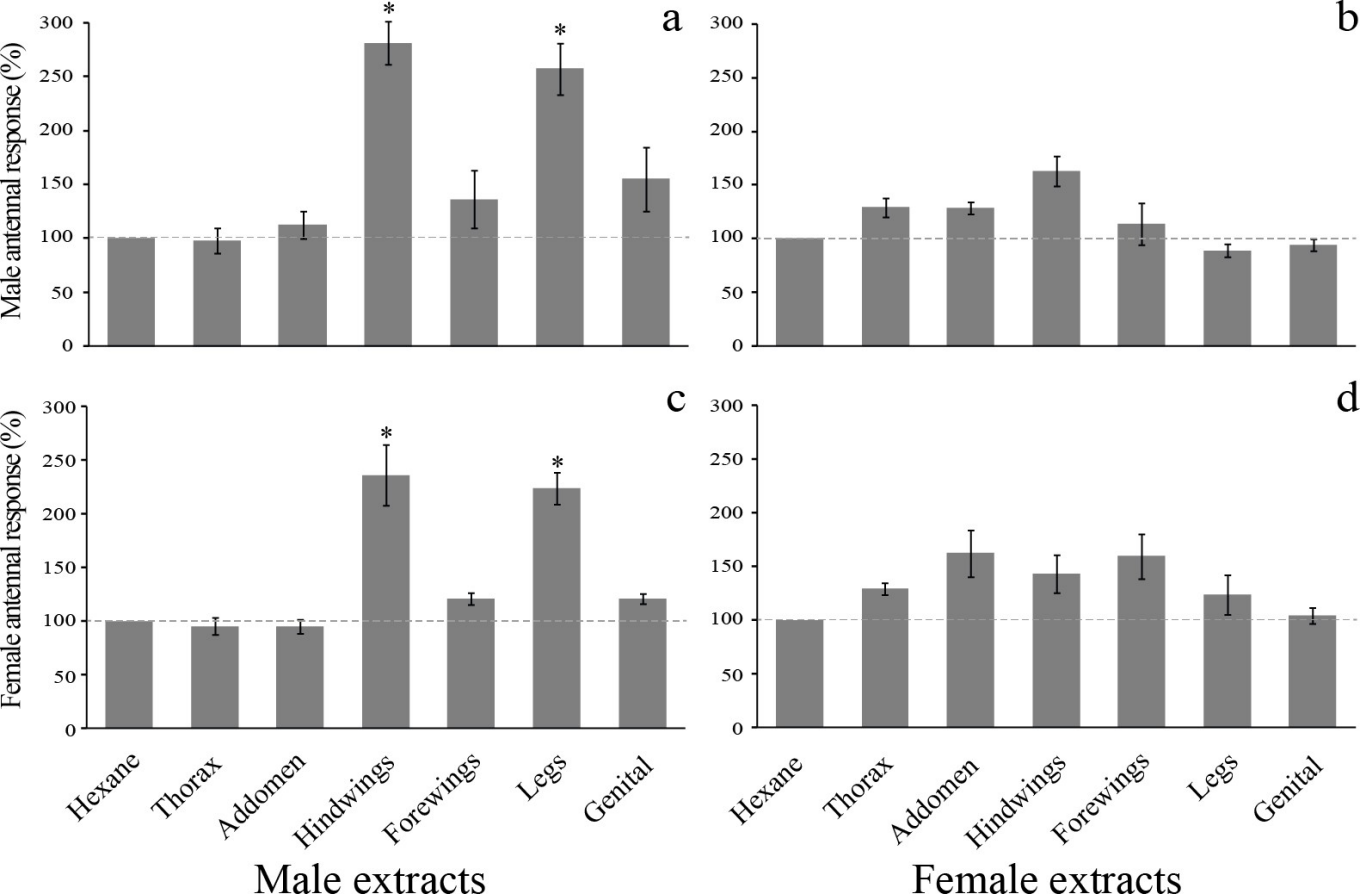
EAG responses to body extracts of *Telchin licus*. Mean (± SEM) EAG responses [expressed as the mean percentage values with respect to the control (hexane = 100%)] for the antennae of male *T*. *licus* to (a) male body extracts and (b) female body extracts and for the antennae of female *T*. *licus* to (c) male body extracts and (d) female body extracts. Bars with (*) are significantly different relative to hexane (p< 0.05; Mann-Whitney test; N = 7 antennae of each sex).

Female and male antennae showed significantly different EAG responses to extracts from the hindwings and legs of *T*. *licus* males when compared to the control (0.39 ± 0.04 mV in males and 0.27 ± 0.03 mV in females in response to male hindwing extract and 0.34 ± 0.02 mV in males and 0.23 ± 0.04 mV in females in response to male leg extract, p< 0.05, N = 7 per sex). In contrast, female body extracts did not trigger responses with significant differences in the antennae of either sex when compared to the negative control (hexane) (Fig 6).

**Fig 7:**
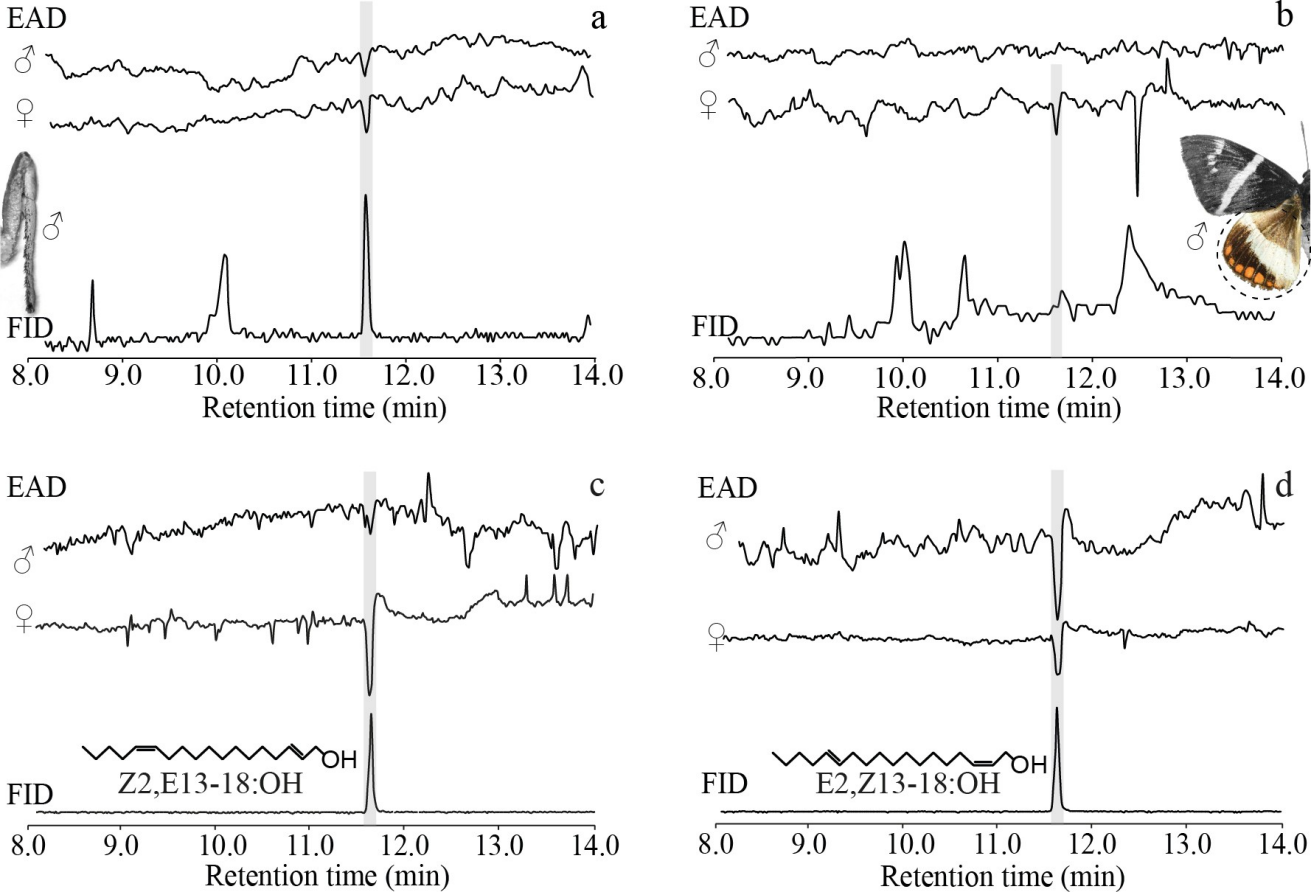
Gas chromatographic analyses of extracts and synthetic references using simultaneous detection with FID and EAD. (a) Male midlegs. (b) Male hindwings. (c) Z2,E13-18:OH. (d) E2,Z13-18:OH. GC-EAD equipped with an Rtx-5 capillary column; 3 antennae were used for each extract or standard.

Analysis by GC-EAD showed that only Z2,E13-18:OH or E2,Z13-18:OH in the male midlegs has constant EAG activity in female and male antennae. However, the low concentration of only Z2,E13-18:OH or E2,Z13-18:OH in male hindwings (0.3 ± 0.1 μg per male, N = 3) elicited a response only in females (Fig 7A and 7B). Additionally, the diastereoisomers Z2,E13-18:OH and E2,Z13-18:OH showed EAG activity in both sexes (Fig 7C and 7D). The EAG response at 12.5 min to the male hindwing extract corresponded to oleic acid, but this was not consistent among replicates (Fig 7B).

## Discussion

Lepidoptera mating behavior relies on the perception of odorant molecules. The main olfactory appendage responsible for the detection of such chemical signals are the antennae. Our morphological analyses verified that the antennae of *T*. *licus* are similar to the antennae of other moths in Castniidae and Sesiidae [12,13,30–33], as well as to those of butterflies in Hesperiidae and Papilionidae [34,35]. The external features of the antennae are believed to have two functions: provide a protective barrier for the sensilla [36], and to trap odorant molecules [37]. Insects use specialized cleaning structures to groom their antennae in order to enhance olfactory acuity [24,38]–the epiphyses found on the forelegs of *T*. *licus* might carry out the same function (S2 Fig).

In this study, we identified six types of sensilla on the antennae of both female and male *T*. *licus*, some of which might be involved in the reception of pheromone cues. Trichodea sensilla were the most abundant type found on the *T*. *licus* antennae and this type of sensilla has been observed in the recently evolved species within lepidopteran phylogeny that use type I and type II sex pheromones in contrast to more basal species that use pheromones with plant volatile-like chemical structures [39].

The morphological differences observed between the antennae of female and male *T*. *licus* are subtle, suggesting that *T*. *licus* has a generalist olfactory system. Other communication signals used by castniids, such as visual cues, may allow a less complex olfaction system than that required in moths with crepuscular or nocturnal habits, as these moths rely heavily on smell to find food and oviposition sites [40] and, especially, to detect low concentrations of sex pheromones.

In moths, sex pheromones are usually produced in a specialized gland commonly located between the eighth and ninth abdominal segments in the intersegmental membrane. During calling behavior, this glandular area is exposed, and the pheromone is released [41]. However, current literature suggests that female Castniidae species investigated thus far, which include only *T*. *licus*, *Eupalamides cyparissias* (Fabricius, 1776) and *P*. *archon*, appear to lack calling behavior. Riolo et al. (2014) observed ovipositor extrusion in *P*. *archon* but concluded that this behavior might be involved in egg loading or thermoregulatory activity.

The ovipositor surface of *T*. *licus* does not exhibit structural modifications (protuberances or grooves) in the intersegmental membrane, a common feature of moth pheromone glands [42,43]. In our histological investigation, we found no evidence of glandular cells. Moreover, in the limited number of studies examining evidence for gland-releasing pheromones in Castniidae, histological and morphological analyses of the ovipositor of *P*. *archon* revealed the absence of glandular cells [13,24].

Other scent-producing organs have been described in the abdomen, thorax, legs, and wings of lepidopteran males, ranging from simple scales and tufts of hair to complex eversible structures [44]. These volatile compounds are often released when the male is close to a female, suggesting to play a key role in courtship behavior [44]. The castniid males studied to date possess several androconia, mostly located on the abdomen and legs [45].

With the exception of the genitals, all body parts of *T*. *licus* males contained a mixture of E2,Z13-18:OH and Z2,E13-18:OH and nearly 93% of the total content was distributed in the abdomen, wings and legs. Quero et al. (2017) speculated that E2,Z13-18:OH in *P*. *archon* is spread by the brush-like paronychia located at the distal tip of the midlegs. Similar structures were observed in the legs of *T*. *licus* males (S2 Fig). These compounds could therefore be released by the midleg basitarsus at high concentrations (50.4 ± 25.8 μg/male) and transferred by contact to other parts of the body.

Regarding chemical communication in the Zygaenoidea-Cossoidea-Sesioidea (ZCS) clade, which includes Castniidae [15,16], the Δ2, Δ13-unsaturation pattern has been described among the sex pheromones of Sesiidae and Cossidae [46–52]. Nevertheless, these compounds are produced by the females [13,25]. In this case, as in many others, conservation of the biosynthetic machinery used to produce the same compounds that serve similar functions, including intraspecific communication, is not restricted to a single sex [53]. In fact, some compounds utilized as type I female sex pheromones in moths are the same compounds used in butterflies, albeit produced by males where they serve a slightly different biological function [54].

The phylogeny of the ZCS clade remains unraveled however recent studies indicate that the Sesiidae and Castniidae families are closely related [16,55]. The fact that these two families might use compounds with similar chemical structures could provide additional evidence to support their close relationship. Furthermore, E2,Z13-18:OH has been described as a minor component of the female sex pheromone in other moth species including the tineid *Tineola bisselliella* (Hummel, 1823) [51,56]. This raises the question of whether basal tineid moths are common ancestors of Sesiidae and Castniidae or perhaps this shared biochemical trait is a result of convergence. Since the number of moths in these families with reported pheromone is limited, further pheromone characterization in combination with phylogenomics would be illuminating.

Finally, Sarto et al. (2012) speculated that E2,Z13-18:OH could act as a male sex pheromone while being released during courtship flight of *P*. *archon*, thus inducing acceptance by the female. However, further behavioral observations raised other possible ecological roles of this compound, such as scent marking to establish territorial boundaries or host marking [26].

## Conclusion

In our search for evidence of chemical communication in *T*. *licus*, we obtained morphological data that revealed a large number of trichoid sensilla on both male and female individuals. The histological data showed no presence of a specialized scent-producing gland in the ovipositor. The results obtained here confirm that male-specific compounds identified in another castniid moth, *P*. *archon*, are also emitted and recognized by the antennae of both sexes of *T*. *licus*. The function of the sensilla and whether E2,Z13-18:OH and Z2,E13-18:OH play an ecological role in castniid moths remain to be elucidated by further investigations, which would shed light on the olfactory cues used in chemical communication by this family.

## Supporting information

S1 Fig Chromatograms of *Telchin licus* male legs GC-FID equipped with an Rtx-5 capillary column. (TIF)

S2 Fig Legs of *Telchin licus* (a) Size comparison of the midleg basitarsus between males and females. (b) Epiphyses of the foreleg. (b) Close-up of the 5th tarsomere of the (c) foreleg, (d) midleg and (e) hindleg showing the arolium (A), claws (Cl) and pulvilli (Pv).
(TIF)

S1 File Total ion chromatograms and mass spectra of *Telchin licus* female body extracts GC-MS equipped with an Rtx-5 capillary column. (PDF)

S2 File Total ion chromatograms and mass spectra of *Telchin licus* male body extracts GC-MS equipped with an Rtx-5 capillary column. (PDF)

S1 Table Composition of *Telchin licus* male and female body extracts. (DOCX)
