# Supplementary figures and images for: Morphological, chemical and electrophysiological investigations of Telchin licus (Lepidoptera: Castniidae)
Source: PLoS One. 2020 Apr 16;15(4):e0231689. doi: 10.1371/journal.pone.0231689 (PMC7162514; doi:10.1371/journal.pone.0231689)

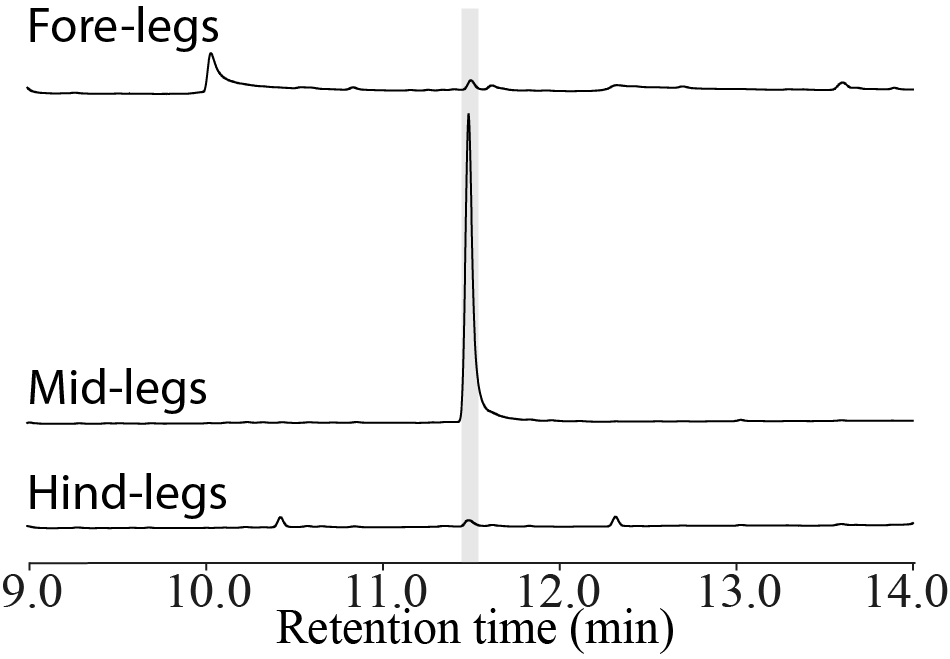

Supplement: S1 Fig — (TIF) [file pone.0231689.s001.tif]

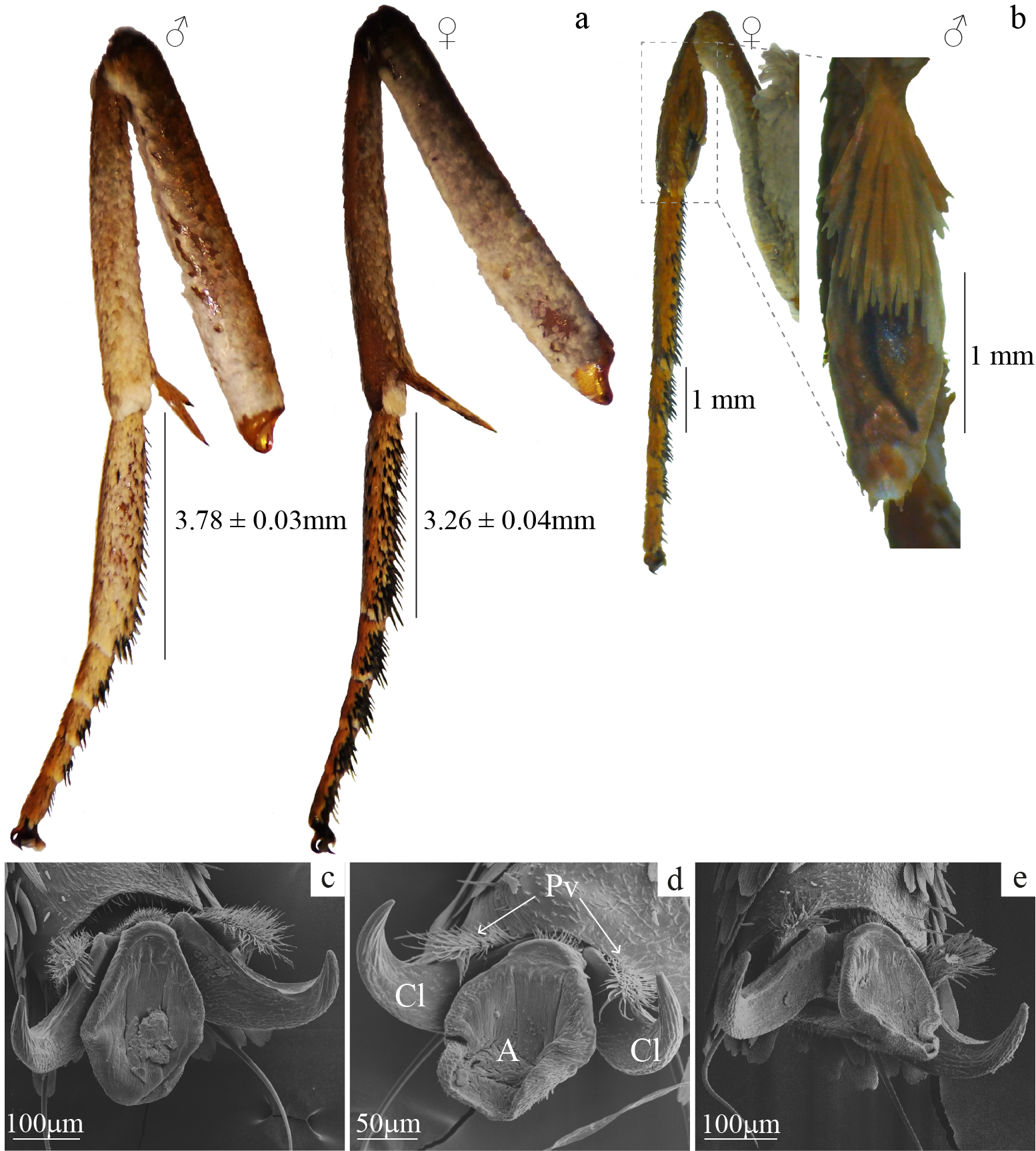

Supplement: S2 Fig — Legs of Telchin licus (a) Size comparison of the midleg basitarsus between males and females. (b) Epiphyses of the foreleg. (b) Close-up of the 5th tarsomere of the (c) foreleg, (d) midleg and (e) hindleg showing the arolium (A), claws (Cl) and pulvilli (Pv). (TIF) [file pone.0231689.s002.tif]
