# Supplementary material for: Morphological, chemical and electrophysiological investigations of Telchin licus (Lepidoptera: Castniidae)
Source: PLoS One. 2020 Apr 16;15(4):e0231689. doi: 10.1371/journal.pone.0231689 (PMC7162514; doi:10.1371/journal.pone.0231689)

Female abdomen extract

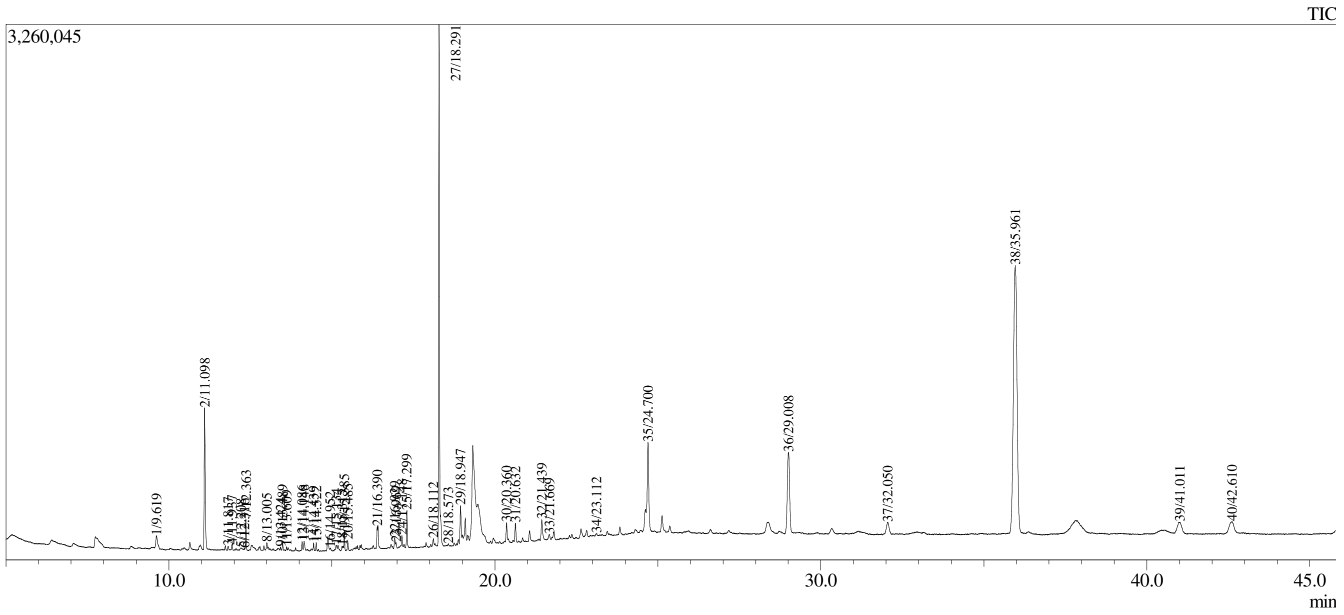

Peak Report TIC

Peak#	R.Time	I.Time	F.Time	Area	Area%	Height	Height%	A/H	Mark	Name
1	9.619	9.555	9.725	330841	1.08	79792	0.87	4.15	V	Octanal
2	11.098	11.055	11.190	1949157	6.38	862331	9.45	2.26		Nonanal
3	11.817	11.785	11.870	49391	0.16	23847	0.26	2.07		2-Nonenal
4	11.937	11.870	11.985	67451	0.22	33580	0.37	2.01	V	Nonanol
5	12.208	12.170	12.225	28831	0.09	12297	0.13	2.34	MI	2-Decanona
6	12.316	12.295	12.330	10791	0.04	8929	0.10	1.21	MI	Dodecane
7	12.363	12.335	12.415	298903	0.98	187110	2.05	1.60	V	Decanal
8	13.005	12.955	13.030	84047	0.28	45970	0.50	1.83	V	2-Decenal
9	13.424	13.400	13.450	25682	0.08	19337	0.21	1.33		Tridecane
10	13.489	13.450	13.525	80026	0.26	53247	0.58	1.50	V	Undecanal
11	13.609	13.580	13.635	32404	0.11	21858	0.24	1.48	MI	Decadienal
12	14.086	14.060	14.115	98401	0.32	58208	0.64	1.69	V	Undecenal
13	14.148	14.115	14.195	143122	0.47	59936	0.66	2.39	V	Undecanol
14	14.439	14.390	14.475	103198	0.34	51707	0.57	2.00	V	Tetradecane
15	14.522	14.475	14.555	95942	0.31	53323	0.58	1.80	V	Dodecanal
16	14.952	14.930	14.995	38058	0.12	17882	0.20	2.13	V	Geranyl acetone
17	15.134	15.110	15.160	43788	0.14	24000	0.26	1.82	MI	Dodecanol
18	15.315	15.260	15.330	55143	0.18	29625	0.32	1.86		Pentadecene
19	15.385	15.360	15.420	177968	0.58	124031	1.36	1.43	V	Pentadecane
20	15.485	15.455	15.535	104437	0.34	63830	0.70	1.64	MI	Tridecanal
21	16.390	16.360	16.400	184477	0.60	133596	1.46	1.38	MI	Tetradecanal
22	16.929	16.885	16.955	132909	0.43	64327	0.71	2.07		Tetradecenal
23	16.969	16.955	17.010	60685	0.20	35008	0.38	1.73	V	Tetradecanol
24	17.148	17.095	17.180	169233	0.55	76184	0.84	2.22	V	2-pentadecanona
25	17.299	17.250	17.380	393725	1.29	227488	2.49	1.73	V	Pentadecanal
26	18.112	18.065	18.145	114922	0.38	48627	0.53	2.36	V	Hexadecenal
27	18.291	18.210	18.425	5916180	19.36	3176117	34.81	1.86	SV	Hexadecanal
28	18.573	18.555	18.600	14972	0.05	10253	0.11	1.46	MI	Trimethyl tridecatrienal
29	18.947	18.905	18.985	574610	1.88	240198	2.63	2.39	V	Hexadecanol
30	20.360	20.320	20.400	281058	0.92	122776	1.35	2.29	V	Octadecenal
31	20.632	20.585	20.690	275682	0.90	119590	1.31	2.31	V	Octadecanal
32	21.439	21.395	21.535	357121	1.17	124864	1.37	2.86		Octadecanol
33	21.669	21.615	21.720	81307	0.27	28471	0.31	2.86		Heneicosane
34	23.112	23.065	23.155	40933	0.13	14551	0.16	2.81	V	Docosane
35	24.700	24.640	24.810	1883461	6.16	545535	5.98	3.45	V	Tricosane
36	29.008	28.890	29.120	2289349	7.49	490738	5.38	4.67		Pentacosane
37	32.050	31.960	32.160	385780	1.26	69371	0.76	5.56		Hexacosane
38	35.961	35.780	36.150	12302489	40.26	1628147	17.85	7.56		Heptacosane
39	41.011	40.840	41.165	655140	2.14	70119	0.77	9.34		Octacosane
40	42.610	42.450	42.765	627064	2.05	66749	0.73	9.39		Squalene
				30558678	100.00	9123549	100.00			

Spectrum

Line#:1 R.Time:9.620(Scan#:925)
MassPeaks:239
RawMode:Averaged 9.615-9.625(924-926) BasePeak:43(8034)
BG Mode:Calc. from Peak Group 1 - Event 1

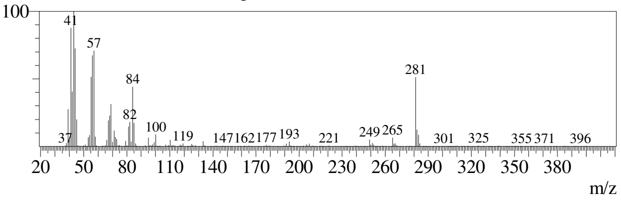

Line#:2 R.Time:11.095(Scan#:1220)
MassPeaks:252
RawMode:Averaged 11.090-11.100(1219-1221) BasePeak:57(96025)
BG Mode:Calc. from Peak Group 1 - Event 1

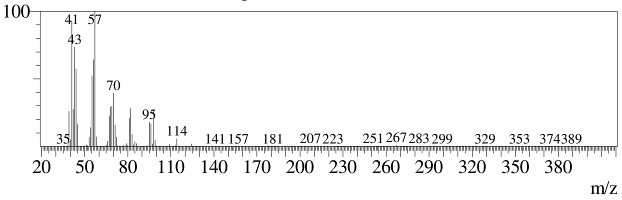

Line#:3 R.Time:11.815(Scan#:1364)
MassPeaks:205
RawMode:Averaged 11.810-11.820(1363-1365) BasePeak:43(2310)
BG Mode:Calc. from Peak Group 1 - Event 1

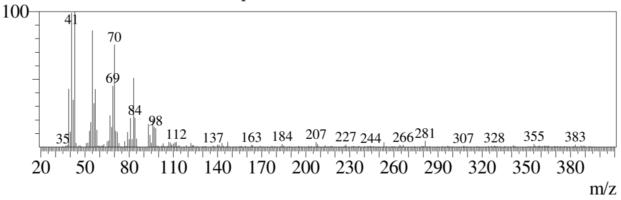

Line#:4 R.Time:11.935(Scan#:1388)
MassPeaks:177
RawMode:Averaged 11.930-11.940(1387-1389) BasePeak:43(3648)
BG Mode:Calc. from Peak Group 1 - Event 1

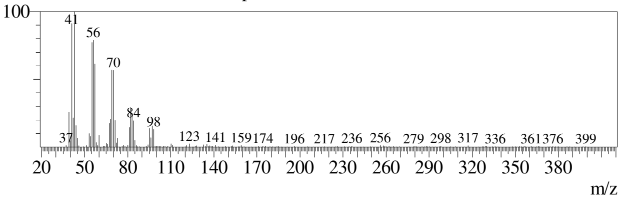

Line#:5 R.Time:12.210(Scan#:1443)
MassPeaks:193
RawMode:Averaged 12.205-12.215(1442-1444) BasePeak:58(1126)
BG Mode:Calc. from Peak Group 1 - Event 1

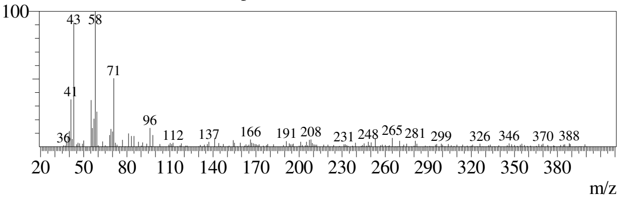

Line#:6 R.Time:12.315(Scan#:1464)
MassPeaks:172
RawMode:Averaged 12.310-12.320(1463-1465) BasePeak:57(1467)
BG Mode:Calc. from Peak Group 1 - Event 1

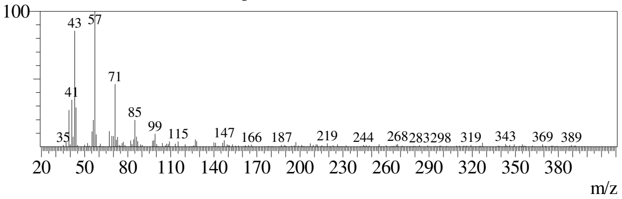

Line#:7 R.Time:12.360(Scan#:1473)
MassPeaks:218
RawMode:Averaged 12.355-12.365(1472-1474) BasePeak:41(17348)
BG Mode:Calc. from Peak Group 1 - Event 1

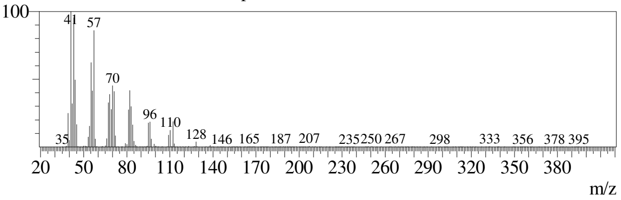

Line#:8 R.Time:13.005(Scan#:1602)
MassPeaks:225
RawMode:Averaged 13.000-13.010(1601-1603) BasePeak:41(3734)
BG Mode:Calc. from Peak Group 1 - Event 1

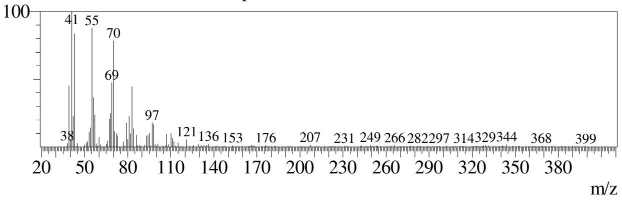

Line#:9 R.Time:13.425(Scan#:1686)
MassPeaks:233
RawMode:Averaged 13.420-13.430(1685-1687) BasePeak:57(3420)
BG Mode:Calc. from Peak Group 1 - Event 1

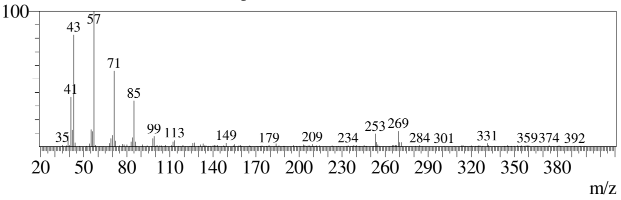

Line#:10 R.Time:13.490(Scan#:1699)
MassPeaks:221
RawMode:Averaged 13.485-13.495(1698-1700) BasePeak:43(5062)
BG Mode:Calc. from Peak Group 1 - Event 1

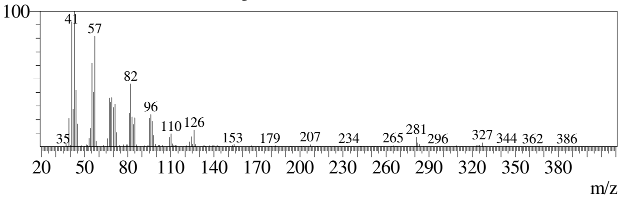

Line#:11 R.Time:13.610(Scan#:1723)
MassPeaks:190
RawMode:Averaged 13.605-13.615(1722-1724) BasePeak:81(3889)
BG Mode:Calc. from Peak Group 1 - Event 1

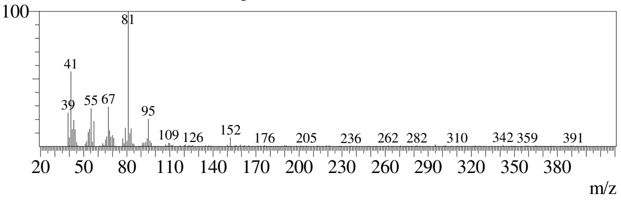

Line#:12 R.Time:14.085(Scan#:1818)
MassPeaks:201
RawMode:Averaged 14.080-14.090(1817-1819) BasePeak:41(5047)
BG Mode:Calc. from Peak Group 1 - Event 1

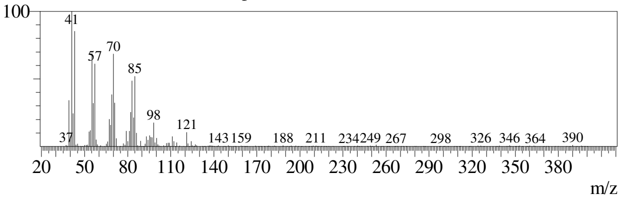

Line#:13 R.Time:14.150(Scan#:1831)
MassPeaks:198
RawMode:Averaged 14.145-14.155(1830-1832) BasePeak:55(4941)
BG Mode:Calc. from Peak Group 1 - Event 1

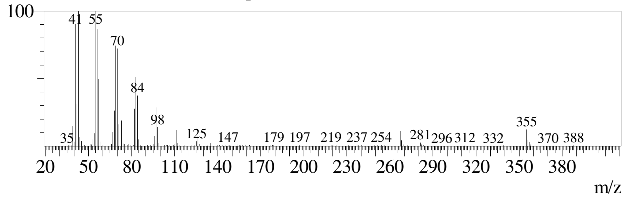

Line#:14 R.Time:14.440(Scan#:1889)
MassPeaks:167
RawMode:Averaged 14.435-14.445(1888-1890) BasePeak:57(9380)
BG Mode:Calc. from Peak Group 1 - Event 1

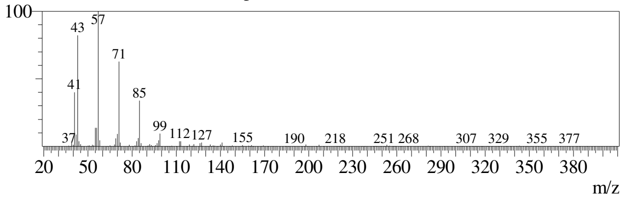

Line#:15 R.Time:14.520(Scan#:1905)
MassPeaks:187
RawMode:Averaged 14.515-14.525(1904-1906) BasePeak:43(4426)
BG Mode:Calc. from Peak Group 1 - Event 1

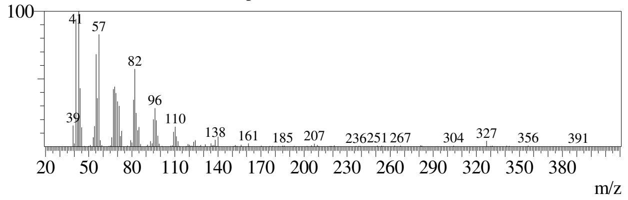

Line#:16 R.Time:14.950(Scan#:1991)
MassPeaks:178
RawMode:Averaged 14.945-14.955(1990-1992) BasePeak:43(3164)
BG Mode:Calc. from Peak Group 1 - Event 1

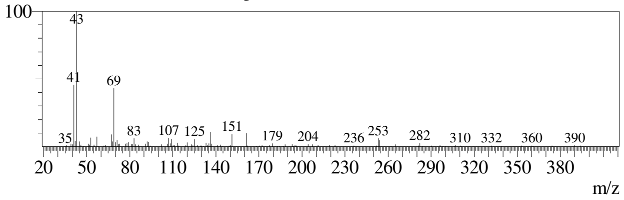

Line#:17 R.Time:15.135(Scan#:2028)
MassPeaks:187
RawMode:Averaged 15.130-15.140(2027-2029) BasePeak:43(2732)
BG Mode:Calc. from Peak Group 1 - Event 1

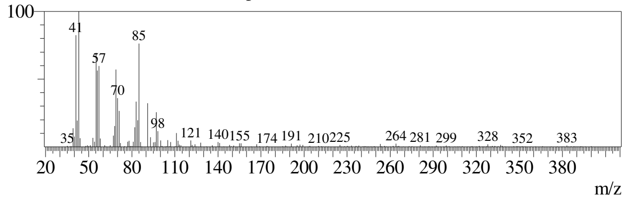

Line#:18 R.Time:15.315(Scan#:2064)
MassPeaks:204
RawMode:Averaged 15.310-15.320(2063-2065) BasePeak:41(1377)
BG Mode:Calc. from Peak Group 1 - Event 1

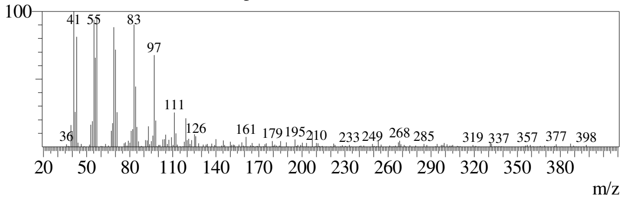

Line#:19 R.Time:15.385(Scan#:2078)
MassPeaks:187
RawMode:Averaged 15.380-15.390(2077-2079) BasePeak:57(22629)
BG Mode:Calc. from Peak Group 1 - Event 1

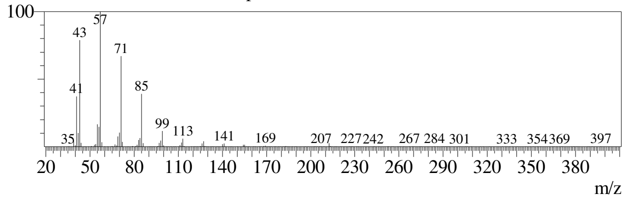

Line#:20 R.Time:15.485(Scan#:2098)
MassPeaks:238
RawMode:Averaged 15.480-15.490(2097-2099) BasePeak:41(4665)
BG Mode:Calc. from Peak Group 1 - Event 1

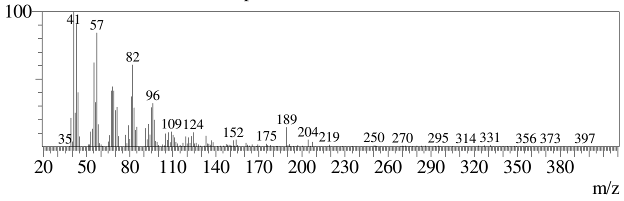

Line#:21 R.Time:16.390(Scan#:2279)
MassPeaks:178
RawMode:Averaged 16.385-16.395(2278-2280) BasePeak:82(3916)
BG Mode:Calc. from Peak Group 1 - Event 1

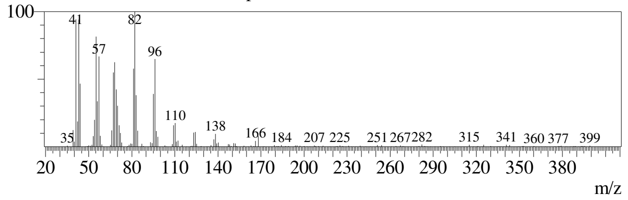

Line#:22 R.Time:16.930(Scan#:2387)
MassPeaks:205
RawMode:Averaged 16.925-16.935(2386-2388) BasePeak:41(4163)
BG Mode:Calc. from Peak Group 1 - Event 1

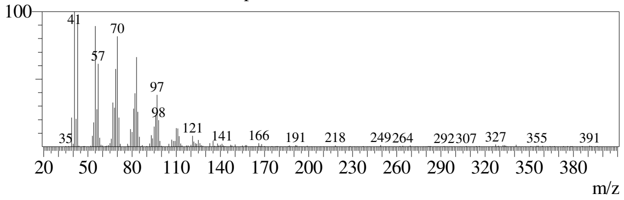

Line#:23 R.Time:16.970(Scan#:2395)
MassPeaks:205
RawMode:Averaged 16.965-16.975(2394-2396) BasePeak:43(1929)
BG Mode:Calc. from Peak Group 1 - Event 1

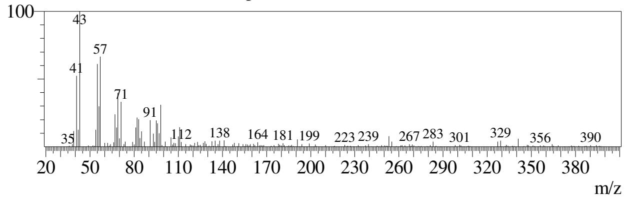

Line#:24 R.Time:17.150(Scan#:2431)
MassPeaks:218
RawMode:Averaged 17.145-17.155(2430-2432) BasePeak:43(10526)
BG Mode:Calc. from Peak Group 1 - Event 1

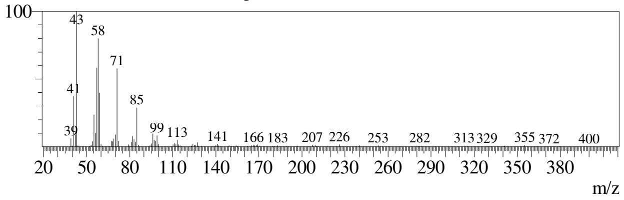

Line#:25 R.Time:17.300(Scan#:2461)
MassPeaks:246
RawMode:Averaged 17.295-17.305(2460-2462) BasePeak:43(19865)
BG Mode:Calc. from Peak Group 1 - Event 1

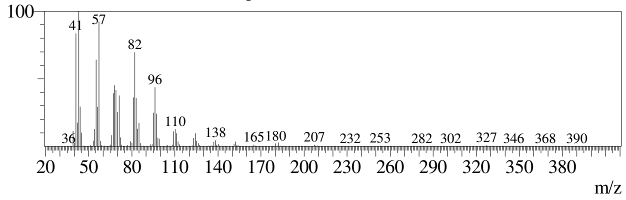

Line#:26 R.Time:18.110(Scan#:2623)
MassPeaks:209
RawMode:Averaged 18.105-18.115(2622-2624) BasePeak:55(3368)
BG Mode:Calc. from Peak Group 1 - Event 1

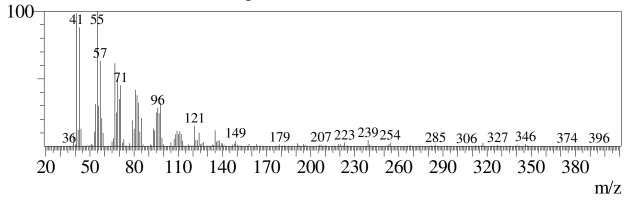

Line#:27 R.Time:18.290(Scan#:2659)
MassPeaks:254
RawMode:Averaged 18.285-18.295(2658-2660) BasePeak:43(273925)
BG Mode:Calc. from Peak Group 1 - Event 1

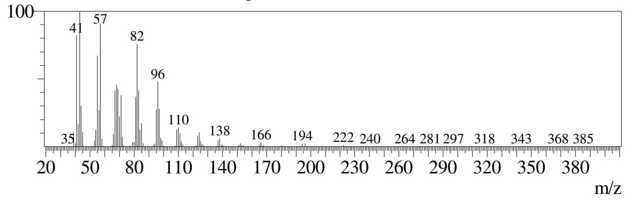

Line#:28 R.Time:18.575(Scan#:2716)
MassPeaks:194
RawMode:Averaged 18.570-18.580(2715-2717) BasePeak:69(1777)
BG Mode:Calc. from Peak Group 1 - Event 1

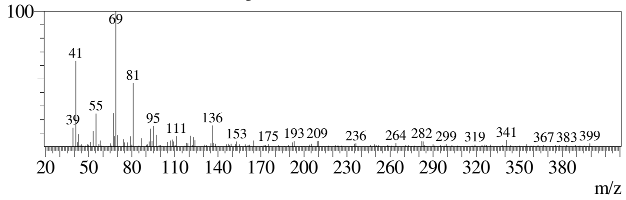

Line#:29 R.Time:18.945(Scan#:2790)
MassPeaks:226
RawMode:Averaged 18.940-18.950(2789-2791) BasePeak:43(18015)
BG Mode:Calc. from Peak Group 1 - Event 1

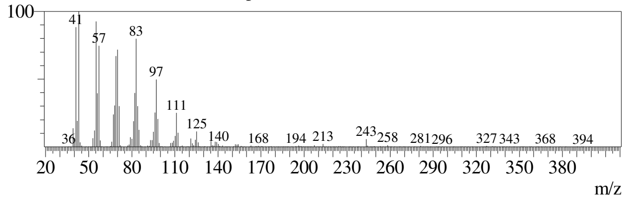

Line#:30 R.Time:20.360(Scan#:3073)
MassPeaks:234
RawMode:Averaged 20.355-20.365(3072-3074) BasePeak:55(8044)
BG Mode:Calc. from Peak Group 1 - Event 1

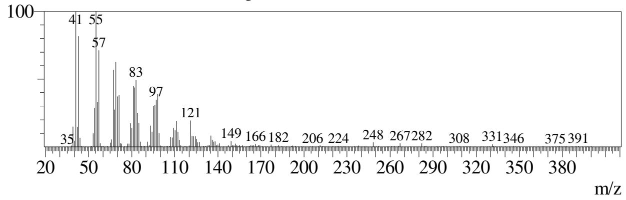

Line#:31 R.Time:20.630(Scan#:3127)
MassPeaks:264
RawMode:Averaged 20.625-20.635(3126-3128) BasePeak:43(10336)
BG Mode:Calc. from Peak Group 1 - Event 1

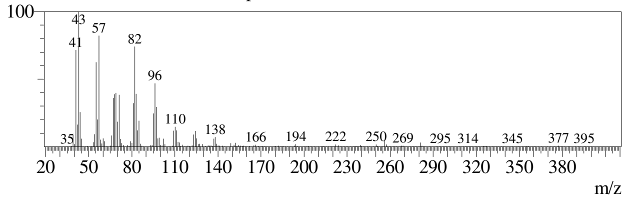

Line#:32 R.Time:21.440(Scan#:3289)
MassPeaks:221
RawMode:Averaged 21.435-21.445(3288-3290) BasePeak:43(12278)
BG Mode:Calc. from Peak Group 1 - Event 1

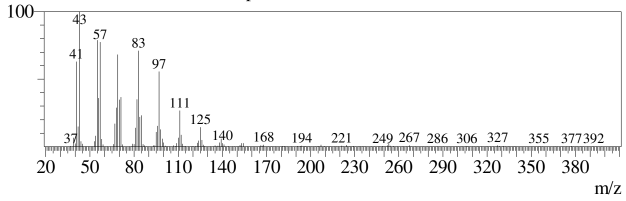

Line#:33 R.Time:21.670(Scan#:3335)
MassPeaks:217
RawMode:Averaged 21.665-21.675(3334-3336) BasePeak:57(5713)
BG Mode:Calc. from Peak Group 1 - Event 1

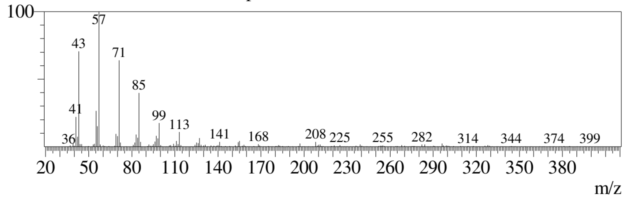

Line#:34 R.Time:23.110(Scan#:3623)
MassPeaks:207
RawMode:Averaged 23.105-23.115(3622-3624) BasePeak:57(2550)
BG Mode:Calc. from Peak Group 1 - Event 1

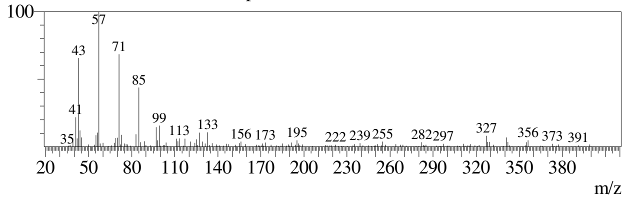

Line#:35 R.Time:24.700(Scan#:3941)
MassPeaks:223
RawMode:Averaged 24.695-24.705(3940-3942) BasePeak:57(89094)
BG Mode:Calc. from Peak Group 1 - Event 1

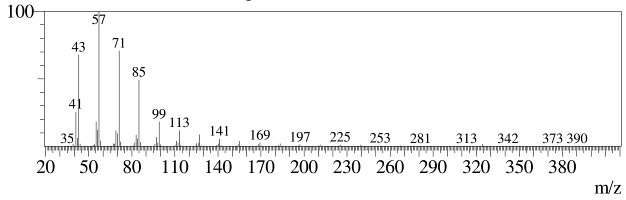

Line#:36 R.Time:29.010(Scan#:4803)
MassPeaks:247
RawMode:Averaged 29.005-29.015(4802-4804) BasePeak:57(90757)
BG Mode:Calc. from Peak Group 1 - Event 1

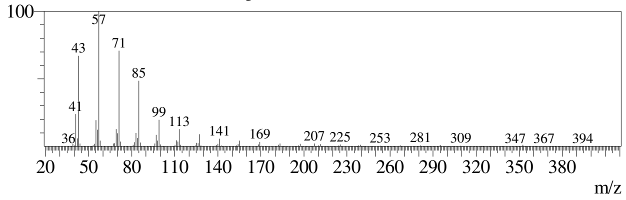

Line#:37 R.Time:32.050(Scan#:5411)
MassPeaks:236
RawMode:Averaged 32.045-32.055(5410-5412) BasePeak:57(12628)
BG Mode:Calc. from Peak Group 1 - Event 1

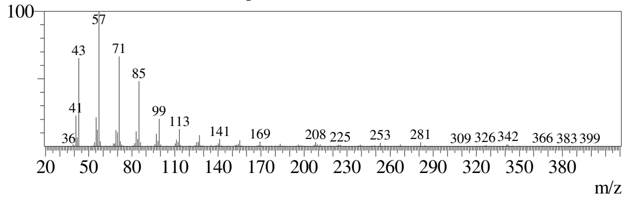

Line#:38 R.Time:35.960(Scan#:6193)
MassPeaks:255
RawMode:Averaged 35.955-35.965(6192-6194) BasePeak:57(292364)
BG Mode:Calc. from Peak Group 1 - Event 1

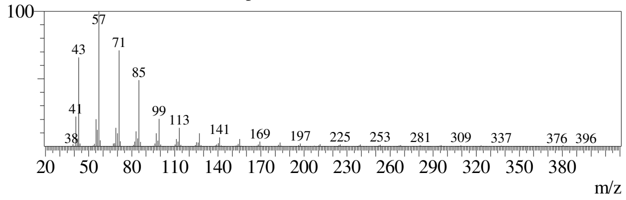

Line#:39 R.Time:41.010(Scan#:7203)
MassPeaks:232
RawMode:Averaged 41.005-41.015(7202-7204) BasePeak:57(12337)
BG Mode:Calc. from Peak Group 1 - Event 1

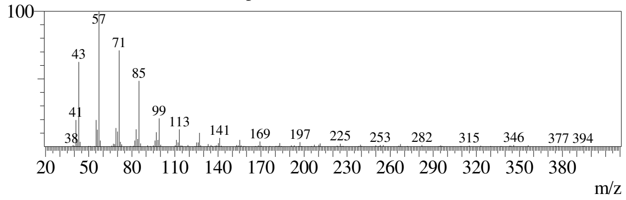

Line#:40 R.Time:42.610(Scan#:7523)
MassPeaks:212
RawMode:Averaged 42.605-42.615(7522-7524) BasePeak:69(17010)
BG Mode:Calc. from Peak Group 1 - Event 1

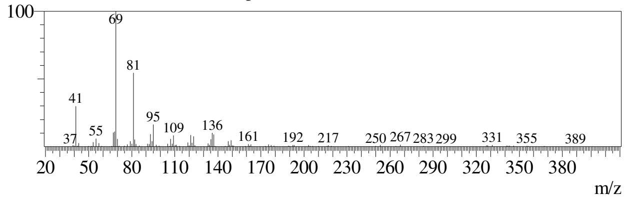

Female thorax extract

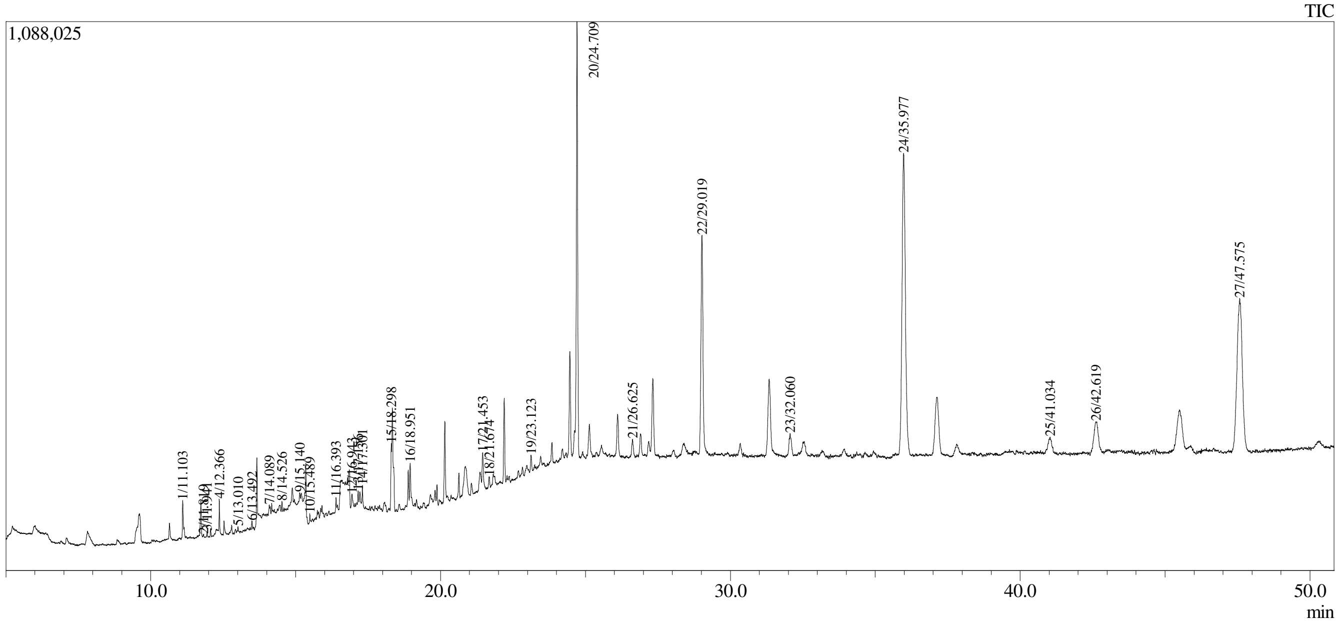

Peak Report TIC

Peak#	R.Time	I.Time	F.Time	Area	Area%	Height	Height%	A/H	Mark	Name
1	11.103	11.055	11.135	150291	0.90	75391	2.43	1.99	MI	Nonanal
2	11.819	11.795	11.855	9704	0.06	5762	0.19	1.68	MI	2-Nonenal
3	11.941	11.900	11.975	16718	0.10	10364	0.33	1.61	MI	Nonanol
4	12.366	12.335	12.410	109985	0.66	67804	2.18	1.62	MI	Decanal
5	13.010	12.985	13.040	12428	0.07	9361	0.30	1.33	MI	2-Decenal
6	13.492	13.450	13.530	30396	0.18	16967	0.55	1.79	MI	Undecanal
7	14.089	14.060	14.120	44862	0.27	18201	0.59	2.46	MI	Undecenal
8	14.526	14.495	14.560	27034	0.16	20065	0.65	1.35	MI	Dodecanal
9	15.140	15.085	15.170	44430	0.27	19706	0.63	2.25	MI	Dodecanol
10	15.489	15.455	15.540	36255	0.22	19278	0.62	1.88	MI	Tridecanal
11	16.393	16.345	16.425	60906	0.36	30376	0.98	2.01	MI	Tetradecanal
12	16.943	16.910	17.005	61705	0.37	23643	0.76	2.61	MI	Tetradecanol
13	17.156	17.130	17.185	45585	0.27	27099	0.87	1.68	MI	Heptadecane
14	17.301	17.260	17.365	96886	0.58	44490	1.43	2.18	MI	Pentadecanal
15	18.298	18.265	18.315	265267	1.58	129020	4.15	2.06	MI	Hexadecanal
16	18.951	18.910	19.045	227184	1.36	83010	2.67	2.74	MI	Hexadecanol
17	21.453	21.415	21.575	223302	1.33	71267	2.29	3.13	MI	Octadecanol
18	21.674	21.605	21.730	74609	0.45	24258	0.78	3.08	MI	Heneicosane
19	23.123	23.080	23.175	88814	0.53	32693	1.05	2.72	MI	Docosane
20	24.709	24.530	24.795	2871215	17.14	862281	27.77	3.33	MI	Tricosane
21	26.625	26.510	26.745	143960	0.86	35356	1.14	4.07	MI	Tetracosane
22	29.019	28.875	29.290	2134486	12.74	434669	14.00	4.91	MI	Pentacosane
23	32.060	31.895	32.190	292826	1.75	47067	1.52	6.22	MI	Hexacosane
24	35.977	35.770	36.330	4808443	28.70	597713	19.25	8.04	MI	Heptacosane
25	41.034	40.855	41.240	327486	1.95	33877	1.09	9.67	MI	Octacosane
26	42.619	42.425	42.865	695683	4.15	63220	2.04	11.00	MI	Squalene
27	47.575	47.275	47.895	3850908	22.99	302686	9.75	12.72	MI	Nonacosane
				16751368	100.00	3105624	100.00			

Spectrum

Line#:1 R.Time:11.105(Scan#:1222)
MassPeaks:186
RawMode:Averaged 11.100-11.110(1221-1223) BasePeak:57(6720)
BG Mode:Calc. from Peak Group 1 - Event 1

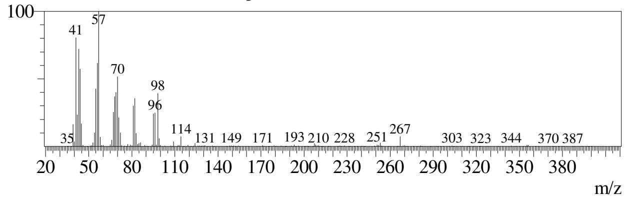

Line#:2 R.Time:11.820(Scan#:1365)
MassPeaks:209
RawMode:Averaged 11.815-11.825(1364-1366) BasePeak:43(481)
BG Mode:Calc. from Peak Group 1 - Event 1

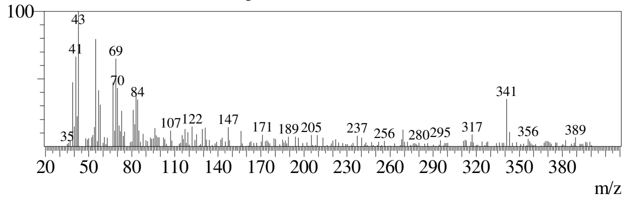

Line#:3 R.Time:11.940(Scan#:1389)
MassPeaks:187
RawMode:Averaged 11.935-11.945(1388-1390) BasePeak:43(935)
BG Mode:Calc. from Peak Group 1 - Event 1

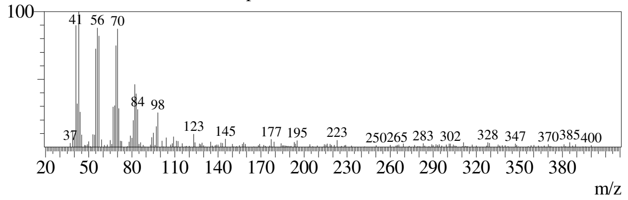

Line#:4 R.Time:12.365(Scan#:1474)
MassPeaks:192
RawMode:Averaged 12.360-12.370(1473-1475) BasePeak:57(5106)
BG Mode:Calc. from Peak Group 1 - Event 1

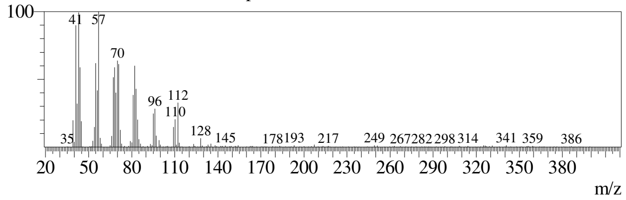

Line#:5 R.Time:13.010(Scan#:1603)
MassPeaks:214
RawMode:Averaged 13.005-13.015(1602-1604) BasePeak:41(792)
BG Mode:Calc. from Peak Group 1 - Event 1

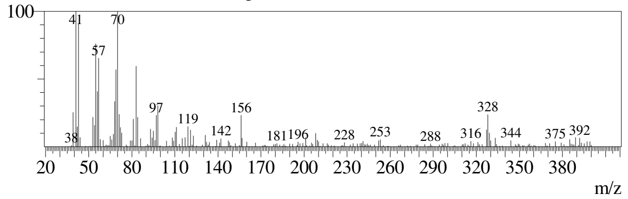

Line#:6 R.Time:13.490(Scan#:1699)
MassPeaks:200
RawMode:Averaged 13.485-13.495(1698-1700) BasePeak:43(1236)
BG Mode:Calc. from Peak Group 1 - Event 1

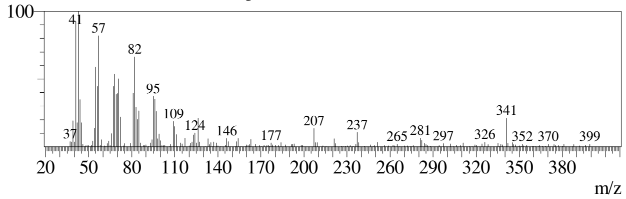

Line#:7 R.Time:14.090(Scan#:1819)
MassPeaks:200
RawMode:Averaged 14.085-14.095(1818-1820) BasePeak:70(1130)
BG Mode:Calc. from Peak Group 1 - Event 1

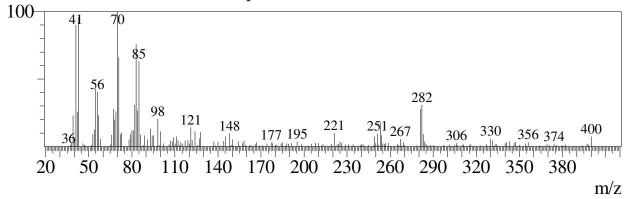

Line#:8 R.Time:14.525(Scan#:1906)
MassPeaks:219
RawMode:Averaged 14.520-14.530(1905-1907) BasePeak:57(1279)
BG Mode:Calc. from Peak Group 1 - Event 1

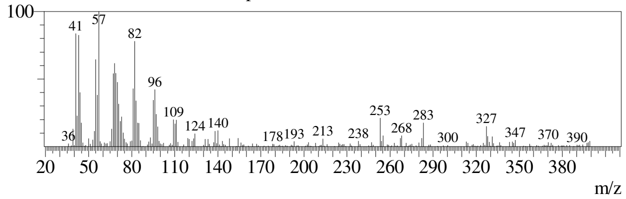

Line#:9 R.Time:15.140(Scan#:2029)
MassPeaks:188
RawMode:Averaged 15.135-15.145(2028-2030) BasePeak:43(1634)
BG Mode:Calc. from Peak Group 1 - Event 1

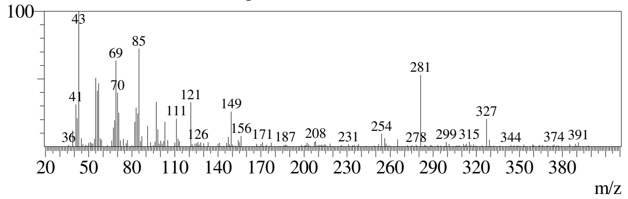

Line#:10 R.Time:15.490(Scan#:2099)
MassPeaks:197
RawMode:Averaged 15.485-15.495(2098-2100) BasePeak:57(1343)
BG Mode:Calc. from Peak Group 1 - Event 1

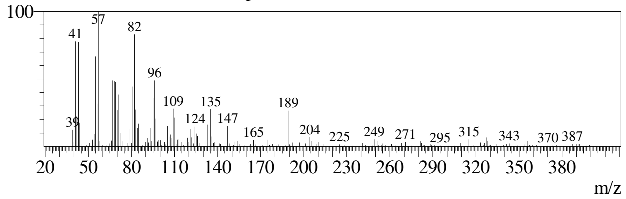

Line#:11 R.Time:16.395(Scan#:2280)
MassPeaks:218
RawMode:Averaged 16.390-16.400(2279-2281) BasePeak:57(1486)
BG Mode:Calc. from Peak Group 1 - Event 1

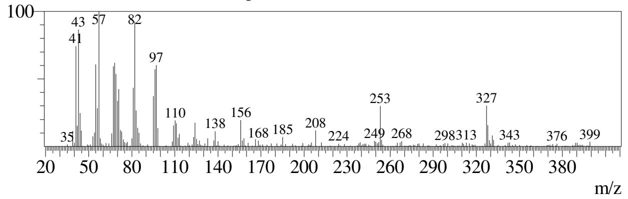

Line#:12 R.Time:16.945(Scan#:2390)
MassPeaks:189
RawMode:Averaged 16.940-16.950(2389-2391) BasePeak:69(1968)
BG Mode:Calc. from Peak Group 1 - Event 1

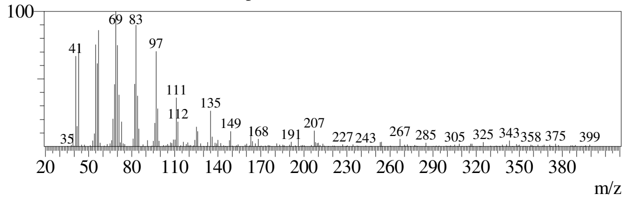

Line#:13 R.Time:17.155(Scan#:2432)
MassPeaks:202
RawMode:Averaged 17.150-17.160(2431-2433) BasePeak:71(3585)
BG Mode:Calc. from Peak Group 1 - Event 1

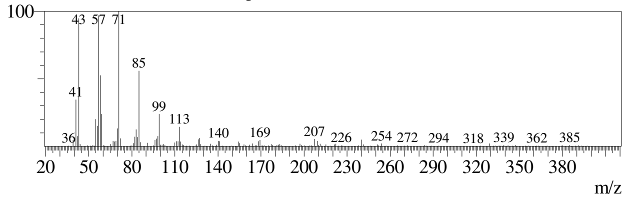

Line#:14 R.Time:17.300(Scan#:2461)
MassPeaks:214
RawMode:Averaged 17.295-17.305(2460-2462) BasePeak:57(2597)
BG Mode:Calc. from Peak Group 1 - Event 1

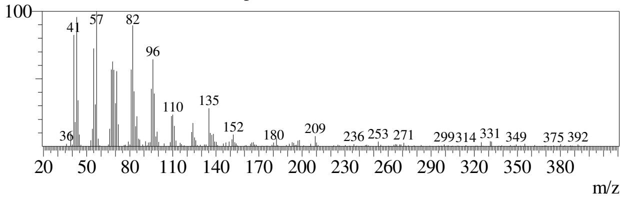

Line#:15 R.Time:18.300(Scan#:2661)
MassPeaks:153
RawMode:Averaged 18.295-18.305(2660-2662) BasePeak:82(5441)
BG Mode:Calc. from Peak Group 1 - Event 1

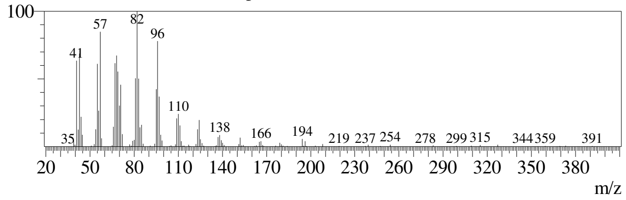

Line#:16 R.Time:18.950(Scan#:2791)
MassPeaks:212
RawMode:Averaged 18.945-18.955(2790-2792) BasePeak:69(6318)
BG Mode:Calc. from Peak Group 1 - Event 1

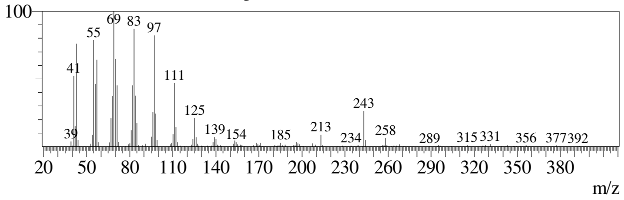

Line#:17 R.Time:21.455(Scan#:3292)
MassPeaks:222
RawMode:Averaged 21.450-21.460(3291-3293) BasePeak:69(4845)
BG Mode:Calc. from Peak Group 1 - Event 1

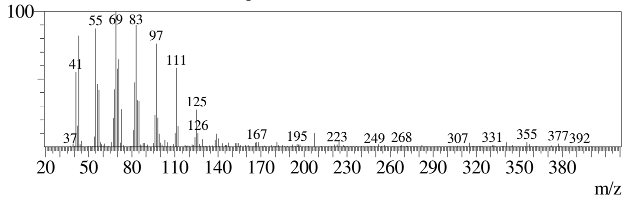

Line#:18 R.Time:21.675(Scan#:3336)
MassPeaks:215
RawMode:Averaged 21.670-21.680(3335-3337) BasePeak:57(3401)
BG Mode:Calc. from Peak Group 1 - Event 1

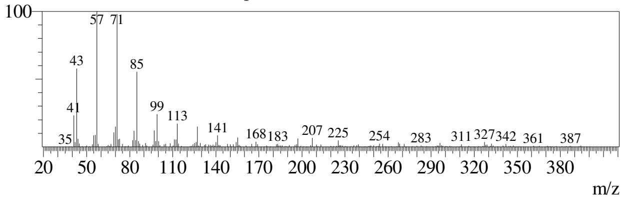

Line#:19 R.Time:23.125(Scan#:3626)
MassPeaks:221
RawMode:Averaged 23.120-23.130(3625-3627) BasePeak:57(5028)
BG Mode:Calc. from Peak Group 1 - Event 1

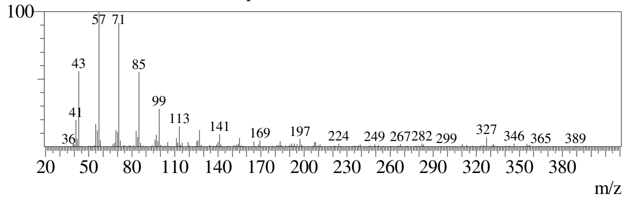

Line#:20 R.Time:24.710(Scan#:3943)
MassPeaks:246
RawMode:Averaged 24.705-24.715(3942-3944) BasePeak:57(137061)
BG Mode:Calc. from Peak Group 1 - Event 1

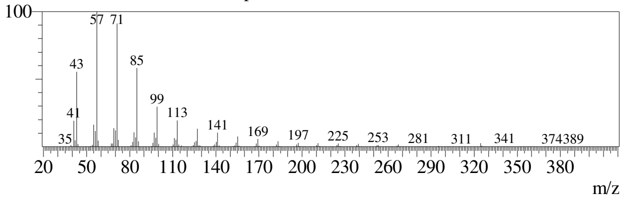

Line#:21 R.Time:26.625(Scan#:4326)
MassPeaks:236
RawMode:Averaged 26.620-26.630(4325-4327) BasePeak:57(4938)
BG Mode:Calc. from Peak Group 1 - Event 1

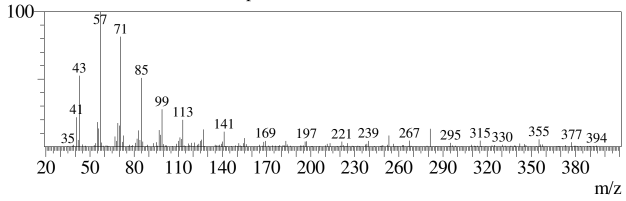

Line#:22 R.Time:29.020(Scan#:4805)
MassPeaks:227
RawMode:Averaged 29.015-29.025(4804-4806) BasePeak:57(67103)
BG Mode:Calc. from Peak Group 1 - Event 1

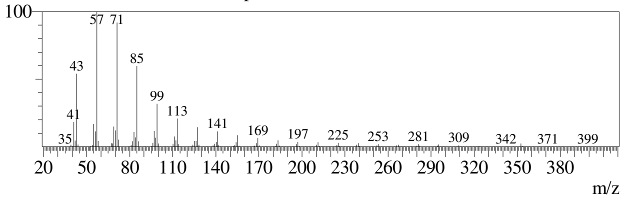

Line#:23 R.Time:32.060(Scan#:5413)
MassPeaks:217
RawMode:Averaged 32.055-32.065(5412-5414) BasePeak:57(6225)
BG Mode:Calc. from Peak Group 1 - Event 1

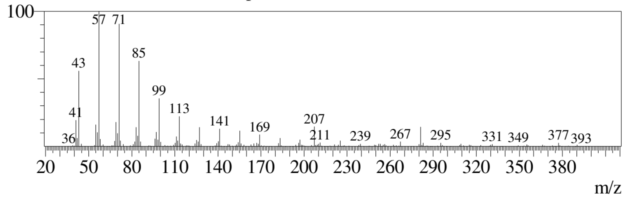

Line#:24 R.Time:35.975(Scan#:6196)
MassPeaks:233
RawMode:Averaged 35.970-35.980(6195-6197) BasePeak:57(89474)
BG Mode:Calc. from Peak Group 1 - Event 1

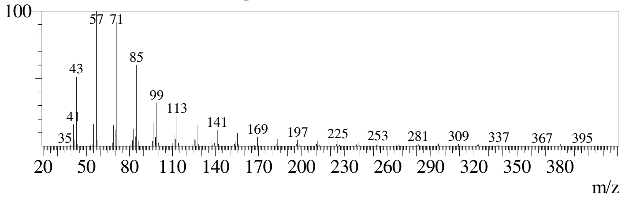

Line#:25 R.Time:41.035(Scan#:7208)
MassPeaks:225
RawMode:Averaged 41.030-41.040(7207-7209) BasePeak:57(4283)
BG Mode:Calc. from Peak Group 1 - Event 1

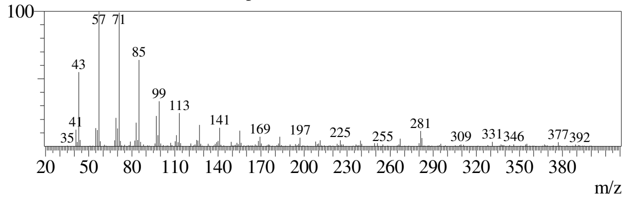

Line#:26 R.Time:42.620(Scan#:7525)
MassPeaks:243
RawMode:Averaged 42.615-42.625(7524-7526) BasePeak:69(14070)
BG Mode:Calc. from Peak Group 1 - Event 1

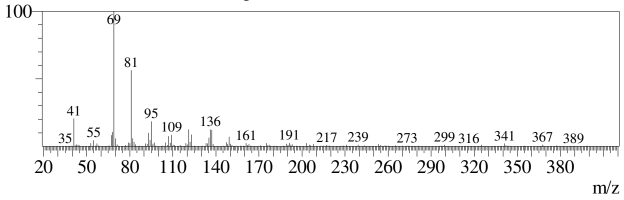

Line#:27 R.Time:47.575(Scan#:8516)
MassPeaks:237
RawMode:Averaged 47.570-47.580(8515-8517) BasePeak:57(43258)
BG Mode:Calc. from Peak Group 1 - Event 1

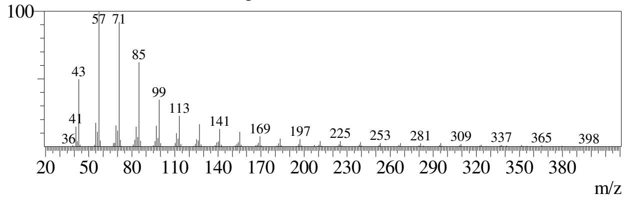

Female hindwings extract

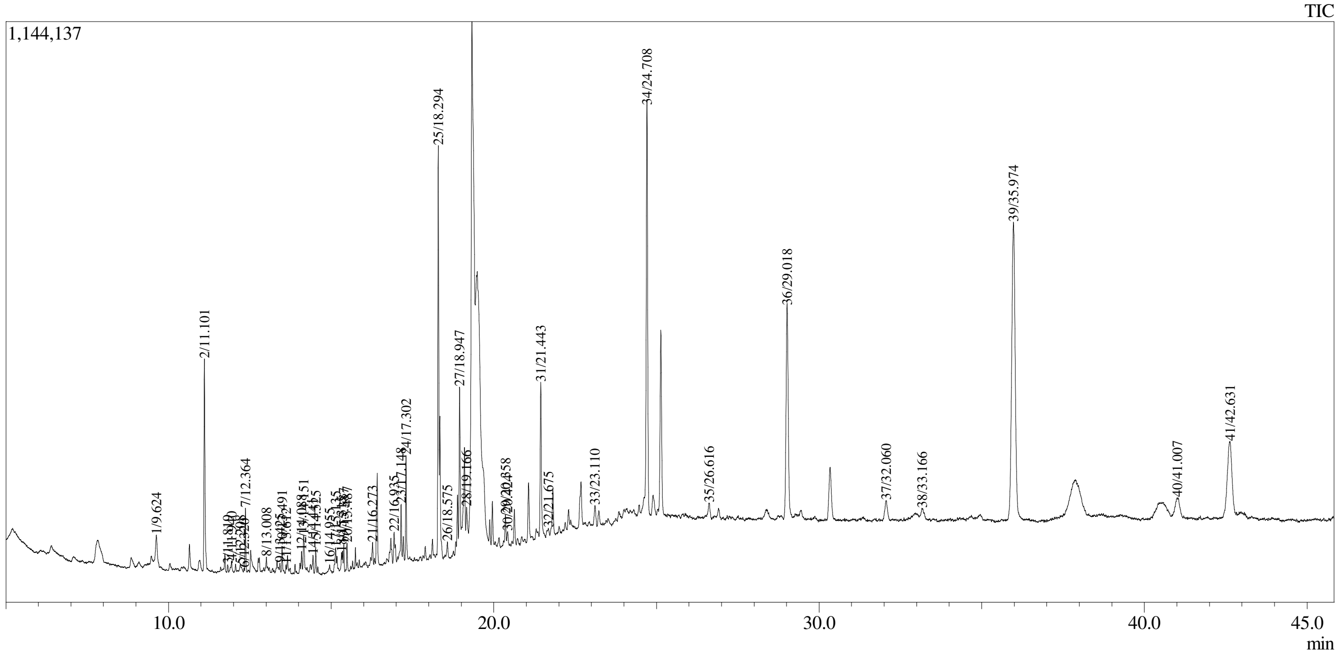

Peak Report TIC

Peak#	R.Time	I.Time	F.Time	Area	Area%	Height	Height%	A/H	Mark	Name
1	9.624	9.550	9.725	273220	1.08	63378	1.05	4.31	MI	Octanal
2	11.101	11.050	11.290	967742	3.82	418863	6.94	2.31	SV	Nonanal
3	11.819	11.790	11.850	32049	0.13	16844	0.28	1.90	V	2-Nonenal
4	11.940	11.850	12.005	82829	0.33	24299	0.40	3.41	V	Nonanol
5	12.208	12.165	12.240	36309	0.14	13519	0.22	2.69	MI	2-Decanona
6	12.320	12.295	12.335	9986	0.04	7866	0.13	1.27	MI	Dodecane
7	12.364	12.335	12.400	191692	0.76	125498	2.08	1.53	MI	Decanal
8	13.008	12.950	13.030	76351	0.30	32299	0.54	2.36	V	2-Decenal
9	13.425	13.400	13.455	35219	0.14	20436	0.34	1.72	V	Tridecane
10	13.491	13.455	13.525	91511	0.36	52218	0.87	1.75	V	Undecanal
11	13.612	13.575	13.630	27341	0.11	17030	0.28	1.61		Decadienal
12	14.088	14.065	14.115	80005	0.32	45185	0.75	1.77	V	Undecenal
13	14.151	14.115	14.235	229963	0.91	76518	1.27	3.01	V	Undecanol
14	14.441	14.390	14.480	91512	0.36	37919	0.63	2.41	V	Tetradecane
15	14.525	14.480	14.570	107678	0.42	54096	0.90	1.99	V	Dodecanal
16	14.955	14.840	15.025	59095	0.23	16721	0.28	3.53		Geranyl acetone
17	15.135	15.070	15.165	137752	0.54	48316	0.80	2.85		Dodecanol
18	15.315	15.250	15.330	75100	0.30	38605	0.64	1.95	V	Pentadecene
19	15.387	15.365	15.420	83416	0.33	54168	0.90	1.54	V	Pentadecane
20	15.487	15.420	15.550	106861	0.42	56793	0.94	1.88		Tridecanal
21	16.273	16.230	16.305	145423	0.57	55472	0.92	2.62	V	Hexadecane
22	16.935	16.890	17.015	286667	1.13	73287	1.21	3.91	V	Tetradecanol
23	17.148	17.065	17.185	365853	1.44	127649	2.11	2.87	V	2-pentadecanona
24	17.302	17.260	17.380	498503	1.97	222882	3.69	2.24	V	Pentadecanal
25	18.294	18.200	18.320	1623487	6.40	830664	13.76	1.95	V	Hexadecanal
26	18.575	18.530	18.640	187064	0.74	48672	0.81	3.84	V	Trimethyl tridecatrienal
27	18.947	18.910	18.995	919009	3.62	352350	5.84	2.61	V	Hexadecanol
28	19.166	19.135	19.220	446197	1.76	114727	1.90	3.89	V	Heptadecenal
29	20.358	20.320	20.395	300221	1.18	95523	1.58	3.14	V	Eicosane
30	20.424	20.395	20.470	220702	0.87	62616	1.04	3.52	V	Hexadecanyl acetate
31	21.443	21.385	21.595	1445464	5.70	353564	5.86	4.09	V	Octadecanol
32	21.675	21.640	21.720	282604	1.11	64650	1.07	4.37	V	Heneicosane
33	23.110	23.020	23.165	699469	2.76	104889	1.74	6.67	V	Docosane
34	24.708	24.515	24.825	4159601	16.40	881004	14.60	4.72	V	Tricosane
35	26.616	26.545	26.695	149986	0.59	32929	0.55	4.55	MI	Tetracosane
36	29.018	28.905	29.185	2993847	11.80	477925	7.92	6.26	V	Pentacosane
37	32.060	31.915	32.175	930116	3.67	83750	1.39	11.11	V	Hexacosane
38	33.166	33.085	33.325	767253	3.02	64871	1.07	11.83	V	Tetracosanal
39	35.974	35.800	36.150	4433579	17.48	587088	9.73	7.55	MI	Heptacosane
40	41.007	40.870	41.180	386916	1.53	39797	0.66	9.72	MI	Octacosane
41	42.631	42.450	42.785	1329054	5.24	140583	2.33	9.45	MI	Squalene
				25366646	100.00	6035463	100.00			

Spectrum

Line#:1 R.Time:9.625(Scan#:926)
MassPeaks:211
RawMode:Averaged 9.620-9.630(925-927) BasePeak:281(6831)
BG Mode:Calc. from Peak Group 1 - Event 1

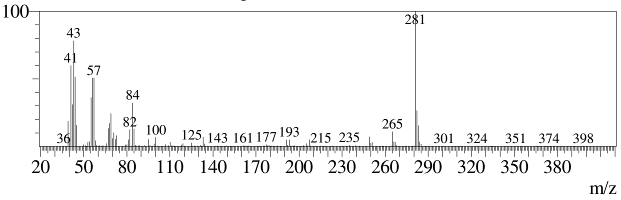

Line#:2 R.Time:11.100(Scan#:1221)
MassPeaks:258
RawMode:Averaged 11.095-11.105(1220-1222) BasePeak:57(46266)
BG Mode:Calc. from Peak Group 1 - Event 1

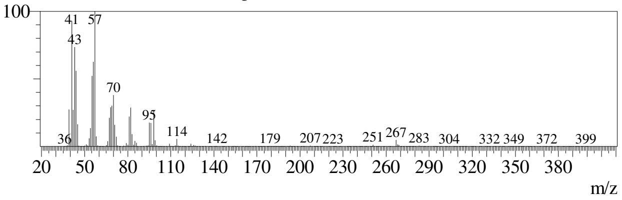

Line#:3 R.Time:11.820(Scan#:1365)
MassPeaks:219
RawMode:Averaged 11.815-11.825(1364-1366) BasePeak:41(1419)
BG Mode:Calc. from Peak Group 1 - Event 1

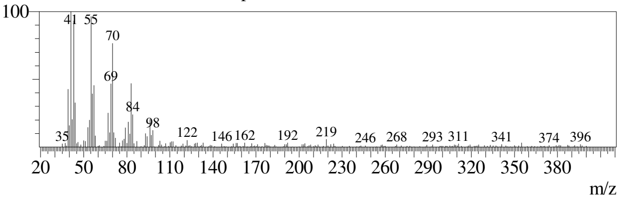

Line#:4 R.Time:11.940(Scan#:1389)
MassPeaks:215
RawMode:Averaged 11.935-11.945(1388-1390) BasePeak:41(2064)
BG Mode:Calc. from Peak Group 1 - Event 1

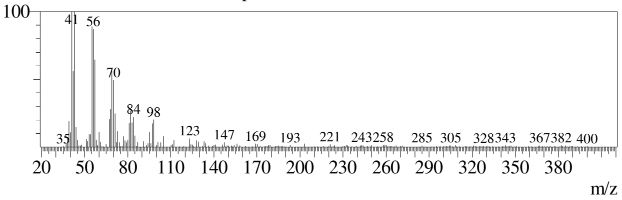

Line#:5 R.Time:12.210(Scan#:1443)
MassPeaks:174
RawMode:Averaged 12.205-12.215(1442-1444) BasePeak:43(2629)
BG Mode:Calc. from Peak Group 1 - Event 1

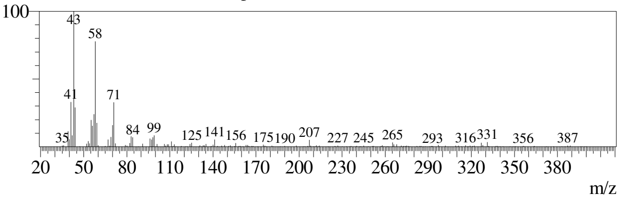

Line#:6 R.Time:12.320(Scan#:1465)
MassPeaks:196
RawMode:Averaged 12.315-12.325(1464-1466) BasePeak:57(1121)
BG Mode:Calc. from Peak Group 1 - Event 1

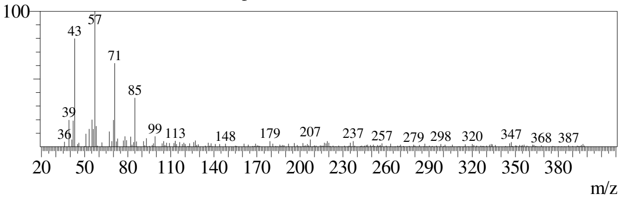

Line#:7 R.Time:12.365(Scan#:1474)
MassPeaks:204
RawMode:Averaged 12.360-12.370(1473-1475) BasePeak:41(11346)
BG Mode:Calc. from Peak Group 1 - Event 1

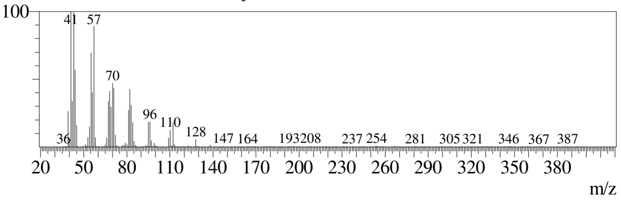

Line#:8 R.Time:13.010(Scan#:1603)
MassPeaks:223
RawMode:Averaged 13.005-13.015(1602-1604) BasePeak:43(2177)
BG Mode:Calc. from Peak Group 1 - Event 1

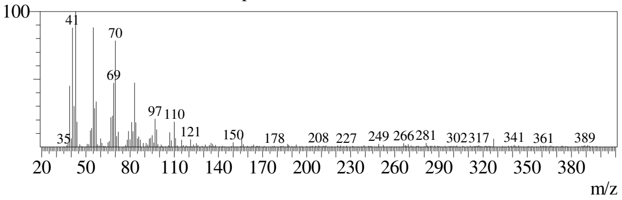

Line#:9 R.Time:13.425(Scan#:1686)
MassPeaks:193
RawMode:Averaged 13.420-13.430(1685-1687) BasePeak:57(2863)
BG Mode:Calc. from Peak Group 1 - Event 1

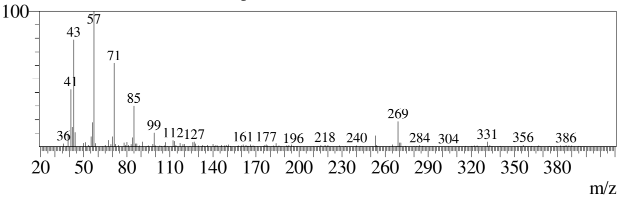

Line#:10 R.Time:13.490(Scan#:1699)
MassPeaks:211
RawMode:Averaged 13.485-13.495(1698-1700) BasePeak:43(4437)
BG Mode:Calc. from Peak Group 1 - Event 1

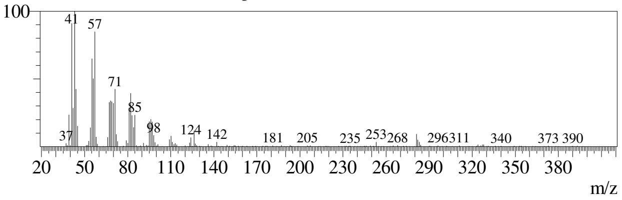

Line#:11 R.Time:13.610(Scan#:1723)
MassPeaks:157
RawMode:Averaged 13.605-13.615(1722-1724) BasePeak:81(3312)
BG Mode:Calc. from Peak Group 1 - Event 1

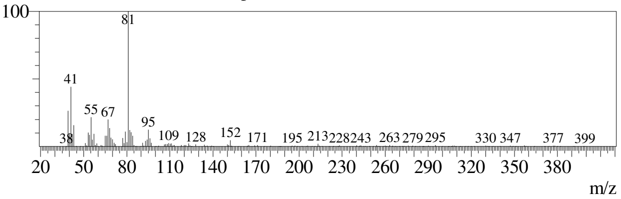

Line#:12 R.Time:14.090(Scan#:1819)
MassPeaks:179
RawMode:Averaged 14.085-14.095(1818-1820) BasePeak:85(3960)
BG Mode:Calc. from Peak Group 1 - Event 1

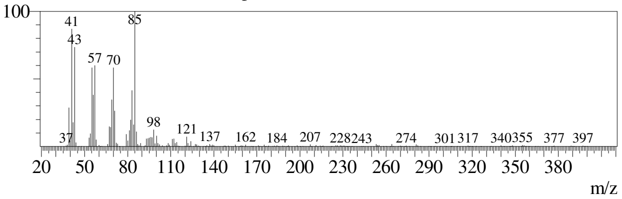

Line#:13 R.Time:14.150(Scan#:1831)
MassPeaks:191
RawMode:Averaged 14.145-14.155(1830-1832) BasePeak:55(6316)
BG Mode:Calc. from Peak Group 1 - Event 1

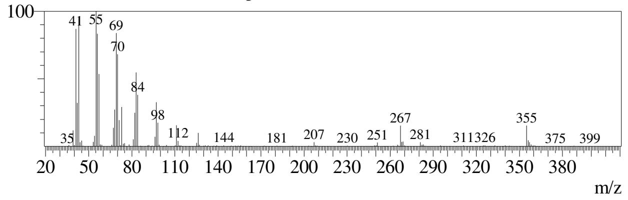

Line#:14 R.Time:14.440(Scan#:1889)
MassPeaks:194
RawMode:Averaged 14.435-14.445(1888-1890) BasePeak:57(6135)
BG Mode:Calc. from Peak Group 1 - Event 1

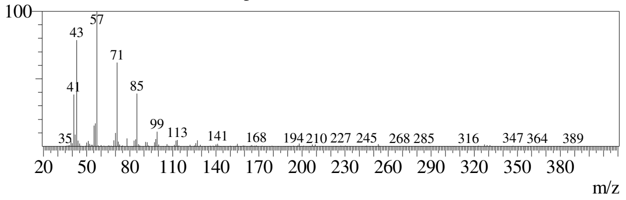

Line#:15 R.Time:14.525(Scan#:1906)
MassPeaks:215
RawMode:Averaged 14.520-14.530(1905-1907) BasePeak:43(4158)
BG Mode:Calc. from Peak Group 1 - Event 1

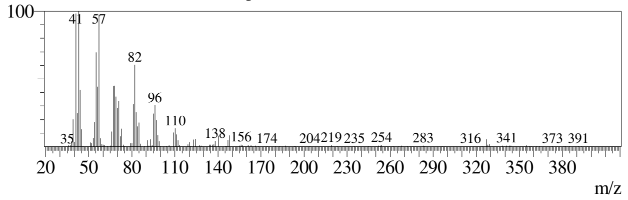

Line#:16 R.Time:14.955(Scan#:1992)
MassPeaks:201
RawMode:Averaged 14.950-14.960(1991-1993) BasePeak:43(4103)
BG Mode:Calc. from Peak Group 1 - Event 1

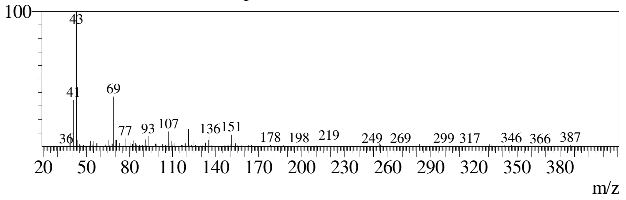

Line#:17 R.Time:15.135(Scan#:2028)
MassPeaks:174
RawMode:Averaged 15.130-15.140(2027-2029) BasePeak:43(3869)
BG Mode:Calc. from Peak Group 1 - Event 1

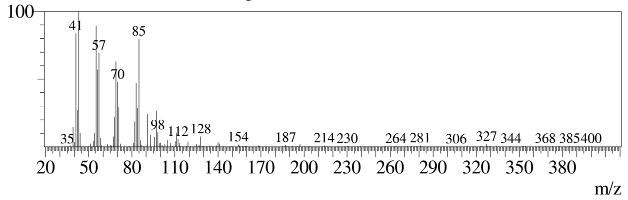

Line#:18 R.Time:15.315(Scan#:2064)
MassPeaks:187
RawMode:Averaged 15.310-15.320(2063-2065) BasePeak:55(1737)
BG Mode:Calc. from Peak Group 1 - Event 1

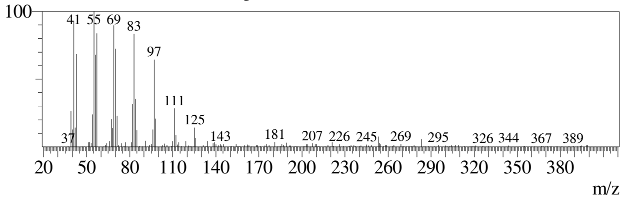

Line#:19 R.Time:15.385(Scan#:2078)
MassPeaks:180
RawMode:Averaged 15.380-15.390(2077-2079) BasePeak:57(9510)
BG Mode:Calc. from Peak Group 1 - Event 1

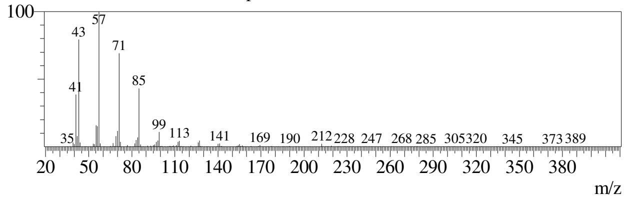

Line#:20 R.Time:15.485(Scan#:2098)
MassPeaks:210
RawMode:Averaged 15.480-15.490(2097-2099) BasePeak:43(4979)
BG Mode:Calc. from Peak Group 1 - Event 1

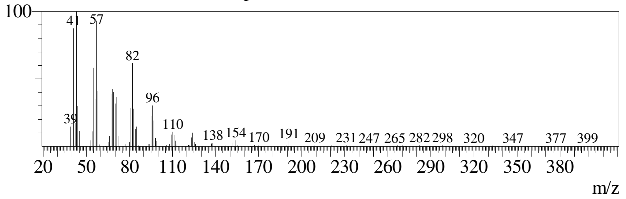

Line#:21 R.Time:16.275(Scan#:2256)
MassPeaks:223
RawMode:Averaged 16.270-16.280(2255-2257) BasePeak:57(5793)
BG Mode:Calc. from Peak Group 1 - Event 1

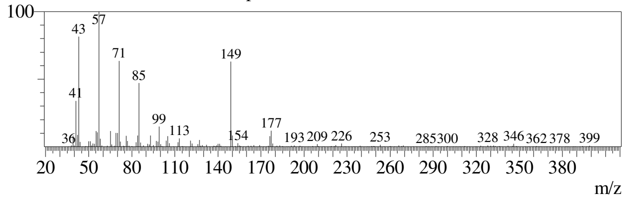

Line#:22 R.Time:16.935(Scan#:2388)
MassPeaks:244
RawMode:Averaged 16.930-16.940(2387-2389) BasePeak:43(5061)
BG Mode:Calc. from Peak Group 1 - Event 1

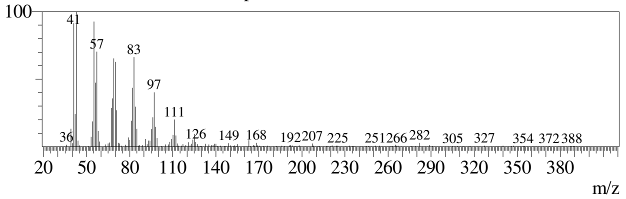

Line#:23 R.Time:17.150(Scan#:2431)
MassPeaks:233
RawMode:Averaged 17.145-17.155(2430-2432) BasePeak:58(17884)
BG Mode:Calc. from Peak Group 1 - Event 1

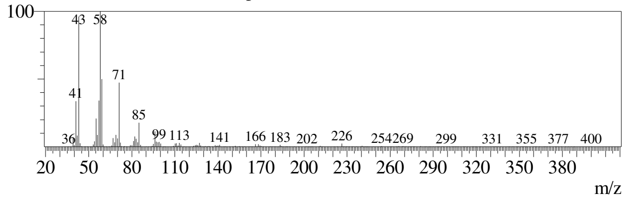

Line#:24 R.Time:17.300(Scan#:2461)
MassPeaks:211
RawMode:Averaged 17.295-17.305(2460-2462) BasePeak:43(18005)
BG Mode:Calc. from Peak Group 1 - Event 1

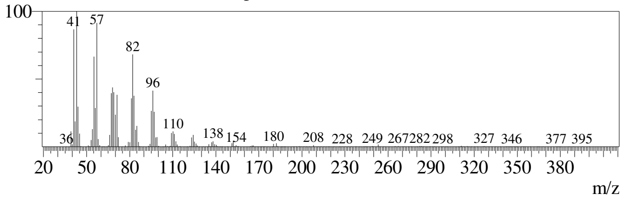

Line#:25 R.Time:18.295(Scan#:2660)
MassPeaks:191
RawMode:Averaged 18.290-18.300(2659-2661) BasePeak:43(59982)
BG Mode:Calc. from Peak Group 1 - Event 1

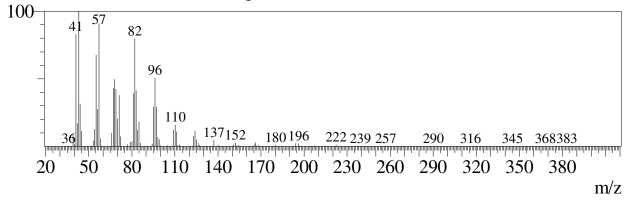

Line#:26 R.Time:18.575(Scan#:2716)
MassPeaks:211
RawMode:Averaged 18.570-18.580(2715-2717) BasePeak:69(6447)
BG Mode:Calc. from Peak Group 1 - Event 1

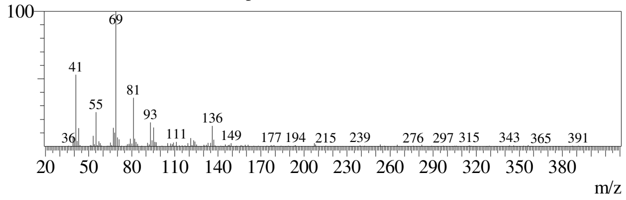

Line#:27 R.Time:18.945(Scan#:2790)
MassPeaks:259
RawMode:Averaged 18.940-18.950(2789-2791) BasePeak:43(25441)
BG Mode:Calc. from Peak Group 1 - Event 1

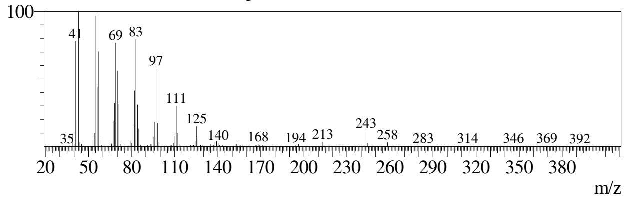

Line#:28 R.Time:19.165(Scan#:2834)
MassPeaks:218
RawMode:Averaged 19.160-19.170(2833-2835) BasePeak:41(3678)
BG Mode:Calc. from Peak Group 1 - Event 1

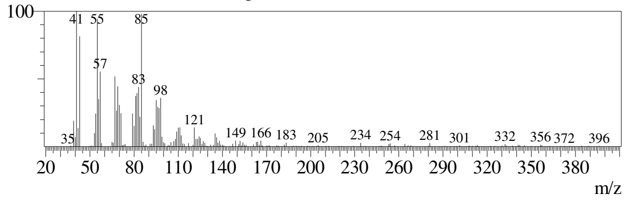

Line#:29 R.Time:20.360(Scan#:3073)
MassPeaks:217
RawMode:Averaged 20.355-20.365(3072-3074) BasePeak:57(8080)
BG Mode:Calc. from Peak Group 1 - Event 1

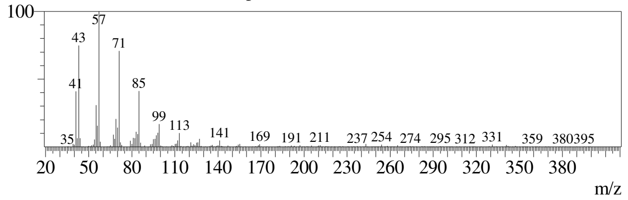

Line#:30 R.Time:20.425(Scan#:3086)
MassPeaks:229
RawMode:Averaged 20.420-20.430(3085-3087) BasePeak:43(2109)
BG Mode:Calc. from Peak Group 1 - Event 1

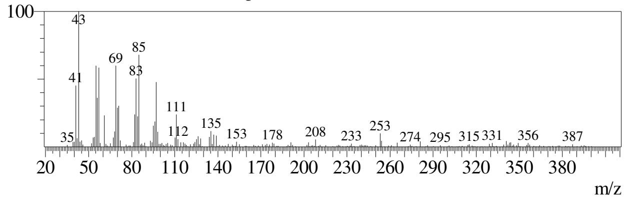

Line#:31 R.Time:21.445(Scan#:3290)
MassPeaks:230
RawMode:Averaged 21.440-21.450(3289-3291) BasePeak:43(25899)
BG Mode:Calc. from Peak Group 1 - Event 1

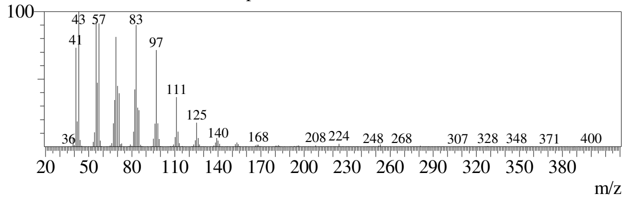

Line#:32 R.Time:21.675(Scan#:3336)
MassPeaks:165
RawMode:Averaged 21.670-21.680(3335-3337) BasePeak:57(1892)
BG Mode:Calc. from Peak Group 1 - Event 1

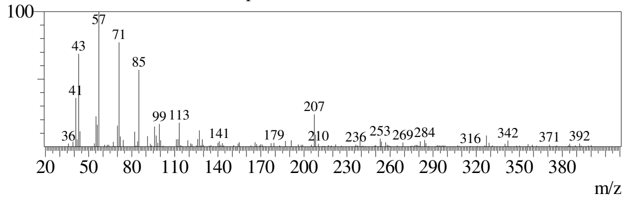

Line#:33 R.Time:23.110(Scan#:3623)
MassPeaks:214
RawMode:Averaged 23.105-23.115(3622-3624) BasePeak:57(6006)
BG Mode:Calc. from Peak Group 1 - Event 1

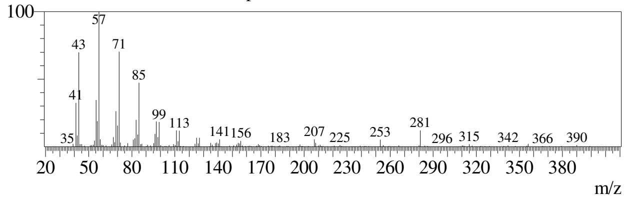

Line#:34 R.Time:24.710(Scan#:3943)
MassPeaks:259
RawMode:Averaged 24.705-24.715(3942-3944) BasePeak:57(147813)
BG Mode:Calc. from Peak Group 1 - Event 1

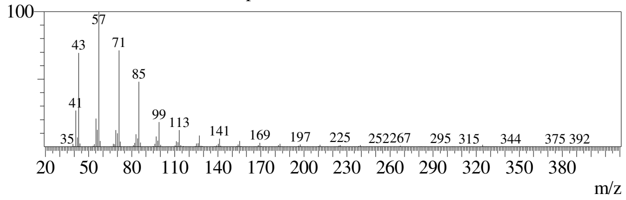

Line#:35 R.Time:26.615(Scan#:4324)
MassPeaks:223
RawMode:Averaged 26.610-26.620(4323-4325) BasePeak:57(4412)
BG Mode:Calc. from Peak Group 1 - Event 1

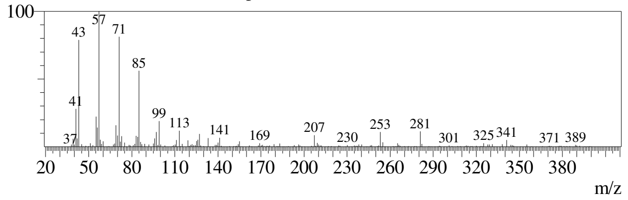

Line#:36 R.Time:29.020(Scan#:4805)
MassPeaks:247
RawMode:Averaged 29.015-29.025(4804-4806) BasePeak:57(75132)
BG Mode:Calc. from Peak Group 1 - Event 1

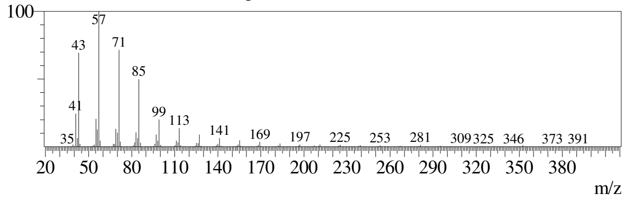

Line#:37 R.Time:32.060(Scan#:5413)
MassPeaks:231
RawMode:Averaged 32.055-32.065(5412-5414) BasePeak:57(6724)
BG Mode:Calc. from Peak Group 1 - Event 1

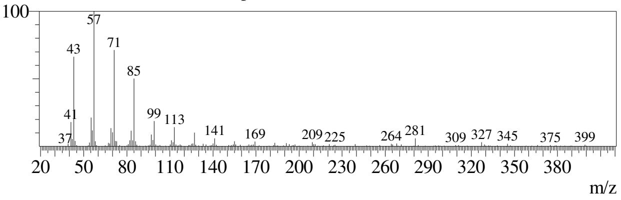

Line#:38 R.Time:33.165(Scan#:5634)
MassPeaks:193
RawMode:Averaged 33.160-33.170(5633-5635) BasePeak:43(1548)
BG Mode:Calc. from Peak Group 1 - Event 1

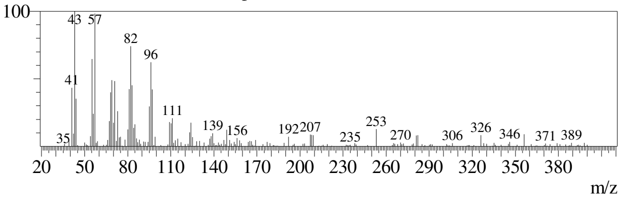

Line#:39 R.Time:35.975(Scan#:6196)
MassPeaks:221
RawMode:Averaged 35.970-35.980(6195-6197) BasePeak:57(103610)
BG Mode:Calc. from Peak Group 1 - Event 1

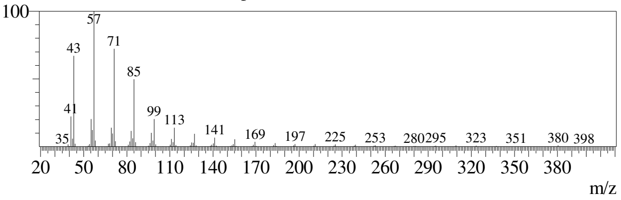

Line#:40 R.Time:41.005(Scan#:7202)
MassPeaks:196
RawMode:Averaged 41.000-41.010(7201-7203) BasePeak:57(6433)
BG Mode:Calc. from Peak Group 1 - Event 1

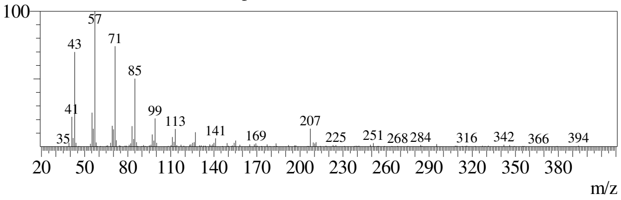

Line#:41 R.Time:42.630(Scan#:7527)
MassPeaks:230
RawMode:Averaged 42.625-42.635(7526-7528) BasePeak:69(36039)
BG Mode:Calc. from Peak Group 1 - Event 1

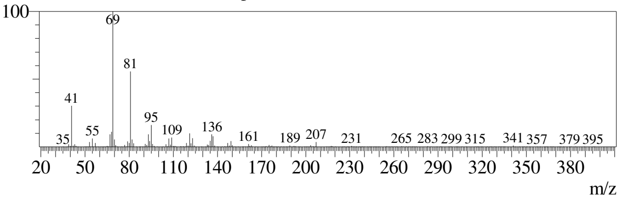

Female forewings extract

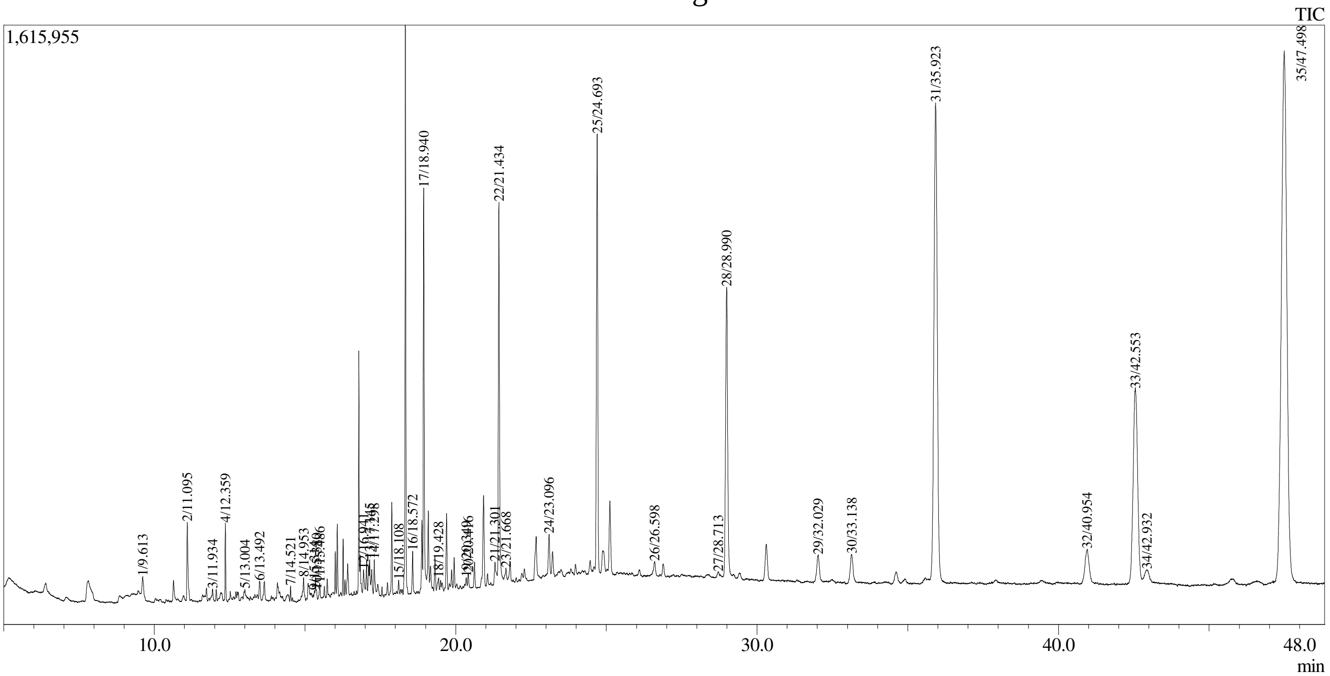

Peak Report TIC

Peak#	R.Time	I.Time	F.Time	Area	Area%	Height	Height%	A/H	Mark	Name
1	9.613	9.570	9.670	172355	0.32	51992	0.56	3.32	MI	Octanal
2	11.095	11.050	11.190	539990	1.02	213817	2.30	2.53	V	Nonanal
3	11.934	11.840	12.000	129929	0.24	35374	0.38	3.67	V	Nonanol
4	12.359	12.285	12.430	361335	0.68	212948	2.30	1.70	V	Decanal
5	13.004	12.985	13.030	63713	0.12	31703	0.34	2.01	V	2-Decenal
6	13.492	13.455	13.565	142445	0.27	52030	0.56	2.74	V	Undecanal
7	14.521	14.495	14.560	58364	0.11	41185	0.44	1.42		Dodecanal
8	14.953	14.890	15.020	213669	0.40	65880	0.71	3.24	V	Geranyl acetone
9	15.314	15.290	15.325	49443	0.09	28874	0.31	1.71	V	Pentadecene
10	15.380	15.370	15.425	51844	0.10	30870	0.33	1.68	V	Pentadecane
11	15.486	15.425	15.540	141236	0.27	48401	0.52	2.92	V	Tridecanal
12	16.941	16.905	17.000	248623	0.47	77264	0.83	3.22	V	Tetradecanol
13	17.145	17.125	17.175	210552	0.40	103425	1.11	2.04	V	2-pentadecanona
14	17.298	17.265	17.365	273438	0.51	102972	1.11	2.66	V	Pentadecanal
15	18.108	18.070	18.145	90950	0.17	41280	0.44	2.20	V	Octadecane
16	18.572	18.515	18.635	267447	0.50	116373	1.25	2.30	V	Trimethyl tridecatrienal
17	18.940	18.905	19.030	2431566	4.57	1092371	11.78	2.23	V	Hexadecanol
18	19.428	19.360	19.470	131551	0.25	36702	0.40	3.58	V	Methyl hexadecanoate
19	20.349	20.310	20.380	69496	0.13	28292	0.30	2.46	V	Eicosane
20	20.416	20.380	20.485	97304	0.18	40384	0.44	2.41	V	Hexadecanyl acetate
21	21.301	21.250	21.375	255158	0.48	61947	0.67	4.12	MI	(Z,E)-2,13-Octadecadienol
22	21.434	21.375	21.595	2881887	5.42	1030963	11.11	2.80	V	Octadecanol
23	21.668	21.595	21.720	182809	0.34	42449	0.46	4.31	V	Heneicosane
24	23.096	22.995	23.160	464422	0.87	120901	1.30	3.84	V	Docosane
25	24.693	24.620	24.800	3668399	6.90	1192576	12.86	3.08	V	Tricosane
26	26.598	26.505	26.685	213401	0.40	41481	0.45	5.14	V	Tetracosane
27	28.713	28.645	28.760	60367	0.11	14530	0.16	4.15		Docosanol
28	28.990	28.830	29.150	3736951	7.02	783726	8.45	4.77	V	Pentacosane
29	32.029	31.915	32.135	473452	0.89	75948	0.82	6.23	V	Hexacosane
30	33.138	33.020	33.290	488079	0.92	76649	0.83	6.37	S	Tetracosanal
31	35.923	35.735	36.160	9976013	18.75	1293451	13.94	7.71	V	Heptacosane
32	40.954	40.760	41.195	895599	1.68	92324	1.00	9.70		Octacosane
33	42.553	42.265	42.775	5507798	10.35	527280	5.68	10.45		Squalene
34	42.932	42.775	43.145	404106	0.76	35436	0.38	11.40	V	Hexacosanal
35	47.498	47.145	47.920	18246387	34.30	1434983	15.47	12.72	V	Nonacosane
				53200078	100.00	9276781	100.00			

Spectrum

Line#:1 R.Time:9.610(Scan#:923)
MassPeaks:226
RawMode:Averaged 9.605-9.615(922-924) BasePeak:281(6694)
BG Mode:Calc. from Peak Group 1 - Event 1

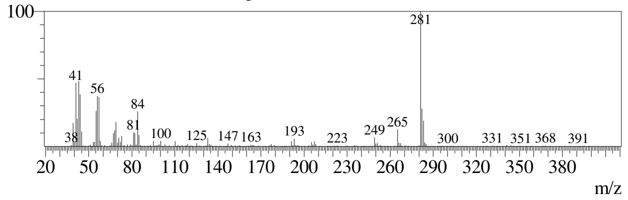

Line#:2 R.Time:11.095(Scan#:1220)
MassPeaks:247
RawMode:Averaged 11.090-11.100(1219-1221) BasePeak:57(23474)
BG Mode:Calc. from Peak Group 1 - Event 1

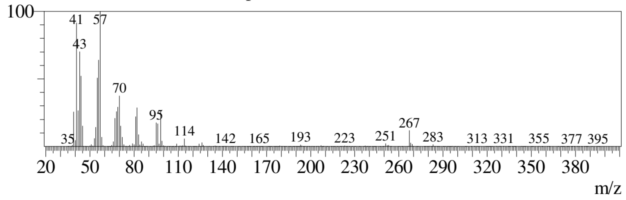

Line#:3 R.Time:11.935(Scan#:1388)
MassPeaks:211
RawMode:Averaged 11.930-11.940(1387-1389) BasePeak:41(3195)
BG Mode:Calc. from Peak Group 1 - Event 1

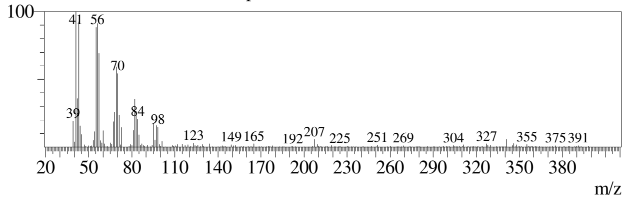

Line#:4 R.Time:12.360(Scan#:1473)
MassPeaks:210
RawMode:Averaged 12.355-12.365(1472-1474) BasePeak:41(18891)
BG Mode:Calc. from Peak Group 1 - Event 1

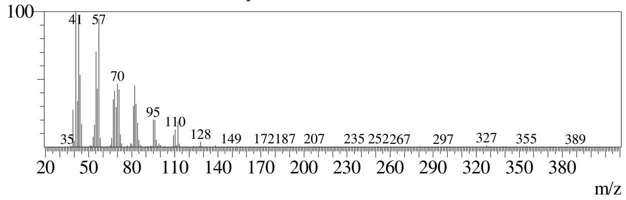

Line#:5 R.Time:13.005(Scan#:1602)
MassPeaks:218
RawMode:Averaged 13.000-13.010(1601-1603) BasePeak:41(962)
BG Mode:Calc. from Peak Group 1 - Event 1

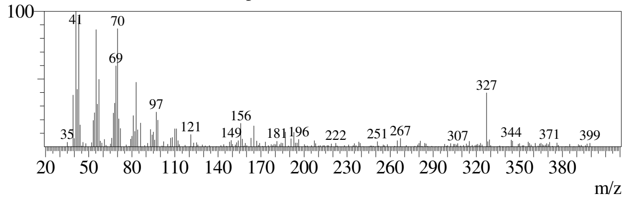

Line#:6 R.Time:13.490(Scan#:1699)
MassPeaks:253
RawMode:Averaged 13.485-13.495(1698-1700) BasePeak:281(3488)
BG Mode:Calc. from Peak Group 1 - Event 1

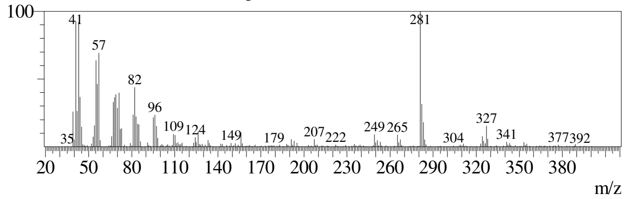

Line#:7 R.Time:14.520(Scan#:1905)
MassPeaks:215
RawMode:Averaged 14.515-14.525(1904-1906) BasePeak:43(3098)
BG Mode:Calc. from Peak Group 1 - Event 1

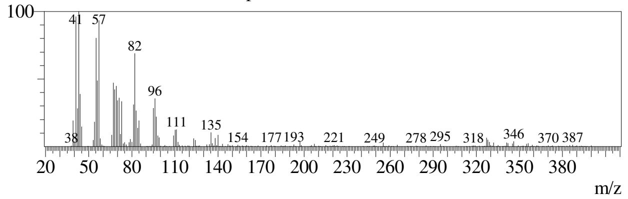

Line#:8 R.Time:14.955(Scan#:1992)
MassPeaks:263
RawMode:Averaged 14.950-14.960(1991-1993) BasePeak:43(12497)
BG Mode:Calc. from Peak Group 1 - Event 1

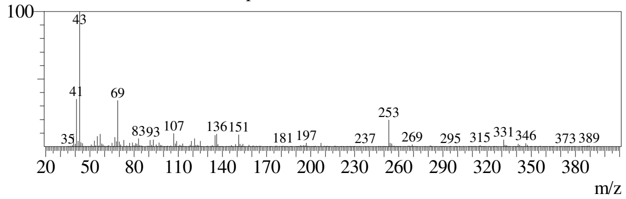

Line#:9 R.Time:15.315(Scan#:2064)
MassPeaks:164
RawMode:Averaged 15.310-15.320(2063-2065) BasePeak:41(859)
BG Mode:Calc. from Peak Group 1 - Event 1

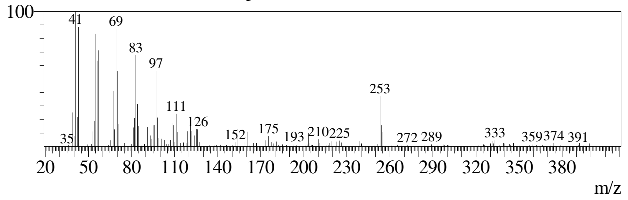

Line#:10 R.Time:15.380(Scan#:2077)
MassPeaks:171
RawMode:Averaged 15.375-15.385(2076-2078) BasePeak:57(1762)
BG Mode:Calc. from Peak Group 1 - Event 1

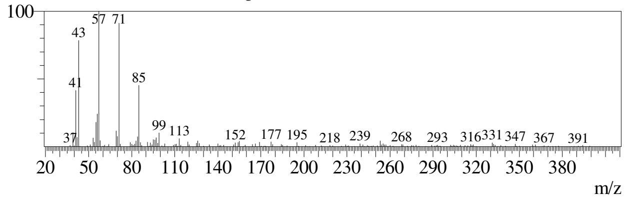

Line#:11 R.Time:15.485(Scan#:2098)
MassPeaks:204
RawMode:Averaged 15.480-15.490(2097-2099) BasePeak:41(3199)
BG Mode:Calc. from Peak Group 1 - Event 1

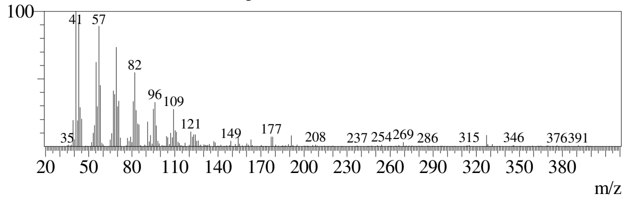

Line#:12 R.Time:16.940(Scan#:2389)
MassPeaks:201
RawMode:Averaged 16.935-16.945(2388-2390) BasePeak:43(4642)
BG Mode:Calc. from Peak Group 1 - Event 1

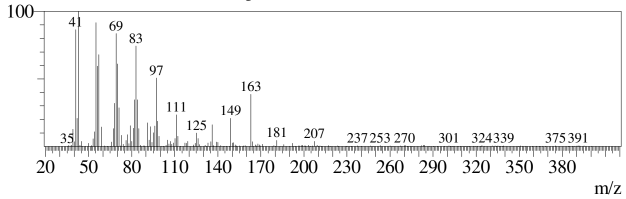

Line#:13 R.Time:17.145(Scan#:2430)
MassPeaks:174
RawMode:Averaged 17.140-17.150(2429-2431) BasePeak:58(11151)
BG Mode:Calc. from Peak Group 1 - Event 1

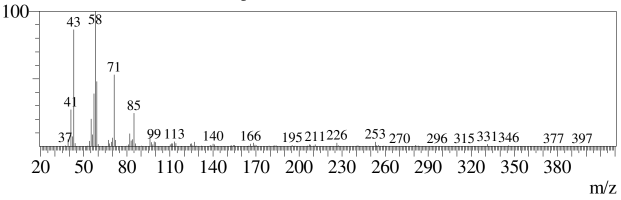

Line#:14 R.Time:17.300(Scan#:2461)
MassPeaks:243
RawMode:Averaged 17.295-17.305(2460-2462) BasePeak:43(7312)
BG Mode:Calc. from Peak Group 1 - Event 1

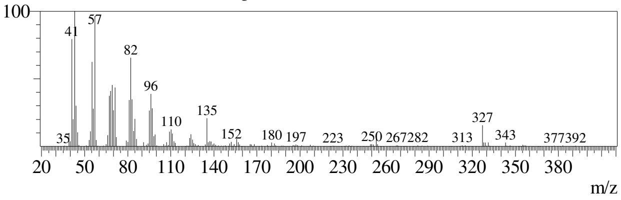

Line#:15 R.Time:18.105(Scan#:2622)
MassPeaks:188
RawMode:Averaged 18.100-18.110(2621-2623) BasePeak:57(6299)
BG Mode:Calc. from Peak Group 1 - Event 1

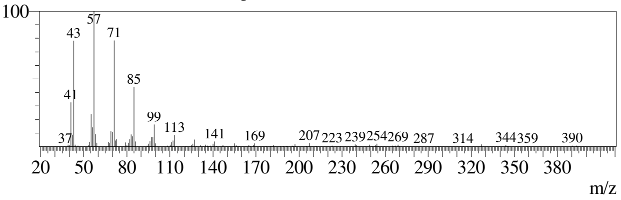

Line#:16 R.Time:18.570(Scan#:2715)
MassPeaks:236
RawMode:Averaged 18.565-18.575(2714-2716) BasePeak:69(23772)
BG Mode:Calc. from Peak Group 1 - Event 1

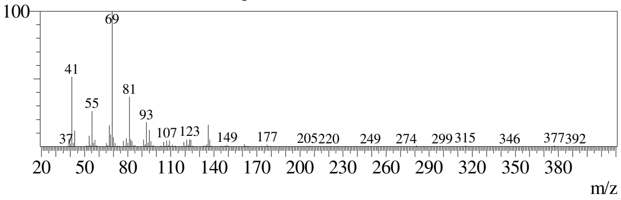

Line#:17 R.Time:18.940(Scan#:2789)
MassPeaks:259
RawMode:Averaged 18.935-18.945(2788-2790) BasePeak:43(86145)
BG Mode:Calc. from Peak Group 1 - Event 1

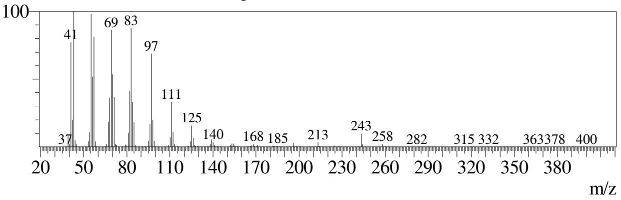

Line#:18 R.Time:19.425(Scan#:2886)
MassPeaks:228
RawMode:Averaged 19.420-19.430(2885-2887) BasePeak:74(4904)
BG Mode:Calc. from Peak Group 1 - Event 1

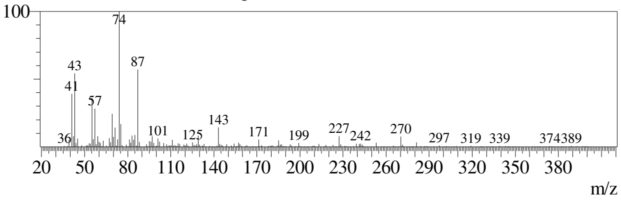

Line#:19 R.Time:20.350(Scan#:3071)
MassPeaks:170
RawMode:Averaged 20.345-20.355(3070-3072) BasePeak:57(4207)
BG Mode:Calc. from Peak Group 1 - Event 1

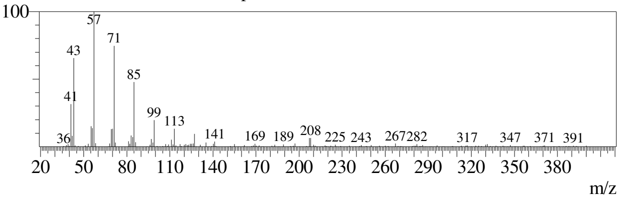

Line#:20 R.Time:20.415(Scan#:3084)
MassPeaks:239
RawMode:Averaged 20.410-20.420(3083-3085) BasePeak:43(3977)
BG Mode:Calc. from Peak Group 1 - Event 1

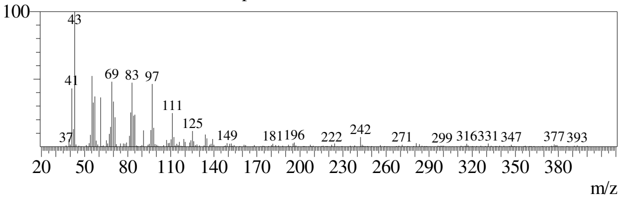

Line#:21 R.Time:21.300(Scan#:3261)
MassPeaks:207
RawMode:Averaged 21.295-21.305(3260-3262) BasePeak:55(4901)
BG Mode:Calc. from Peak Group 1 - Event 1

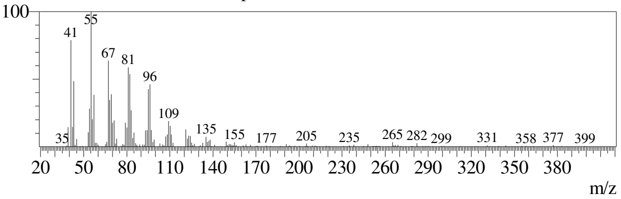

Line#:22 R.Time:21.435(Scan#:3288)
MassPeaks:238
RawMode:Averaged 21.430-21.440(3287-3289) BasePeak:43(83174)
BG Mode:Calc. from Peak Group 1 - Event 1

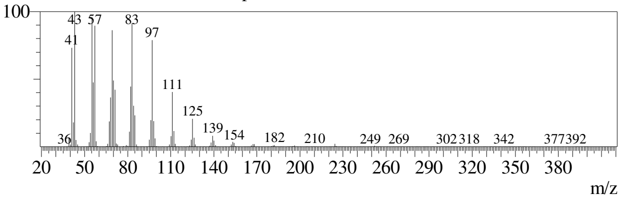

Line#:23 R.Time:21.670(Scan#:3335)
MassPeaks:215
RawMode:Averaged 21.665-21.675(3334-3336) BasePeak:57(5136)
BG Mode:Calc. from Peak Group 1 - Event 1

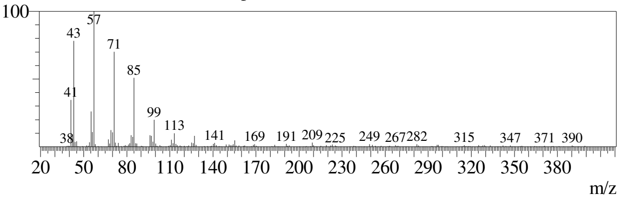

Line#:24 R.Time:23.095(Scan#:3620)
MassPeaks:240
RawMode:Averaged 23.090-23.100(3619-3621) BasePeak:57(15274)
BG Mode:Calc. from Peak Group 1 - Event 1

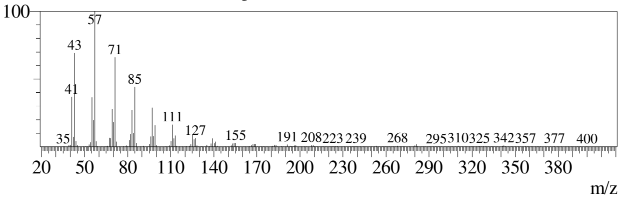

Line#:25 R.Time:24.695(Scan#:3940)
MassPeaks:240
RawMode:Averaged 24.690-24.700(3939-3941) BasePeak:57(210481)
BG Mode:Calc. from Peak Group 1 - Event 1

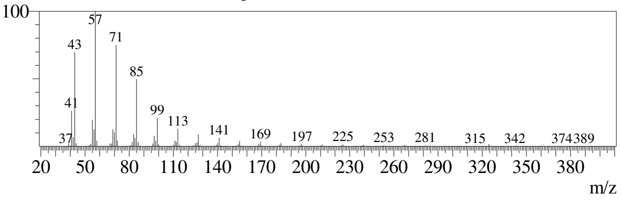

Line#:26 R.Time:26.600(Scan#:4321)
MassPeaks:212
RawMode:Averaged 26.595-26.605(4320-4322) BasePeak:57(6708)
BG Mode:Calc. from Peak Group 1 - Event 1

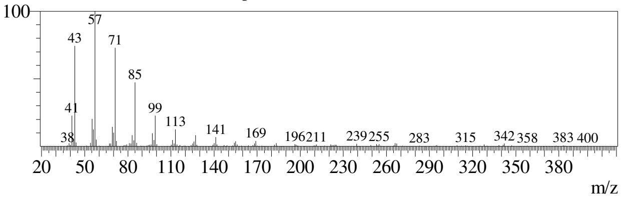

Line#:27 R.Time:28.715(Scan#:4744)
MassPeaks:215
RawMode:Averaged 28.710-28.720(4743-4745) BasePeak:43(1113)
BG Mode:Calc. from Peak Group 1 - Event 1

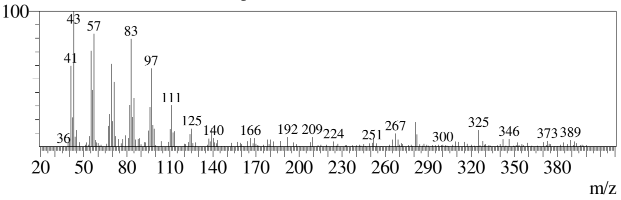

Line#:28 R.Time:28.990(Scan#:4799)
MassPeaks:203
RawMode:Averaged 28.985-28.995(4798-4800) BasePeak:57(137857)
BG Mode:Calc. from Peak Group 1 - Event 1

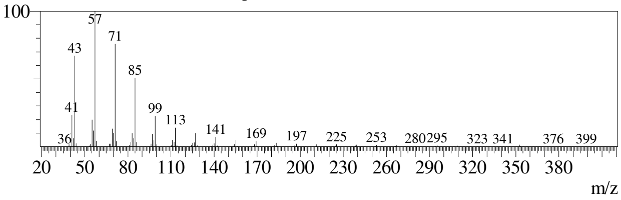

Line#:29 R.Time:32.030(Scan#:5407)
MassPeaks:242
RawMode:Averaged 32.025-32.035(5406-5408) BasePeak:57(11913)
BG Mode:Calc. from Peak Group 1 - Event 1

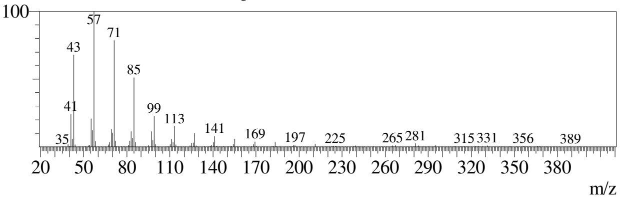

Line#:30 R.Time:33.140(Scan#:5629)
MassPeaks:251
RawMode:Averaged 33.135-33.145(5628-5630) BasePeak:57(6796)
BG Mode:Calc. from Peak Group 1 - Event 1

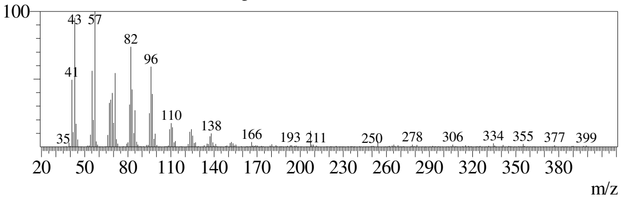

Line#:31 R.Time:35.925(Scan#:6186)
MassPeaks:265
RawMode:Averaged 35.920-35.930(6185-6187) BasePeak:57(222183)
BG Mode:Calc. from Peak Group 1 - Event 1

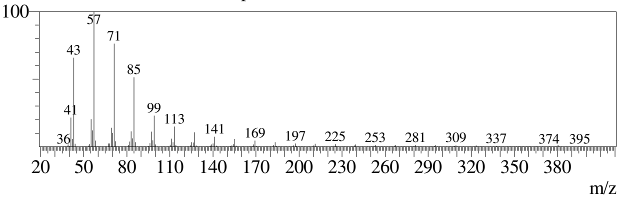

Line#:32 R.Time:40.955(Scan#:7192)
MassPeaks:242
RawMode:Averaged 40.950-40.960(7191-7193) BasePeak:57(15296)
BG Mode:Calc. from Peak Group 1 - Event 1

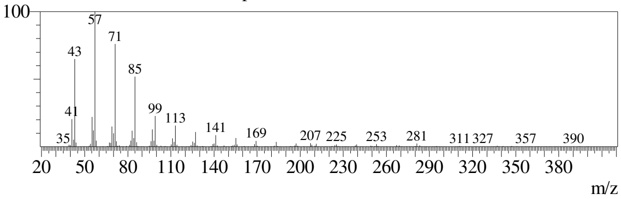

Line#:33 R.Time:42.555(Scan#:7512)
MassPeaks:285
RawMode:Averaged 42.550-42.560(7511-7513) BasePeak:69(128452)
BG Mode:Calc. from Peak Group 1 - Event 1

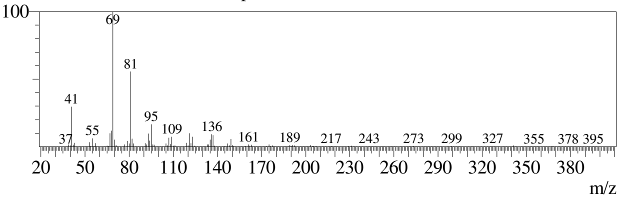

Line#:34 R.Time:42.930(Scan#:7587)
MassPeaks:196
RawMode:Averaged 42.925-42.935(7586-7588) BasePeak:57(3269)
BG Mode:Calc. from Peak Group 1 - Event 1

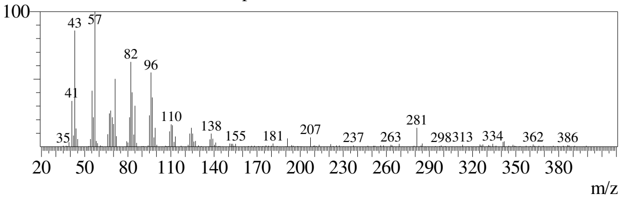

Line#:35 R.Time:47.500(Scan#:8501)
MassPeaks:253
RawMode:Averaged 47.495-47.505(8500-8502) BasePeak:57(239552)
BG Mode:Calc. from Peak Group 1 - Event 1

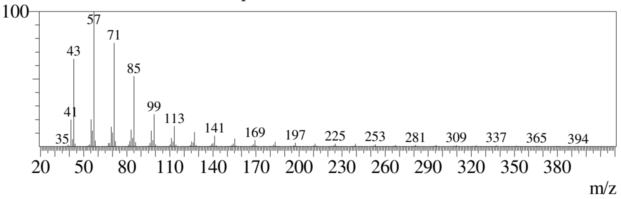

Female legs extract

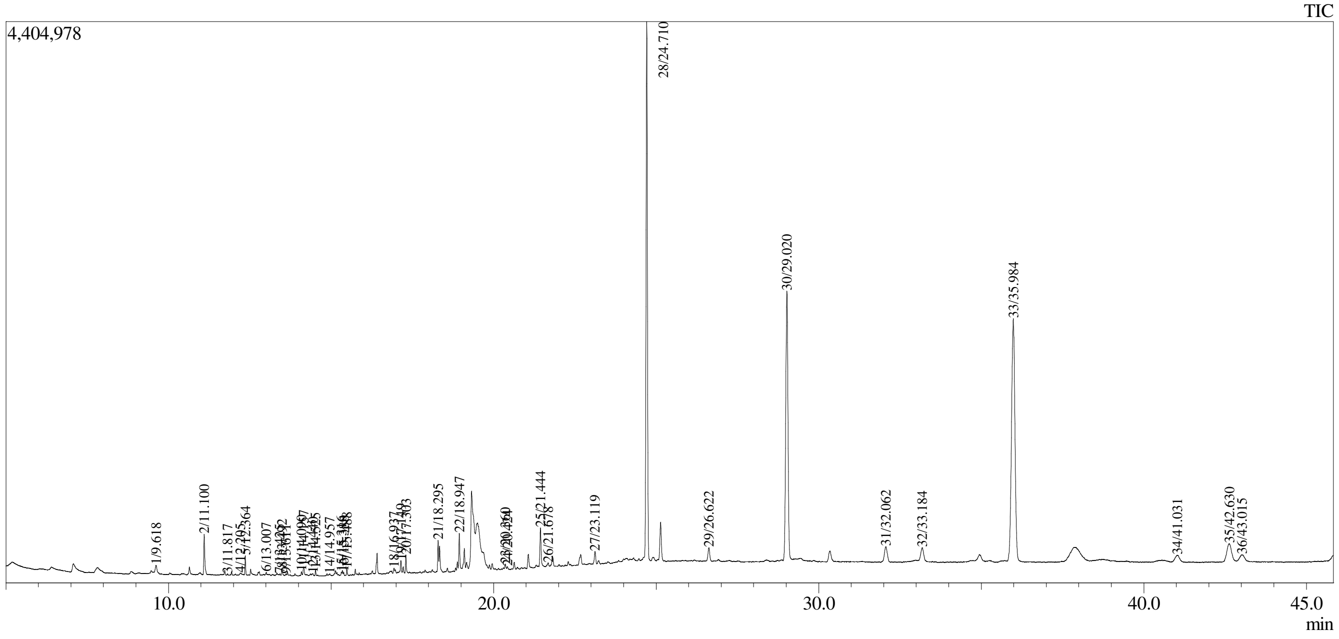

Peak Report TIC

Peak#	R.Time	I.Time	F.Time	Area	Area%	Height	Height%	A/H	Mark	Name
1	9.618	9.555	9.730	275556	0.60	66545	0.60	4.14	MI	Octanal
2	11.100	11.065	11.190	697324	1.52	317969	2.87	2.19		Nonanal
3	11.817	11.785	11.850	22451	0.05	12059	0.11	1.86		2-Nonenal
4	12.205	12.175	12.335	44264	0.10	11920	0.11	3.71		2-Decanona
5	12.364	12.335	12.410	229871	0.50	151789	1.37	1.51		Decanal
6	13.007	12.990	13.035	26346	0.06	19316	0.17	1.36	V	2-Decenal
7	13.425	13.410	13.445	12932	0.03	10909	0.10	1.19	MI	Tridecane
8	13.492	13.455	13.530	89847	0.20	50525	0.46	1.78		Undecanal
9	13.611	13.590	13.625	14963	0.03	12128	0.11	1.23	MI	Decadienal
10	14.090	14.055	14.115	49798	0.11	30338	0.27	1.64		Undecenal
11	14.157	14.115	14.205	158613	0.35	62735	0.57	2.53	V	Undecanol
12	14.440	14.400	14.480	41709	0.09	15947	0.14	2.62	V	Tetradecane
13	14.525	14.490	14.565	74451	0.16	52197	0.47	1.43		Dodecanal
14	14.957	14.900	15.000	33932	0.07	13129	0.12	2.58	V	Geranyl acetone
15	15.316	15.285	15.330	36161	0.08	25553	0.23	1.42		Pentadecene
16	15.388	15.370	15.420	37348	0.08	25989	0.23	1.44	V	Pentadecane
17	15.488	15.455	15.540	81106	0.18	50587	0.46	1.60		Tridecanal
18	16.937	16.895	16.960	89136	0.19	40966	0.37	2.18		Tetradecanol
19	17.149	17.120	17.180	175189	0.38	99477	0.90	1.76	V	2-pentadecanona
20	17.303	17.260	17.365	244859	0.53	139260	1.26	1.76		Pentadecanal
21	18.295	18.255	18.320	459620	1.00	247813	2.24	1.85		Hexadecanal
22	18.947	18.910	19.015	699159	1.53	298980	2.70	2.34	V	Hexadecanol
23	20.360	20.330	20.400	101413	0.22	43433	0.39	2.33	V	Eicosane
24	20.424	20.400	20.480	50627	0.11	22234	0.20	2.28	V	Hexadecanyl acetate
25	21.444	21.340	21.560	851312	1.86	308640	2.79	2.76	V	Octadecanol
26	21.678	21.560	21.725	106441	0.23	29344	0.26	3.63	V	Heneicosane
27	23.119	23.055	23.175	298337	0.65	99984	0.90	2.98		Docosane
28	24.710	24.635	24.815	12764845	27.84	4230212	38.18	3.02	V	Tricosane
29	26.622	26.545	26.705	394337	0.86	104267	0.94	3.78		Tetracosane
30	29.020	28.905	29.160	9631798	21.01	2110173	19.04	4.56		Pentacosane
31	32.062	31.955	32.195	719059	1.57	119704	1.08	6.01		Hexacosane
32	33.184	33.080	33.300	596521	1.30	100956	0.91	5.91		Tetracosanal
33	35.984	35.805	36.200	14100858	30.76	1904107	17.18	7.41		Heptacosane
34	41.031	40.835	41.245	519079	1.13	53413	0.48	9.72		Octacosane
35	42.630	42.405	42.855	1496069	3.26	142063	1.28	10.53		Squalene
36	43.015	42.855	43.275	618622	1.35	55857	0.50	11.08	V	Hexacosanal
				45843953	100.00	11080518	100.00			

Spectrum

Line#:1 R.Time:9.620(Scan#:925)
MassPeaks:206
RawMode:Averaged 9.615-9.625(924-926) BasePeak:281(7493)
BG Mode:Calc. from Peak Group 1 - Event 1

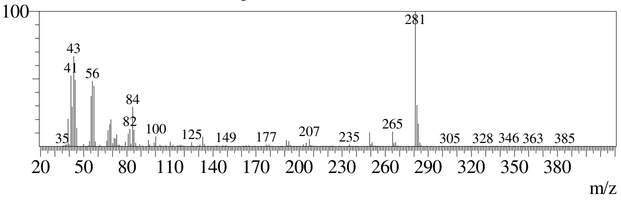

Line#:2 R.Time:11.100(Scan#:1221)
MassPeaks:235
RawMode:Averaged 11.095-11.105(1220-1222) BasePeak:57(34464)
BG Mode:Calc. from Peak Group 1 - Event 1

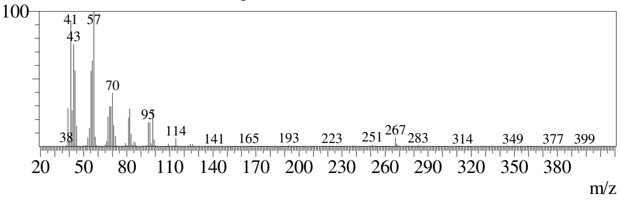

Line#:3 R.Time:11.815(Scan#:1364)
MassPeaks:225
RawMode:Averaged 11.810-11.820(1363-1365) BasePeak:41(1113)
BG Mode:Calc. from Peak Group 1 - Event 1

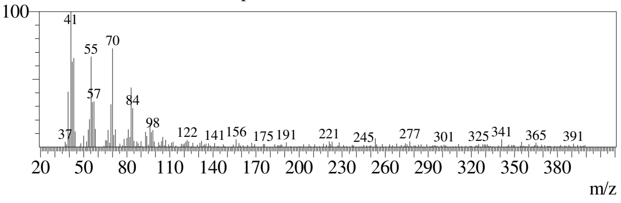

Line#:4 R.Time:12.205(Scan#:1442)
MassPeaks:197
RawMode:Averaged 12.200-12.210(1441-1443) BasePeak:43(2195)
BG Mode:Calc. from Peak Group 1 - Event 1

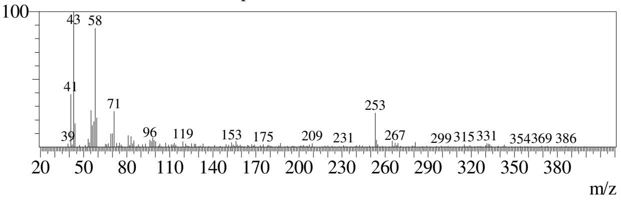

Line#:5 R.Time:12.365(Scan#:1474)
MassPeaks:208
RawMode:Averaged 12.360-12.370(1473-1475) BasePeak:41(14107)
BG Mode:Calc. from Peak Group 1 - Event 1

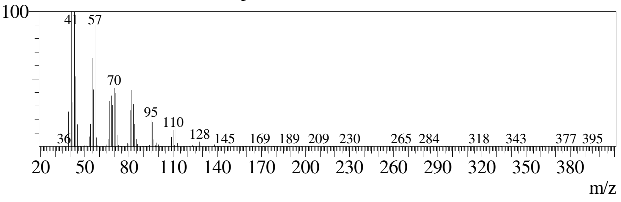

Line#:6 R.Time:13.005(Scan#:1602)
MassPeaks:161
RawMode:Averaged 13.000-13.010(1601-1603) BasePeak:43(1842)
BG Mode:Calc. from Peak Group 1 - Event 1

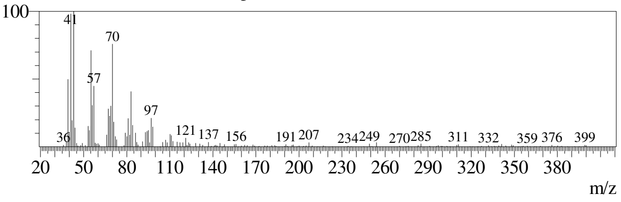

Line#:7 R.Time:13.425(Scan#:1686)
MassPeaks:203
RawMode:Averaged 13.420-13.430(1685-1687) BasePeak:57(1363)
BG Mode:Calc. from Peak Group 1 - Event 1

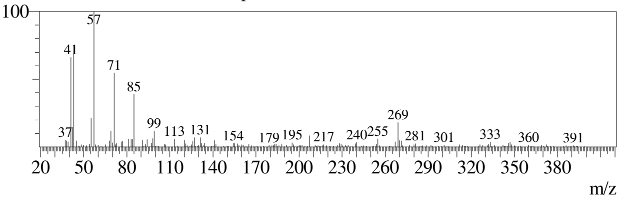

Line#:8 R.Time:13.490(Scan#:1699)
MassPeaks:186
RawMode:Averaged 13.485-13.495(1698-1700) BasePeak:43(4400)
BG Mode:Calc. from Peak Group 1 - Event 1

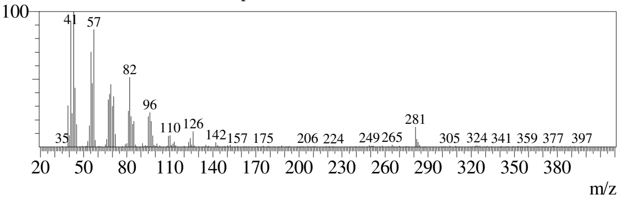

Line#:9 R.Time:13.610(Scan#:1723)
MassPeaks:197
RawMode:Averaged 13.605-13.615(1722-1724) BasePeak:81(2125)
BG Mode:Calc. from Peak Group 1 - Event 1

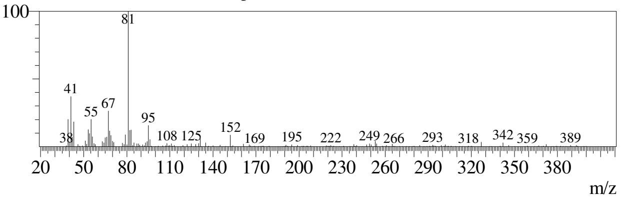

Line#:10 R.Time:14.090(Scan#:1819)
MassPeaks:186
RawMode:Averaged 14.085-14.095(1818-1820) BasePeak:85(2816)
BG Mode:Calc. from Peak Group 1 - Event 1

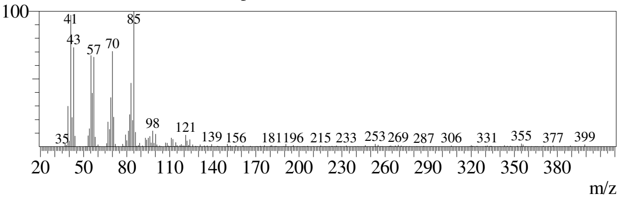

Line#:11 R.Time:14.155(Scan#:1832)
MassPeaks:244
RawMode:Averaged 14.150-14.160(1831-1833) BasePeak:43(4809)
BG Mode:Calc. from Peak Group 1 - Event 1

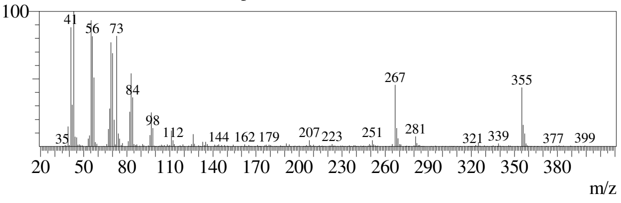

Line#:12 R.Time:14.440(Scan#:1889)
MassPeaks:191
RawMode:Averaged 14.435-14.445(1888-1890) BasePeak:57(2597)
BG Mode:Calc. from Peak Group 1 - Event 1

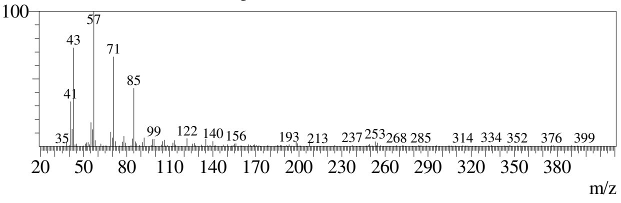

Line#:13 R.Time:14.525(Scan#:1906)
MassPeaks:229
RawMode:Averaged 14.520-14.530(1905-1907) BasePeak:41(4384)
BG Mode:Calc. from Peak Group 1 - Event 1

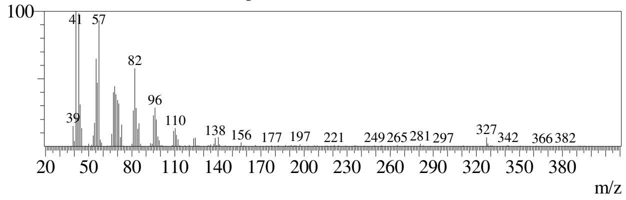

Line#:14 R.Time:14.955(Scan#:1992)
MassPeaks:206
RawMode:Averaged 14.950-14.960(1991-1993) BasePeak:43(3140)
BG Mode:Calc. from Peak Group 1 - Event 1

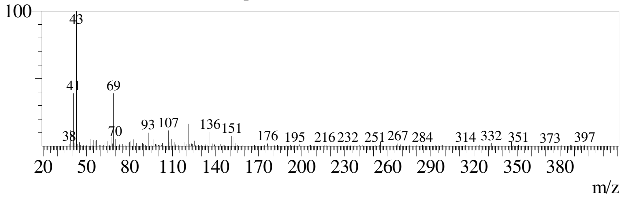

Line#:15 R.Time:15.315(Scan#:2064)
MassPeaks:196
RawMode:Averaged 15.310-15.320(2063-2065) BasePeak:41(1385)
BG Mode:Calc. from Peak Group 1 - Event 1

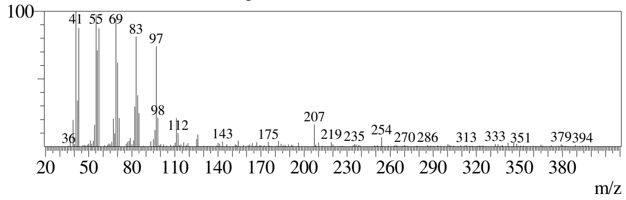

Line#:16 R.Time:15.390(Scan#:2079)
MassPeaks:189
RawMode:Averaged 15.385-15.395(2078-2080) BasePeak:57(4252)
BG Mode:Calc. from Peak Group 1 - Event 1

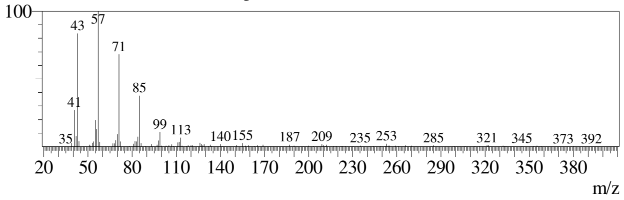

Line#:17 R.Time:15.490(Scan#:2099)
MassPeaks:186
RawMode:Averaged 15.485-15.495(2098-2100) BasePeak:57(4291)
BG Mode:Calc. from Peak Group 1 - Event 1

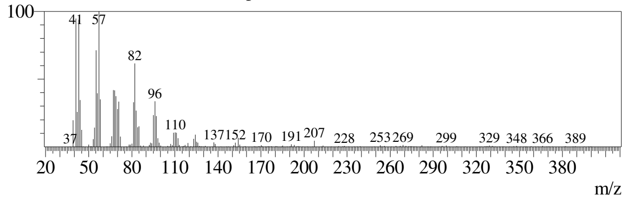

Line#:18 R.Time:16.935(Scan#:2388)
MassPeaks:206
RawMode:Averaged 16.930-16.940(2387-2389) BasePeak:41(2480)
BG Mode:Calc. from Peak Group 1 - Event 1

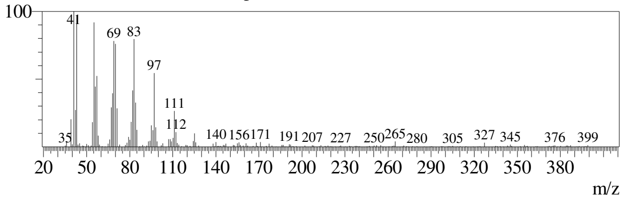

Line#:19 R.Time:17.150(Scan#:2431)
MassPeaks:195
RawMode:Averaged 17.145-17.155(2430-2432) BasePeak:58(16334)
BG Mode:Calc. from Peak Group 1 - Event 1

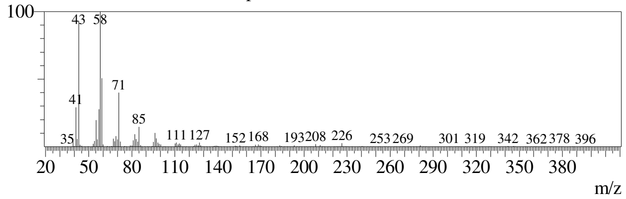

Line#:20 R.Time:17.305(Scan#:2462)
MassPeaks:223
RawMode:Averaged 17.300-17.310(2461-2463) BasePeak:43(12089)
BG Mode:Calc. from Peak Group 1 - Event 1

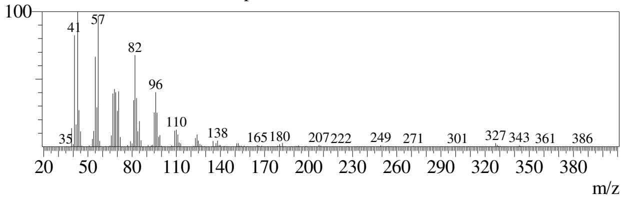

Line#:21 R.Time:18.295(Scan#:2660)
MassPeaks:189
RawMode:Averaged 18.290-18.300(2659-2661) BasePeak:43(17412)
BG Mode:Calc. from Peak Group 1 - Event 1

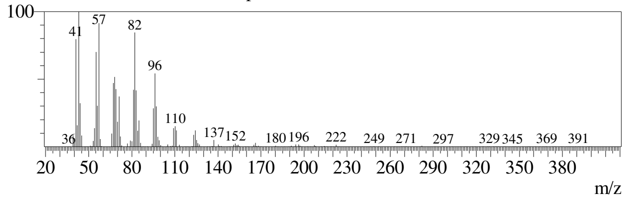

Line#:22 R.Time:18.945(Scan#:2790)
MassPeaks:241
RawMode:Averaged 18.940-18.950(2789-2791) BasePeak:43(24720)
BG Mode:Calc. from Peak Group 1 - Event 1

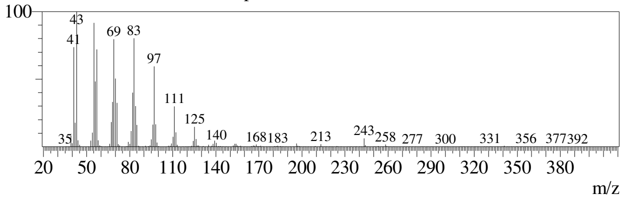

Line#:23 R.Time:20.360(Scan#:3073)
MassPeaks:211
RawMode:Averaged 20.355-20.365(3072-3074) BasePeak:57(5777)
BG Mode:Calc. from Peak Group 1 - Event 1

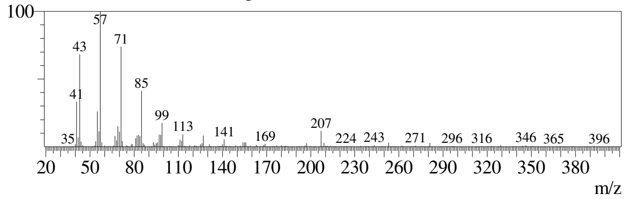

Line#:24 R.Time:20.425(Scan#:3086)
MassPeaks:240
RawMode:Averaged 20.420-20.430(3085-3087) BasePeak:43(1476)
BG Mode:Calc. from Peak Group 1 - Event 1

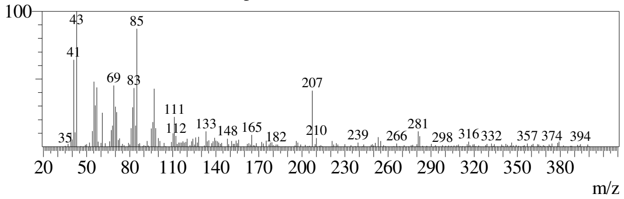

Line#:25 R.Time:21.445(Scan#:3290)
MassPeaks:258
RawMode:Averaged 21.440-21.450(3289-3291) BasePeak:43(25736)
BG Mode:Calc. from Peak Group 1 - Event 1

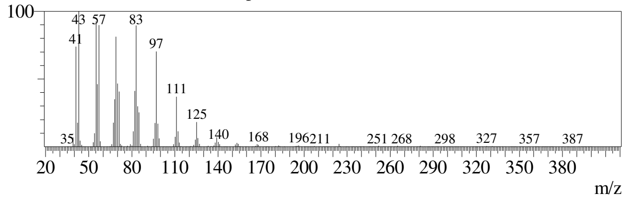

Line#:26 R.Time:21.680(Scan#:3337)
MassPeaks:212
RawMode:Averaged 21.675-21.685(3336-3338) BasePeak:57(4580)
BG Mode:Calc. from Peak Group 1 - Event 1

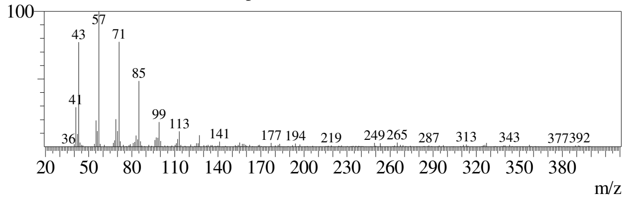

Line#:27 R.Time:23.120(Scan#:3625)
MassPeaks:218
RawMode:Averaged 23.115-23.125(3624-3626) BasePeak:57(17546)
BG Mode:Calc. from Peak Group 1 - Event 1

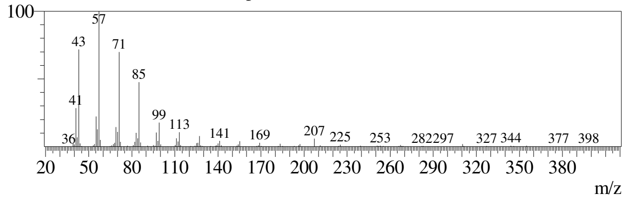

Line#:28 R.Time:24.710(Scan#:3943)
MassPeaks:278
RawMode:Averaged 24.705-24.715(3942-3944) BasePeak:57(767977)
BG Mode:Calc. from Peak Group 1 - Event 1

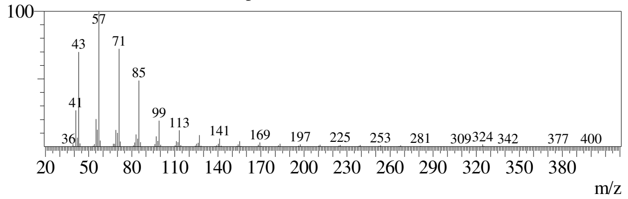

Line#:29 R.Time:26.620(Scan#:4325)
MassPeaks:219
RawMode:Averaged 26.615-26.625(4324-4326) BasePeak:57(18966)
BG Mode:Calc. from Peak Group 1 - Event 1

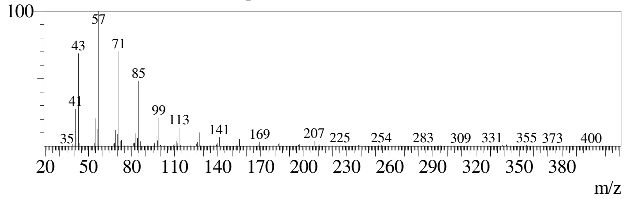

Line#:30 R.Time:29.020(Scan#:4805)
MassPeaks:277
RawMode:Averaged 29.015-29.025(4804-4806) BasePeak:57(377284)
BG Mode:Calc. from Peak Group 1 - Event 1

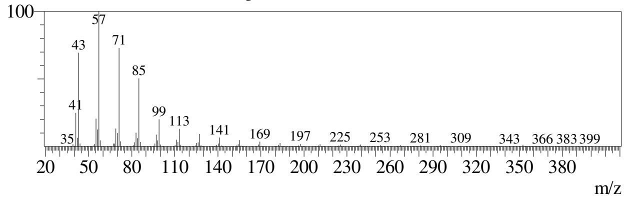

Line#:31 R.Time:32.060(Scan#:5413)
MassPeaks:240
RawMode:Averaged 32.055-32.065(5412-5414) BasePeak:57(21254)
BG Mode:Calc. from Peak Group 1 - Event 1

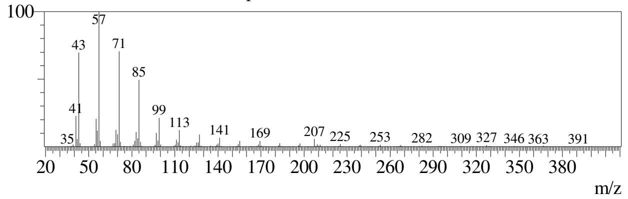

Line#:32 R.Time:33.185(Scan#:5638)
MassPeaks:258
RawMode:Averaged 33.180-33.190(5637-5639) BasePeak:57(8459)
BG Mode:Calc. from Peak Group 1 - Event 1

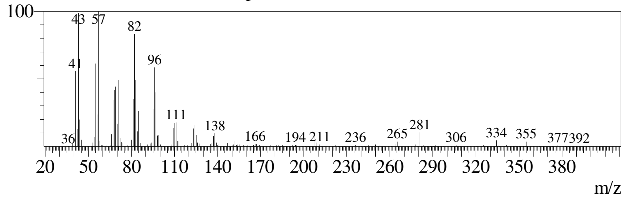

Line#:33 R.Time:35.985(Scan#:6198)
MassPeaks:242
RawMode:Averaged 35.980-35.990(6197-6199) BasePeak:57(335982)
BG Mode:Calc. from Peak Group 1 - Event 1

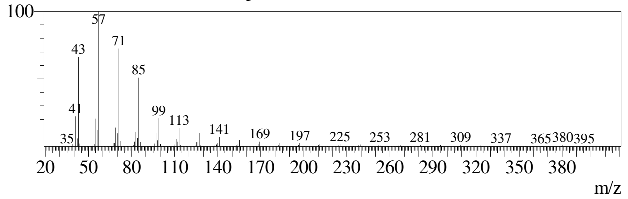

Line#:34 R.Time:41.030(Scan#:7207)
MassPeaks:229
RawMode:Averaged 41.025-41.035(7206-7208) BasePeak:57(9652)
BG Mode:Calc. from Peak Group 1 - Event 1

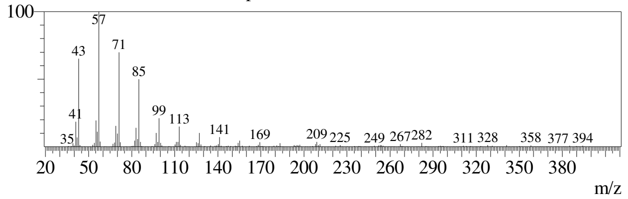

Line#:35 R.Time:42.630(Scan#:7527)
MassPeaks:249
RawMode:Averaged 42.625-42.635(7526-7528) BasePeak:69(35571)
BG Mode:Calc. from Peak Group 1 - Event 1

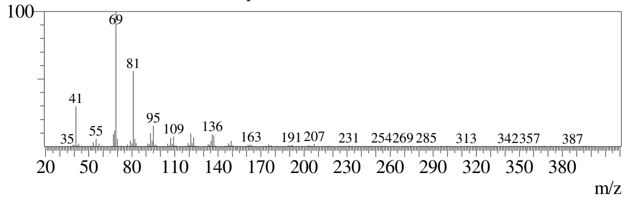

Line#:36 R.Time:43.015(Scan#:7604)
MassPeaks:235
RawMode:Averaged 43.010-43.020(7603-7605) BasePeak:43(4318)
BG Mode:Calc. from Peak Group 1 - Event 1

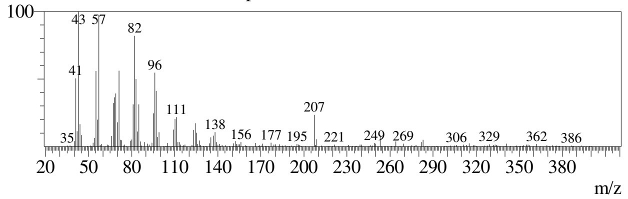

Female genitalia extract

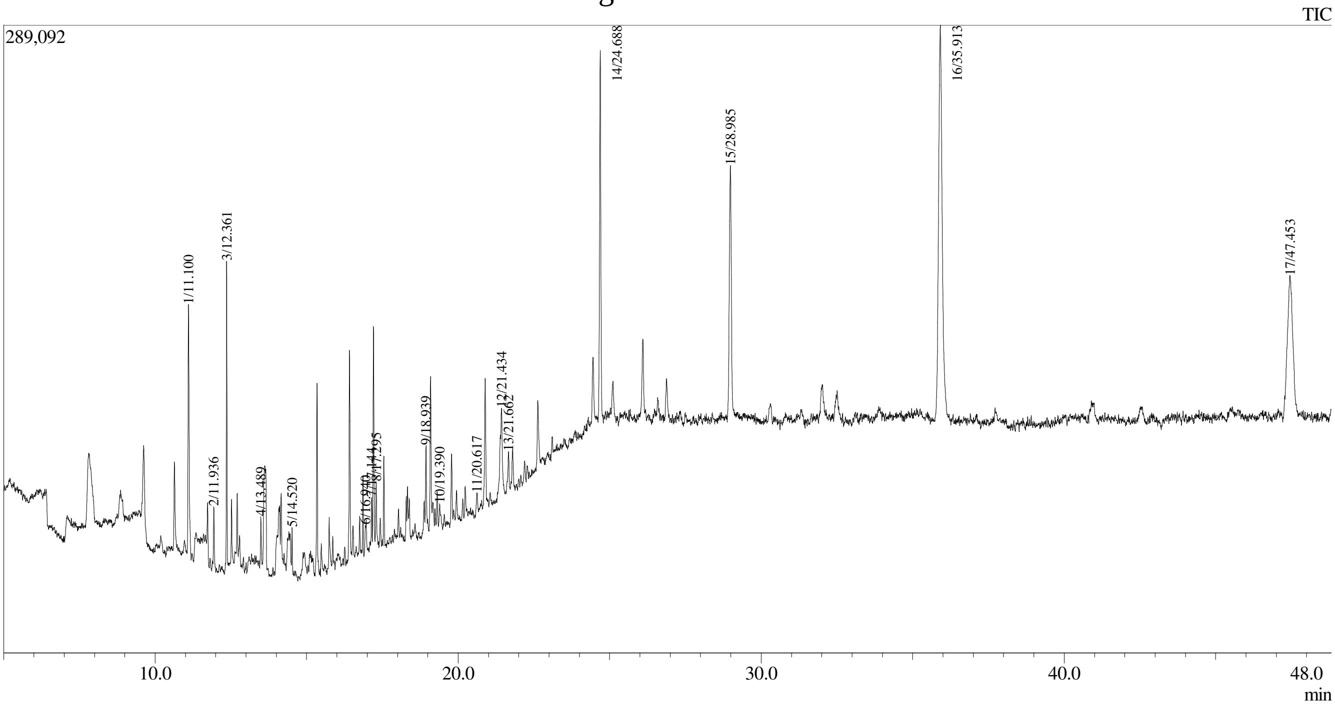

Peak Report TIC

Peak#	R.Time	I.Time	F.Time	Area	Area%	Height	Height%	A/H	Mark	Name
1	11.100	11.015	11.185	309522	6.50	116981	10.83	2.65	V	Nonanal
2	11.936	11.910	12.005	59703	1.25	29952	2.77	1.99	V	Nonanol
3	12.361	12.290	12.405	235120	4.94	141478	13.10	1.66		Decanal
4	13.489	13.450	13.530	63760	1.34	26700	2.47	2.39	V	Undecanal
5	14.520	14.495	14.555	40983	0.86	24039	2.23	1.70	V	Dodecanal
6	16.940	16.890	16.950	33228	0.70	16004	1.48	2.08	V	Tetradecanol
7	17.144	17.110	17.170	57464	1.21	27725	2.57	2.07	V	2-pentadecanona
8	17.295	17.250	17.360	92885	1.95	33827	3.13	2.75	V	Pentadecanal
9	18.939	18.900	18.980	106312	2.23	42078	3.89	2.53	V	Hexadecanol
10	19.390	19.350	19.420	34463	0.72	12970	1.20	2.66	V	Heptadecanal
11	20.617	20.570	20.670	30524	0.64	10137	0.94	3.01		Octadecanal
12	21.434	21.405	21.545	142949	3.00	42034	3.89	3.40	V	Octadecanol
13	21.662	21.545	21.720	73542	1.55	19842	1.84	3.71	V	Heneicosane
14	24.688	24.615	24.790	535425	11.25	171361	15.86	3.12	S	Tricosane
15	28.985	28.880	29.275	579538	12.18	117840	10.91	4.92	S	Pentacosane
16	35.913	35.675	36.135	1505047	31.63	181130	16.77	8.31	V	Heptacosane
17	47.453	47.215	47.720	858044	18.03	66227	6.13	12.96		Nonacosane
				4758509	100.00	1080325	100.00			

Spectrum

Line#:1 R.Time:11.100(Scan#:1221)
MassPeaks:247
RawMode:Averaged 11.095-11.105(1220-1222) BasePeak:57(12656)
BG Mode:Calc. from Peak Group 1 - Event 1

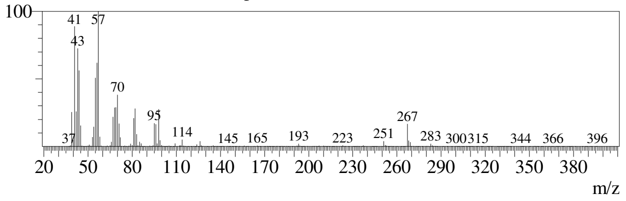

Line#:2 R.Time:11.935(Scan#:1388)
MassPeaks:202
RawMode:Averaged 11.930-11.940(1387-1389) BasePeak:41(2625)
BG Mode:Calc. from Peak Group 1 - Event 1

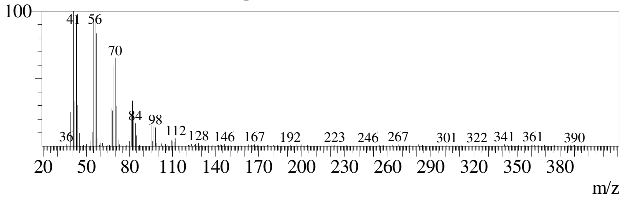

Line#:3 R.Time:12.360(Scan#:1473)
MassPeaks:223
RawMode:Averaged 12.355-12.365(1472-1474) BasePeak:41(12604)
BG Mode:Calc. from Peak Group 1 - Event 1

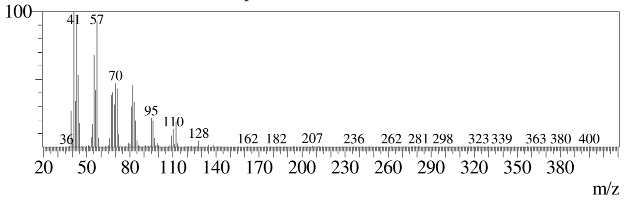

Line#:4 R.Time:13.490(Scan#:1699)
MassPeaks:198
RawMode:Averaged 13.485-13.495(1698-1700) BasePeak:43(1833)
BG Mode:Calc. from Peak Group 1 - Event 1

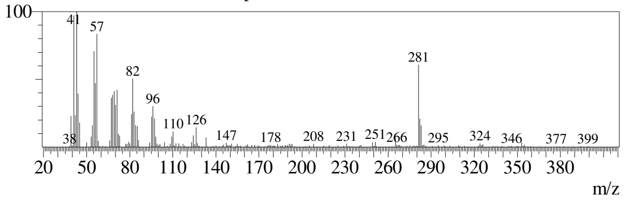

Line#:5 R.Time:14.520(Scan#:1905)
MassPeaks:213
RawMode:Averaged 14.515-14.525(1904-1906) BasePeak:43(1688)
BG Mode:Calc. from Peak Group 1 - Event 1

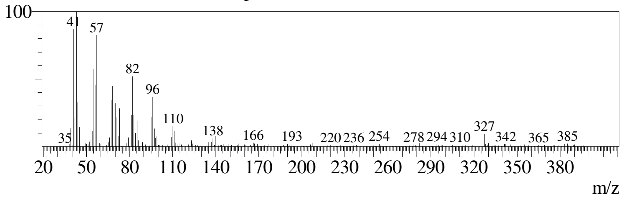

Line#:6 R.Time:16.940(Scan#:2389)
MassPeaks:184
RawMode:Averaged 16.935-16.945(2388-2390) BasePeak:43(528)
BG Mode:Calc. from Peak Group 1 - Event 1

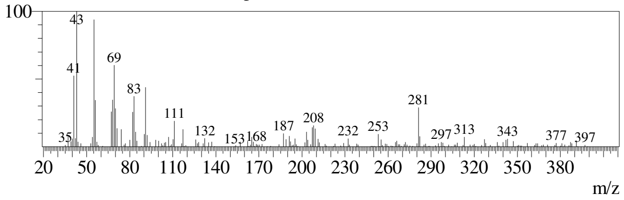

Line#:7 R.Time:17.145(Scan#:2430)
MassPeaks:242
RawMode:Averaged 17.140-17.150(2429-2431) BasePeak:58(3066)
BG Mode:Calc. from Peak Group 1 - Event 1

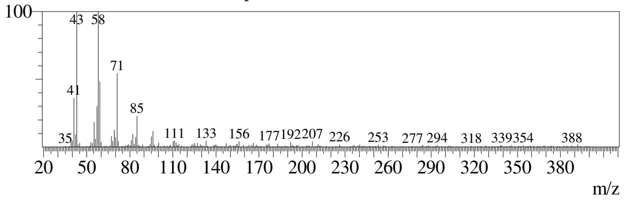

Line#:8 R.Time:17.295(Scan#:2460)
MassPeaks:233
RawMode:Averaged 17.290-17.300(2459-2461) BasePeak:43(2080)
BG Mode:Calc. from Peak Group 1 - Event 1

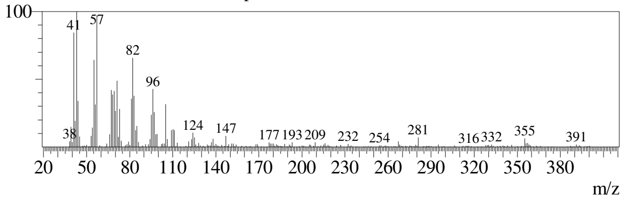

Line#:9 R.Time:18.940(Scan#:2789)
MassPeaks:221
RawMode:Averaged 18.935-18.945(2788-2790) BasePeak:43(3031)
BG Mode:Calc. from Peak Group 1 - Event 1

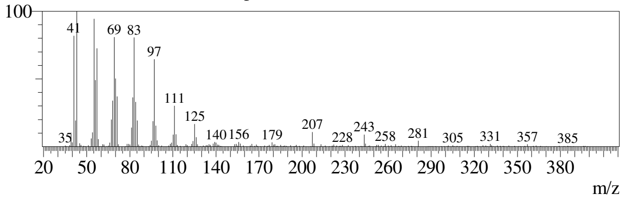

Line#:10 R.Time:19.390(Scan#:2879)
MassPeaks:221
RawMode:Averaged 19.385-19.395(2878-2880) BasePeak:207(501)
BG Mode:Calc. from Peak Group 1 - Event 1

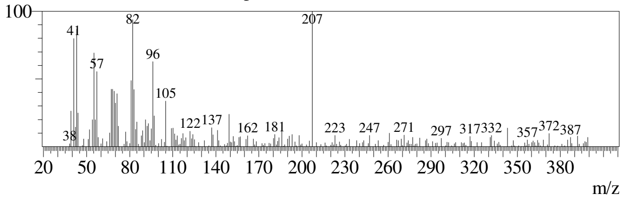

Line#:11 R.Time:20.615(Scan#:3124)
MassPeaks:220
RawMode:Averaged 20.610-20.620(3123-3125) BasePeak:57(888)
BG Mode:Calc. from Peak Group 1 - Event 1

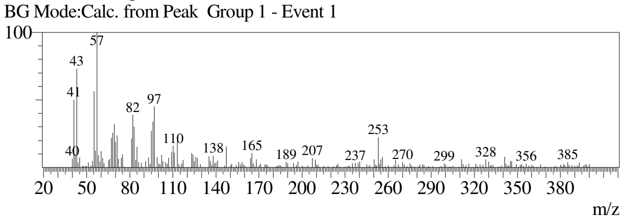

Line#:12 R.Time:21.435(Scan#:3288)
MassPeaks:236
RawMode:Averaged 21.430-21.440(3287-3289) BasePeak:43(1961)
BG Mode:Calc. from Peak Group 1 - Event 1

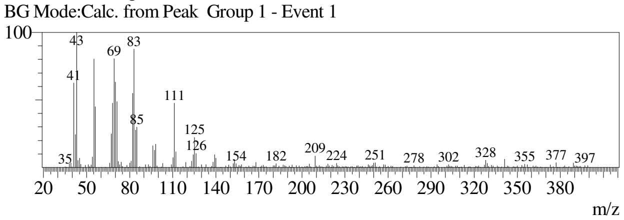

Line#:13 R.Time:21.660(Scan#:3333)
MassPeaks:221
RawMode:Averaged 21.655-21.665(3332-3334) BasePeak:57(3106)
BG Mode:Calc. from Peak Group 1 - Event 1

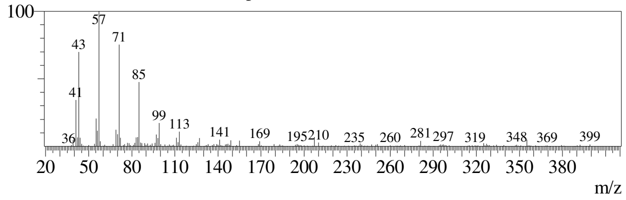

Line#:14 R.Time:24.690(Scan#:3939)
MassPeaks:247
RawMode:Averaged 24.685-24.695(3938-3940) BasePeak:57(30829)
BG Mode:Calc. from Peak Group 1 - Event 1

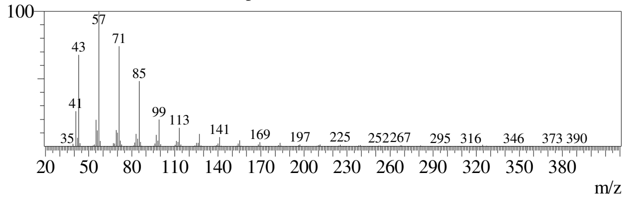

Line#:15 R.Time:28.985(Scan#:4798)
MassPeaks:242
RawMode:Averaged 28.980-28.990(4797-4799) BasePeak:57(20580)
BG Mode:Calc. from Peak Group 1 - Event 1

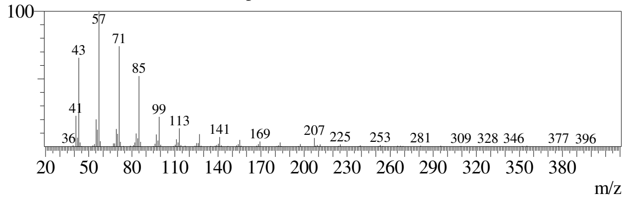

Line#:16 R.Time:35.915(Scan#:6184)
MassPeaks:221
RawMode:Averaged 35.910-35.920(6183-6185) BasePeak:57(30849)
BG Mode:Calc. from Peak Group 1 - Event 1

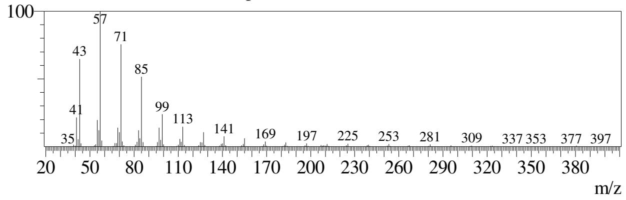

Line#:17 R.Time:47.455(Scan#:8492)
MassPeaks:230
RawMode:Averaged 47.450-47.460(8491-8493) BasePeak:57(10157)
BG Mode:Calc. from Peak Group 1 - Event 1

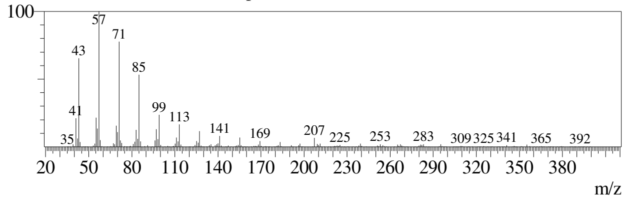

Supplement: S1 File — (PDF) [file pone.0231689.s003.pdf]
