# Supplementary material for: Morphological, chemical and electrophysiological investigations of Telchin licus (Lepidoptera: Castniidae)
Source: PLoS One. 2020 Apr 16;15(4):e0231689. doi: 10.1371/journal.pone.0231689 (PMC7162514; doi:10.1371/journal.pone.0231689)

Male abdomen extract

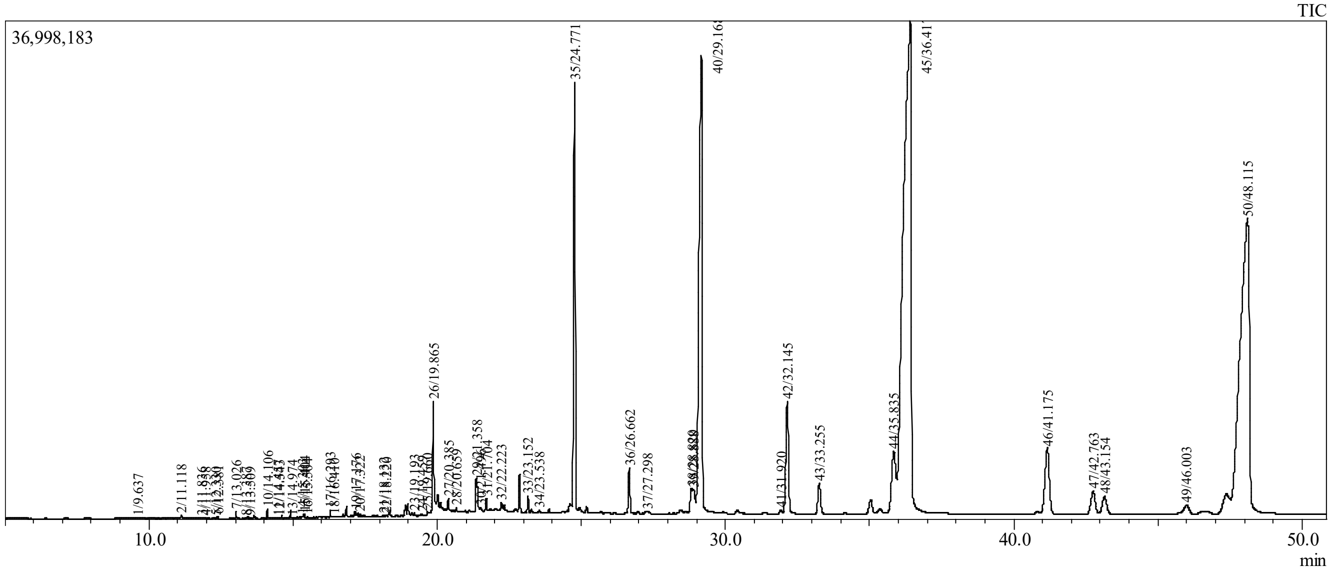

Peak Report TIC

Peak#	R.Time	I.Time	F.Time	Area	Area%	Height	Height%	A/H	Mark	Name
1	9.637	9.600	9.700	162117	0.01	59388	0.03	2.73		Octanal
2	11.118	11.070	11.175	594096	0.03	257641	0.15	2.31		Nonanal
3	11.836	11.805	11.875	141958	0.01	81532	0.05	1.74		2-Nonenal
4	11.958	11.945	12.010	40741	0.00	18062	0.01	2.26	MI	Nonanol
5	12.330	12.310	12.360	59208	0.00	30846	0.02	1.92		Dodecane
6	12.381	12.360	12.425	230712	0.01	150602	0.09	1.53	V	Decanal
7	13.026	12.985	13.070	839640	0.05	553154	0.32	1.52		2-Decenal
8	13.387	13.355	13.415	112363	0.01	54371	0.03	2.07		2,4-Decadienal
9	13.509	13.480	13.545	114134	0.01	71394	0.04	1.60		Undecanal
10	14.106	14.065	14.155	992030	0.05	680174	0.39	1.46		Undecenal
11	14.457	14.430	14.485	62910	0.00	46034	0.03	1.37		Tetradecane
12	14.543	14.515	14.575	123498	0.01	87261	0.05	1.42		Dodecanal
13	14.974	14.945	15.035	133653	0.01	65686	0.04	2.03	V	Geranyl acetone
14	15.303	15.270	15.320	226316	0.01	128354	0.07	1.76	V	Pentadecene
15	15.404	15.380	15.455	475145	0.03	291558	0.17	1.63	V	Pentadecane
16	15.504	15.460	15.530	122160	0.01	73443	0.04	1.66		Tridecanal
17	16.293	16.255	16.325	784731	0.04	527028	0.30	1.49		Hexadecane
18	16.410	16.325	16.435	249384	0.01	87631	0.05	2.85	V	Tetradecanal
19	17.176	17.155	17.200	669171	0.04	353383	0.20	1.89	V	Heptadecane
20	17.322	17.280	17.380	409603	0.02	187652	0.11	2.18		Pentadecanal
21	18.132	18.100	18.165	134280	0.01	76313	0.04	1.76		Octadecane
22	18.220	18.180	18.285	345647	0.02	97155	0.06	3.56		Hexadecenal
23	19.193	19.160	19.275	617399	0.03	210901	0.12	2.93	V	Nonadecano
24	19.459	19.395	19.495	396293	0.02	150085	0.09	2.64		Methyl hexadecanoate
25	19.660	19.620	19.735	929562	0.05	237255	0.14	3.92		(Z)-9-Hexadecenoic acid
26	19.865	19.735	19.960	25871088	1.39	8271036	4.75	3.13	V	Hexadecanoic acid
27	20.385	20.340	20.510	1860286	0.10	844794	0.48	2.20		Eicosane
28	20.659	20.615	20.705	547545	0.03	240960	0.14	2.27		Octadecanal
29	21.358	21.305	21.390	7846185	0.42	2499629	1.43	3.14	MI	(Z,E)-2,13-Octadecadienol
30	21.496	21.455	21.585	1109466	0.06	261051	0.15	4.25	V	Octadecanol
31	21.704	21.585	21.755	2556121	0.14	920061	0.53	2.78	V	Heneicosane
32	22.223	22.160	22.285	2046687	0.11	611998	0.35	3.34		(Z)-9-Octadecenoic acid
33	23.152	23.095	23.215	3189786	0.17	1191135	0.68	2.68		Docosane
34	23.538	23.490	23.590	343932	0.02	141582	0.08	2.43		Eicosanal
35	24.771	24.675	24.885	130232013	7.02	31912177	18.31	4.08	V	Tricosane
36	26.662	26.565	26.770	12857154	0.69	3382771	1.94	3.80		Tetracosane
37	27.298	27.265	27.420	956230	0.05	198159	0.11	4.83	V	Docosanal
38	28.820	28.710	28.860	9495015	0.51	1791169	1.03	5.30		Docosanol
39	28.888	28.860	28.975	9348333	0.50	1705048	0.98	5.48	V	Pentacosene
40	29.168	28.975	29.450	257718829	13.88	33934521	19.47	7.59	V	Pentacosane
41	31.920	31.810	31.995	1549708	0.08	260151	0.15	5.96		Hexacosene
42	32.145	31.995	32.370	53063041	2.86	8303389	4.76	6.39	V	Hexacosane
43	33.255	33.100	33.430	15089889	0.81	2284400	1.31	6.61		Tetracosanal
44	35.835	35.590	35.955	47998588	2.59	4587397	2.63	10.46		Tetracosanol
45	36.417	35.955	37.430	682500371	36.77	36039867	20.68	18.94	SV	Heptacosane
46	41.175	40.930	41.680	52235727	2.81	4908685	2.82	10.64	V	Octacosane
47	42.763	42.455	42.955	18694724	1.01	1700804	0.98	10.99		Squalene
48	43.154	42.955	43.455	14327053	0.77	1320951	0.76	10.85	V	Hexacosanal
49	46.003	45.505	46.255	9758246	0.53	680241	0.39	14.35		Hexacosanol
50	48.115	47.530	48.830	486106679	26.19	21709586	12.46	22.39	V	Nonacosane
				1856269447	100.00	174278465	100.00			

Spectrum

Line#:1 R.Time:9.635(Scan#:928)
MassPeaks:202
RawMode:Averaged 9.630-9.640(927-929) BasePeak:43(7699)
BG Mode:Calc. from Peak Group 1 - Event 1

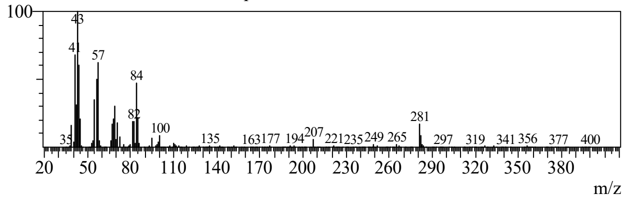

Line#:2 R.Time:11.115(Scan#:1224)
MassPeaks:194
RawMode:Averaged 11.110-11.120(1223-1225) BasePeak:57(27971)
BG Mode:Calc. from Peak Group 1 - Event 1

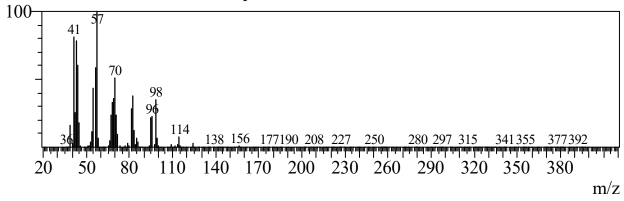

Line#:3 R.Time:11.835(Scan#:1368)
MassPeaks:214
RawMode:Averaged 11.830-11.840(1367-1369) BasePeak:43(7933)
BG Mode:Calc. from Peak Group 1 - Event 1

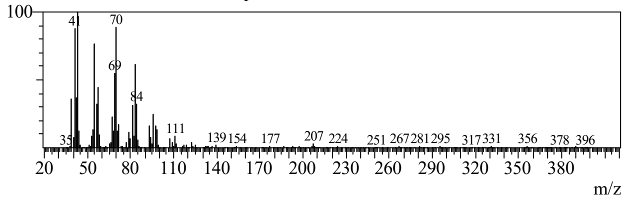

Line#:4 R.Time:11.960(Scan#:1393)
MassPeaks:186
RawMode:Averaged 11.955-11.965(1392-1394) BasePeak:41(900)
BG Mode:Calc. from Peak Group 1 - Event 1

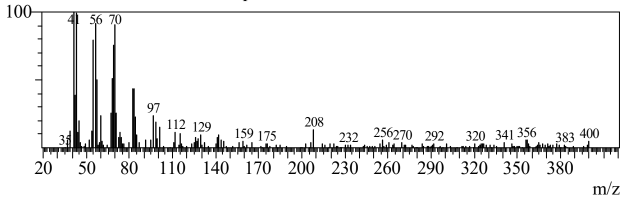

Line#:5 R.Time:12.330(Scan#:1467)
MassPeaks:149
RawMode:Averaged 12.325-12.335(1466-1468) BasePeak:57(5866)
BG Mode:Calc. from Peak Group 1 - Event 1

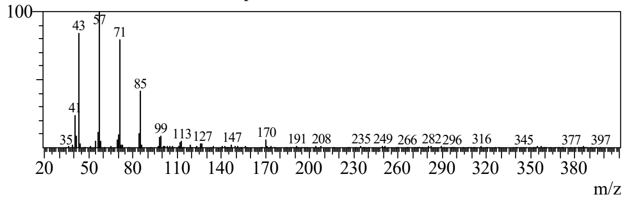

Line#:6 R.Time:12.380(Scan#:1477)
MassPeaks:193
RawMode:Averaged 12.375-12.385(1476-1478) BasePeak:43(10959)
BG Mode:Calc. from Peak Group 1 - Event 1

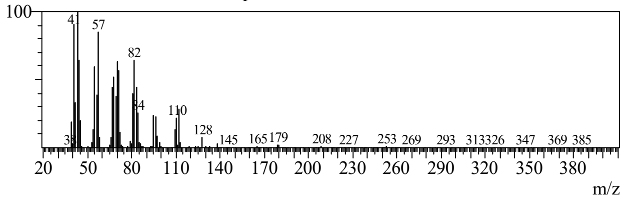

Line#:7 R.Time:13.025(Scan#:1606)
MassPeaks:202
RawMode:Averaged 13.020-13.030(1605-1607) BasePeak:43(53018)
BG Mode:Calc. from Peak Group 1 - Event 1

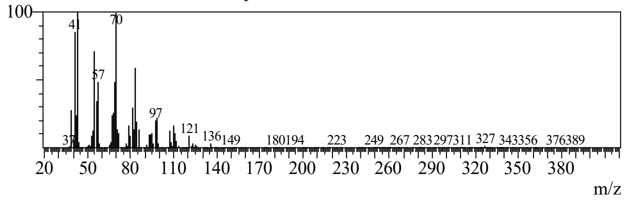

Line#:8 R.Time:13.385(Scan#:1678)
MassPeaks:186
RawMode:Averaged 13.380-13.390(1677-1679) BasePeak:81(13272)
BG Mode:Calc. from Peak Group 1 - Event 1

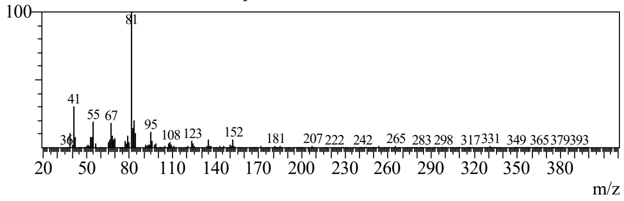

Line#:9 R.Time:13.510(Scan#:1703)
MassPeaks:216
RawMode:Averaged 13.505-13.515(1702-1704) BasePeak:281(5104)
BG Mode:Calc. from Peak Group 1 - Event 1

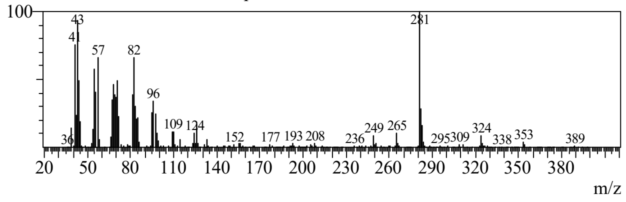

Line#:10 R.Time:14.105(Scan#:1822)
MassPeaks:225
RawMode:Averaged 14.100-14.110(1821-1823) BasePeak:70(62868)
BG Mode:Calc. from Peak Group 1 - Event 1

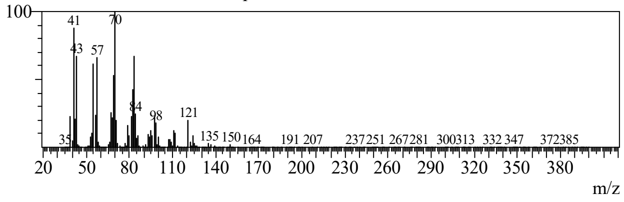

Line#:11 R.Time:14.455(Scan#:1892)
MassPeaks:195
RawMode:Averaged 14.450-14.460(1891-1893) BasePeak:57(7896)
BG Mode:Calc. from Peak Group 1 - Event 1

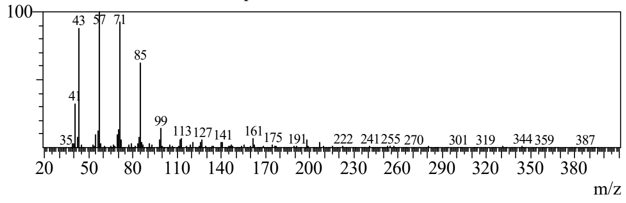

Line#:12 R.Time:14.545(Scan#:1910)
MassPeaks:237
RawMode:Averaged 14.540-14.550(1909-1911) BasePeak:43(5951)
BG Mode:Calc. from Peak Group 1 - Event 1

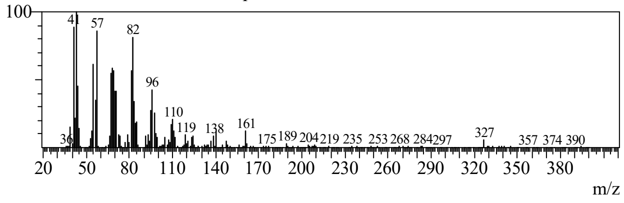

Line#:13 R.Time:14.975(Scan#:1996)
MassPeaks:221
RawMode:Averaged 14.970-14.980(1995-1997) BasePeak:43(15265)
BG Mode:Calc. from Peak Group 1 - Event 1

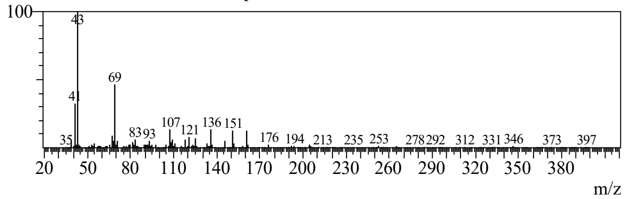

Line#:14 R.Time:15.305(Scan#:2062)
MassPeaks:180
RawMode:Averaged 15.300-15.310(2061-2063) BasePeak:69(7797)
BG Mode:Calc. from Peak Group 1 - Event 1

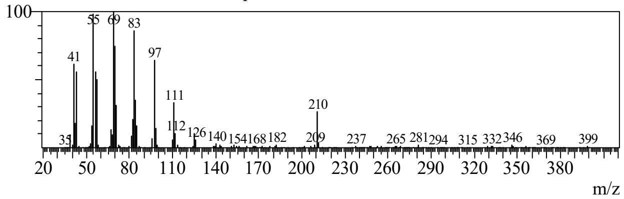

Line#:15 R.Time:15.405(Scan#:2082)
MassPeaks:204
RawMode:Averaged 15.400-15.410(2081-2083) BasePeak:71(40464)
BG Mode:Calc. from Peak Group 1 - Event 1

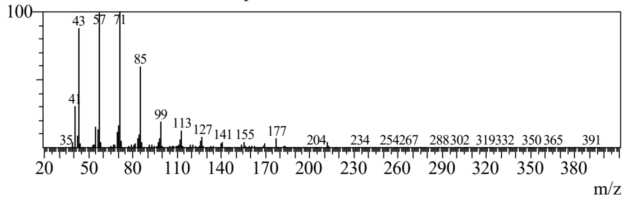

Line#:16 R.Time:15.505(Scan#:2102)
MassPeaks:190
RawMode:Averaged 15.500-15.510(2101-2103) BasePeak:43(5478)
BG Mode:Calc. from Peak Group 1 - Event 1

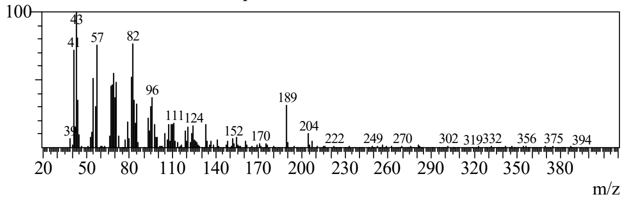

Line#:17 R.Time:16.295(Scan#:2260)
MassPeaks:228
RawMode:Averaged 16.290-16.300(2259-2261) BasePeak:57(84202)
BG Mode:Calc. from Peak Group 1 - Event 1

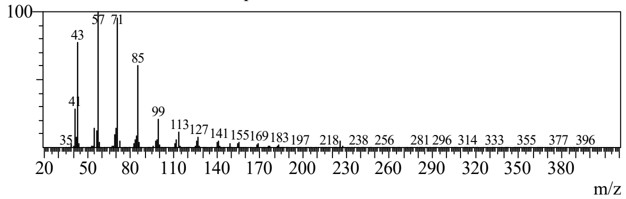

Line#:18 R.Time:16.410(Scan#:2283)
MassPeaks:180
RawMode:Averaged 16.405-16.415(2282-2284) BasePeak:82(5255)
BG Mode:Calc. from Peak Group 1 - Event 1

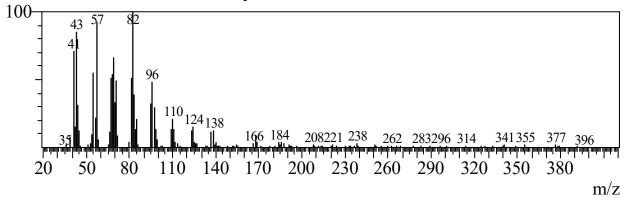

Line#:19 R.Time:17.175(Scan#:2436)
MassPeaks:183
RawMode:Averaged 17.170-17.180(2435-2437) BasePeak:71(37298)
BG Mode:Calc. from Peak Group 1 - Event 1

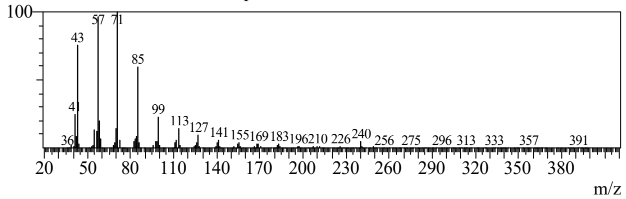

Line#:20 R.Time:17.320(Scan#:2465)
MassPeaks:219
RawMode:Averaged 17.315-17.325(2464-2466) BasePeak:57(12716)
BG Mode:Calc. from Peak Group 1 - Event 1

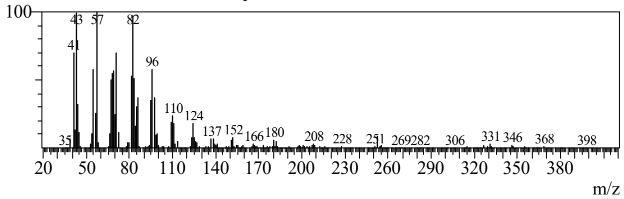

Line#:21 R.Time:18.130(Scan#:2627)
MassPeaks:202
RawMode:Averaged 18.125-18.135(2626-2628) BasePeak:71(10678)
BG Mode:Calc. from Peak Group 1 - Event 1

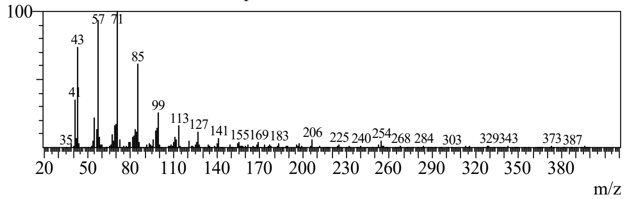

Line#:22 R.Time:18.220(Scan#:2645)
MassPeaks:243
RawMode:Averaged 18.215-18.225(2644-2646) BasePeak:55(7056)
BG Mode:Calc. from Peak Group 1 - Event 1

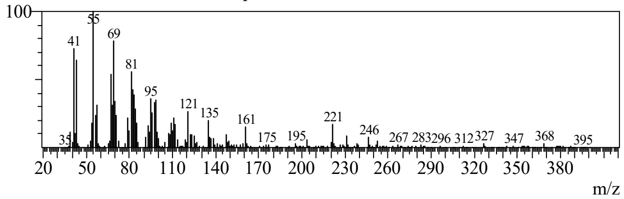

Line#:23 R.Time:19.195(Scan#:2840)
MassPeaks:213
RawMode:Averaged 19.190-19.200(2839-2841) BasePeak:57(19830)
BG Mode:Calc. from Peak Group 1 - Event 1

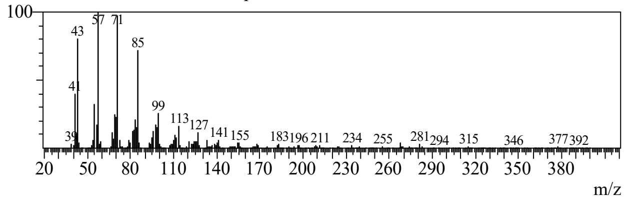

Line#:24 R.Time:19.460(Scan#:2893)
MassPeaks:231
RawMode:Averaged 19.455-19.465(2892-2894) BasePeak:74(32272)
BG Mode:Calc. from Peak Group 1 - Event 1

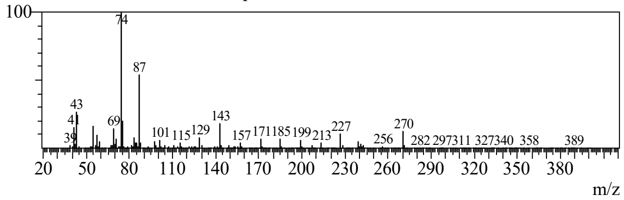

Line#:25 R.Time:19.660(Scan#:2933)
MassPeaks:289
RawMode:Averaged 19.655-19.665(2932-2934) BasePeak:69(12355)
BG Mode:Calc. from Peak Group 1 - Event 1

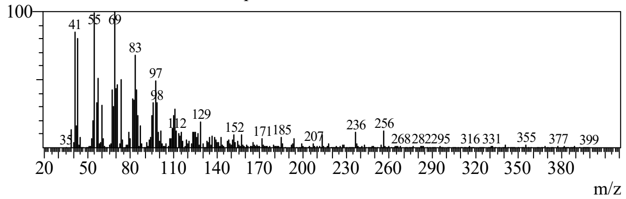

Line#:26 R.Time:19.865(Scan#:2974)
MassPeaks:302
RawMode:Averaged 19.860-19.870(2973-2975) BasePeak:73(655522)
BG Mode:Calc. from Peak Group 1 - Event 1

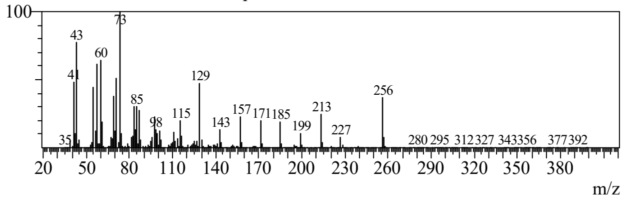

Line#:27 R.Time:20.385(Scan#:3078)
MassPeaks:228
RawMode:Averaged 20.380-20.390(3077-3079) BasePeak:71(124653)
BG Mode:Calc. from Peak Group 1 - Event 1

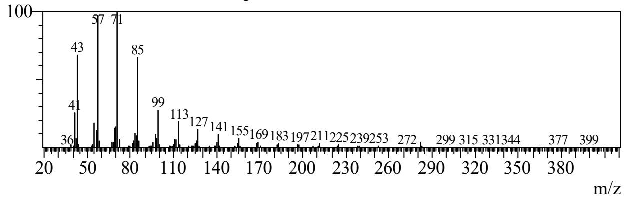

Line#:28 R.Time:20.660(Scan#:3133)
MassPeaks:244
RawMode:Averaged 20.655-20.665(3132-3134) BasePeak:43(17218)
BG Mode:Calc. from Peak Group 1 - Event 1

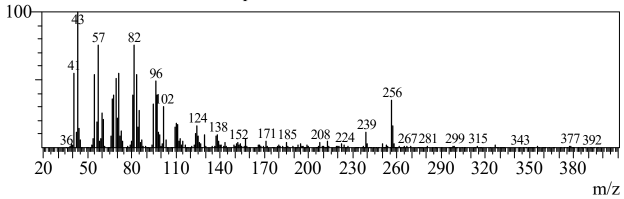

Line#:29 R.Time:21.360(Scan#:3273)
MassPeaks:217
RawMode:Averaged 21.355-21.365(3272-3274) BasePeak:81(106163)
BG Mode:Calc. from Peak Group 1 - Event 1

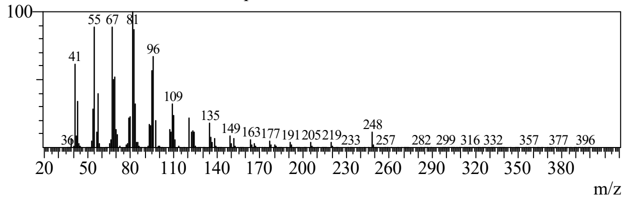

Line#:30 R.Time:21.495(Scan#:3300)
MassPeaks:186
RawMode:Averaged 21.490-21.500(3299-3301) BasePeak:83(16712)
BG Mode:Calc. from Peak Group 1 - Event 1

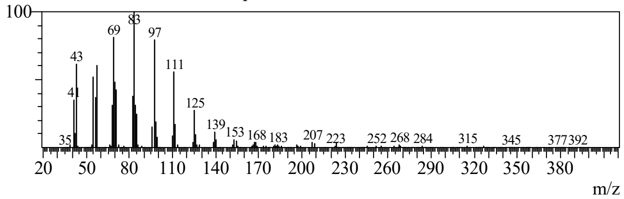

Line#:31 R.Time:21.705(Scan#:3342)
MassPeaks:256
RawMode:Averaged 21.700-21.710(3341-3343) BasePeak:71(132802)
BG Mode:Calc. from Peak Group 1 - Event 1

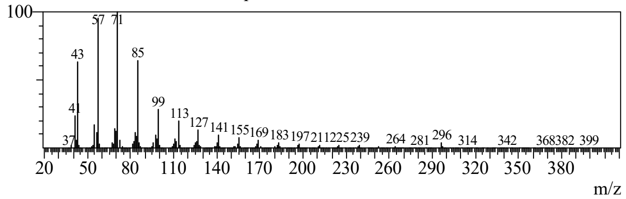

Line#:32 R.Time:22.225(Scan#:3446)
MassPeaks:290
RawMode:Averaged 22.220-22.230(3445-3447) BasePeak:55(23828)
BG Mode:Calc. from Peak Group 1 - Event 1

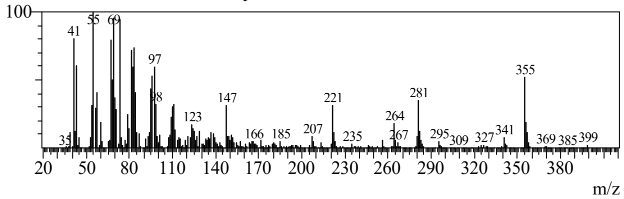

Line#:33 R.Time:23.150(Scan#:3631)
MassPeaks:212
RawMode:Averaged 23.145-23.155(3630-3632) BasePeak:71(183587)
BG Mode:Calc. from Peak Group 1 - Event 1

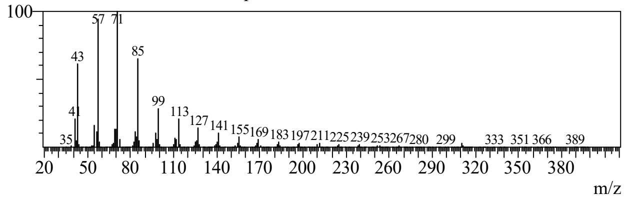

Line#:34 R.Time:23.540(Scan#:3709)
MassPeaks:240
RawMode:Averaged 23.535-23.545(3708-3710) BasePeak:82(13515)
BG Mode:Calc. from Peak Group 1 - Event 1

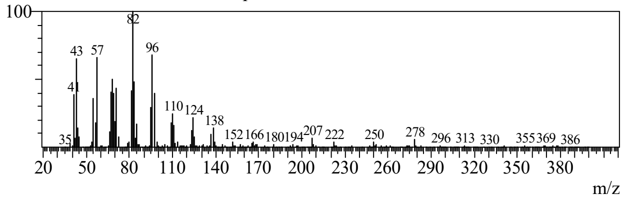

Line#:35 R.Time:24.770(Scan#:3955)
MassPeaks:147
RawMode:Averaged 24.765-24.775(3954-3956) BasePeak:57(4774642)
BG Mode:Calc. from Peak Group 1 - Event 1

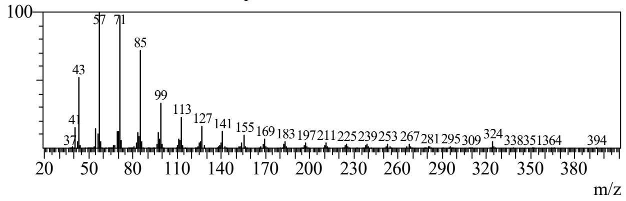

Line#:36 R.Time:26.660(Scan#:4333)
MassPeaks:230
RawMode:Averaged 26.655-26.665(4332-4334) BasePeak:71(501922)
BG Mode:Calc. from Peak Group 1 - Event 1

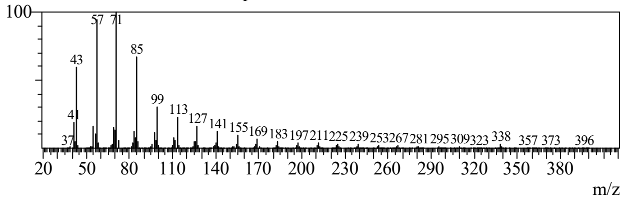

Line#:37 R.Time:27.300(Scan#:4461)
MassPeaks:207
RawMode:Averaged 27.295-27.305(4460-4462) BasePeak:82(8294)
BG Mode:Calc. from Peak Group 1 - Event 1

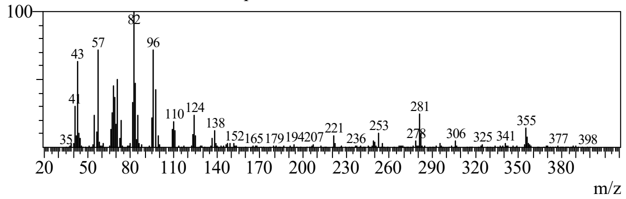

Line#:38 R.Time:28.820(Scan#:4765)
MassPeaks:216
RawMode:Averaged 28.815-28.825(4764-4766) BasePeak:83(50811)
BG Mode:Calc. from Peak Group 1 - Event 1

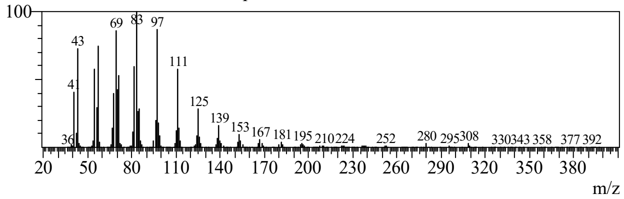

Line#:39 R.Time:28.890(Scan#:4779)
MassPeaks:216
RawMode:Averaged 28.885-28.895(4778-4780) BasePeak:97(26305)
BG Mode:Calc. from Peak Group 1 - Event 1

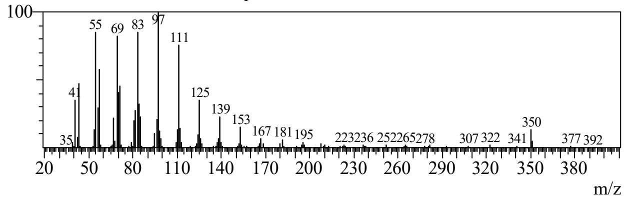

Line#:40 R.Time:29.170(Scan#:4835)
MassPeaks:183
RawMode:Averaged 29.165-29.175(4834-4836) BasePeak:57(4814672)
BG Mode:Calc. from Peak Group 1 - Event 1

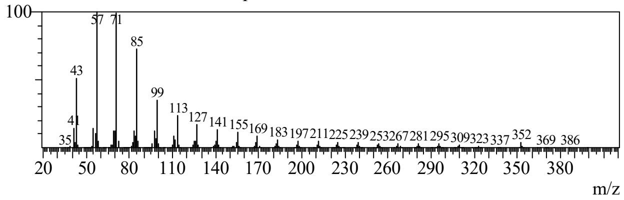

Line#:41 R.Time:31.920(Scan#:5385)
MassPeaks:220
RawMode:Averaged 31.915-31.925(5384-5386) BasePeak:97(19084)
BG Mode:Calc. from Peak Group 1 - Event 1

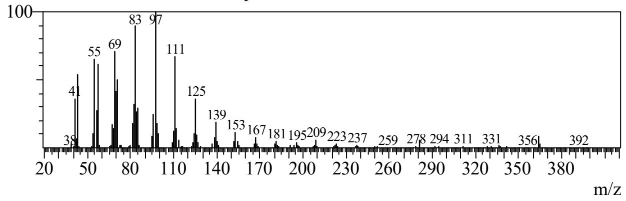

Line#:42 R.Time:32.145(Scan#:5430)
MassPeaks:203
RawMode:Averaged 32.140-32.150(5429-5431) BasePeak:71(1195440)
BG Mode:Calc. from Peak Group 1 - Event 1

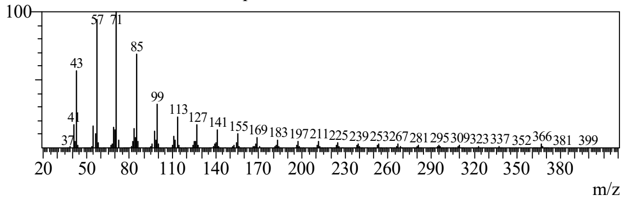

Line#:43 R.Time:33.255(Scan#:5652)
MassPeaks:273
RawMode:Averaged 33.250-33.260(5651-5653) BasePeak:82(206119)
BG Mode:Calc. from Peak Group 1 - Event 1

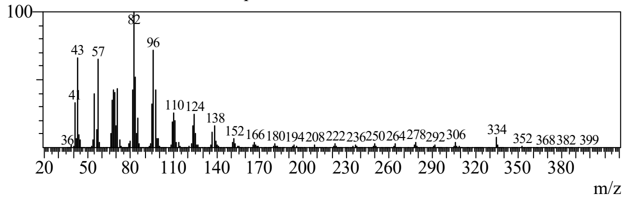

Line#:44 R.Time:35.835(Scan#:6168)
MassPeaks:189
RawMode:Averaged 35.830-35.840(6167-6169) BasePeak:97(269642)
BG Mode:Calc. from Peak Group 1 - Event 1

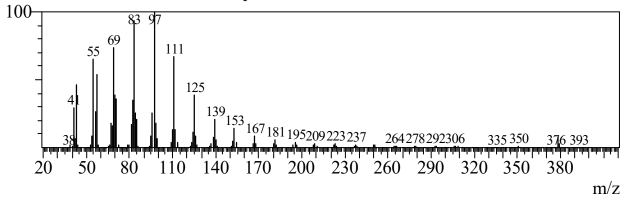

Line#:45 R.Time:36.415(Scan#:6284)
MassPeaks:192
RawMode:Averaged 36.410-36.420(6283-6285) BasePeak:57(4878129)
BG Mode:Calc. from Peak Group 1 - Event 1

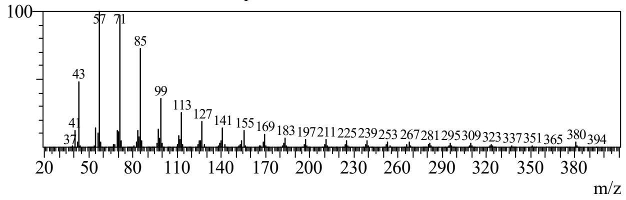

Line#:46 R.Time:41.175(Scan#:7236)
MassPeaks:243
RawMode:Averaged 41.170-41.180(7235-7237) BasePeak:71(679745)
BG Mode:Calc. from Peak Group 1 - Event 1

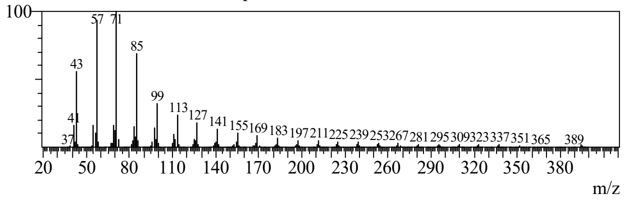

Line#:47 R.Time:42.765(Scan#:7554)
MassPeaks:278
RawMode:Averaged 42.760-42.770(7553-7555) BasePeak:69(386687)
BG Mode:Calc. from Peak Group 1 - Event 1

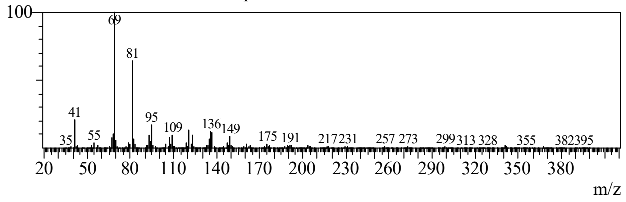

Line#:48 R.Time:43.155(Scan#:7632)
MassPeaks:260
RawMode:Averaged 43.150-43.160(7631-7633) BasePeak:82(114413)
BG Mode:Calc. from Peak Group 1 - Event 1

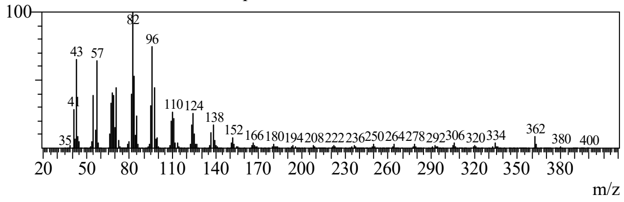

Line#:49 R.Time:46.005(Scan#:8202)
MassPeaks:230
RawMode:Averaged 46.000-46.010(8201-8203) BasePeak:97(55665)
BG Mode:Calc. from Peak Group 1 - Event 1

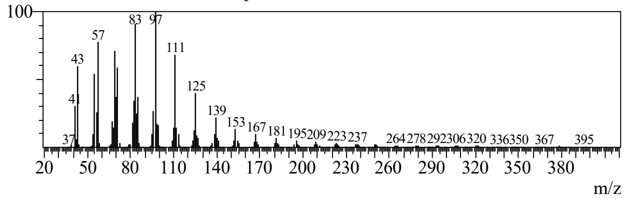

Line#:50 R.Time:48.115(Scan#:8624)
MassPeaks:228
RawMode:Averaged 48.110-48.120(8623-8625) BasePeak:71(2910501)
BG Mode:Calc. from Peak Group 1 - Event 1

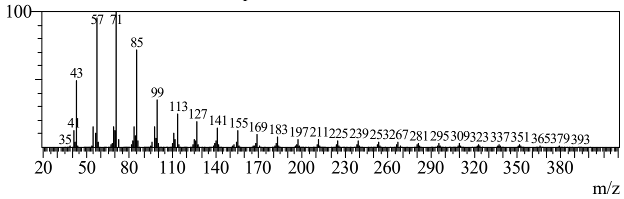

Male thorax extract

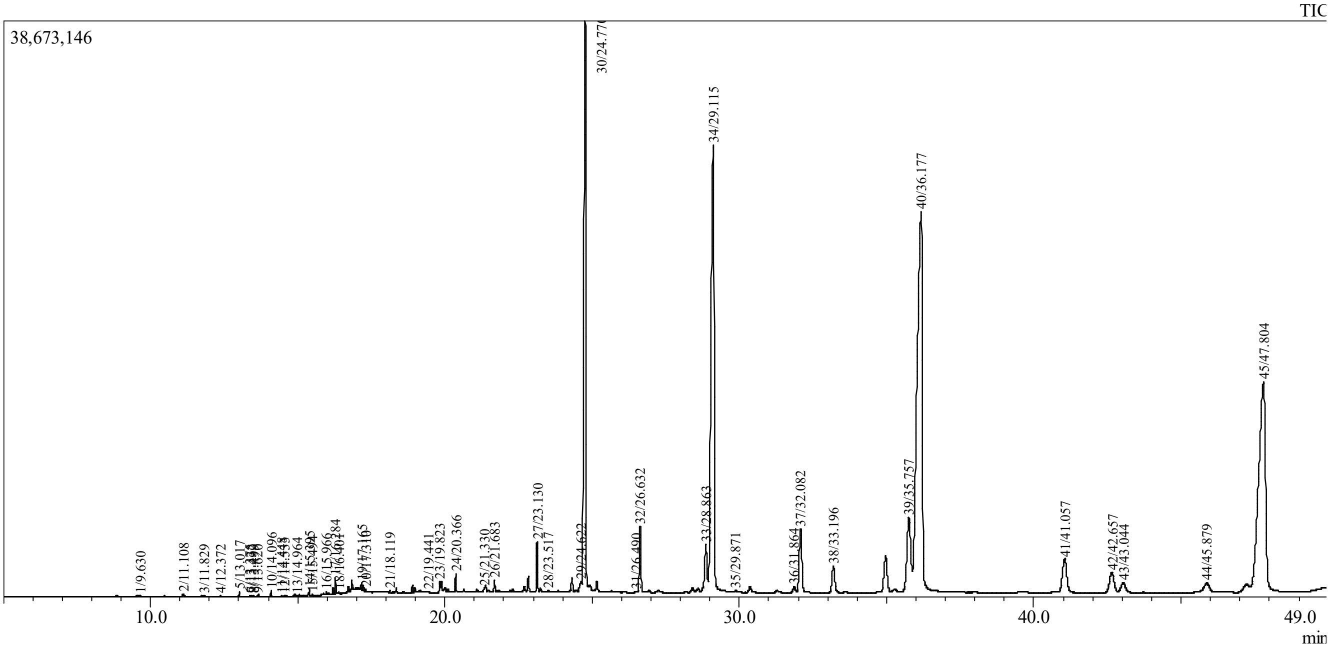

Peak Report TIC

Peak#	R.Time	I.Time	F.Time	Area	Area%	Height	Height%	A/H	Mark	Name
1	9.630	9.555	9.700	219332	0.02	58470	0.04	3.75	MI	Octanal
2	11.108	11.070	11.165	299240	0.03	136279	0.10	2.20		Nonanal
3	11.829	11.800	11.865	70165	0.01	40199	0.03	1.75		2-Nonenal
4	12.372	12.345	12.410	148780	0.01	98833	0.07	1.51		Decanal
5	13.017	12.980	13.055	425435	0.04	283342	0.20	1.50		2-Decenal
6	13.375	13.325	13.410	66083	0.01	29217	0.02	2.26	MI	2,4-Decadienal
7	13.432	13.410	13.465	82354	0.01	63158	0.04	1.30		Tridecane
8	13.499	13.470	13.530	73702	0.01	47722	0.03	1.54		Undecanal
9	13.620	13.530	13.635	103836	0.01	68418	0.05	1.52	V	Decadienal
10	14.096	14.060	14.145	545977	0.05	371823	0.26	1.47		Undecenal
11	14.448	14.405	14.475	59786	0.01	39042	0.03	1.53	MI	Tetradecane
12	14.533	14.505	14.565	85798	0.01	58635	0.04	1.46		Dodecanal
13	14.964	14.935	14.990	72613	0.01	46290	0.03	1.57	V	Geranyl acetone
14	15.395	15.370	15.430	601483	0.05	422481	0.30	1.42	V	Pentadecane
15	15.494	15.465	15.525	82676	0.01	56055	0.04	1.47		Tridecanal
16	15.966	15.945	15.990	247339	0.02	167090	0.12	1.48	V	2-metil-pentadecane
17	16.284	16.245	16.315	1602833	0.14	1104925	0.77	1.45		Hexadecane
18	16.401	16.315	16.435	281569	0.02	81480	0.06	3.46	V	Tetradecanal
19	17.165	17.140	17.205	1360343	0.12	634129	0.44	2.15	V	Heptadecane
20	17.310	17.275	17.350	448189	0.04	207124	0.14	2.16	V	Pentadecanal
21	18.119	18.085	18.155	212002	0.02	121389	0.08	1.75		Octadecane
22	19.441	19.380	19.485	264985	0.02	86602	0.06	3.06		Methyl hexadecanoate
23	19.823	19.765	19.855	1724457	0.15	726254	0.51	2.37		Hexadecanoic acid
24	20.366	20.310	20.415	2568839	0.22	1188603	0.83	2.16		Eicosane
25	21.330	21.280	21.350	630340	0.05	247116	0.17	2.55		(Z,E)-2,13-Octadecadienol
26	21.683	21.625	21.735	1915369	0.17	775301	0.54	2.47		Heneicosane
27	23.130	23.065	23.195	9148663	0.79	3398671	2.38	2.69		Docosane
28	23.517	23.475	23.565	173383	0.01	64410	0.05	2.69		Eicosanal
29	24.622	24.520	24.650	3107932	0.27	633575	0.44	4.91		Eicosanol
30	24.770	24.650	24.875	184486053	15.95	38233006	26.76	4.83	V	Tricosane
31	26.490	26.440	26.540	252889	0.02	71979	0.05	3.51		Heneicosanol
32	26.632	26.540	26.750	16897358	1.46	4401686	3.08	3.84	V	Tetracosane
33	28.863	28.680	28.940	18221127	1.58	3186072	2.23	5.72	V	Docosanol
34	29.115	28.940	29.415	217554655	18.81	29974545	20.98	7.26	V	Pentacosane
35	29.871	29.755	29.965	496732	0.04	107051	0.07	4.64		Tricosanal
36	31.864	31.725	31.945	2213013	0.19	377153	0.26	5.87		Tricosanol
37	32.082	31.945	32.305	26679958	2.31	4261148	2.98	6.26	V	Hexacosane
38	33.196	33.015	33.405	11347200	0.98	1743532	1.22	6.51		Tetracosanal
39	35.757	35.445	35.875	46582680	4.03	5044747	3.53	9.23	V	Tetracosanol
40	36.177	35.875	37.050	318231275	27.52	25487717	17.84	12.49	V	Heptacosane
41	41.057	40.850	41.425	23114441	2.00	2231676	1.56	10.36	V	Octacosane
42	42.657	42.325	42.850	14785482	1.28	1340889	0.94	11.03		Squalene
43	43.044	42.850	43.400	7096178	0.61	632632	0.44	11.22	V	Hexacosanal
44	45.879	45.400	46.175	8071886	0.70	585551	0.41	13.79		Hexacosanol
45	47.804	47.350	48.575	233642840	20.21	13949017	9.76	16.75	V	Nonacosane
				1156297270	100.00	142885034	100.00			

Spectrum

Line#:1 R.Time:9.630(Scan#:927)
MassPeaks:226
RawMode:Averaged 9.625-9.635(926-928) BasePeak:281(5507)
BG Mode:Calc. from Peak Group 1 - Event 1

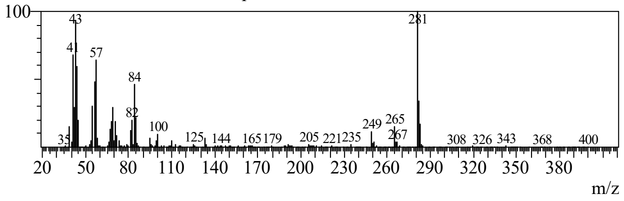

Line#:2 R.Time:11.110(Scan#:1223)
MassPeaks:201
RawMode:Averaged 11.105-11.115(1222-1224) BasePeak:57(14383)
BG Mode:Calc. from Peak Group 1 - Event 1

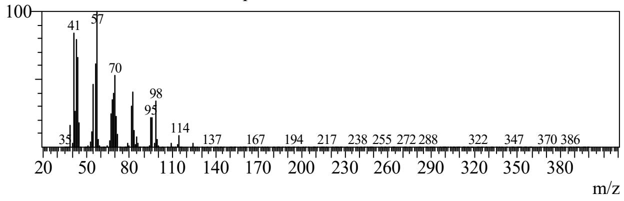

Line#:3 R.Time:11.830(Scan#:1367)
MassPeaks:202
RawMode:Averaged 11.825-11.835(1366-1368) BasePeak:43(4092)
BG Mode:Calc. from Peak Group 1 - Event 1

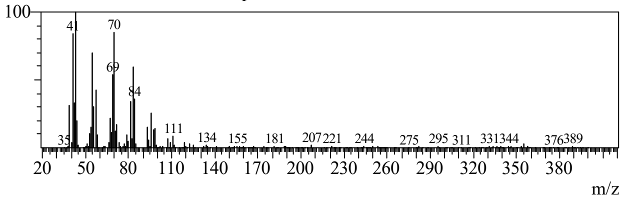

Line#:4 R.Time:12.370(Scan#:1475)
MassPeaks:201
RawMode:Averaged 12.365-12.375(1474-1476) BasePeak:43(7826)
BG Mode:Calc. from Peak Group 1 - Event 1

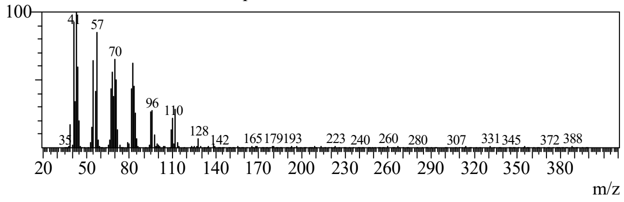

Line#:5 R.Time:13.015(Scan#:1604)
MassPeaks:193
RawMode:Averaged 13.010-13.020(1603-1605) BasePeak:70(27946)
BG Mode:Calc. from Peak Group 1 - Event 1

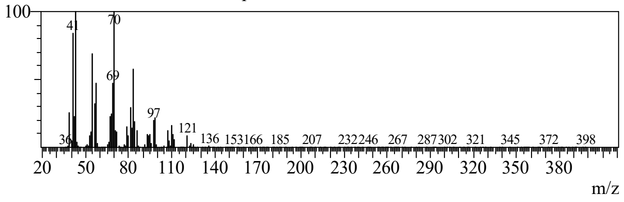

Line#:6 R.Time:13.375(Scan#:1676)
MassPeaks:178
RawMode:Averaged 13.370-13.380(1675-1677) BasePeak:81(7764)
BG Mode:Calc. from Peak Group 1 - Event 1

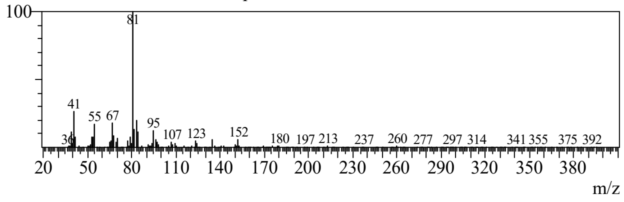

Line#:7 R.Time:13.430(Scan#:1687)
MassPeaks:209
RawMode:Averaged 13.425-13.435(1686-1688) BasePeak:57(10513)
BG Mode:Calc. from Peak Group 1 - Event 1

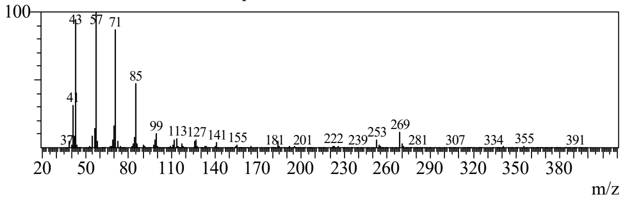

Line#:8 R.Time:13.500(Scan#:1701)
MassPeaks:221
RawMode:Averaged 13.495-13.505(1700-1702) BasePeak:43(3802)
BG Mode:Calc. from Peak Group 1 - Event 1

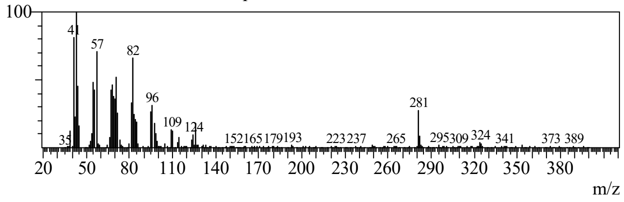

Line#:9 R.Time:13.620(Scan#:1725)
MassPeaks:164
RawMode:Averaged 13.615-13.625(1724-1726) BasePeak:81(16643)
BG Mode:Calc. from Peak Group 1 - Event 1

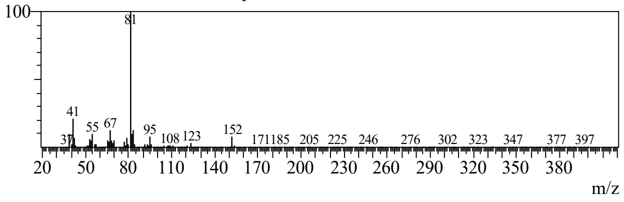

Line#:10 R.Time:14.095(Scan#:1820)
MassPeaks:223
RawMode:Averaged 14.090-14.100(1819-1821) BasePeak:70(34718)
BG Mode:Calc. from Peak Group 1 - Event 1

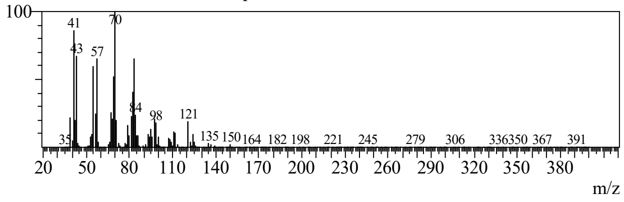

Line#:11 R.Time:14.450(Scan#:1891)
MassPeaks:194
RawMode:Averaged 14.445-14.455(1890-1892) BasePeak:57(6339)
BG Mode:Calc. from Peak Group 1 - Event 1

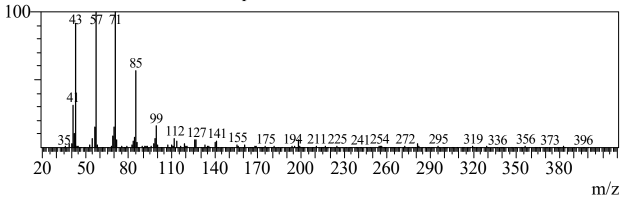

Line#:12 R.Time:14.535(Scan#:1908)
MassPeaks:207
RawMode:Averaged 14.530-14.540(1907-1909) BasePeak:43(4064)
BG Mode:Calc. from Peak Group 1 - Event 1

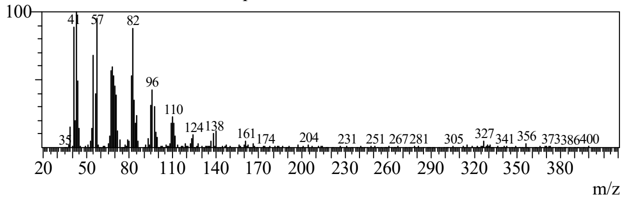

Line#:13 R.Time:14.965(Scan#:1994)
MassPeaks:234
RawMode:Averaged 14.960-14.970(1993-1995) BasePeak:43(11427)
BG Mode:Calc. from Peak Group 1 - Event 1

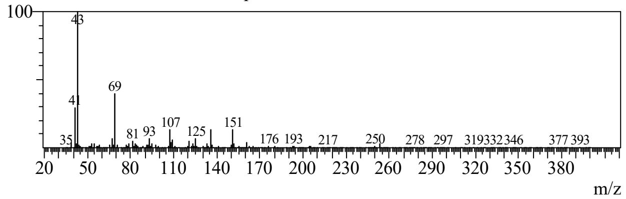

Line#:14 R.Time:15.395(Scan#:2080)
MassPeaks:199
RawMode:Averaged 15.390-15.400(2079-2081) BasePeak:57(67269)
BG Mode:Calc. from Peak Group 1 - Event 1

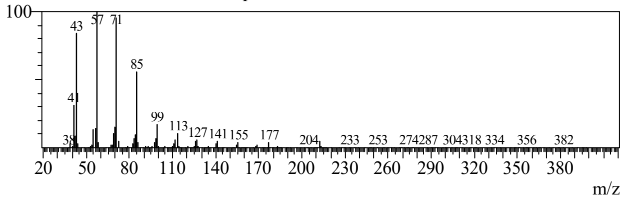

Line#:15 R.Time:15.495(Scan#:2100)
MassPeaks:209
RawMode:Averaged 15.490-15.500(2099-2101) BasePeak:43(4995)
BG Mode:Calc. from Peak Group 1 - Event 1

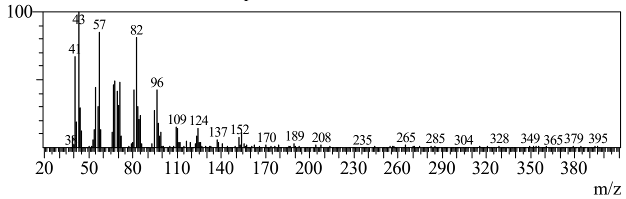

Line#:16 R.Time:15.965(Scan#:2194)
MassPeaks:218
RawMode:Averaged 15.960-15.970(2193-2195) BasePeak:57(18473)
BG Mode:Calc. from Peak Group 1 - Event 1

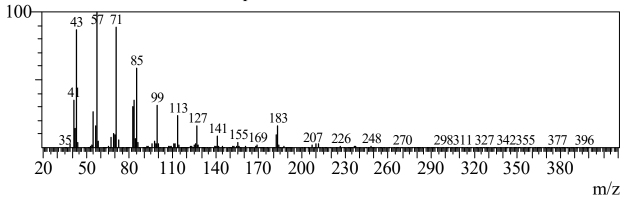

Line#:17 R.Time:16.285(Scan#:2258)
MassPeaks:196
RawMode:Averaged 16.280-16.290(2257-2259) BasePeak:57(179371)
BG Mode:Calc. from Peak Group 1 - Event 1

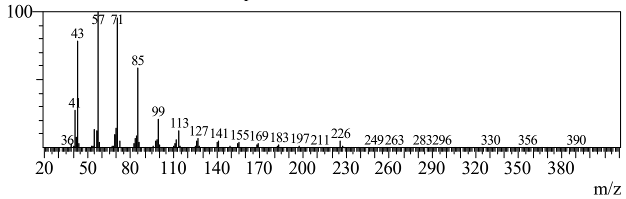

Line#:18 R.Time:16.400(Scan#:2281)
MassPeaks:206
RawMode:Averaged 16.395-16.405(2280-2282) BasePeak:57(6022)
BG Mode:Calc. from Peak Group 1 - Event 1

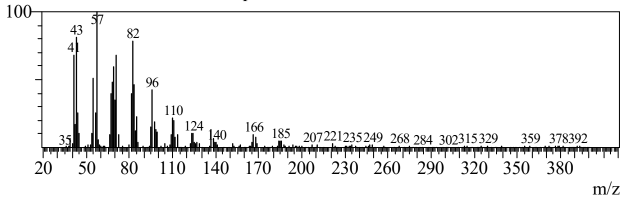

Line#:19 R.Time:17.165(Scan#:2434)
MassPeaks:189
RawMode:Averaged 17.160-17.170(2433-2435) BasePeak:57(71904)
BG Mode:Calc. from Peak Group 1 - Event 1

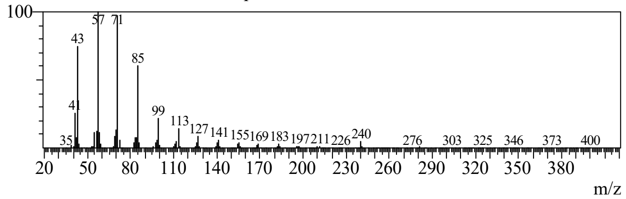

Line#:20 R.Time:17.310(Scan#:2463)
MassPeaks:216
RawMode:Averaged 17.305-17.315(2462-2464) BasePeak:43(13356)
BG Mode:Calc. from Peak Group 1 - Event 1

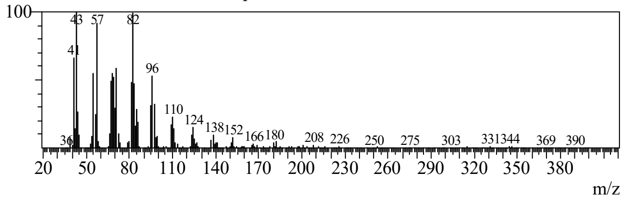

Line#:21 R.Time:18.120(Scan#:2625)
MassPeaks:218
RawMode:Averaged 18.115-18.125(2624-2626) BasePeak:71(17251)
BG Mode:Calc. from Peak Group 1 - Event 1

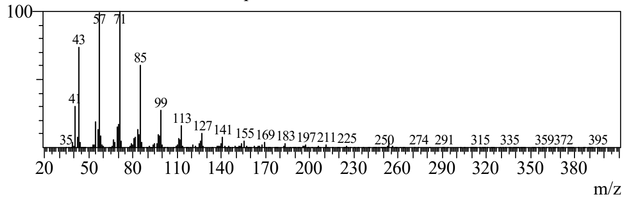

Line#:22 R.Time:19.440(Scan#:2889)
MassPeaks:219
RawMode:Averaged 19.435-19.445(2888-2890) BasePeak:74(18484)
BG Mode:Calc. from Peak Group 1 - Event 1

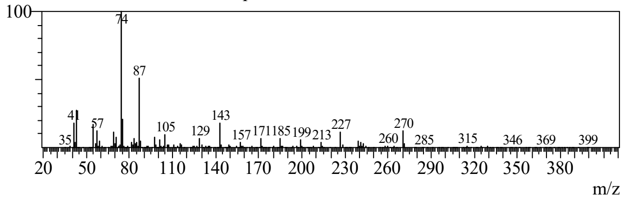

Line#:23 R.Time:19.825(Scan#:2966)
MassPeaks:263
RawMode:Averaged 19.820-19.830(2965-2967) BasePeak:73(44399)
BG Mode:Calc. from Peak Group 1 - Event 1

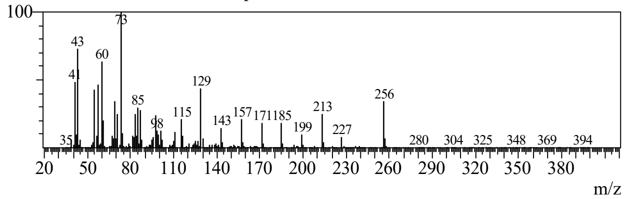

Line#:24 R.Time:20.365(Scan#:3074)
MassPeaks:219
RawMode:Averaged 20.360-20.370(3073-3075) BasePeak:71(184410)
BG Mode:Calc. from Peak Group 1 - Event 1

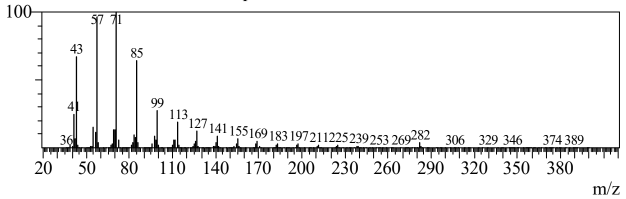

Line#:25 R.Time:21.330(Scan#:3267)
MassPeaks:212
RawMode:Averaged 21.325-21.335(3266-3268) BasePeak:81(5981)
BG Mode:Calc. from Peak Group 1 - Event 1

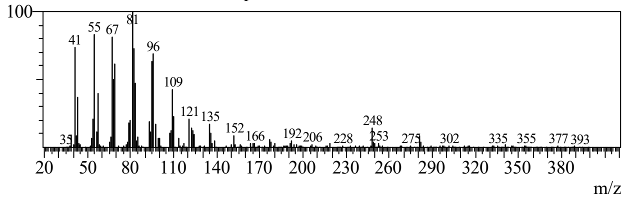

Line#:26 R.Time:21.685(Scan#:3338)
MassPeaks:224
RawMode:Averaged 21.680-21.690(3337-3339) BasePeak:71(118486)
BG Mode:Calc. from Peak Group 1 - Event 1

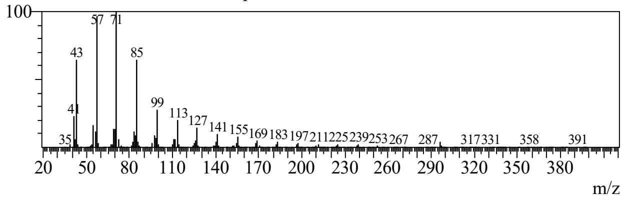

Line#:27 R.Time:23.130(Scan#:3627)
MassPeaks:197
RawMode:Averaged 23.125-23.135(3626-3628) BasePeak:71(517878)
BG Mode:Calc. from Peak Group 1 - Event 1

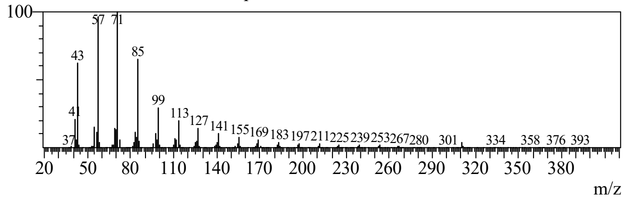

Line#:28 R.Time:23.515(Scan#:3704)
MassPeaks:219
RawMode:Averaged 23.510-23.520(3703-3705) BasePeak:82(6159)
BG Mode:Calc. from Peak Group 1 - Event 1

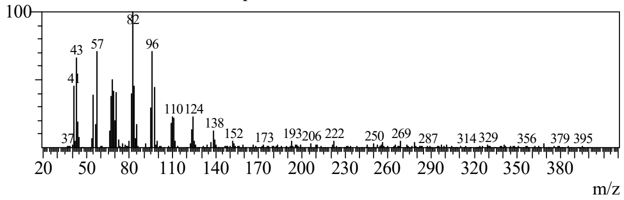

Line#:29 R.Time:24.620(Scan#:3925)
MassPeaks:214
RawMode:Averaged 24.615-24.625(3924-3926) BasePeak:83(26659)
BG Mode:Calc. from Peak Group 1 - Event 1

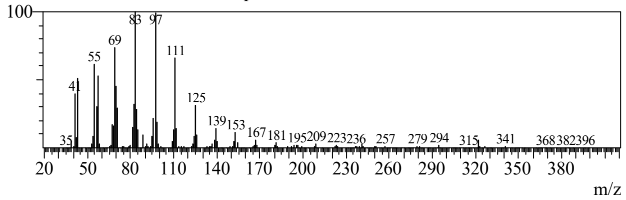

Line#:30 R.Time:24.770(Scan#:3955)
MassPeaks:166
RawMode:Averaged 24.765-24.775(3954-3956) BasePeak:57(5887093)
BG Mode:Calc. from Peak Group 1 - Event 1

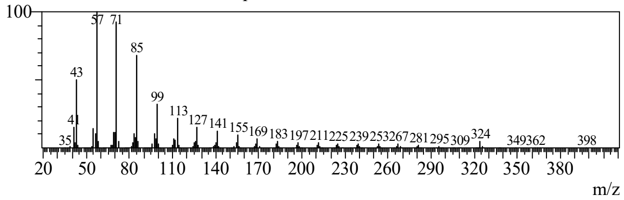

Line#:31 R.Time:26.490(Scan#:4299)
MassPeaks:234
RawMode:Averaged 26.485-26.495(4298-4300) BasePeak:97(6095)
BG Mode:Calc. from Peak Group 1 - Event 1

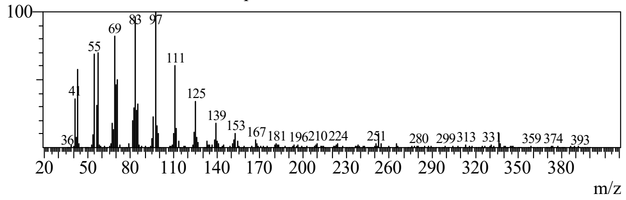

Line#:32 R.Time:26.630(Scan#:4327)
MassPeaks:239
RawMode:Averaged 26.625-26.635(4326-4328) BasePeak:71(661388)
BG Mode:Calc. from Peak Group 1 - Event 1

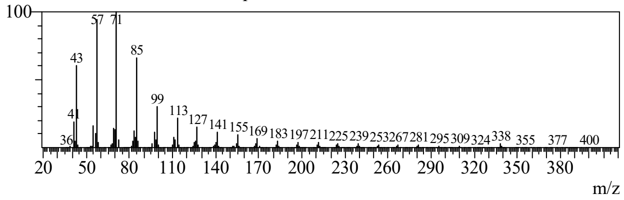

Line#:33 R.Time:28.865(Scan#:4774)
MassPeaks:237
RawMode:Averaged 28.860-28.870(4773-4775) BasePeak:97(229924)
BG Mode:Calc. from Peak Group 1 - Event 1

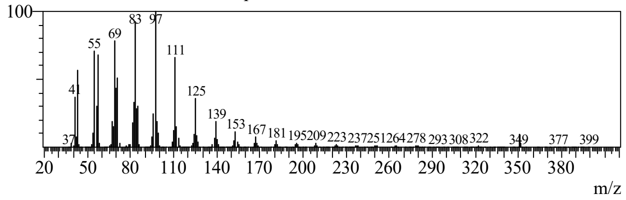

Line#:34 R.Time:29.115(Scan#:4824)
MassPeaks:206
RawMode:Averaged 29.110-29.120(4823-4825) BasePeak:57(4308614)
BG Mode:Calc. from Peak Group 1 - Event 1

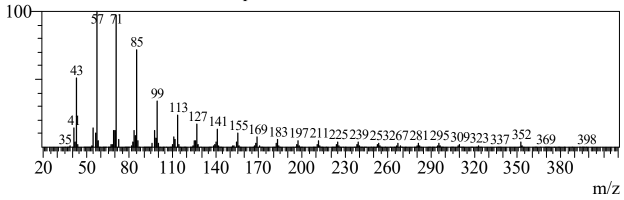

Line#:35 R.Time:29.870(Scan#:4975)
MassPeaks:221
RawMode:Averaged 29.865-29.875(4974-4976) BasePeak:82(10620)
BG Mode:Calc. from Peak Group 1 - Event 1

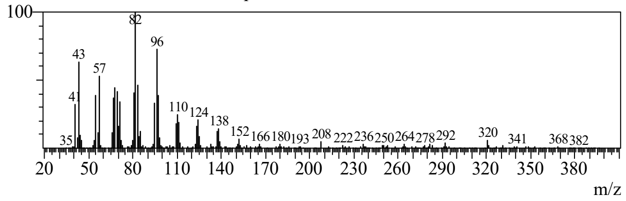

Line#:36 R.Time:31.865(Scan#:5374)
MassPeaks:234
RawMode:Averaged 31.860-31.870(5373-5375) BasePeak:97(27960)
BG Mode:Calc. from Peak Group 1 - Event 1

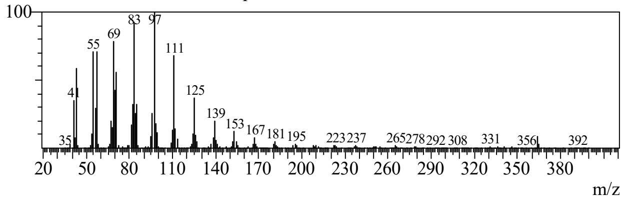

Line#:37 R.Time:32.080(Scan#:5417)
MassPeaks:235
RawMode:Averaged 32.075-32.085(5416-5418) BasePeak:71(621276)
BG Mode:Calc. from Peak Group 1 - Event 1

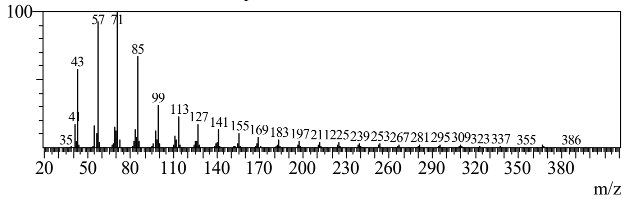

Line#:38 R.Time:33.195(Scan#:5640)
MassPeaks:261
RawMode:Averaged 33.190-33.200(5639-5641) BasePeak:82(156995)
BG Mode:Calc. from Peak Group 1 - Event 1

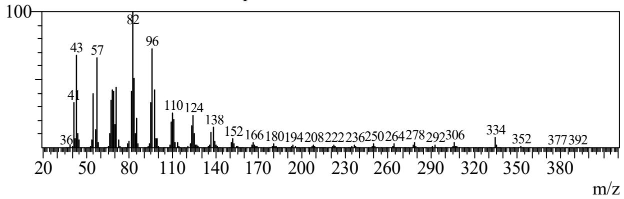

Line#:39 R.Time:35.755(Scan#:6152)
MassPeaks:244
RawMode:Averaged 35.750-35.760(6151-6153) BasePeak:97(351415)
BG Mode:Calc. from Peak Group 1 - Event 1

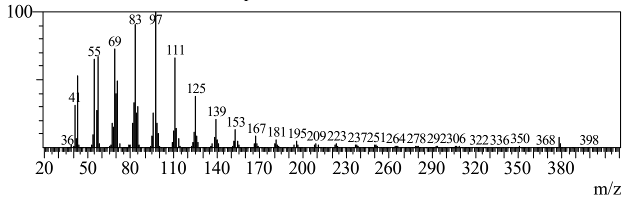

Line#:40 R.Time:36.175(Scan#:6236)
MassPeaks:209
RawMode:Averaged 36.170-36.180(6235-6237) BasePeak:71(3450511)
BG Mode:Calc. from Peak Group 1 - Event 1

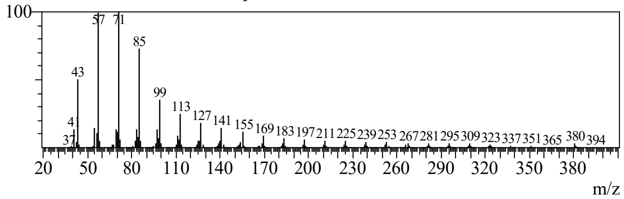

Line#:41 R.Time:41.055(Scan#:7212)
MassPeaks:259
RawMode:Averaged 41.050-41.060(7211-7213) BasePeak:71(311736)
BG Mode:Calc. from Peak Group 1 - Event 1

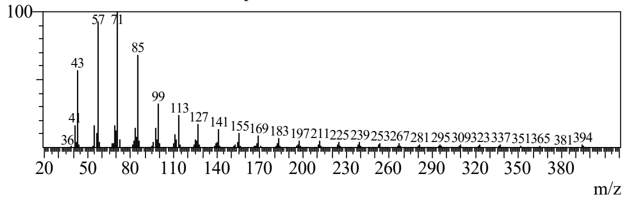

Line#:42 R.Time:42.655(Scan#:7532)
MassPeaks:287
RawMode:Averaged 42.650-42.660(7531-7533) BasePeak:69(296957)
BG Mode:Calc. from Peak Group 1 - Event 1

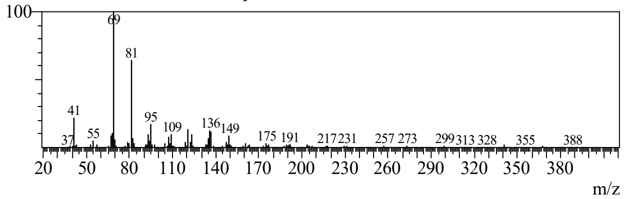

Line#:43 R.Time:43.045(Scan#:7610)
MassPeaks:259
RawMode:Averaged 43.040-43.050(7609-7611) BasePeak:82(53065)
BG Mode:Calc. from Peak Group 1 - Event 1

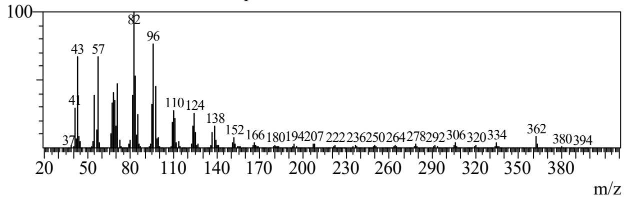

Line#:44 R.Time:45.880(Scan#:8177)
MassPeaks:240
RawMode:Averaged 45.875-45.885(8176-8178) BasePeak:97(49327)
BG Mode:Calc. from Peak Group 1 - Event 1

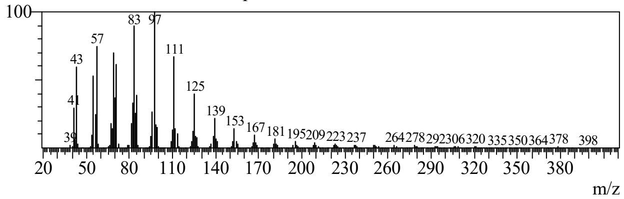

Line#:45 R.Time:47.805(Scan#:8562)
MassPeaks:240
RawMode:Averaged 47.800-47.810(8561-8563) BasePeak:71(1907941)
BG Mode:Calc. from Peak Group 1 - Event 1

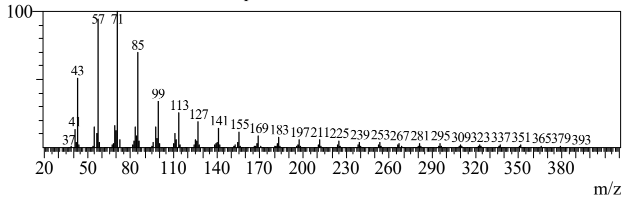

Male hindwings extract

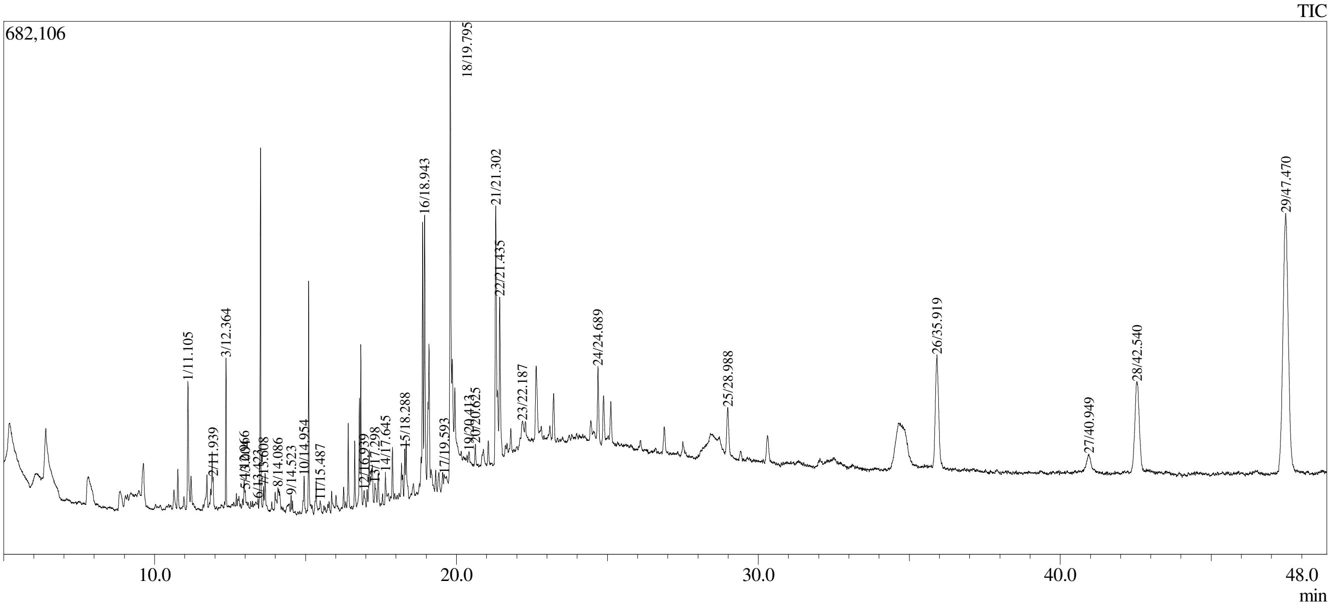

Peak Report TIC

Peak#	R.Time	I.Time	F.Time	Area	Area%	Height	Height%	A/H	Mark	Name
1	11.105	11.040	11.175	412830	3.16	164139	5.19	2.52		Nonanal
2	11.939	11.925	12.000	78722	0.60	42531	1.34	1.85	V	Nonanol
3	12.364	12.335	12.405	293747	2.25	191464	6.05	1.53	V	Decanal
4	12.966	12.945	12.990	68365	0.52	36037	1.14	1.90	V	Nonanoic acid
5	13.004	12.990	13.030	40523	0.31	23883	0.76	1.70	V	2-Decenal
6	13.423	13.405	13.445	21271	0.16	13700	0.43	1.55	V	Tridecane
7	13.608	13.565	13.630	57828	0.44	30362	0.96	1.90	V	Decadienal
8	14.086	14.060	14.100	57833	0.44	31478	1.00	1.84	V	Undecenal
9	14.523	14.495	14.545	37013	0.28	23012	0.73	1.61	V	Dodecanal
10	14.954	14.930	14.980	58453	0.45	39980	1.26	1.46	MI	Geranyl acetone
11	15.487	15.410	15.570	69617	0.53	17240	0.55	4.04	V	Tridecanal
12	16.939	16.895	17.005	75282	0.58	22252	0.70	3.38	V	Tetradecanol
13	17.298	17.260	17.365	106597	0.81	29763	0.94	3.58	V	Pentadecanal
14	17.645	17.580	17.700	104318	0.80	41734	1.32	2.50	V	Tetradecanoic acid
15	18.288	18.250	18.310	133998	1.02	65888	2.08	2.03	V	Hexadecanal
16	18.943	18.910	19.010	924480	7.07	359395	11.36	2.57	V	Hexadecanol
17	19.593	19.570	19.620	48860	0.37	19991	0.63	2.44	V	(Z)-9-Hexadecenoic acid
18	19.795	19.730	19.840	1423940	10.89	594540	18.80	2.40	V	Hexadecanoic acid
19	20.413	20.375	20.460	104950	0.80	28224	0.89	3.72	V	Hexadecanyl acetate
20	20.625	20.575	20.665	88202	0.67	32197	1.02	2.74	V	Octadecanal
21	21.302	21.240	21.345	893302	6.83	330070	10.44	2.71		(Z,E)-2,13-Octadecadienol
22	21.435	21.395	21.560	656977	5.02	211589	6.69	3.10	SV	Octadecanol
23	22.187	22.135	22.250	244326	1.87	42872	1.36	5.70	V	(Z)-9-Octadecenoic acid
24	24.689	24.625	24.765	305758	2.34	98437	3.11	3.11	V	Tricosane
25	28.988	28.895	29.085	280352	2.14	60380	1.91	4.64		Pentacosane
26	35.919	35.755	36.130	1097682	8.39	145072	4.59	7.57		Heptacosane
27	40.949	40.845	41.075	191489	1.46	22607	0.71	8.47	V	Octacosane
28	42.540	42.340	42.765	1181558	9.03	115344	3.65	10.24		Squalene
29	47.470	47.205	47.765	4022895	30.75	328211	10.38	12.26		Nonacosane
				13081168	100.00	3162392	100.00			

Spectrum

Line#:1 R.Time:11.105(Scan#:1222)
MassPeaks:224
RawMode:Averaged 11.100-11.110(1221-1223) BasePeak:57(18162)
BG Mode:Calc. from Peak Group 1 - Event 1

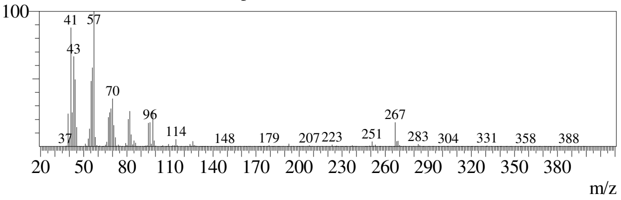

Line#:2 R.Time:11.940(Scan#:1389)
MassPeaks:193
RawMode:Averaged 11.935-11.945(1388-1390) BasePeak:41(2321)
BG Mode:Calc. from Peak Group 1 - Event 1

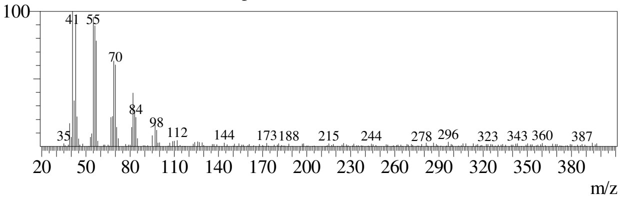

Line#:3 R.Time:12.365(Scan#:1474)
MassPeaks:228
RawMode:Averaged 12.360-12.370(1473-1475) BasePeak:41(16838)
BG Mode:Calc. from Peak Group 1 - Event 1

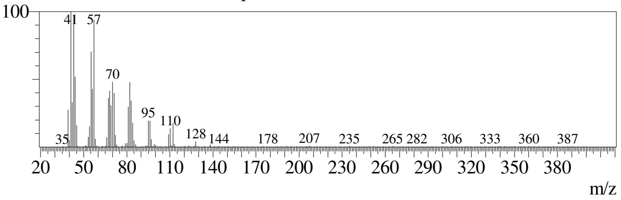

Line#:4 R.Time:12.965(Scan#:1594)
MassPeaks:201
RawMode:Averaged 12.960-12.970(1593-1595) BasePeak:60(3086)
BG Mode:Calc. from Peak Group 1 - Event 1

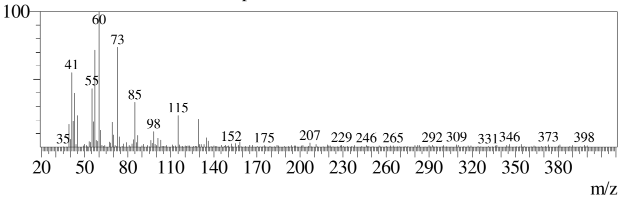

Line#:5 R.Time:13.005(Scan#:1602)
MassPeaks:176
RawMode:Averaged 13.000-13.010(1601-1603) BasePeak:41(921)
BG Mode:Calc. from Peak Group 1 - Event 1

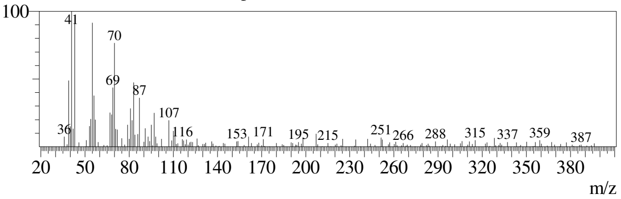

Line#:6 R.Time:13.425(Scan#:1686)
MassPeaks:198
RawMode:Averaged 13.420-13.430(1685-1687) BasePeak:57(1484)
BG Mode:Calc. from Peak Group 1 - Event 1

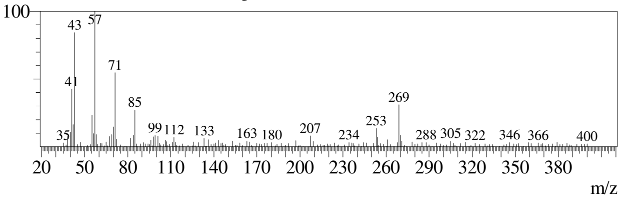

Line#:7 R.Time:13.610(Scan#:1723)
MassPeaks:174
RawMode:Averaged 13.605-13.615(1722-1724) BasePeak:81(5325)
BG Mode:Calc. from Peak Group 1 - Event 1

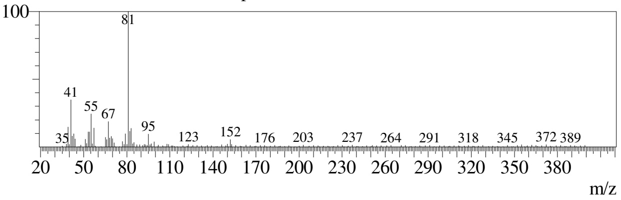

Line#:8 R.Time:14.085(Scan#:1818)
MassPeaks:190
RawMode:Averaged 14.080-14.090(1817-1819) BasePeak:43(1113)
BG Mode:Calc. from Peak Group 1 - Event 1

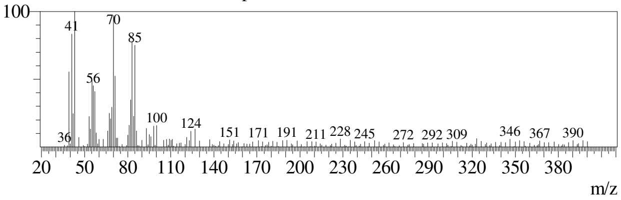

Line#:9 R.Time:14.525(Scan#:1906)
MassPeaks:190
RawMode:Averaged 14.520-14.530(1905-1907) BasePeak:43(1583)
BG Mode:Calc. from Peak Group 1 - Event 1

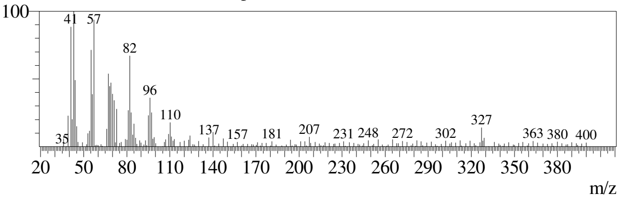

Line#:10 R.Time:14.955(Scan#:1992)
MassPeaks:206
RawMode:Averaged 14.950-14.960(1991-1993) BasePeak:43(10756)
BG Mode:Calc. from Peak Group 1 - Event 1

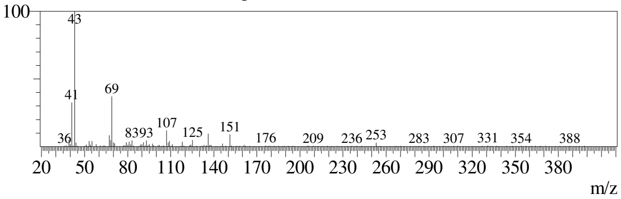

Line#:11 R.Time:15.485(Scan#:2098)
MassPeaks:214
RawMode:Averaged 15.480-15.490(2097-2099) BasePeak:41(1036)
BG Mode:Calc. from Peak Group 1 - Event 1

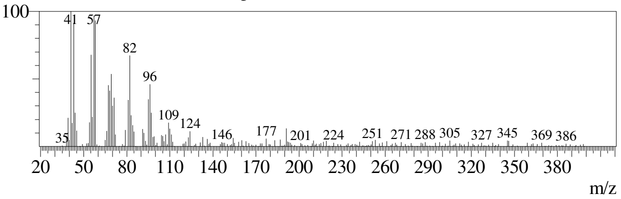

Line#:12 R.Time:16.940(Scan#:2389)
MassPeaks:222
RawMode:Averaged 16.935-16.945(2388-2390) BasePeak:43(1547)
BG Mode:Calc. from Peak Group 1 - Event 1

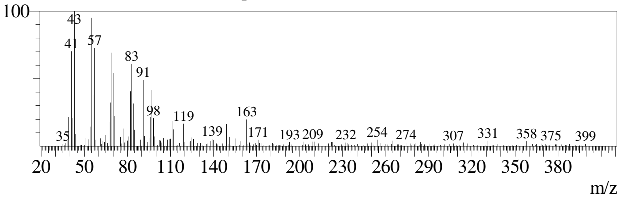

Line#:13 R.Time:17.300(Scan#:2461)
MassPeaks:218
RawMode:Averaged 17.295-17.305(2460-2462) BasePeak:43(2153)
BG Mode:Calc. from Peak Group 1 - Event 1

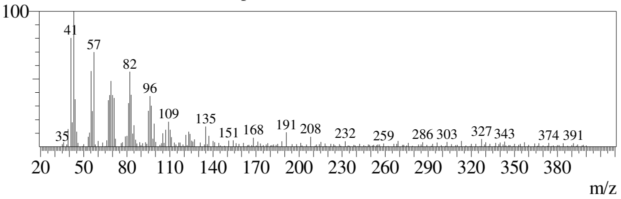

Line#:14 R.Time:17.645(Scan#:2530)
MassPeaks:234
RawMode:Averaged 17.640-17.650(2529-2531) BasePeak:73(3248)
BG Mode:Calc. from Peak Group 1 - Event 1

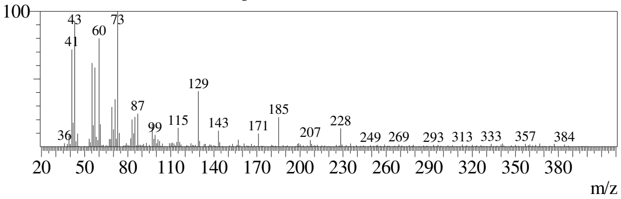

Line#:15 R.Time:18.290(Scan#:2659)
MassPeaks:214
RawMode:Averaged 18.285-18.295(2658-2660) BasePeak:43(3567)
BG Mode:Calc. from Peak Group 1 - Event 1

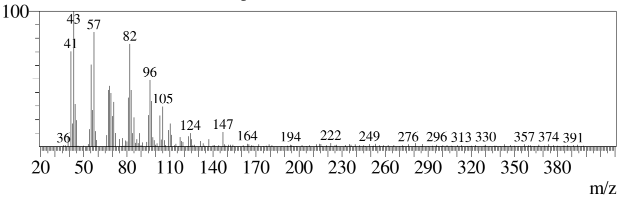

Line#:16 R.Time:18.945(Scan#:2790)
MassPeaks:248
RawMode:Averaged 18.940-18.950(2789-2791) BasePeak:243(24429)
BG Mode:Calc. from Peak Group 1 - Event 1

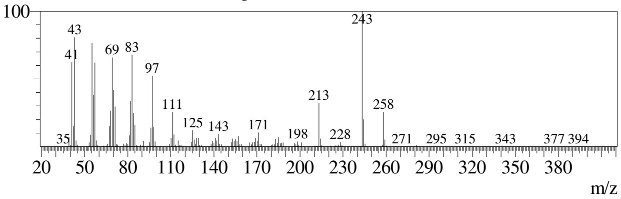

Line#:17 R.Time:19.595(Scan#:2920)
MassPeaks:203
RawMode:Averaged 19.590-19.600(2919-2921) BasePeak:55(782)
BG Mode:Calc. from Peak Group 1 - Event 1

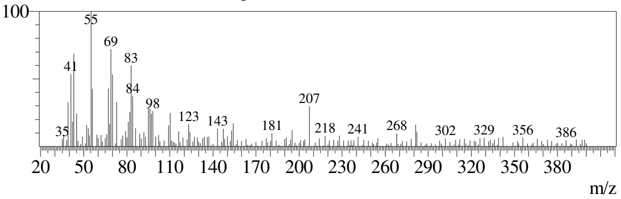

Line#:18 R.Time:19.795(Scan#:2960)
MassPeaks:276
RawMode:Averaged 19.790-19.800(2959-2961) BasePeak:43(42448)
BG Mode:Calc. from Peak Group 1 - Event 1

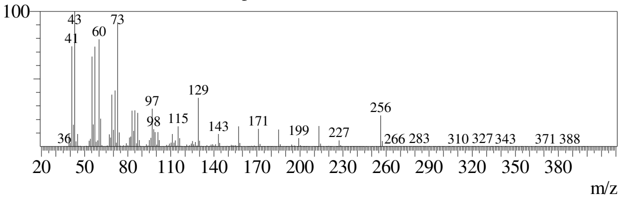

Line#:19 R.Time:20.415(Scan#:3084)
MassPeaks:205
RawMode:Averaged 20.410-20.420(3083-3085) BasePeak:43(1710)
BG Mode:Calc. from Peak Group 1 - Event 1

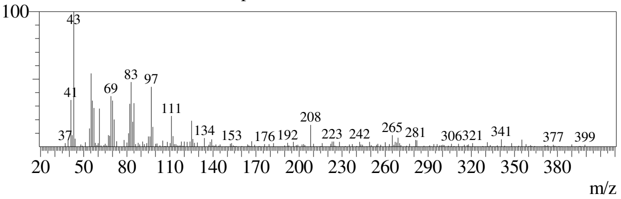

Line#:20 R.Time:20.625(Scan#:3126)
MassPeaks:242
RawMode:Averaged 20.620-20.630(3125-3127) BasePeak:43(2293)
BG Mode:Calc. from Peak Group 1 - Event 1

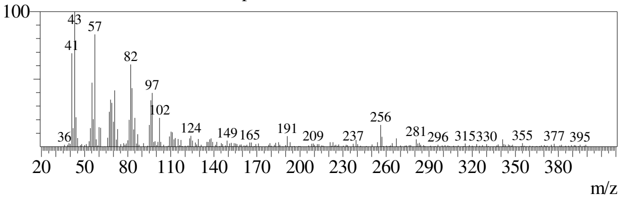

Line#:21 R.Time:21.300(Scan#:3261)
MassPeaks:225
RawMode:Averaged 21.295-21.305(3260-3262) BasePeak:55(28042)
BG Mode:Calc. from Peak Group 1 - Event 1

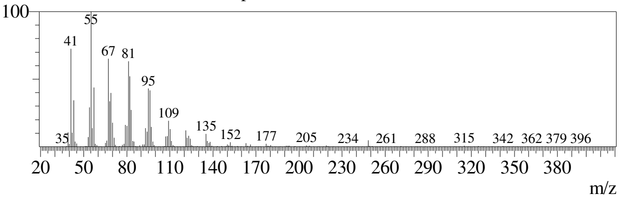

Line#:22 R.Time:21.435(Scan#:3288)
MassPeaks:220
RawMode:Averaged 21.430-21.440(3287-3289) BasePeak:43(15432)
BG Mode:Calc. from Peak Group 1 - Event 1

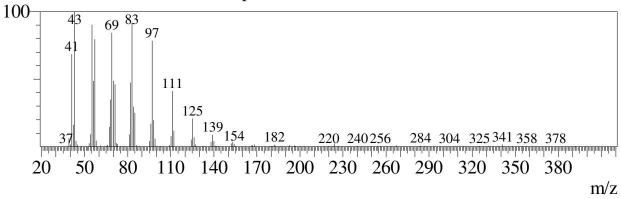

Line#:23 R.Time:22.185(Scan#:3438)
MassPeaks:207
RawMode:Averaged 22.180-22.190(3437-3439) BasePeak:55(1423)
BG Mode:Calc. from Peak Group 1 - Event 1

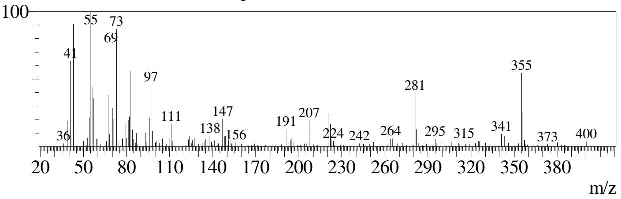

Line#:24 R.Time:24.690(Scan#:3939)
MassPeaks:215
RawMode:Averaged 24.685-24.695(3938-3940) BasePeak:57(17001)
BG Mode:Calc. from Peak Group 1 - Event 1

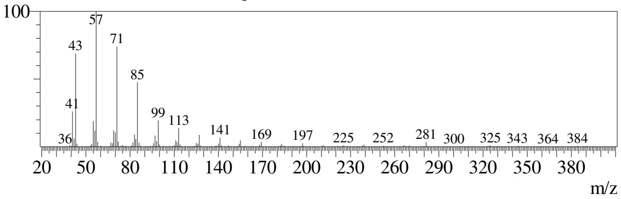

Line#:25 R.Time:28.990(Scan#:4799)
MassPeaks:208
RawMode:Averaged 28.985-28.995(4798-4800) BasePeak:57(10852)
BG Mode:Calc. from Peak Group 1 - Event 1

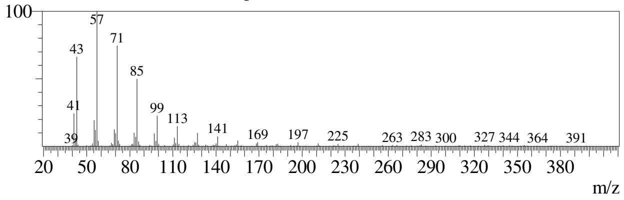

Line#:26 R.Time:35.920(Scan#:6185)
MassPeaks:234
RawMode:Averaged 35.915-35.925(6184-6186) BasePeak:57(24174)
BG Mode:Calc. from Peak Group 1 - Event 1

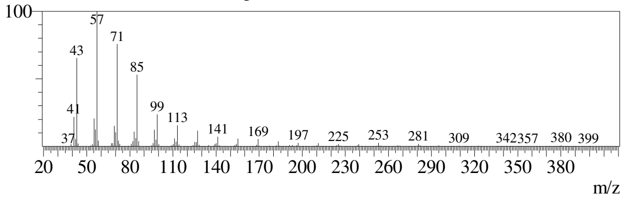

Line#:27 R.Time:40.950(Scan#:7191)
MassPeaks:215
RawMode:Averaged 40.945-40.955(7190-7192) BasePeak:57(2521)
BG Mode:Calc. from Peak Group 1 - Event 1

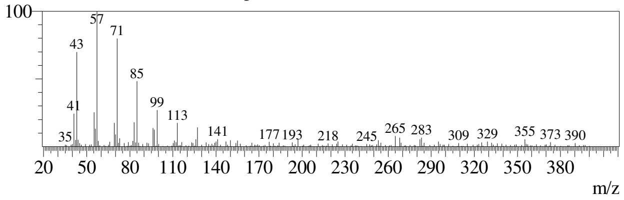

Line#:28 R.Time:42.540(Scan#:7509)
MassPeaks:238
RawMode:Averaged 42.535-42.545(7508-7510) BasePeak:69(28106)
BG Mode:Calc. from Peak Group 1 - Event 1

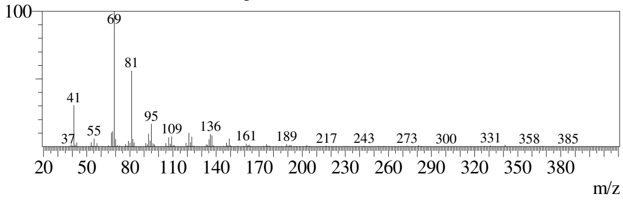

Line#:29 R.Time:47.470(Scan#:8495)
MassPeaks:226
RawMode:Averaged 47.465-47.475(8494-8496) BasePeak:57(55801)
BG Mode:Calc. from Peak Group 1 - Event 1

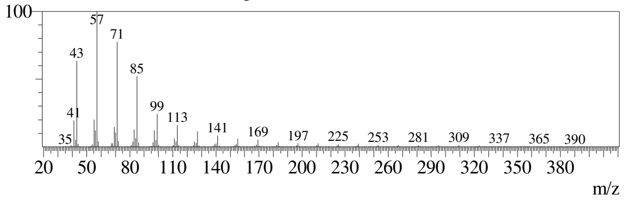

Male forewings extract

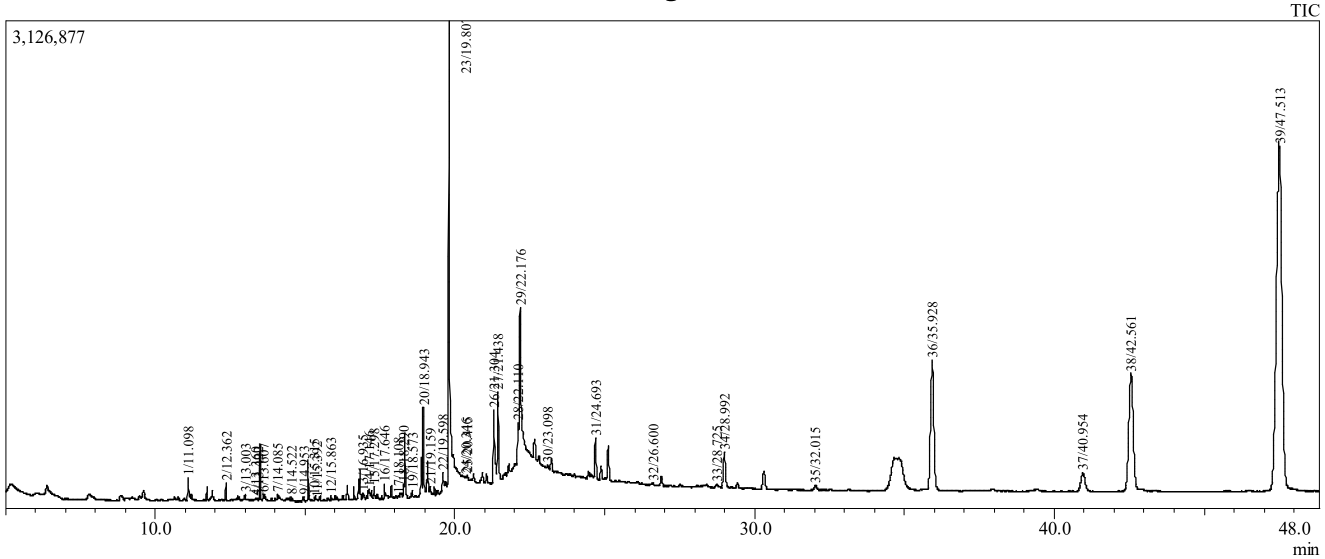

Peak Report TIC

Peak#	R.Time	I.Time	F.Time	Area	Area%	Height	Height%	A/H	Mark	Name
1	11.098	11.025	11.175	416599	0.51	145773	1.20	2.86		Nonanal
2	12.362	12.330	12.410	180396	0.22	111673	0.92	1.62	V	Decanal
3	13.003	12.985	13.030	67067	0.08	38021	0.31	1.76	V	2-Decenal
4	13.360	13.315	13.405	61393	0.08	16243	0.13	3.78	V	2,4-Decadienal
5	13.422	13.405	13.445	28028	0.03	17173	0.14	1.63	V	Tridecane
6	13.607	13.565	13.635	93648	0.12	46144	0.38	2.03	V	Decadienal
7	14.085	14.050	14.100	89405	0.11	47042	0.39	1.90	V	Undecenal
8	14.522	14.495	14.545	49178	0.06	31224	0.26	1.58	V	Dodecanal
9	14.953	14.930	14.975	29613	0.04	19642	0.16	1.51	MI	Geranyl acetone
10	15.315	15.260	15.330	47471	0.06	25089	0.21	1.89	V	Pentadecene
11	15.392	15.375	15.420	31494	0.04	14687	0.12	2.14	V	Pentadecane
12	15.863	15.820	15.950	104863	0.13	34363	0.28	3.05	V	Dodecanoic acid
13	16.935	16.890	16.960	121903	0.15	42510	0.35	2.87	V	Tetradecanol
14	17.146	17.125	17.170	135075	0.17	70836	0.58	1.91	V	2-pentadecanona
15	17.298	17.260	17.370	208639	0.26	82120	0.67	2.54	V	Pentadecanal
16	17.646	17.590	17.715	244059	0.30	98491	0.81	2.48		Tetradecanoic acid
17	18.108	18.065	18.140	56309	0.07	20663	0.17	2.73	V	Octadecane
18	18.290	18.255	18.300	145682	0.18	88208	0.72	1.65	V	Hexadecanal
19	18.573	18.550	18.600	47685	0.06	29644	0.24	1.61	MI	Trimethyl tridecatrienal
20	18.943	18.910	19.020	1376925	1.70	576113	4.73	2.39	V	Hexadecanol
21	19.159	19.135	19.225	196896	0.24	60202	0.49	3.27	V	Heptadecenal
22	19.598	19.560	19.660	531409	0.66	147879	1.21	3.59	V	(Z)-9-Hexadecenoic acid
23	19.807	19.730	21.230	15711475	19.41	3034370	24.92	5.18	SV	Hexadecanoic acid
24	20.346	20.310	20.380	32157	0.04	11923	0.10	2.70	TV	Eicosane
25	20.415	20.380	20.475	66933	0.08	26793	0.22	2.50	TV	Hexadecanyl acetate
26	21.304	21.255	21.345	1244030	1.54	468467	3.85	2.66	MI	(Z,E)-2,13-Octadecadienol
27	21.438	21.395	21.565	2268814	2.80	631802	5.19	3.59	V	Octadecanol
28	22.110	22.040	22.130	1536821	1.90	425600	3.50	3.61	V	(Z,Z)-9,12-Octadecadienoic acid
29	22.176	22.130	22.440	8084611	9.99	1165461	9.57	6.94	V	(Z)-9-Octadecenoic acid
30	23.098	23.055	23.160	785702	0.97	138248	1.14	5.68	V	Docosane
31	24.693	24.625	24.790	1054093	1.30	290528	2.39	3.63	V	Tricosane
32	26.600	26.560	26.670	63603	0.08	15869	0.13	4.01	V	Tetracosane
33	28.725	28.645	28.790	99636	0.12	19060	0.16	5.23		Docosanol
34	28.992	28.865	29.155	1107983	1.37	229828	1.89	4.82	SV	Pentacosane
35	32.015	31.900	32.145	214189	0.26	34087	0.28	6.28	V	Hexacosane
36	35.928	35.750	36.170	6217493	7.68	829454	6.81	7.50		Heptacosane
37	40.954	40.710	41.220	1184540	1.46	117370	0.96	10.09		Octacosane
38	42.561	42.290	42.820	7813184	9.65	755557	6.21	10.34		Squalene
39	47.513	47.130	48.530	29191035	36.07	2216746	18.21	13.17	V	Nonacosane
				80940036	100.00	12174903	100.00			

Spectrum

Line#:1 R.Time:11.100(Scan#:1221)
MassPeaks:213
RawMode:Averaged 11.095-11.105(1220-1222) BasePeak:57(15326)
BG Mode:Calc. from Peak Group 1 - Event 1

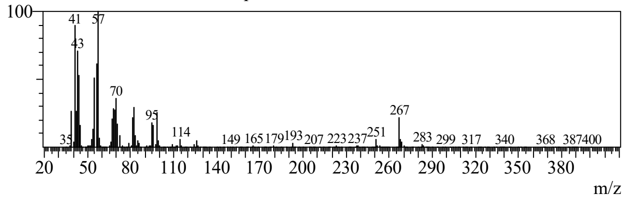

Line#:2 R.Time:12.360(Scan#:1473)
MassPeaks:224
RawMode:Averaged 12.355-12.365(1472-1474) BasePeak:41(9868)
BG Mode:Calc. from Peak Group 1 - Event 1

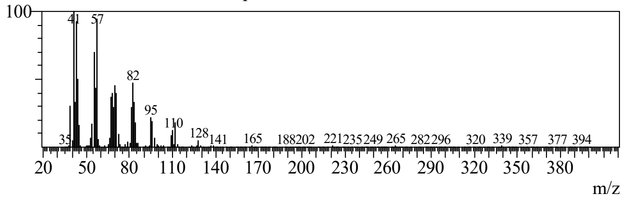

Line#:3 R.Time:13.005(Scan#:1602)
MassPeaks:186
RawMode:Averaged 13.000-13.010(1601-1603) BasePeak:41(2212)
BG Mode:Calc. from Peak Group 1 - Event 1

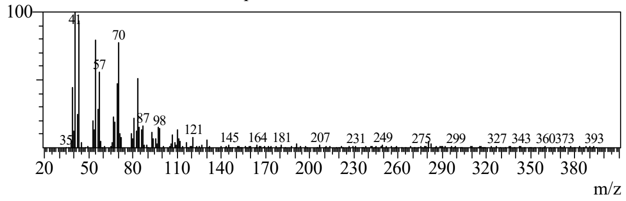

Line#:4 R.Time:13.360(Scan#:1673)
MassPeaks:157
RawMode:Averaged 13.355-13.365(1672-1674) BasePeak:81(3241)
BG Mode:Calc. from Peak Group 1 - Event 1

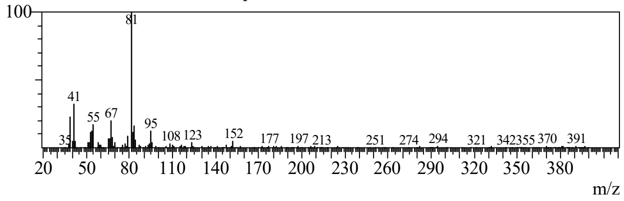

Line#:5 R.Time:13.420(Scan#:1685)
MassPeaks:197
RawMode:Averaged 13.415-13.425(1684-1686) BasePeak:57(2128)
BG Mode:Calc. from Peak Group 1 - Event 1

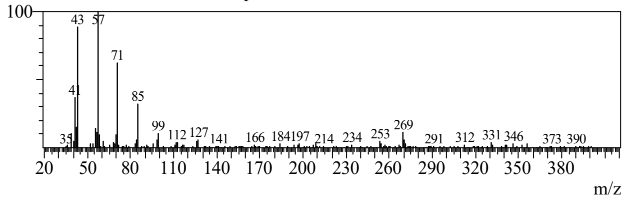

Line#:6 R.Time:13.605(Scan#:1722)
MassPeaks:178
RawMode:Averaged 13.600-13.610(1721-1723) BasePeak:81(9627)
BG Mode:Calc. from Peak Group 1 - Event 1

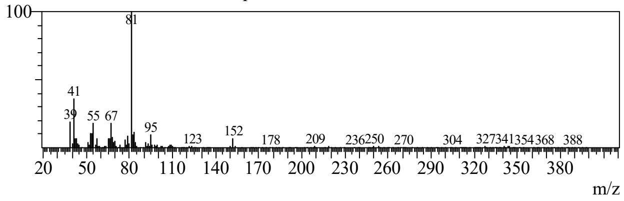

Line#:7 R.Time:14.085(Scan#:1818)
MassPeaks:217
RawMode:Averaged 14.080-14.090(1817-1819) BasePeak:41(2156)
BG Mode:Calc. from Peak Group 1 - Event 1

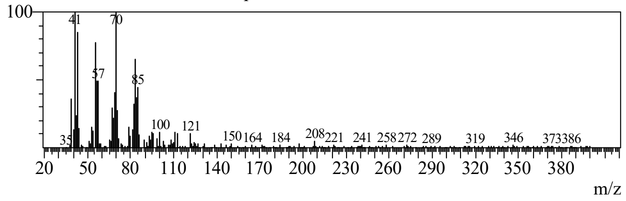

Line#:8 R.Time:14.520(Scan#:1905)
MassPeaks:187
RawMode:Averaged 14.515-14.525(1904-1906) BasePeak:41(2173)
BG Mode:Calc. from Peak Group 1 - Event 1

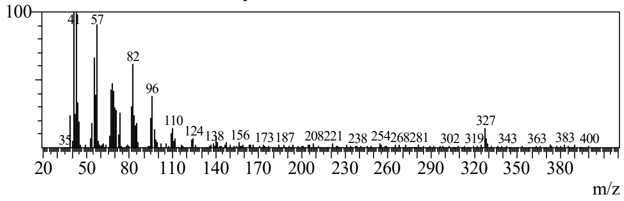

Line#:9 R.Time:14.950(Scan#:1991)
MassPeaks:204
RawMode:Averaged 14.945-14.955(1990-1992) BasePeak:43(5175)
BG Mode:Calc. from Peak Group 1 - Event 1

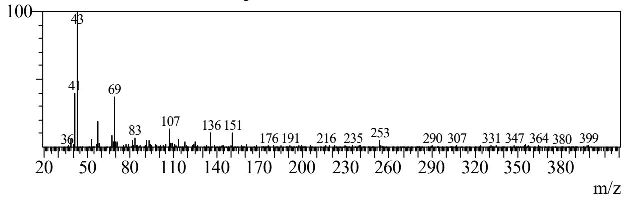

Line#:10 R.Time:15.315(Scan#:2064)
MassPeaks:179
RawMode:Averaged 15.310-15.320(2063-2065) BasePeak:41(1269)
BG Mode:Calc. from Peak Group 1 - Event 1

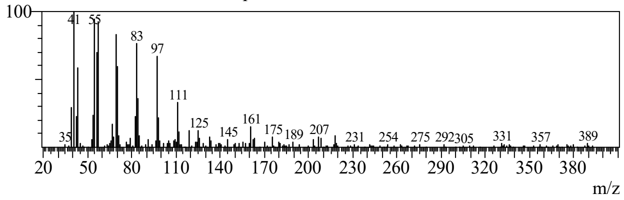

Line#:11 R.Time:15.390(Scan#:2079)
MassPeaks:173
RawMode:Averaged 15.385-15.395(2078-2080) BasePeak:57(1774)
BG Mode:Calc. from Peak Group 1 - Event 1

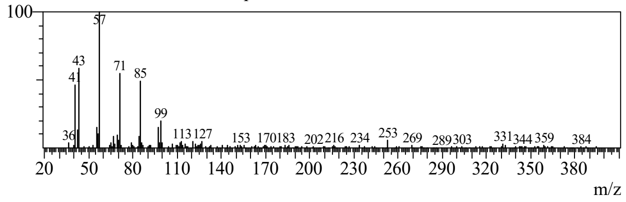

Line#:12 R.Time:15.865(Scan#:2174)
MassPeaks:224
RawMode:Averaged 15.860-15.870(2173-2175) BasePeak:73(3154)
BG Mode:Calc. from Peak Group 1 - Event 1

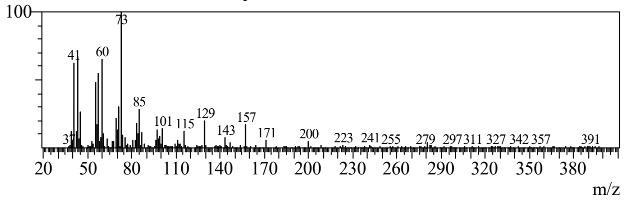

Line#:13 R.Time:16.935(Scan#:2388)
MassPeaks:176
RawMode:Averaged 16.930-16.940(2387-2389) BasePeak:55(2724)
BG Mode:Calc. from Peak Group 1 - Event 1

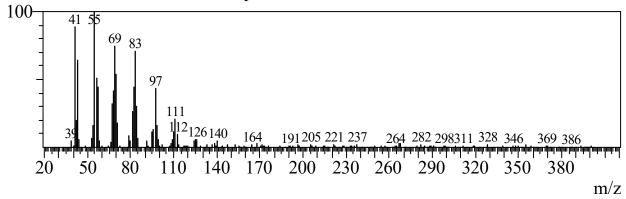

Line#:14 R.Time:17.145(Scan#:2430)
MassPeaks:190
RawMode:Averaged 17.140-17.150(2429-2431) BasePeak:58(8484)
BG Mode:Calc. from Peak Group 1 - Event 1

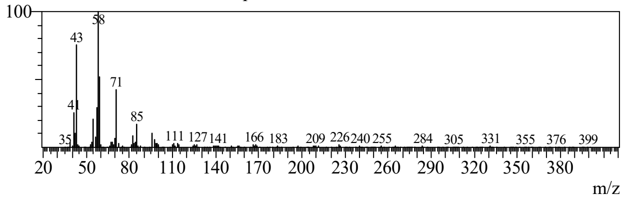

Line#:15 R.Time:17.300(Scan#:2461)
MassPeaks:240
RawMode:Averaged 17.295-17.305(2460-2462) BasePeak:43(6025)
BG Mode:Calc. from Peak Group 1 - Event 1

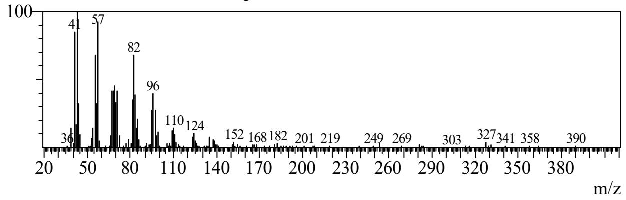

Line#:16 R.Time:17.645(Scan#:2530)
MassPeaks:240
RawMode:Averaged 17.640-17.650(2529-2531) BasePeak:73(8065)
BG Mode:Calc. from Peak Group 1 - Event 1

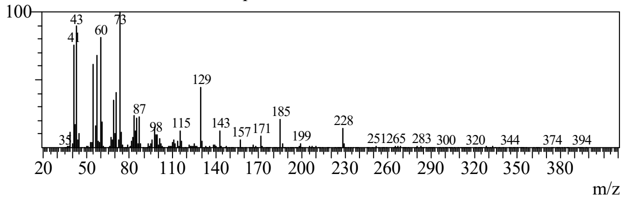

Line#:17 R.Time:18.105(Scan#:2622)
MassPeaks:187
RawMode:Averaged 18.100-18.110(2621-2623) BasePeak:57(2284)
BG Mode:Calc. from Peak Group 1 - Event 1

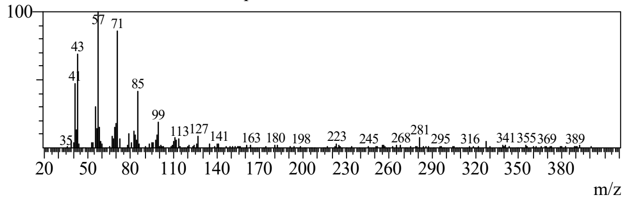

Line#:18 R.Time:18.290(Scan#:2659)
MassPeaks:222
RawMode:Averaged 18.285-18.295(2658-2660) BasePeak:57(2189)
BG Mode:Calc. from Peak Group 1 - Event 1

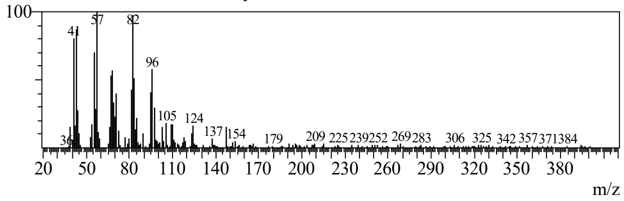

Line#:19 R.Time:18.575(Scan#:2716)
MassPeaks:205
RawMode:Averaged 18.570-18.580(2715-2717) BasePeak:69(7008)
BG Mode:Calc. from Peak Group 1 - Event 1

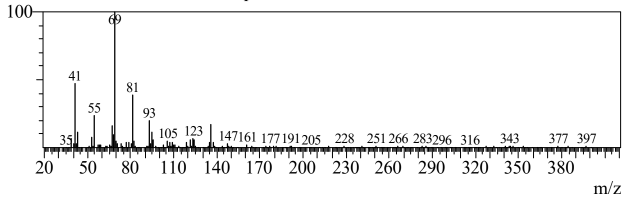

Line#:20 R.Time:18.940(Scan#:2789)
MassPeaks:257
RawMode:Averaged 18.935-18.945(2788-2790) BasePeak:55(39978)
BG Mode:Calc. from Peak Group 1 - Event 1

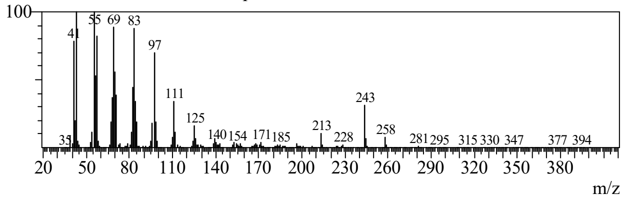

Line#:21 R.Time:19.160(Scan#:2833)
MassPeaks:213
RawMode:Averaged 19.155-19.165(2832-2834) BasePeak:41(2585)
BG Mode:Calc. from Peak Group 1 - Event 1

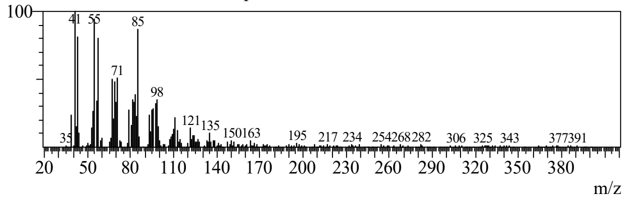

Line#:22 R.Time:19.595(Scan#:2920)
MassPeaks:256
RawMode:Averaged 19.590-19.600(2919-2921) BasePeak:55(9885)
BG Mode:Calc. from Peak Group 1 - Event 1

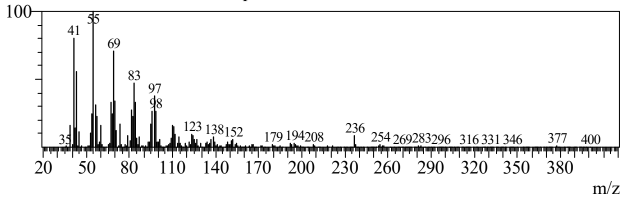

Line#:23 R.Time:19.805(Scan#:2962)
MassPeaks:306
RawMode:Averaged 19.800-19.810(2961-2963) BasePeak:43(248693)
BG Mode:Calc. from Peak Group 1 - Event 1

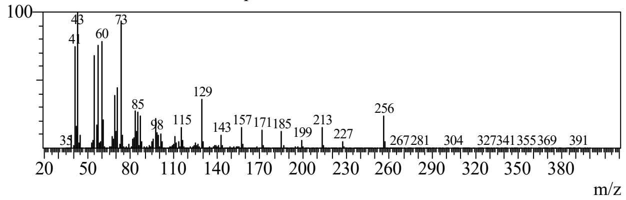

Line#:24 R.Time:20.345(Scan#:3070)
MassPeaks:199
RawMode:Averaged 20.340-20.350(3069-3071) BasePeak:57(1496)
BG Mode:Calc. from Peak Group 1 - Event 1

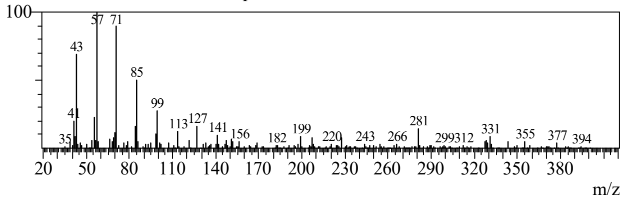

Line#:25 R.Time:20.415(Scan#:3084)
MassPeaks:217
RawMode:Averaged 20.410-20.420(3083-3085) BasePeak:43(2736)
BG Mode:Calc. from Peak Group 1 - Event 1

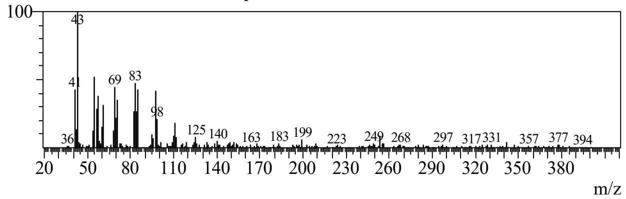

Line#:26 R.Time:21.305(Scan#:3262)
MassPeaks:237
RawMode:Averaged 21.300-21.310(3261-3263) BasePeak:55(39348)
BG Mode:Calc. from Peak Group 1 - Event 1

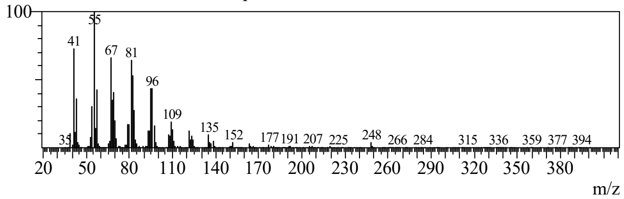

Line#:27 R.Time:21.440(Scan#:3289)
MassPeaks:224
RawMode:Averaged 21.435-21.445(3288-3290) BasePeak:43(44600)
BG Mode:Calc. from Peak Group 1 - Event 1

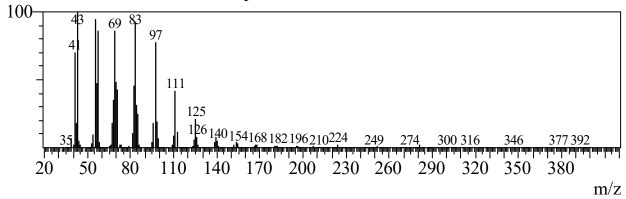

Line#:28 R.Time:22.110(Scan#:3423)
MassPeaks:268
RawMode:Averaged 22.105-22.115(3422-3424) BasePeak:67(7245)
BG Mode:Calc. from Peak Group 1 - Event 1

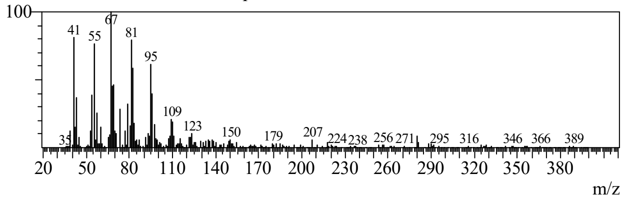

Line#:29 R.Time:22.175(Scan#:3436)
MassPeaks:301
RawMode:Averaged 22.170-22.180(3435-3437) BasePeak:55(63993)
BG Mode:Calc. from Peak Group 1 - Event 1

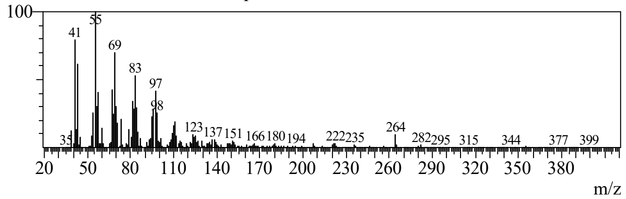

Line#:30 R.Time:23.100(Scan#:3621)
MassPeaks:209
RawMode:Averaged 23.095-23.105(3620-3622) BasePeak:57(3252)
BG Mode:Calc. from Peak Group 1 - Event 1

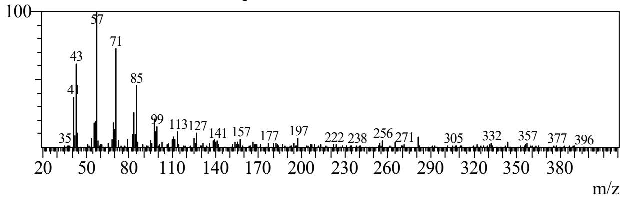

Line#:31 R.Time:24.695(Scan#:3940)
MassPeaks:227
RawMode:Averaged 24.690-24.700(3939-3941) BasePeak:57(45860)
BG Mode:Calc. from Peak Group 1 - Event 1

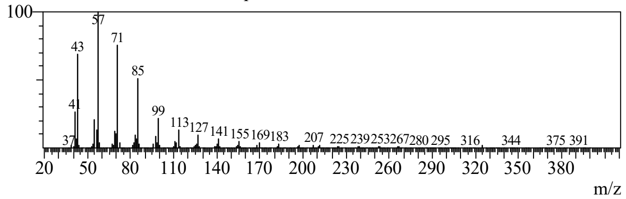

Line#:32 R.Time:26.600(Scan#:4321)
MassPeaks:193
RawMode:Averaged 26.595-26.605(4320-4322) BasePeak:57(1873)
BG Mode:Calc. from Peak Group 1 - Event 1

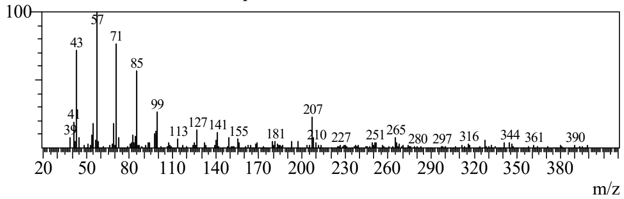

Line#:33 R.Time:28.725(Scan#:4746)
MassPeaks:215
RawMode:Averaged 28.720-28.730(4745-4747) BasePeak:57(1274)
BG Mode:Calc. from Peak Group 1 - Event 1

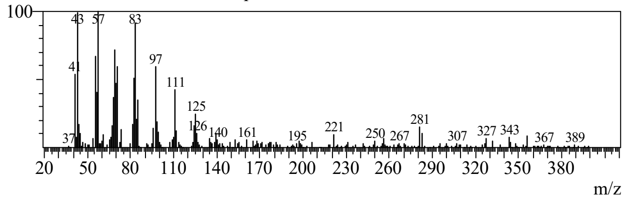

Line#:34 R.Time:28.990(Scan#:4799)
MassPeaks:226
RawMode:Averaged 28.985-28.995(4798-4800) BasePeak:57(40597)
BG Mode:Calc. from Peak Group 1 - Event 1

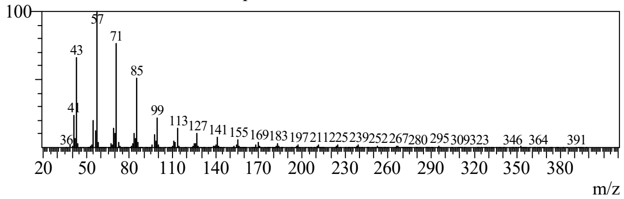

Line#:35 R.Time:32.015(Scan#:5404)
MassPeaks:229
RawMode:Averaged 32.010-32.020(5403-5405) BasePeak:57(5611)
BG Mode:Calc. from Peak Group 1 - Event 1

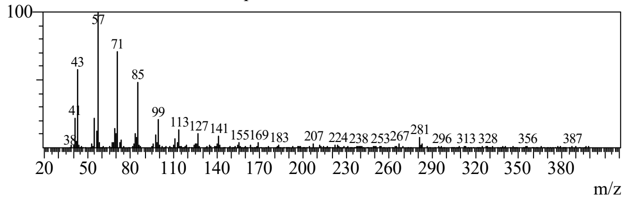

Line#:36 R.Time:35.930(Scan#:6187)
MassPeaks:252
RawMode:Averaged 35.925-35.935(6186-6188) BasePeak:57(140310)
BG Mode:Calc. from Peak Group 1 - Event 1

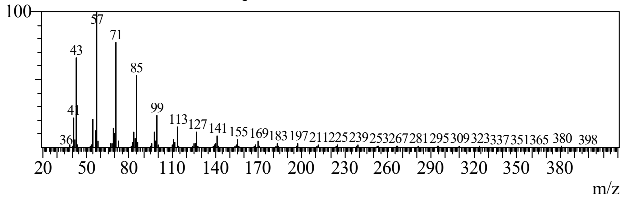

Line#:37 R.Time:40.955(Scan#:7192)
MassPeaks:263
RawMode:Averaged 40.950-40.960(7191-7193) BasePeak:57(19333)
BG Mode:Calc. from Peak Group 1 - Event 1

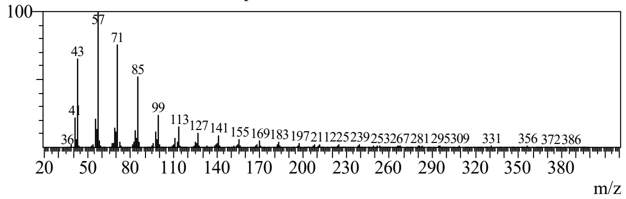

Line#:38 R.Time:42.560(Scan#:7513)
MassPeaks:263
RawMode:Averaged 42.555-42.565(7512-7514) BasePeak:69(185481)
BG Mode:Calc. from Peak Group 1 - Event 1

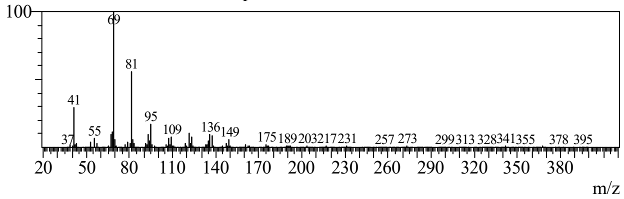

Line#:39 R.Time:47.515(Scan#:8504)
MassPeaks:285
RawMode:Averaged 47.510-47.520(8503-8505) BasePeak:57(367901)
BG Mode:Calc. from Peak Group 1 - Event 1

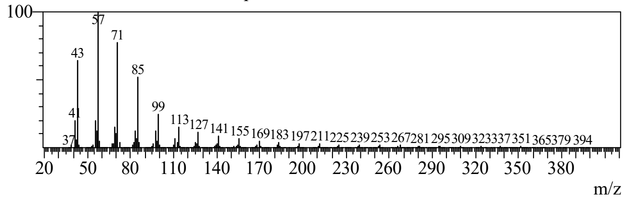

Male legs extract

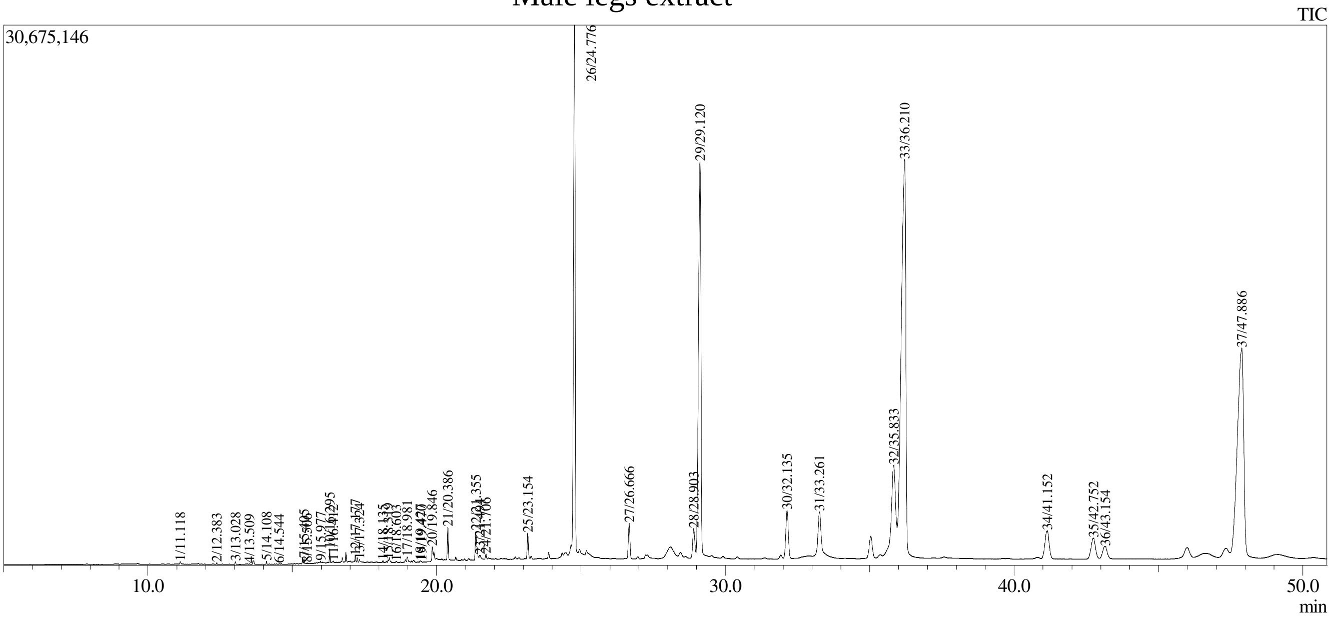

Peak Report TIC

Peak#	R.Time	I.Time	F.Time	Area	Area%	Height	Height%	A/H	Mark	Name
1	11.118	11.080	11.170	289962	0.03	137564	0.12	2.11		Nonanal
2	12.383	12.355	12.420	138220	0.02	89943	0.08	1.54		Decanal
3	13.028	12.995	13.070	214376	0.02	138690	0.12	1.55		2-Decenal
4	13.509	13.480	13.540	49455	0.01	35430	0.03	1.40		Undecanal
5	14.108	14.070	14.145	253167	0.03	171720	0.15	1.47		Undecenal
6	14.544	14.515	14.575	66234	0.01	46466	0.04	1.43		Dodecanal
7	15.405	15.385	15.440	412863	0.05	297126	0.26	1.39	V	Pentadecane
8	15.506	15.470	15.530	61133	0.01	39279	0.03	1.56		Tridecanal
9	15.977	15.955	16.000	161715	0.02	109565	0.10	1.48	V	2-metil-pentadecane
10	16.295	16.255	16.330	1128553	0.13	781833	0.69	1.44	V	Hexadecane
11	16.412	16.330	16.480	300146	0.03	88268	0.08	3.40	V	Tetradecanal
12	17.177	17.145	17.210	507539	0.06	328616	0.29	1.54		Heptadecane
13	17.324	17.285	17.360	248513	0.03	140684	0.12	1.77		Pentadecanal
14	18.135	18.095	18.175	171776	0.02	98859	0.09	1.74		Octadecane
15	18.319	18.285	18.340	295558	0.03	156095	0.14	1.89		Hexadecanal
16	18.603	18.570	18.650	101323	0.01	52023	0.05	1.95		Trimethyl tridecatrienal
17	18.981	18.945	19.065	824364	0.10	291093	0.26	2.83	V	Hexadecanol
18	19.427	19.385	19.460	231015	0.03	90875	0.08	2.54		Heptadecanal
19	19.470	19.460	19.500	53684	0.01	40775	0.04	1.32	V	Methyl hexadecanoate
20	19.846	19.785	19.875	1713549	0.20	791079	0.69	2.17		Hexadecanoic acid
21	20.386	20.325	20.495	4201120	0.49	1865971	1.64	2.25		Eicosane
22	21.355	21.290	21.450	6352407	0.73	1621188	1.42	3.92		(Z,E)-2,13-Octadecadienol
23	21.494	21.450	21.620	1306291	0.15	258849	0.23	5.05	V	Octadecanol
24	21.706	21.620	21.750	838679	0.10	307345	0.27	2.73	V	Heneicosane
25	23.154	23.090	23.225	4186700	0.48	1477283	1.30	2.83		Docosane
26	24.776	24.680	24.890	120567790	13.95	30090948	26.38	4.01	V	Tricosane
27	26.666	26.575	26.770	7761735	0.90	2024047	1.77	3.83		Tetracosane
28	28.903	28.780	28.980	8375194	0.97	1662583	1.46	5.04		Docosanol
29	29.120	28.980	29.360	133223710	15.41	22451616	19.68	5.93	V	Pentacosane
30	32.135	32.005	32.295	16519938	1.91	2739492	2.40	6.03	V	Hexacosane
31	33.261	33.055	33.890	20808265	2.41	2529844	2.22	8.23		Tetracosanal
32	35.833	35.440	35.960	59095297	6.84	5258473	4.61	11.24	V	Tetracosanol
33	36.210	35.960	37.010	249054703	28.81	22504728	19.73	11.07	V	Heptacosane
34	41.152	40.935	41.460	16371233	1.89	1596046	1.40	10.26	V	Octacosane
35	42.752	42.460	42.960	12847726	1.49	1179656	1.03	10.89		Squalene
36	43.154	42.960	43.485	7941565	0.92	710850	0.62	11.17	V	Hexacosanal
37	47.886	47.485	48.485	187829045	21.73	11862667	10.40	15.83	V	Nonacosane
				864504543	100.00	114067569	100.00			

Spectrum

Line#:1 R.Time:11.120(Scan#:1225)
MassPeaks:208
RawMode:Averaged 11.115-11.125(1224-1226) BasePeak:57(14092)
BG Mode:Calc. from Peak Group 1 - Event 1

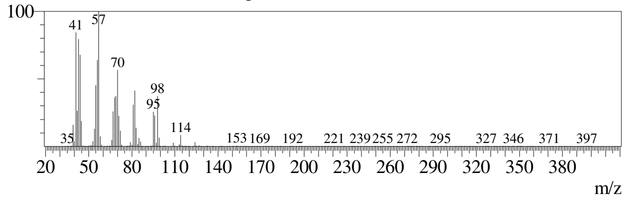

Line#:2 R.Time:12.380(Scan#:1477)
MassPeaks:185
RawMode:Averaged 12.375-12.385(1476-1478) BasePeak:43(7313)
BG Mode:Calc. from Peak Group 1 - Event 1

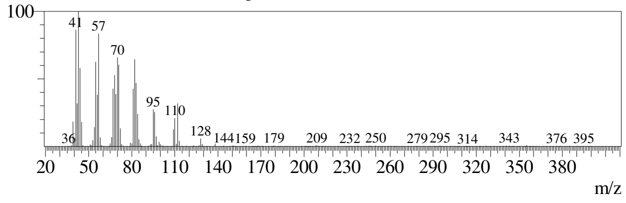

Line#:3 R.Time:13.030(Scan#:1607)
MassPeaks:193
RawMode:Averaged 13.025-13.035(1606-1608) BasePeak:43(13260)
BG Mode:Calc. from Peak Group 1 - Event 1

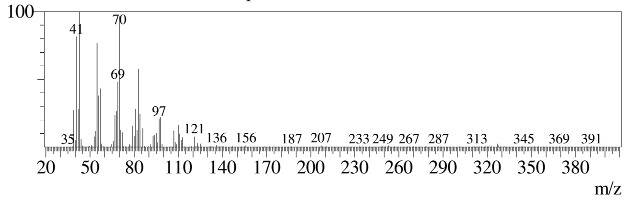

Line#:4 R.Time:13.510(Scan#:1703)
MassPeaks:204
RawMode:Averaged 13.505-13.515(1702-1704) BasePeak:43(2754)
BG Mode:Calc. from Peak Group 1 - Event 1

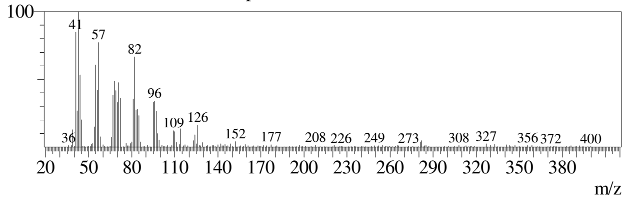

Line#:5 R.Time:14.110(Scan#:1823)
MassPeaks:212
RawMode:Averaged 14.105-14.115(1822-1824) BasePeak:70(15639)
BG Mode:Calc. from Peak Group 1 - Event 1

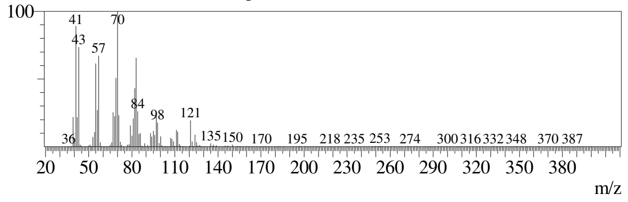

Line#:6 R.Time:14.545(Scan#:1910)
MassPeaks:240
RawMode:Averaged 14.540-14.550(1909-1911) BasePeak:43(3190)
BG Mode:Calc. from Peak Group 1 - Event 1

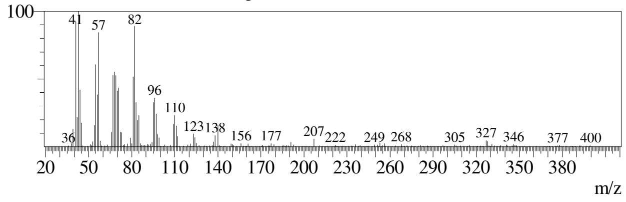

Line#:7 R.Time:15.405(Scan#:2082)
MassPeaks:177
RawMode:Averaged 15.400-15.410(2081-2083) BasePeak:57(45492)
BG Mode:Calc. from Peak Group 1 - Event 1

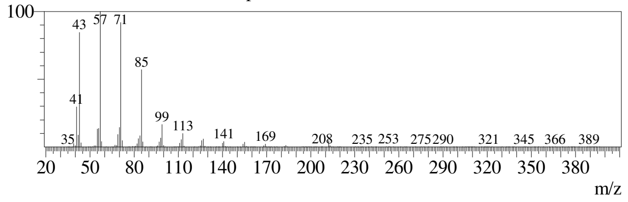

Line#:8 R.Time:15.505(Scan#:2102)
MassPeaks:179
RawMode:Averaged 15.500-15.510(2101-2103) BasePeak:43(3419)
BG Mode:Calc. from Peak Group 1 - Event 1

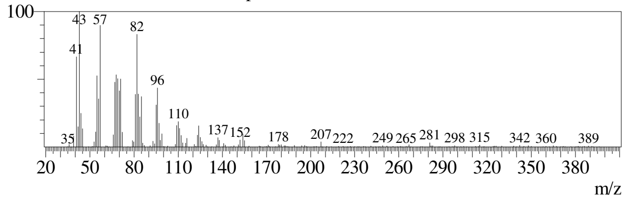

Line#:9 R.Time:15.975(Scan#:2196)
MassPeaks:223
RawMode:Averaged 15.970-15.980(2195-2197) BasePeak:57(12001)
BG Mode:Calc. from Peak Group 1 - Event 1

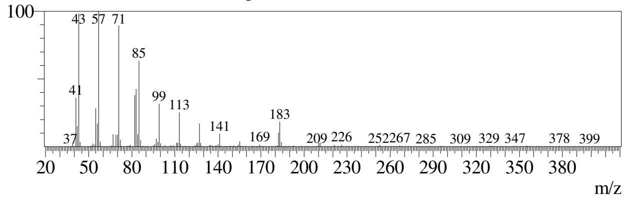

Line#:10 R.Time:16.295(Scan#:2260)
MassPeaks:188
RawMode:Averaged 16.290-16.300(2259-2261) BasePeak:57(126540)
BG Mode:Calc. from Peak Group 1 - Event 1

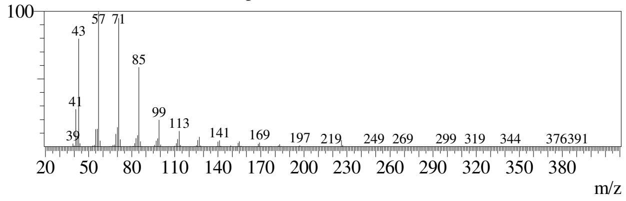

Line#:11 R.Time:16.410(Scan#:2283)
MassPeaks:241
RawMode:Averaged 16.405-16.415(2282-2284) BasePeak:57(6024)
BG Mode:Calc. from Peak Group 1 - Event 1

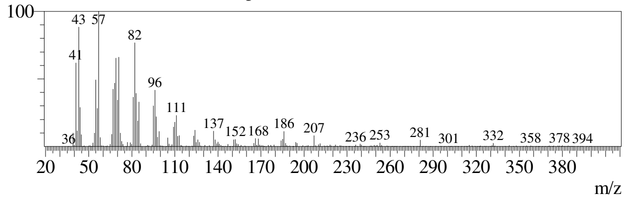

Line#:12 R.Time:17.175(Scan#:2436)
MassPeaks:207
RawMode:Averaged 17.170-17.180(2435-2437) BasePeak:71(51296)
BG Mode:Calc. from Peak Group 1 - Event 1

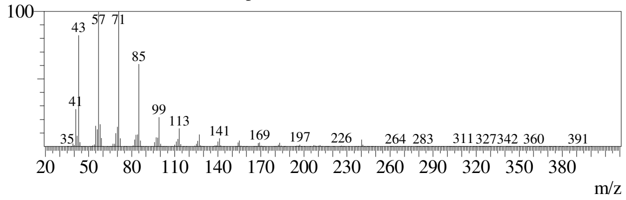

Line#:13 R.Time:17.325(Scan#:2466)
MassPeaks:241
RawMode:Averaged 17.320-17.330(2465-2467) BasePeak:43(10287)
BG Mode:Calc. from Peak Group 1 - Event 1

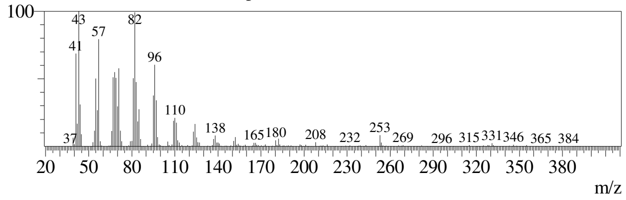

Line#:14 R.Time:18.135(Scan#:2628)
MassPeaks:210
RawMode:Averaged 18.130-18.140(2627-2629) BasePeak:71(14009)
BG Mode:Calc. from Peak Group 1 - Event 1

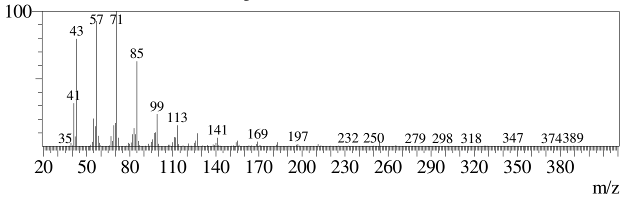

Line#:15 R.Time:18.320(Scan#:2665)
MassPeaks:214
RawMode:Averaged 18.315-18.325(2664-2666) BasePeak:82(9783)
BG Mode:Calc. from Peak Group 1 - Event 1

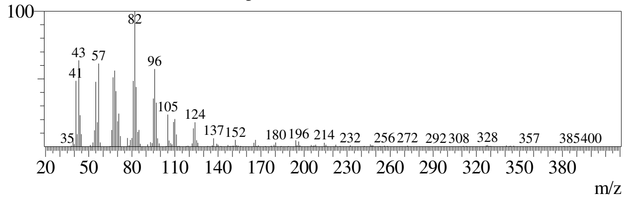

Line#:16 R.Time:18.605(Scan#:2722)
MassPeaks:213
RawMode:Averaged 18.600-18.610(2721-2723) BasePeak:69(12661)
BG Mode:Calc. from Peak Group 1 - Event 1

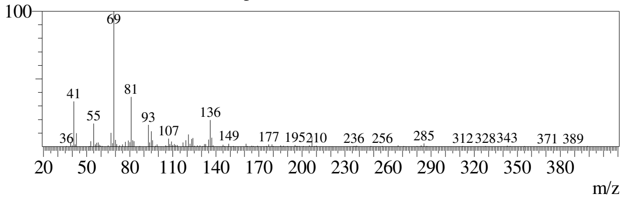

Line#:17 R.Time:18.980(Scan#:2797)
MassPeaks:281
RawMode:Averaged 18.975-18.985(2796-2798) BasePeak:243(18794)
BG Mode:Calc. from Peak Group 1 - Event 1

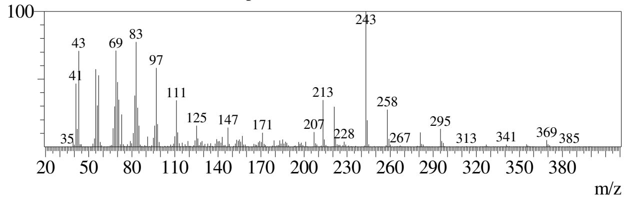

Line#:18 R.Time:19.425(Scan#:2886)
MassPeaks:216
RawMode:Averaged 19.420-19.430(2885-2887) BasePeak:82(6622)
BG Mode:Calc. from Peak Group 1 - Event 1

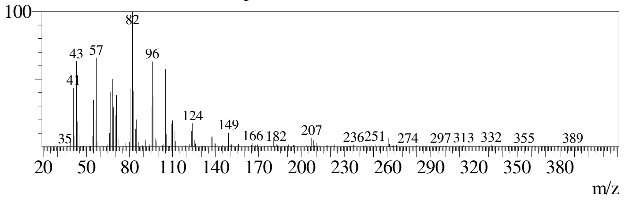

Line#:19 R.Time:19.470(Scan#:2895)
MassPeaks:177
RawMode:Averaged 19.465-19.475(2894-2896) BasePeak:74(585)
BG Mode:Calc. from Peak Group 1 - Event 1

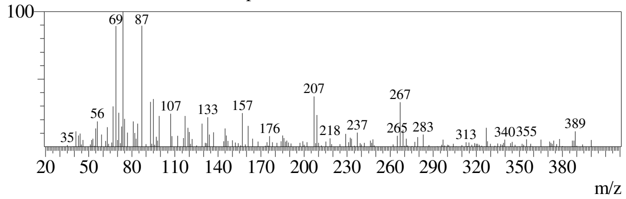

Line#:20 R.Time:19.845(Scan#:2970)
MassPeaks:263
RawMode:Averaged 19.840-19.850(2969-2971) BasePeak:73(51057)
BG Mode:Calc. from Peak Group 1 - Event 1

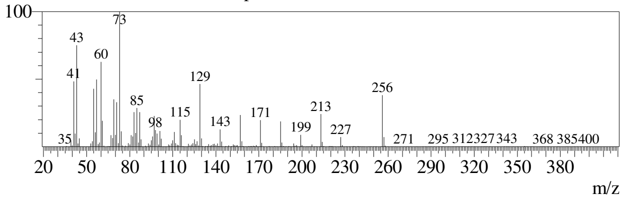

Line#:21 R.Time:20.385(Scan#:3078)
MassPeaks:240
RawMode:Averaged 20.380-20.390(3077-3079) BasePeak:71(284095)
BG Mode:Calc. from Peak Group 1 - Event 1

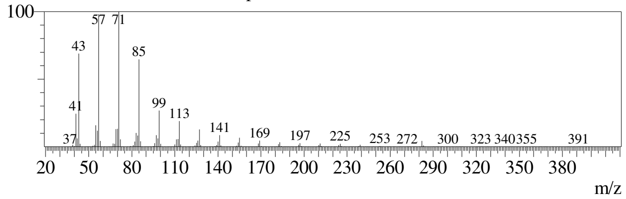

Line#:22 R.Time:21.355(Scan#:3272)
MassPeaks:263
RawMode:Averaged 21.350-21.360(3271-3273) BasePeak:55(115220)
BG Mode:Calc. from Peak Group 1 - Event 1

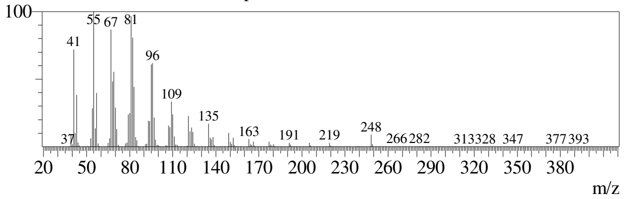

Line#:23 R.Time:21.495(Scan#:3300)
MassPeaks:197
RawMode:Averaged 21.490-21.500(3299-3301) BasePeak:83(15951)
BG Mode:Calc. from Peak Group 1 - Event 1

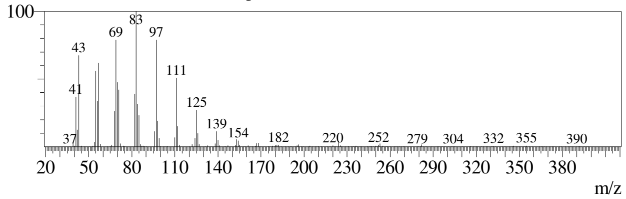

Line#:24 R.Time:21.705(Scan#:3342)
MassPeaks:212
RawMode:Averaged 21.700-21.710(3341-3343) BasePeak:71(43581)
BG Mode:Calc. from Peak Group 1 - Event 1

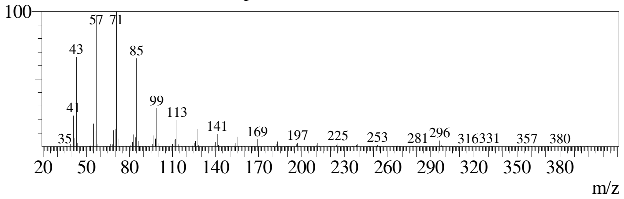

Line#:25 R.Time:23.155(Scan#:3632)
MassPeaks:213
RawMode:Averaged 23.150-23.160(3631-3633) BasePeak:71(210967)
BG Mode:Calc. from Peak Group 1 - Event 1

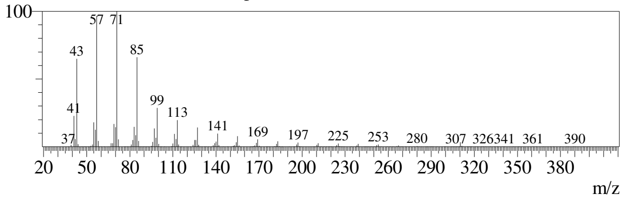

Line#:26 R.Time:24.775(Scan#:3956)
MassPeaks:144
RawMode:Averaged 24.770-24.780(3955-3957) BasePeak:57(4513882)
BG Mode:Calc. from Peak Group 1 - Event 1

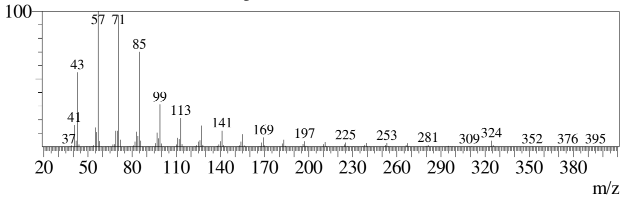

Line#:27 R.Time:26.665(Scan#:4334)
MassPeaks:232
RawMode:Averaged 26.660-26.670(4333-4335) BasePeak:71(301405)
BG Mode:Calc. from Peak Group 1 - Event 1

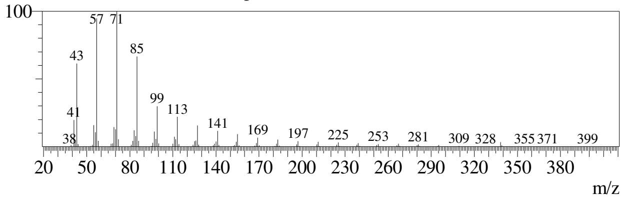

Line#:28 R.Time:28.905(Scan#:4782)
MassPeaks:238
RawMode:Averaged 28.900-28.910(4781-4783) BasePeak:97(128277)
BG Mode:Calc. from Peak Group 1 - Event 1

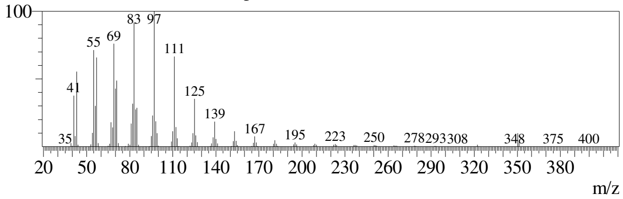

Line#:29 R.Time:29.120(Scan#:4825)
MassPeaks:188
RawMode:Averaged 29.115-29.125(4824-4826) BasePeak:71(3258653)
BG Mode:Calc. from Peak Group 1 - Event 1

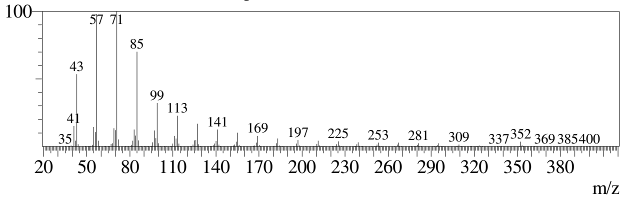

Line#:30 R.Time:32.135(Scan#:5428)
MassPeaks:230
RawMode:Averaged 32.130-32.140(5427-5429) BasePeak:71(396752)
BG Mode:Calc. from Peak Group 1 - Event 1

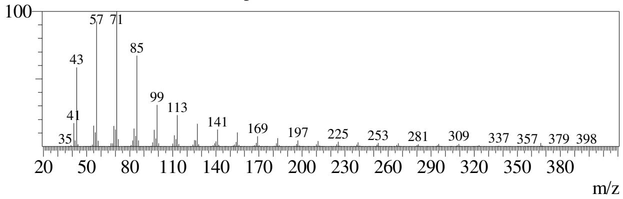

Line#:31 R.Time:33.260(Scan#:5653)
MassPeaks:337
RawMode:Averaged 33.255-33.265(5652-5654) BasePeak:82(201409)
BG Mode:Calc. from Peak Group 1 - Event 1

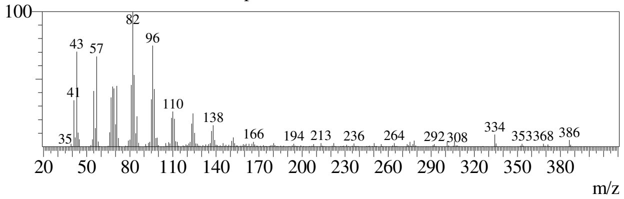

Line#:32 R.Time:35.830(Scan#:6167)
MassPeaks:234
RawMode:Averaged 35.825-35.835(6166-6168) BasePeak:97(336395)
BG Mode:Calc. from Peak Group 1 - Event 1

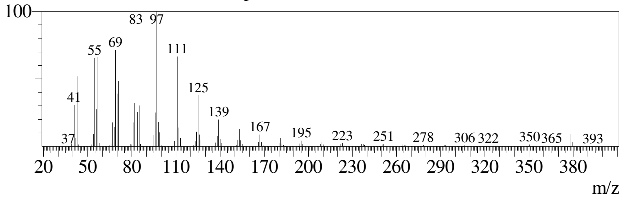

Line#:33 R.Time:36.210(Scan#:6243)
MassPeaks:200
RawMode:Averaged 36.205-36.215(6242-6244) BasePeak:71(2985653)
BG Mode:Calc. from Peak Group 1 - Event 1

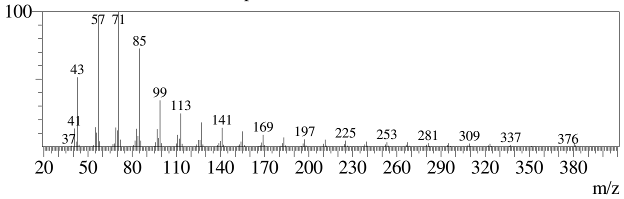

Line#:34 R.Time:41.150(Scan#:7231)
MassPeaks:263
RawMode:Averaged 41.145-41.155(7230-7232) BasePeak:71(224352)
BG Mode:Calc. from Peak Group 1 - Event 1

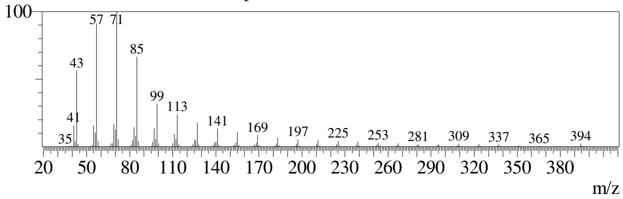

Line#:35 R.Time:42.750(Scan#:7551)
MassPeaks:295
RawMode:Averaged 42.745-42.755(7550-7552) BasePeak:69(263666)
BG Mode:Calc. from Peak Group 1 - Event 1

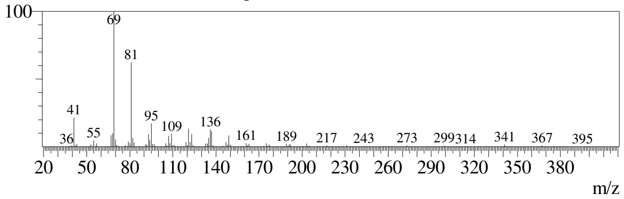

Line#:36 R.Time:43.155(Scan#:7632)
MassPeaks:262
RawMode:Averaged 43.150-43.160(7631-7633) BasePeak:82(60086)
BG Mode:Calc. from Peak Group 1 - Event 1

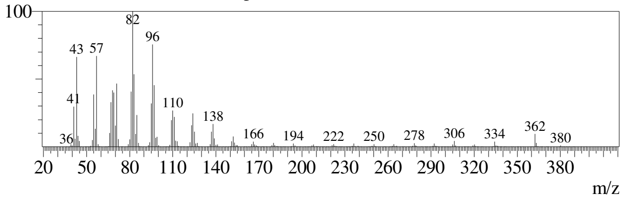

Line#:37 R.Time:47.885(Scan#:8578)
MassPeaks:239
RawMode:Averaged 47.880-47.890(8577-8579) BasePeak:71(1618483)
BG Mode:Calc. from Peak Group 1 - Event 1

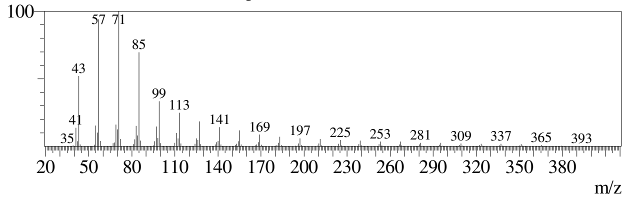

Male genitalia extract

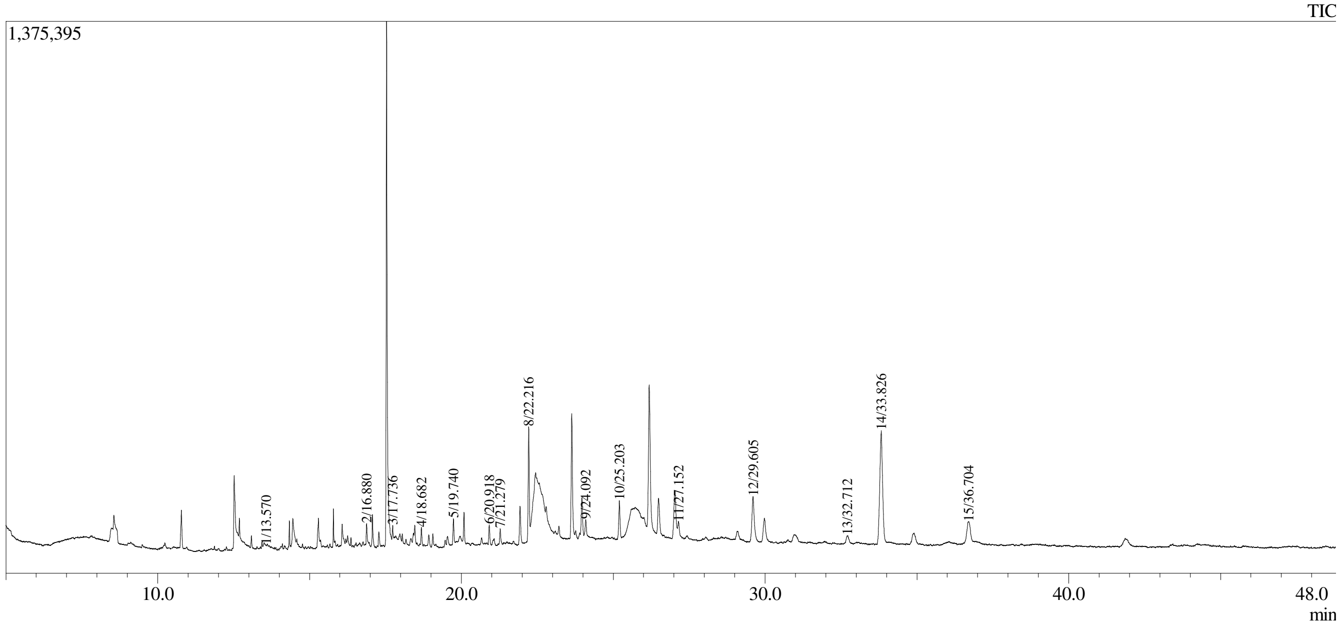

Peak Report TIC

Peak#	R.Time	I.Time	F.Time	Area	Area%	Height	Height%	A/H	Mark	Name
1	13.570	13.560	13.585	19615	0.36	13699	1.07	1.43	V	Dodecanal
2	16.880	16.850	16.945	163198	2.99	61735	4.83	2.64	V	Octadecane
3	17.736	17.705	17.775	72289	1.32	36041	2.82	2.01	T	Nonadecano
4	18.682	18.640	18.740	92753	1.70	45866	3.59	2.02	TV	Eicosane
5	19.740	19.700	19.795	176586	3.24	70321	5.50	2.51	V	Heneicosane
6	20.918	20.875	20.990	121242	2.22	50616	3.96	2.40	V	Docosane
7	21.279	21.220	21.345	123826	2.27	42128	3.29	2.94	V	Eicosanal
8	22.216	22.160	22.265	769048	14.09	292889	22.89	2.63	V	Tricosane
9	24.092	24.030	24.200	173291	3.18	46488	3.63	3.73	V	Docosanal
10	25.203	25.105	25.270	322485	5.91	98460	7.70	3.28	V	Pentacosane
11	27.152	27.110	27.330	258888	4.74	48171	3.77	5.37	V	Hexacosane
12	29.605	29.480	29.810	632761	11.59	112059	8.76	5.65		Heptacosane
13	32.712	32.570	32.910	157516	2.89	21419	1.67	7.35		Octacosane
14	33.826	33.695	33.980	1828978	33.51	281683	22.02	6.49	MI	Squalene
15	36.704	36.340	36.840	544907	9.98	57783	4.52	9.43	V	Nonacosane
				5457383	100.00	1279358	100.00			

Spectrum

Line#:1 R.Time:13.570(Scan#:1715)
MassPeaks:188
RawMode:Averaged 13.565-13.575(1714-1716) BasePeak:44(479)
BG Mode:Calc. from Peak Group 1 - Event 1

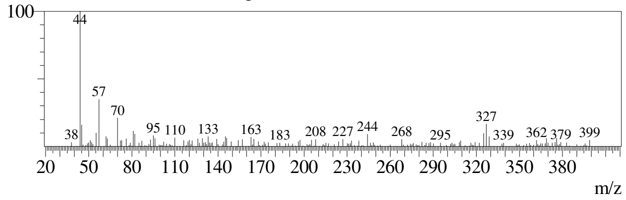

Line#:2 R.Time:16.880(Scan#:2377)
MassPeaks:184
RawMode:Averaged 16.875-16.885(2376-2378) BasePeak:57(9588)
BG Mode:Calc. from Peak Group 1 - Event 1

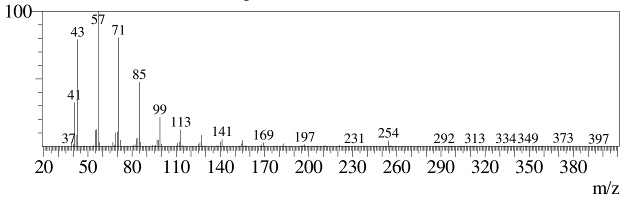

Line#:3 R.Time:17.735(Scan#:2548)
MassPeaks:189
RawMode:Averaged 17.730-17.740(2547-2549) BasePeak:57(6343)
BG Mode:Calc. from Peak Group 1 - Event 1

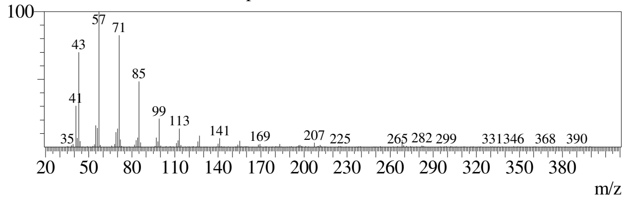

Line#:4 R.Time:18.680(Scan#:2737)
MassPeaks:201
RawMode:Averaged 18.675-18.685(2736-2738) BasePeak:57(7511)
BG Mode:Calc. from Peak Group 1 - Event 1

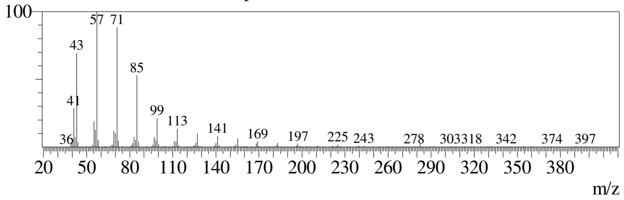

Line#:5 R.Time:19.740(Scan#:2949)
MassPeaks:196
RawMode:Averaged 19.735-19.745(2948-2950) BasePeak:57(10969)
BG Mode:Calc. from Peak Group 1 - Event 1

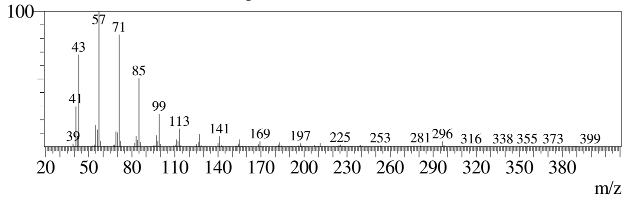

Line#:6 R.Time:20.920(Scan#:3185)
MassPeaks:198
RawMode:Averaged 20.915-20.925(3184-3186) BasePeak:57(8502)
BG Mode:Calc. from Peak Group 1 - Event 1

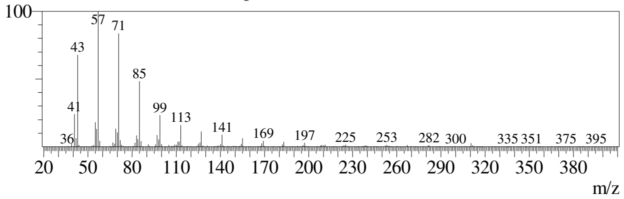

Line#:7 R.Time:21.280(Scan#:3257)
MassPeaks:235
RawMode:Averaged 21.275-21.285(3256-3258) BasePeak:43(3189)
BG Mode:Calc. from Peak Group 1 - Event 1

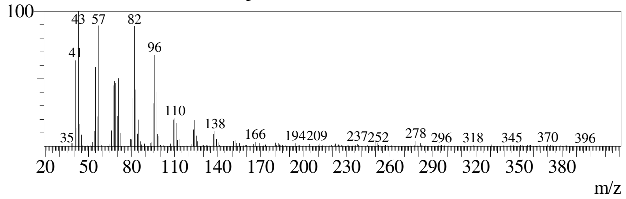

Line#:8 R.Time:22.215(Scan#:3444)
MassPeaks:245
RawMode:Averaged 22.210-22.220(3443-3445) BasePeak:57(42770)
BG Mode:Calc. from Peak Group 1 - Event 1

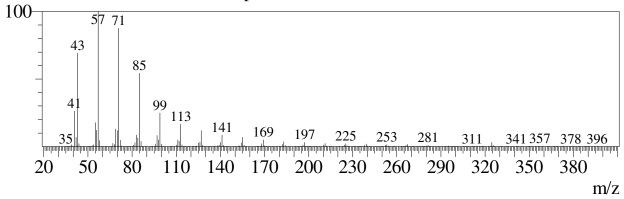

Line#:9 R.Time:24.090(Scan#:3819)
MassPeaks:205
RawMode:Averaged 24.085-24.095(3818-3820) BasePeak:43(3393)
BG Mode:Calc. from Peak Group 1 - Event 1

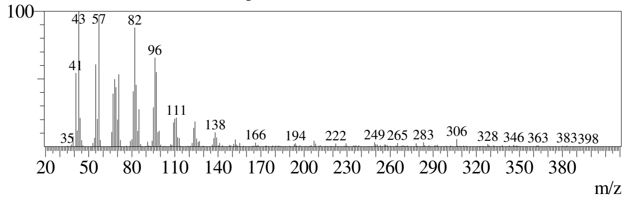

Line#:10 R.Time:25.205(Scan#:4042)
MassPeaks:234
RawMode:Averaged 25.200-25.210(4041-4043) BasePeak:57(14718)
BG Mode:Calc. from Peak Group 1 - Event 1

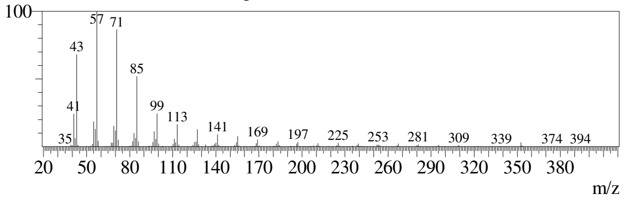

Line#:11 R.Time:27.150(Scan#:4431)
MassPeaks:186
RawMode:Averaged 27.145-27.155(4430-4432) BasePeak:57(4329)
BG Mode:Calc. from Peak Group 1 - Event 1

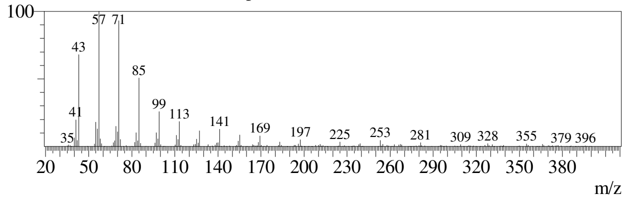

Line#:12 R.Time:29.605(Scan#:4922)
MassPeaks:247
RawMode:Averaged 29.600-29.610(4921-4923) BasePeak:57(17103)
BG Mode:Calc. from Peak Group 1 - Event 1

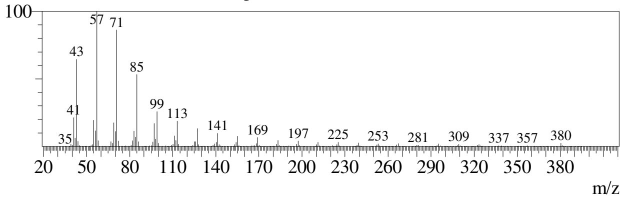

Line#:13 R.Time:32.710(Scan#:5543)
MassPeaks:213
RawMode:Averaged 32.705-32.715(5542-5544) BasePeak:57(3139)
BG Mode:Calc. from Peak Group 1 - Event 1

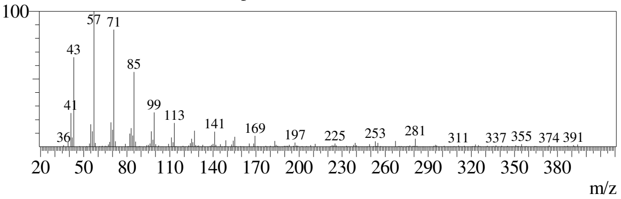

Line#:14 R.Time:33.825(Scan#:5766)
MassPeaks:292
RawMode:Averaged 33.820-33.830(5765-5767) BasePeak:69(66157)
BG Mode:Calc. from Peak Group 1 - Event 1

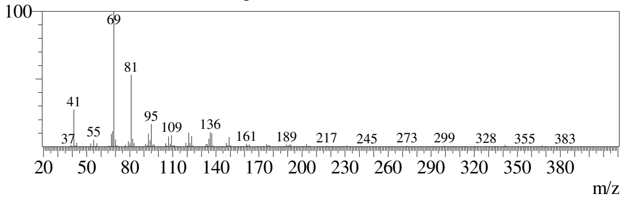

Line#:15 R.Time:36.705(Scan#:6342)
MassPeaks:214
RawMode:Averaged 36.700-36.710(6341-6343) BasePeak:57(8061)
BG Mode:Calc. from Peak Group 1 - Event 1

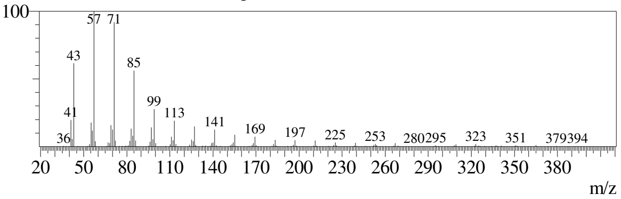

Supplement: S2 File — (PDF) [file pone.0231689.s004.pdf]
