# Supplementary material for: Morphological, chemical and electrophysiological investigations of Telchin licus (Lepidoptera: Castniidae)
Source: PLoS One. 2020 Apr 16;15(4):e0231689. doi: 10.1371/journal.pone.0231689 (PMC7162514; doi:10.1371/journal.pone.0231689)
Supplement: S1 Table — (DOCX) [file pone.0231689.s005.docx]

**S1 Table. Composition of *Telchin licus* male and female body extracts*.***

|  |  |  |  |  |  | **Female** | | | | | | **Male** | | | | | |
| --- | --- | --- | --- | --- | --- | --- | --- | --- | --- | --- | --- | --- | --- | --- | --- | --- | --- |
| **KI^a^** | **Compound** |  |  |  |  | **AB** | **TX** | **HW** | **FW** | **L** | **G** | **AB** | **TX** | **HW** | **FW** | **L** | **G** |
| **HYDROCARBONS** | | **M^+^** | **B** | **M^+^-18** |  |  |  |  |  |  |  |  |  |  |  |  |  |
| 1202 | Dodecane | 170 | 57 |  | Std | + |  | + |  |  |  | + |  |  |  |  |  |
| 1301 | Tridecane | 184 | 57 |  | Std | + |  | + |  | + |  |  | + | + | + |  |  |
| 1401 | Tetradecane | 198 | 57 |  | Std | + |  | + |  | + |  | + | + |  |  |  |  |
| 1493 | Pentadecene | 210 | 69 |  |  | + |  | + | + | + |  | + |  |  | + |  |  |
| 1501 | Pentadecane | 212 | 57 |  | Std | + |  | + | + | + |  | + | + |  | + | + |  |
| 1566 | Metil-pentadecane^♂^ | 226 | 57 |  |  |  |  |  |  |  |  |  | + |  |  | + |  |
| 1602 | Hexadecane | 226 | 57 |  | Std |  |  | + |  |  |  | + | + |  |  | + |  |
| 1702 | Heptadecane | 240 | 71 |  | Std |  | + |  |  |  |  | + | + |  |  | + |  |
| 1802 | Octadecane | 254 | 71 |  | Std |  |  |  | + |  |  | + | + |  | + | + | + |
| 1901 | Nonadecane^♂^ | 268 | 57 |  | Std |  |  |  |  |  |  | + |  |  |  |  | + |
| 2001 | Eicosane | 282 | 71 |  | Std |  |  | + | + | + |  | + | + |  | + | + | + |
| 2101 | Heneicosane | 296 | 71 |  | Std | + | + | + | + | + | + | + | + |  |  | + | + |
| 2201 | Docosane | 310 | 71 |  | Std | + | + | + | + | + |  | + | + |  | + | + | + |
| 2302 | Tricosane | 324 | 57 |  | Std | ++ | +++ | +++ | +++ | ++++ | + | +++ | +++ | + | + | +++ | + |
| 2402 | Tetracosane | 338 | 71 |  | Std |  | + | + | + | + |  | + | + |  | + | + |  |
| 2497 | Pentacosene^♂^ | 350 | 97 |  |  |  |  |  |  |  |  | + |  |  |  |  |  |
| 2502 | Pentacosane | 352 | 57 |  | Std | +++ | +++ | +++ | +++ | +++ | + | +++ | +++ | + | + | +++ | + |
| 2598 | Hexacosene^♂^ | 364 | 97 |  |  |  |  |  |  |  |  | + |  |  |  |  |  |
| 2602 | Hexacosane | 366 | 71 |  | Std | + | + | + | + | + |  | +++ | + |  | + | + | + |
| 2703 | Heptacosane | 380 | 71 |  | Std | ++++ | +++ | +++ | +++ | ++++ | ++ | ++++ | ++++ | + | + | +++ | + |
| 2802 | Octacosane | 394 | 71 |  | Std | + | + | + | + | + |  | +++ | + | + | + | + | + |
| 2900 | Nonacosane | 408 | 71 |  | Std |  | +++ |  | ++++ |  | + | ++++ | +++ | + | ++ | +++ | + |
| **ALDEHYDES** | |  |  |  |  |  |  |  |  |  |  |  |  |  |  |  |  |
| 1001 | Octanal | 128 | 43 | 110 |  | + |  | + | + | + |  | + | + |  |  |  |  |
| 1103 | Nonanal | 142 | 57 | 124 | Std | ++ | + | + | + | + | + | + | + | + | + | + |  |
| 1161 | 2-Nonenal | 140 | 70 | 122 | Std | + | + | + |  | + |  | + | + |  |  |  |  |
| 1205 | Decanal | 156 | 43 | 138 | Std | + | + | + | + | + | + | + | + | + | + | + |  |
| 1263 | 2-Decenal | 154 | 70 | 136 | Std | + | + | + | + | + |  | + | + | + | + |  |  |
| 1296 | 2,4-Decadienal^♂^ | 152 | 81 |  | Std |  |  |  |  |  |  | + | + |  | + |  |  |
| 1307 | Undecanal | 170 | 43 | 152 |  | + | + | + | + | + | + | + | + |  |  | + |  |
| 1319 | Decadienal | 152 | 81 |  |  | + |  | + |  | + |  |  | + | + | + |  |  |
| 1366 | Undecenal | 168 | 70 | 150 |  | + | + | + |  | + |  | + | + | + | + |  |  |
| 1410 | Dodecanal | 184 | 43 |  |  | + | + | + | + | + | + | + | + | + | + | + | + |
| 1513 | Tridecanal | 198 | 43 |  |  | + | + | + | + | + |  | + | + | + |  | + |  |
| 1615 | Tetradecanal | 212 | 43 |  |  | + | + |  |  |  |  | + | + |  |  | + |  |
| 1675 | Tetradecenal^♀^ | 210 | 41 | 192 |  | + |  |  |  |  |  |  |  |  |  |  |  |
| 1716 | Pentadecanal | 226 | 82 | 208 |  | + | + | + | + | + | + | + | + | + | + | + |  |
| 1806 | 9-Hexadecenal | 238 | 55 |  |  | + |  |  |  |  |  | + |  |  |  |  |  |
| 1818 | Hexadecanal | 240 | 82 |  |  | +++ | + | ++ |  | + |  |  |  | + | + | + |  |
| 1900 | Heptadecenal | 252 | 41 | 234 |  |  |  | + |  |  |  |  |  |  | + |  |  |
| 1921 | Heptadecanal | 254 | 82 | 236 |  |  |  |  |  |  | + |  |  |  |  | + |  |
| 2001 | 9-Octadecenal^♀^ | 266 | 55 | 248 |  | + |  |  |  |  |  |  |  |  |  |  |  |
| 2022 | Octadecanal | 268 | 82 | 250 |  | + |  |  |  |  | + | + |  | + |  |  |  |
| 2126 | Nonadecanal^♂^ | 282 | 82 | 264 |  |  |  |  |  |  |  |  |  |  |  |  |  |
| 2227 | Eicosanal^♂^ | 296 | 82 | 278 |  |  |  |  |  |  |  | + | + |  |  |  | + |
| 2430 | Docosanal^♂^ | 324 | 82 | 306 |  |  |  |  |  |  |  | + |  |  |  |  | + |
| 2531 | Tricosanal^♂^ | 338 | 82 | 320 |  |  |  |  |  |  |  |  | + |  |  |  |  |
| 2631 | Tetracosanal | 352 | 82 | 334 |  |  |  | + | + | + |  | + | + |  |  | + |  |
| 2834 | Hexacosanal | 380 | 82 | 362 |  |  |  |  | + | + |  | + | + |  |  | + |  |

|  |  |  |  |  |  | **Female** | | | | | | **Male** | | | | | |
| --- | --- | --- | --- | --- | --- | --- | --- | --- | --- | --- | --- | --- | --- | --- | --- | --- | --- |
| **KI^a^** | **Compound** |  |  |  | **^b^** | **AB** | **TX** | **HW** | **FW** | **L** | **G** | **AB** | **TX** | **HW** | **FW** | **L** | **G** |
| **ALCOHOLS** | | **M^+^** | **B** | **M^+^-18** |  |  |  |  |  |  |  |  |  |  |  |  |  |
| 1170 | Nonanol | 144 | 43 |  | Std | + | + | + | + |  | + | + |  | + |  |  |  |
| 1372 | Undecanol^♀^ | 172 | 55 | 154 |  | + |  | + |  | + |  |  |  |  |  |  |  |
| 1474 | Dodecanol^♀^ | 186 | 43 | 168 | Std | + | + | + |  |  |  |  |  |  |  |  |  |
| 1676 | Tetradecanol | 214 | 43 | 196 |  | + | + | + | + | + | + |  |  | + | + |  |  |
| 1880 | Hexadecanol | 242 | 83 | 224 | Std | + | + | + | +++ | + | + |  |  | + | + | + |  |
| 2075 | Octadecanol | 270 | 83 | 252 |  | + | + | ++ | +++ | + | + | + |  | + | + | + |  |
| 2084 | (*Z,E*)/(*E,Z*)-2,13-Octadecadienol | 266 | 55 | 248 | Std |  |  |  |  |  |  | + | + | + | + | +++ |  |
| 2296 | Eicosanol^♂^ | 298 | 97 | 280 |  |  |  |  |  |  |  |  | + |  |  |  |  |
| 2396 | Heneicosanol^♂^ | 312 | 97 | 294 |  |  |  |  |  |  |  |  | + |  |  |  |  |
| 2494 | Docosanol | 326 | 97 | 308 | Std |  |  |  | + |  |  | + | + |  | + | + |  |
| 2596 | Tricosanol^♂^ | 340 | 57 | 322 |  |  |  |  |  |  |  |  | + |  |  |  |  |
| 2698 | Tetracosanol^♂^ |  | 97 | 336 |  |  |  |  |  |  |  | ++ | ++ |  |  | ++ |  |
| 2878 | Hexacosanol^♂^ |  | 97 | 364 |  |  |  |  |  |  |  | + | + |  |  |  |  |
| **CARBOXYLIC ACIDS** | |  |  |  |  |  |  |  |  |  |  |  |  |  |  |  |  |
| 1259 | Nonanoic acid | 158 | 60 |  | Std |  |  |  |  |  |  |  |  | + |  |  |  |
| 1554 | Dodecanoic acid | 200 | 73 |  | Std |  |  |  |  |  |  |  |  |  | + |  |  |
| 1752 | Tetradecanoic acid | 228 | 73 |  | Std |  |  |  |  |  |  |  |  | + | + |  |  |
| 1939 | (*Z*)-9-Hexadecenoic acid | 254 | 69 |  | Std |  |  |  |  |  |  | + |  | + | + |  |  |
| 1956 | Hexadecanoic acid | 256 | 73 |  | Std |  |  |  |  |  |  | + | + | + | + | + |  |
| 2131 | (*Z,Z*)-9,12-Octadecadienoic acid | 280 | 67 |  | Std |  |  |  |  |  |  |  |  |  | + |  |  |
| 2137 | (*Z*)-9-Octadecenoic acid | 282 | 55 |  | Std |  |  |  |  |  |  | + |  | + | + |  |  |
| **KETONES** | |  |  |  |  |  |  |  |  |  |  |  |  |  |  |  |  |
| 1192 | 2-Decanone | 156 | 43 |  |  | + |  | + |  | + |  |  |  |  |  |  |  |
| 1699 | 2-Pentadecanone | 226 | 43 |  |  | + |  | + | + | + | + |  |  |  | + |  |  |
| **ESTERS** | |  |  |  |  |  |  |  |  |  |  |  |  |  |  |  |  |
| 1924 | Methyl hexadecanoate | 270 | 74 |  | Std |  |  |  | + |  |  | + | + |  |  | + |  |
| 2006 | Hexadecanyl acetate | 284 | 43 |  |  |  |  | + | + | + |  |  |  | + | + |  |  |
| **TERPENOIDS** | |  |  |  |  |  |  |  |  |  |  |  |  |  |  |  |  |
| 1455 | Geranyl acetone | 194 | 43 |  | Std | + |  | + | + | + |  | + | + | + | + |  |  |
| 1845 | Trimethyl tridecatrienal | 248 | 69 |  |  | + |  | + | + |  |  |  |  |  | + | + |  |
| 2827 | Squalene | 410 | 69 |  | Std | + | + | ++ | +++ | ++ |  | + | + | + | + | + | + |

^a^ Kovats index measured on an Rtx-5 column.

^b^ Std, identification based on a standard

M^+^: Molecular ion peak; B: base peak; M^+^-18: molecular ion peak - H_2_O

Relative abundance in the abdomen (AB), thorax (TX), hindwings (HW), forewings (FW), legs (L) and genitalia (G).

+ Relative abundance < 0.5%

++ Relative abundance 0.5% - 1%

+++ Relative abundance 1% - 5%

++++ Relative abundance 5% - 12%
